# Global temperature constraints on *Aedes aegypti* and *Ae. albopictus* persistence and competence for dengue virus transmission

**DOI:** 10.1186/1756-3305-7-338

**Published:** 2014-07-22

**Authors:** Oliver J Brady, Nick Golding, David M Pigott, Moritz U G Kraemer, Jane P Messina, Robert C Reiner Jr, Thomas W Scott, David L Smith, Peter W Gething, Simon I Hay

**Affiliations:** 1 Spatial Ecology and Epidemiology Group, Tinbergen Building, Department of Zoology, University of Oxford, South Parks Road, Oxford, United Kingdom; 2 Department of Entomology and Nematology, University of California, Davis, CA, USA; 3 Fogarty International Center, National Institutes of Health, Bethesda, MD 20892, USA; 4 Department of Epidemiology, Johns Hopkins Bloomberg School of Public Health, Baltimore, MD, USA

**Keywords:** *Aedes*, *Aegypti*, *Albopictus*, Temperature, Distribution, Suitability, Competence, Disease model, Map

## Abstract

**Background:**

Dengue is a disease that has undergone significant expansion over the past hundred years. Understanding what factors limit the distribution of transmission can be used to predict current and future limits to further dengue expansion. While not the only factor, temperature plays an important role in defining these limits. Previous attempts to analyse the effect of temperature on the geographic distribution of dengue have not considered its dynamic intra-annual and diurnal change and its cumulative effects on mosquito and virus populations.

**Methods:**

Here we expand an existing modelling framework with new temperature-based relationships to model an index proportional to the basic reproductive number of the dengue virus. This model framework is combined with high spatial and temporal resolution global temperature data to model the effects of temperature on *Aedes aegypti* and *Ae. albopictus* persistence and competence for dengue virus transmission.

**Results:**

Our model predicted areas where temperature is not expected to permit transmission and/or *Aedes* persistence throughout the year. By reanalysing existing experimental data our analysis indicates that *Ae. albopictus*, often considered a minor vector of dengue, has comparable rates of virus dissemination to its primary vector, *Ae. aegypti*, and when the longer lifespan of *Ae. albopictus* is considered its competence for dengue virus transmission far exceeds that of *Ae. aegypti*.

**Conclusions:**

These results can be used to analyse the effects of temperature and other contributing factors on the expansion of dengue or its *Aedes* vectors. Our finding that *Ae. albopictus* has a greater capacity for dengue transmission than *Ae. aegypti* is contrary to current explanations for the comparative rarity of dengue transmission in established *Ae. albopictus* populations. This suggests that the limited capacity of *Ae. albopictus* to transmit DENV is more dependent on its ecology than vector competence. The recommendations, which we explicitly outlined here, point to clear targets for entomological investigation.

## Background

Dengue is the most clinically important viral vector-borne disease with 96 million apparent infections per year among a population-at-risk of nearly four billion in 128 countries [1-3]. During the last century dengue virus (DENV) has rapidly expanded from its tropical origins to sub-tropical and temperate climates. Similarly, the geographic limits of transmission and endemic regions (with year round transmission) continue to expand [4-7]. While significant improvements have been made to our understanding of and our ability to map the current global distribution of dengue [1,2], we do not yet know which factors define the current and future geographic limits to the extent and endemicity of transmission of this still expanding viral infection. These limits are likely defined by a combination of different host (both mosquito and human) and virus factors in different environments. While the study of these factors in isolation is unlikely to define the full constraints of transmission, it nevertheless can identify areas where transmission cannot occur.

Of the many factors affecting dengue transmission, temperature has been most frequently investigated and rigorously quantified [8,9]. A variety of experiments have quantified how key vector and virus dynamics vary with temperature [10,11] and high spatial and temporal resolution meteorological data are available to apply these relationships on a global scale [12]. Using accumulated theoretical and experimental knowledge of the epidemiology of dengue, we use a dengue transmission model to identify where and when temperature is likely to be the most important factor limiting the rate of transmission. Among the limitations temperature can impose on DENV transmission, the most directly affected and most clearly understood mechanisms are its effects on adult female mosquito survivorship and DENV extrinsic incubation period (EIP). Transmission is only permitted when the longevity of the vector exceeds the EIP of the DENV. This is equivalent to the time between the mosquito imbibing an infected blood meal and becoming infectious (able to onwardly transmit the virus by bite). Similarly, a key limitation on vector persistence, the ability for mosquito population dynamics to sustain a population of adult mosquitoes, can be evaluated by determining the proportion of adult female mosquitoes that survive long enough to complete their first gonotrophic cycle (FGC), or the time between taking a blood meal and first oviposition [13]. Evaluating the effects of temperature on these two processes on a population level can determine when and where temperature causes breaks in transmission or vector population persistence. This can also give insights into the relative intensities of transmission and oviposition as if a greater proportion of mosquitoes survive the EIP and FGC they are thus more likely to deliver more infectious bites or oviposit a greater number of eggs respectively.

Evaluating the limits to global DENV transmission is complicated by differences in the global distributions of the DENV’s primary vector, *Aedes aegypti*, and what is often considered a secondary vector, *Ae. albopictus*[14,15]. While *Ae. aegypti* appears to be confined to tropical and subtropical climates [8,16], *Ae. albopictus* eggs can undergo long periods of diapause that allow populations to persist in temperate environments despite winter temperatures that are unsuitable for adult survival. Survival of eggs for relatively long periods of time also allows them to be transported long distances using global travel and trade networks [17,18], resulting in multiple introductions outside their native range into tropical and some temperate environments, most well documented in Europe and North America [17]. The role of *Ae. albopictus* in DENV transmission remains unclear. In areas where it is the only DENV vector, outbreaks are rare and limited in incidence [15,19,20], whereas even recently introduced, isolated populations of *Ae. aegypti* have produced explosive and sustained outbreaks [21,22]. It has been suggested that this difference is primarily due to inter-species differences in the rate of DENV dissemination through the mosquito midgut to the salivary glands, leading to a longer EIP in *Ae. albopictus*[15]. The effect of this difference on transmission has not, however, been quantified taking into account the predicted longer lifespan of *Ae. albopictus*[10]. Alternative explanations involve differences in human blood feeding rates (*Ae. aegypti* has been known to deliver more frequent bites with a higher proportion of blood meals taken from humans than *Ae. albopictus*[23,24]) and possible differences in the proportion of mosquitoes per human resulting from their respective ecologies. *Ae. aegypti* typically inhabits more urban environments than *Ae. albopictus*, and even when both inhabit urban environments, *Ae. aegypti* typically lives in closer proximity to humans [25,26]. While both of these factors are also driven by temperature, they also have strong ecological influences that may in many cases be greater than the effect of temperature. We therefore chose to restrict our analysis to the effects of temperature on the duration of EIP and FGC.

Previous attempts to model the effects of temperature on *Ae. aegypti* and *Ae. albopictus* population persistence and ability to transmit DENV have either taken a statistical or mechanistic (biological or deterministic) modelling approach. Statistical or empirical approaches that attempt to find correlations between various measures of temperature and dengue or vector observations often find weak or only transient associations [27,28]. These approaches cannot be easily designed to include the complexities of the temperature response due to its highly non-linear effects and the dynamic time scale at which it acts [29]. Such limits are often simply defined by a constant mean or minimum temperature that fails to account for seasonal and daily temperature fluctuations throughout the year. Statistical approaches are further restricted in their ability to detect the limits of the distribution due to the inherent scarcity of reported data from environments where transmission or mosquito populations are marginal [30,31]. Conversely, previous mechanistic modelling approaches typically use temperature-based relationships defined by just one or two empirical studies and lack the geographic validation of the statistical approaches [9,32,33]. Furthermore, many studies using mechanistic models are not widely generalisable due to their reliance on locally-specific parameter estimates, particularly estimates of immature mosquito habitat and fluctuations in adult mosquito population sizes. This results in a high degree of uncertainty when trying to evaluate the effects of temperature on the persistence of vector populations and their ability to transmit DENV and this uncertainty is rarely quantified in the final prediction.

In this study we attempt to elucidate the thermal limits of *Ae. aegypti* and *Ae. albopictus* persistence and DENV transmission at a high spatial and temporal resolution using an empirically-parameterised mechanistic model that explicitly considers the interplay between temperature-dependent EIP and adult vector survival. We use existing temperature-based relationships [10,11,34] and define new ones by applying complex modelling techniques to the increasing breadth of data from many different laboratory and field conditions, also including measures of uncertainty in our estimates. These relationships are then combined in a dynamic modelling framework that evaluates the cumulative effects of changing temperature regimes on an index proportional to vectorial capacity. This approach reveals different distinct limits to persistence and virus transmission for each species and goes on to indicate that temperature can also limit levels of endemicity of DENV transmission. In addition to these findings, the predictive maps produced from these models can be used as more biologically-appropriate covariates than typical temperature averages in statistical modelling of dengue and as an informative subcomponent to link vector and human models of DENV transmission.

## Methods

### Relating temperature to transmission potential

Here we adapt a modelling framework from Gething *et al.*[35,36] that evaluates the principal effects of temperature on the basic reproductive number (*R*_0_) of a pathogen. The concept of *R*_0_, the number of secondary cases that will arise from a single infectious human in a totally susceptible population, is a natural framework for evaluating relative changes in transmission potential for a variety of vector borne diseases [37-40]. The entomological, and thus strongly temperature dependent, components of *R*_0_ can be summarised by the equation for vectorial capacity (*V*):

(1) $$V=\;\frac{ma^{2}}{g}e^{-\mathrm{gn}},$$

where *m* is the ratio of adult female mosquitoes to humans, *α* is the human blood feeding rate, *g* is the instantaneous per-capita death rate of adult female mosquitoes and *n* is the length of EIP for the pathogen. All of the parameters in vectorial capacity are likely to be affected to some extent by temperature, either directly or indirectly through interaction with the environment. In our generalised global approach, specific details of the environment are unknown, so we therefore chose to confine our analyses to mosquito mortality (*g*) and pathogen extrinsic incubation period (*n*) due to the direct effects of temperature on these parameters and subsequent potential virus transmission and because of the abundance of information available. If the human population size is stable, *m* can be replaced by two entomological parameters describing mosquito population dynamics: *m* = *λ* / *g*, where *λ* is the emergence rate of new adult mosquitoes. Though mosquito mortality already appears twice in Eq. 1, this substitution reveals a third order effect of mortality on transmission through effects on the adult population size [41] and allows us to define an index of temperature suitability *Z* as a function of temperature, *T*, which is linearly proportional to vectorial capacity over small changes in temperature:

(2) $$Z\left(T\right)=\;\frac{e^{-g\left(T\right)n\left(T\right)}}{g{\left(T\right)}^{2}}\;\propto \;V\left(T\right)=\;\lambda a^{2}\left(\frac{e^{-g\left(T\right)n\left(T\right)}}{g{\left(T\right)}^{2}}\right).$$

*Z*(*T*) therefore effectively measures the relative number of infectious mosquitoes supported in an environment with temperature *T*, given a constant emergence rate *λ* and human blood feeding rate *α*. As estimates for *λ* and *α* are not readily available across the wide range of different environments that exist globally, *Z*(*T*) provides a suitable metric for evaluating the effects of temperature on vectorial capacity, and therefore *R*_0_. While *Z*(*T*) can only measure relative rather than absolute change in vectorial capacity, a value of *Z*(*T*) approximately equal to zero indicates that an environment does not permit onward transmission and can thus be used to predict the geographical limits of transmission.

While *Z*(*T*) can be used to estimate relative suitability for transmission as a function of temperature, a modified version of the model can be used to estimate suitability for the persistence of mosquito populations also as a function of temperature. Rather than surviving long enough to transmit the virus, we need to evaluate whether the mosquito can survive long enough to complete one gonotrophic cycle (*c*), the minimum necessary for reproduction and thus persistence of a permanent mosquito population. A simple substitution of *c* for *n* in equation 2 enables the calculation of temperature suitability for oviposition. This allows us to evaluate the limits temperature places on the persistence of adult *Ae. aegypti* and *Ae. albopictus* populations, assuming the environment can support the juvenile stages of each species:

(3) $$Z_{\mathrm{ovi}}\left(T\right)=\;\frac{e^{-g\left(T\right)c\left(T\right)}}{g{\left(T\right)}^{2}}.$$

### Temperature-based relationships

Calculation of temperature suitability for transmission and vector persistence requires evidence-based models for mosquito mortality, EIP and the length of the FGC. For the first two relationships we draw on two previously published studies investigating the effects of temperature on these parameters and incorporate both mean prediction and the uncertainty in these estimates. For FGC, we apply an existing model to newly available data from a literature review of entomological life cycle experiments.

#### Adult mosquito survivorship

Brady *et al.*[10] used generalised additive model regression to fit an empirical relationship between temperature and adult female survival in both *Ae. albopictus and Ae. aegypti* using over 350 laboratory and 50 field studies. An initial model of survival in a laboratory setting at a range of different temperatures was fitted, whilst accounting for potentially confounding factors. The laboratory survival model was then adapted to estimate survival under field conditions by calculating the additional hazard observed in the field as quantified by mark-release-recapture experiments. Laboratory and field models were derived separately for *Ae. aegypti* and *Ae. albopictus*, but in both mortality was modelled as a function of both temperature (*T*) and mosquito age (*D*):

(4) $$\mathrm{logit}\left(-\ln\left(g_{D,T}\right)\right)=\;f_{s}\left(D,T\right)+\;\gamma_{s}\;,$$

where *f* is a smooth term and *y* is the constant additional field mortality hazard for each mosquito species *s*.

#### *DENV incubation period in* Aedes aegypti

Chan and Johansson [11] used censored Bayesian time-to-event hazard models to parameterise a log-normal relationship between temperature and DENV EIP. This model was fitted to data from 140 mosquito transmission experiments where a cohort of mosquitoes was infected, incubated at constant temperature *T*, then transmission to human or primate hosts monitored at defined time intervals, accounting for inter-study differences in competence, to estimate the expected EIP duration (*n*).

(5) $$n_{T}=\;e^{\mathrm{\beta X}+\frac{1}{2\tau }}$$

(6) $$\mathrm{\beta X}=\;\beta_{0}+\;\beta_{T}T$$

The vast majority of EIP experimental data was conducted using *Ae. aegypti* (96%), while only six experiments concerned EIP in *Ae. albopictus.* As these data were insufficient for detecting EIP differences between the two species, this model was fitted using only experiments that used *Ae. aegypti*, however, this yielded no difference in the fitted parameters than in the model that used data from both species. The final log-normal model was fitted with parameter estimates τ = 4.9, *β*_0_ = 2.9, and *β*_*T*_ = – 0.08.

#### Inter-species differences in DENV incubation period

Few experiments have been conducted that have been designed to accurately measure DENV EIP in *Ae. albopictus.* To account for potential differences in EIPs for *Ae. albopictus*, we assessed data from vector competence assays in which cohorts of mosquitoes are experimentally infected and later assessed for disseminated DENV infection using immunofluorescent assays or similar techniques on cohorts extracted at one or more pre-defined time points.

To collate a comprehensive dataset with which to parameterise this model, databases of published literature (Pubmed and Google Scholar) were searched for the terms “aedes” AND “competence” and relevant data were extracted from the full text articles. Studies were included if DENV was ingested orally, DENV dissemination was measured in the head or salivary gland tissues and the mosquitoes had a laboratory colonisation history of five generations or fewer, as it has been found that long-term laboratory colonisation can increase vector competence [15]. The data extracted were: number infected at the beginning of the experiment, number sampled and number DENV positive at each time points and incubation temperature. We used this data to fit the following hierarchical model:

(7) $$y_{t,T,\;i,j}\;\sim \;\mathrm{Bin}\left(P_{t,T,\;i,j}\;,\;n_{t,\;T,\;i,j}\right)\;,$$

where *y* is the number of mosquitoes of species *j* in study *i* with DENV in their salivary glands at the assay time point *t* after incubation at temperature *T*, *n* is the number of mosquitoes infected at the beginning of the experiment and *P* is the probability of infection which is determined by:

$$P_{t,T,\;i,j}=\Phi \left(t_{i}|\;\mu_{\mathrm{EI}P_{T,i,j}},\;{\sigma_{\mathrm{EIP}}}_{T,\;i,j}^{2}\right)\;,$$

in which Φ is the cumulative distribution function of the Gaussian distribution, *t*_*i*_ is the time in days between the initial infection and the assay, *μ*_*EIP*_ is the mean EIP and *σ*_*EIP*_^2^ is the variance in EIP in each DENV assay experiment. We then used the assay data to calculate species-specific proportional differences (*α*_*j*_) in the mean predicted EIP duration (from the EIP-temperature model, ${\hat{\mu }}_{\mathrm{EI}P_{T}}$), taking into account inter-study variation due to different experimental designs, vector strains and virus types (*ϵ*_*i*_):

$$\mu_{\mathrm{EI}P_{T,i,j}}={\hat{\mu }}_{\mathrm{EI}P_{T}}+\;\alpha_{j}\;.{\hat{\mu }}_{\mathrm{EI}P_{T}}+\;\epsilon_{i}$$

$$\epsilon_{i}\;\sim \;N\left(0,\;{\sigma_{\mathrm{study}}}^{2}\right)$$

$$\ln\left({\sigma_{\mathrm{EIP}}}_{T,\;i,\;j}\right)\;\sim \;N\left(\mu_{\mathrm{assay}},\;{\sigma_{\mathrm{assay}}}^{2}\right)\;.$$

If viral dissemination and infectiousness are equivalent, we would expect *α*_*aegypti*_ = 0, however due to various factors associated with sensitivities to DENV detection, we may expect detection of DENV dissemination to occur after the mosquito becomes infectious (*α*_*aegypti*_ > 0). Alternatively, transmission experiments may produce a higher rate of false-negatives than dissemination assays [42] (*α*_*aegypti*_ < 0). We therefore calculated species-specific values for *α*_*j*_ and took the relative differences in the means to calculate the efficiency adjustment for EIP duration in *Ae. albopictus* (*φ*):

$$\varphi =\frac{{\hat{\mu }}_{\mathrm{EI}P_{T}}+\alpha_{\mathrm{aegypti}}.{\hat{\mu }}_{\mathrm{EI}P_{T}}\;}{{\hat{\mu }}_{\mathrm{EI}P_{T}}+\alpha_{\mathrm{albopictus}}.{\hat{\mu }}_{\mathrm{EI}P_{T}}}=\;\frac{1+\;\alpha_{\mathrm{aegypti}}}{1+\;\alpha_{\mathrm{albopictus}}}\;.$$

The above model was fitted as a hierarchical Bayesian model. A vague zero-mean Gaussian prior was specified for *α*_*j*_. Twenty-eight assay experiments estimated the infected population at multiple time points by taking samples from the overall population. In order to incorporate this information, a cumulative normal distribution function was fitted to these experiments by least squares regression to generate empirical (though weakly informative) priors for *μ*_*assay*_ and *σ*_*assay*_^2^. Finally, we specified weakly informative priors for *σ*_*study*_^2^ based on previous models of DENV EIP [11]. See Additional file 1 for the specific priors used. The model was parameterised by MCMC using WinBUGS [43]. Three Markov chains were run for 10000 iterations each with the first 2000 iterations discarded. Convergence was assessed based on visualisations and the Gelman-Rubin statistic.

#### Length of first gonotrophic cycle

To calculate the relationship between temperature and length of the first gonotrophic cycle we fitted an enzyme kinetics model first suggested by Focks *et al.*[9] to an updated database. The database was updated by searching literature databases spanning 1960-2014 for the terms “aedes” and “oviposition” or “gonotrophic”. Experiments that monitored time of emergence, temperature and time of first oviposition were included as long as the mosquitoes had access to a food source and oviposition substrate. Due to the limited detail with which the data were presented, we were only able to extract mean time to oviposition, thus limiting the scope of our modelling options. The four parameters of the following equation was then optimised by minimising mean-squared error using the “L-BFGS-B” method with no upper or lower bounds in R version 3.0.1 [44]:

$$c\left(T_{k}\right)=\frac{\rho \left(T_{k}/298\right)e^{\left[\left(H_{A}/1.987\right)\left(\left(1/298\right)-\left(1/T_{k}\right)\right)\right]\;}}{1+\;e^{\left[\left(H_{H}/1.987\right)\left(\left(1/T_{0.5}\right)-\left(1/T_{k}\right)\right)\right]}}\;,$$

where *T*_*k*_ is the temperature in °K, and *ρ*, *H*_*A*_, *H*_*H*_ and *T*_0.5_ are parameters to fit. The model was run 500 times with different starting values to ensure global and not local minima were found. Uncertainty in these parameters was estimated using the inverse Hessian of the likelihood surface.

### Calculating temperature suitability at high temporal and spatial resolution

Long term average (1950-2000) interpolated weather station temperature data was obtained from WorldClim in the form of global 5 km × 5 km global rasters containing monthly maximum and minimum temperatures [12]. These were aligned back to back into three repeated years then cubic spline interpolated using the stats package in R to give annual weekly maximum and minimum temperature values pixel-by-pixel. Finally, to simulate the effects of diurnal temperature changes, weekly maximum and minimum temperatures were used to fit a sinusoidal day and exponential night temperature profile for each day of the week [32,45-49]. This resulted in global rasters giving interpolated temperature values at two hour time steps throughout an average year. We limited our predictions of temperature suitability to areas with a maximum monthly temperature exceeding 13°C for *Ae. albopictus* and 14°C for *Ae. aegypti*. These thresholds represent the observed temperatures below which biting and movement behaviours are impaired [8,24,46,49]. This impairment affects survival, blood feeding and larval development in ways that are outside the scope of the mortality models included in this analysis. As such, we do not predict below these temperature limits.

Vectorial capacity, and thus *Z*(*T*), is a measure of transmission potential indexed to the time point when a mosquito becomes infected after blood feeding on an infectious human. To calculate the number of infectious mosquito days that arise from this one event we therefore need to consider: i) mosquito mortality in the time preceding mosquito infection, ii) mosquito mortality and virus EIP proceeding infection but preceding mosquito infectiousness and iii) mosquito mortality after they become infectious. In an environment with constantly changing temperatures it is important to incorporate the sequence and magnitude of temperature changes over time and their resultant effect on *Z*(*T*). In a temporal sense, therefore, *Z*(*T*)_*i*_ measures the relative number of infectious mosquito days that will arise in the future following infection on day *i*.

To capture changes in *Z*(*T*)_*i*_ over time, we used a simulation-based discrete time step approach at a high temporal resolution to estimate the integral of the *Z*(*T*) time series, where *T* is a vector of different temperatures. This modelled adult mosquito population size in three compartments: i) uninfected, ii) infected but not yet infectious and iii) infectious, each made up of cohorts that emerge or are infected at different times. Temperature- and age-dependent death rates were applied to each compartment up until an imposed maximum lifetime of 22 days for *Ae. aegypti* and 65 days for *Ae. albopictus*, by which time best-case survival is below 1% in the mosquito survival model [10]. At each two hour time point a population of 100 adult mosquitoes (note that the choice of emerging population size is arbitrary as it has no effect on the relative measure *Z*(*T*)) was simulated to emerge into the uninfected compartment and all mosquitoes in all compartments were exposed to the mortality rate for that time point. Two days after emergence, when *Aedes* mosquitoes are first capable of blood feeding [50], all surviving mosquitoes in the uninfected compartment become infected. For infected mosquitoes, temperature dependent EIP completion proportion was tracked using equations 5 and 6 for each cohort in the infected compartment. Upon completion of the EIP, mosquitoes were transferred from the infected to infectious compartments then the number of infectious mosquito-days was summed up until the maximum lifetime.

By summing the number of infectious mosquito-days arising from cohorts of different ages all infected at time point *i*, the contribution of time step *i* to future transmission, or *Z*(*T*)_*i*_, can be estimated. From the surveillance perspective, however, it would be useful to know the proportion of vectors infectious *X*_*i*_ at time step *i* given the environmental conditions that have occurred in the time leading up to that point. We therefore also calculate this number as the proportion of mosquitoes from all existing cohorts at time step *i* that are infectious. Each model was initiated with a burn in period exceeding the maximum lifetime of the vector, after which *Z*(*T*)_*i*_ and *X*(*T*)_*i*_ was calculated at two hour time steps from 1^st^ January to 31^st^ December. Equivalent time series for *Z*_*ovi*_(*T*)_*i*_ and *X*_*ovi*_(*T*)_*i*_ were also evaluated using the same approach with three compartments: i) newly emerged, ii) blood-fed but not ovipositing and iii) ovipositing.

### Summarising annual time series

The four time series outputs from the temperature suitability simulation (*Z*(*T*), *X*(*T*), *Z*_*ovi*_(*T*) and *X*_*ovi*_(*T*)) represent subtly different measures of the effect of temperature on dengue transmission and *Aedes* persistence. Over the course of an average year, if we take the sum of all the *Z*(*T*) and *X*(*T*) values for each time step ∑ *Z*(*T*) = ∑ *X*(*T*), and ∑ *Z*_*ovi*_(*T*) = ∑ *X*_*ovi*_(*T*), however, the timing of peaks and troughs will differ. *Z*(*T*)_*i*_ and *Z*_*ovi*_ (*T*)_i_ summarise the contribution of a single event at time *i* to future transmission and oviposition respectively. This is equivalent to the introduction of one infectious human or one emerging population of *Aedes* mosquitoes. By contrast *X*(*T*)_*i*_ and *X*_*ovi*_(*T*)_*i*_ measure the contribution of all past events to suitability at time *i*, which is equivalent to multiple infectious events or emerging mosquito populations having occurred in the past that determine current suitability. Each of these measures has different implications depending on DENV endemicity and *Aedes* persistence status in the environments they summarise. The global distribution of dengue transmission varies by season, however, the global distribution of *Ae. aegypti* and *Ae. albopictus* shows little intra-annual variation mainly due to a limited flight range [8,51]. It follows that DENV transmission can exist in many stable states between sporadic transmission (*Z*(*T*)) and hyperendemicity (*X*(*T*)), however, *Aedes* populations are either absent with a risk of introduction (*Z*_*ovi*_(*T*)), or persist year-round with varying degrees of suitability (*X*_*ovi*_(*T*)). The measures for oviposition therefore reflect more absolute limits, while the measures of transmission document two ends of the spectrum of the many possible transmission intensities.

#### Vector temperature suitability

To determine the thermal limits of *Ae. aegypti* and *Ae. albopictus* persistence, we compared our measures of persistence suitability (*X*_*ovi*_(*T*)) to known geographic occurrences of the vectors. Both *Ae. aegypti* and *Ae. albopictus* populations are robust to brief to medium term periods of environmental unsuitability due to transient behavioural and physiological adaptations at various life-cycle stages [52,53]. To determine the maximum unsuitable time period for each species the number of days in the year where *X*_*ovi*_(*T*)_*i*_ = 0 was calculated for 4053 known geographical occurrences of *Ae. aegypti* and 1459 of *Ae. albopictus*. This database of known *Ae. aegypti* and *Ae. albopictus* occurrence points is composed of precise geographic locations where each species has been identified and reported in peer-reviewed literature sources. Full details of the database sources and extraction are available in Additional file 2. As the majority of these occurrence points only detect presence at one point in time, the database may contain identifications of temporary novel introductions as opposed to confirmed year round persistence of mosquito populations. As these records are likely to exist in the minority given their relative rarity, we chose a threshold of the number of suitable days (*X*_*ovi*_(*T*)_*i*_ > 0) in an average year that incorporated 95% of these known occurrences. Separate thresholds were chosen for each species then the *X*_*ovi*_(*T*) index, normalised between 0 and 1, was displayed inside these limits, to show cumulative temperature suitability from established populations. In subsequent maps of temperature suitability for dengue transmission, the limits to vector establishment were used as a constraint, as significant dengue outbreaks can only be sustained in established vector populations.

#### Exploring spatial and temporal variations in suitability

To compare intra-annual variation in suitability across the globe two separate maps were created summarising the *Z*(*T*) and *X*(*T*) time series. The number of days in a year where *Z*(*T*)_*i*_ > 0 or *X*(*T*)_*i*_ > 0 were calculated and mapped on a continuous scale.

Finally, to further distinguish areas that have year-round temperature suitability for oviposition or transmission we summed the total values of *Z*(*T*) or *Z*_*ovi*_(*T*) for each time step and each vector species. These annual totals were then divided by the maximum pixel value for each species to give a relative index between 0 and 1 that can be compared across geographic areas, but not between species. To compare temperature suitability between species total values were normalised relative to the maximum value in either species.

Weekly average animations were also created to display intra-annual variation in temperature suitability. Animations were created for constrained *X*_*ovi*_(*T*), *Z*(*T*) and *X*(*T*) indices and are available in Additional files 3, 4, 5, 6, 7 and 8.

### Quantifying uncertainty

To quantify uncertainty in our temperature suitability predictions, uncertainty in the individual temperature-based relationships parameterised from empirical data was propagated through the model using a Monte-Carlo method. Global pixels were divided into 10000 bins using k-means clustering based on their annual temperature profiles then, for each bin the pixel closest to the mean temperature regime of all pixels within the bin was selected as representative. The simulation model was re-run 100 times on the 10000 representative pixels but each time using a different set of parameters to define the temperature-based relationships. For each model run the parameters from one of the 200 bootstrap models used to fit the data [10] (selected at random) was used to form the temperature-vector survival relationship. For the temperature-EIP relationship for *Ae. aegypti* we took one sample at random from the posterior distribution of the parameters directly from the MCMC chain which gave us one set of model parameters. For the relative EIP differences between *Ae. aegypti* and *Ae. albopictus*, one sample from the posterior distribution of *φ* was selected at random from the MCMC chains. Parameter estimates for the temperature-time to oviposition relationship were obtained by randomly sampling from a multivariate normal distribution with mean vector given by the optimal parameters and covariance matrix given by the negative inverse Hessian matrix estimated during optimisation. The model for each species was run 100 times with these randomly selected parameters and uncertainty was quantified as the interquartile range of the predictions. The uncertainty analysis was performed at 20 km × 20 km resolution rather than the full 5 km × 5 km resolution of the final model due to computational constraints. Note that the model is entirely based on temperature and so is not scale-dependent.

## Results

### Temperature-based relationships

**Figure 1:**
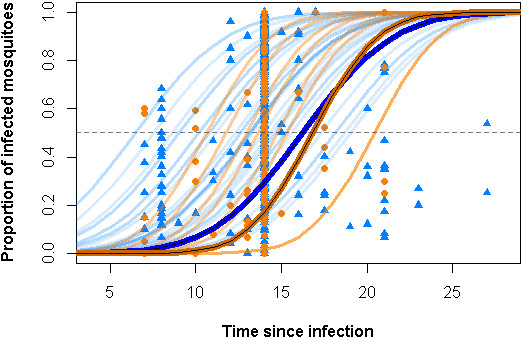
**Rate of incubation in *Ae. aegypti *(blue) and *Ae. albopictus *(orange).** The data from each individual assay are shown by blue triangles for *Ae. aegypti* (n = 373) and orange circles for *Ae. albopictus* (n = 125). The fitted lines for rate of EIP completion for each experiment are shown in faded blue lines for *Ae. aegypti* and faded orange lines for *Ae. alboipictus*. The mean fit for each species is shown in thick solid blue (*Ae. aegypti*) and orange/brown (*Ae. albopictus*) lines. The black dotted line indicates the point at which 50% of the population will have completed incubation, which is equivilent to the mean EIP.

In this analysis we considered three separate temperature-dependent relationships for each of *Ae. aegypti* and *Ae. albopictus*. The relationship between temperature and adult female survival of the two species is available elsewhere [10,54,55]. Similarly, temperature-dependent EIP in *Ae. aegypti* has been modelled previously using data from natural transmission experiments [11], however the corresponding relationship for *Ae. albopictus* was calculated for the first time using assay data collected in this analysis. In total we found 29 references detailing 498 assay time-point measurements that met our inclusion criteria (373 for *Ae. aegypti*, 125 for *Ae. albopictus*). Of the 29 different studies, ten tested the competence of both *Ae. aegypti* and *Ae. albopictus*. Fitting the previously described Bayesian hierarchical model we found a significant difference in mean EIP as determined by transmission experiments and mean EIP as determined by assay experiments for *Ae. aegypti* (*α*_*aegypti*_ = 1.10, standard deviation 0.97 − 1.23), suggesting that mosquitoes become infectious before the DENV is detectable in the head tissues through standard immunofluorescent assays. The corresponding difference between mean EIP for *Ae. aegypti*, as determined by transmission experiments, and mean EIP for *Ae. albopictus*, as determined by assay experiments, was slightly higher (*α*_*albopictus*_ = 1.17, sd 1.03 − 1.30). Taking the difference between the two kinds of experiment into account, mean EIP for *Ae. albopictus* was found to only be 1.03 times longer than for *Ae. aegypti*. This equates to a very similar relative efficiency (*φ*) of 0.968 (0.95-0.98), which was used to rescale the temperature-EIP relationship, as determined by transmission experiments, to predict EIP for *Ae. albopictus*. We found the variance for the rate of EIP accumulation to be slightly higher for *Ae. aegypti*. This would mean infectious mosquitoes would be seen earlier, but complete infection of the cohort would be observed later, with the mean time to infection very similar to that of *Ae. albopictus* (Figure 1). The study effect in all analyses was found to be significant, indicating the high variability in measurement of mean EIP when using different experimental designs and different mosquito and viral strains.

**Figure 2:**
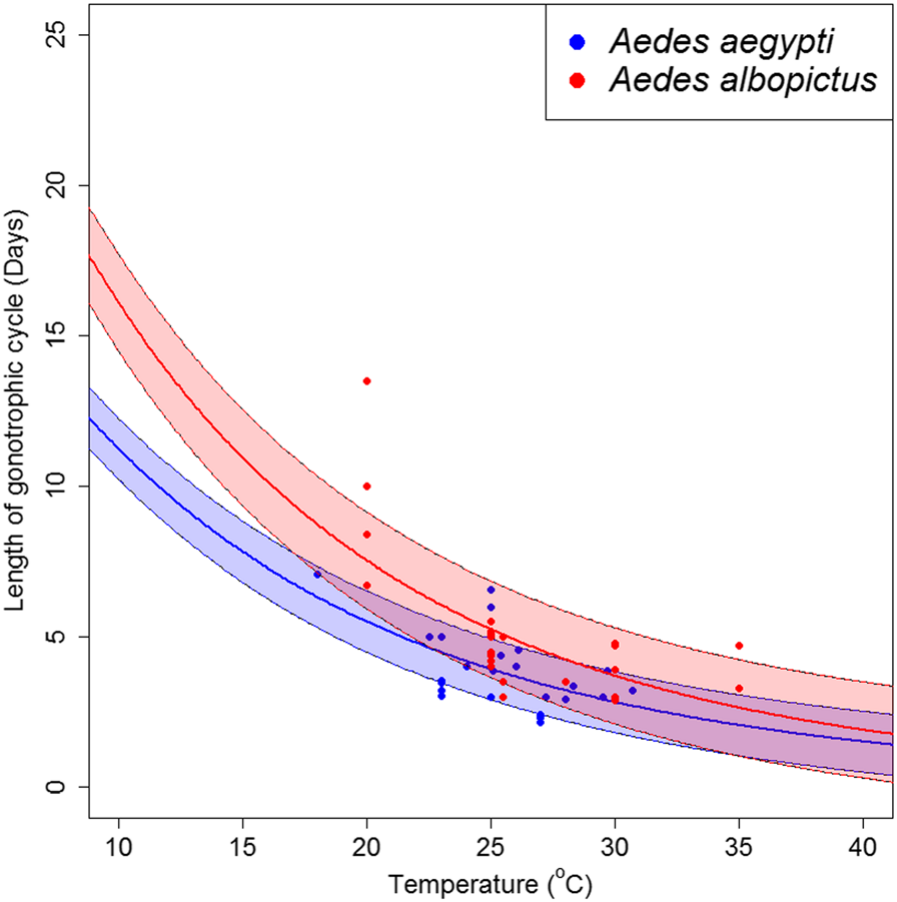
**Relationship between temperature and length of the first gonotrophic cycle for *Ae. aegypti *and *Ae. albopictus*.** The bold line shows the predicted mean first gonotrophic cycle length and the shaded areas show the prediction standard deviation for *Ae. aegypti* (blue) and *Ae. albopictus* (red).

The relationship between temperature and FGC length was defined by optimising an existing enzyme kinetics equation to new data. A total of 13 references contained 54 experiments that timed the mean length of the FGC (divided equally between *Ae. aegypti* and *Ae. albopictus*). The optimised enzyme kinetics equation for each species is shown in Figure 2. The model predicts broadly similar FGC length for the two species with *Ae. albopictus* having a slightly longer FGC length as well as higher uncertainty in its estimation.

### Temperature suitability for oviposition

**Figure 3:**
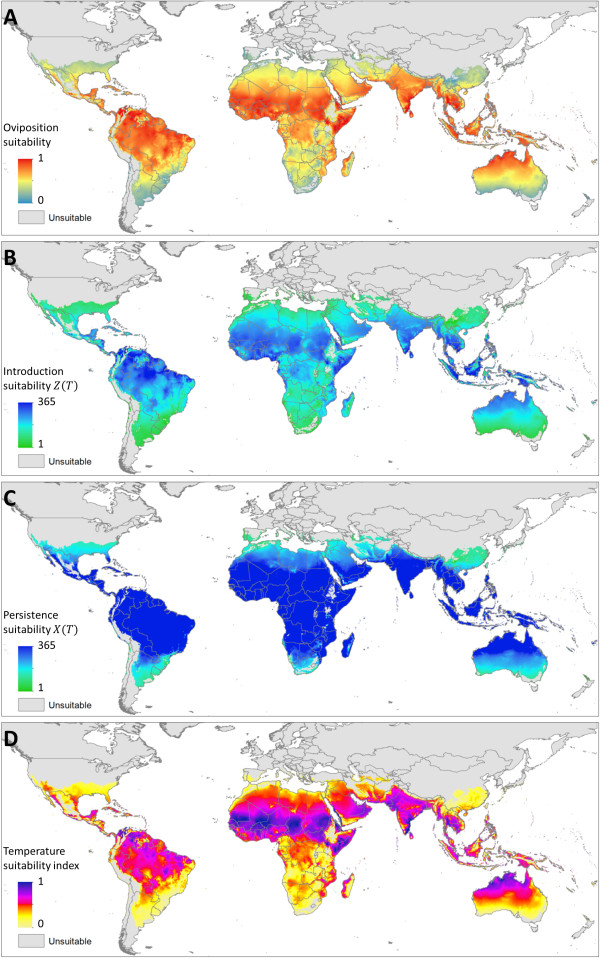
***Ae. aegypti *temperature suitability for persistence and DENV transmission. (A)** The annualised summary of temperature suitability for oviposition (*X*_*ovi*_(*T*)) on a normalised scale. **(B)** Introduction suitability; the number of days in a year where introduction of a DENV infected human would lead to ongoing transmission (*Z*(*T*)_*i*_ > 0). **(C)** Persistence suitability; the number of days in the year where onward DENV transmission could occur if a constant source of infectious humans were available (*X*(*T*)_*i*_ > 0). **(D)** The annualised summary of temperature suitability (*X*(*T*)) on a normalised scale. Predictions in all above maps are constrained to areas that permit oviposition (*X*_*ovi*_(*T*)_*i*_ > 0) on 219 or more days in the year, as determined by comparison with known occurrences of *Ae.aegypti*.

**Figure 4:**
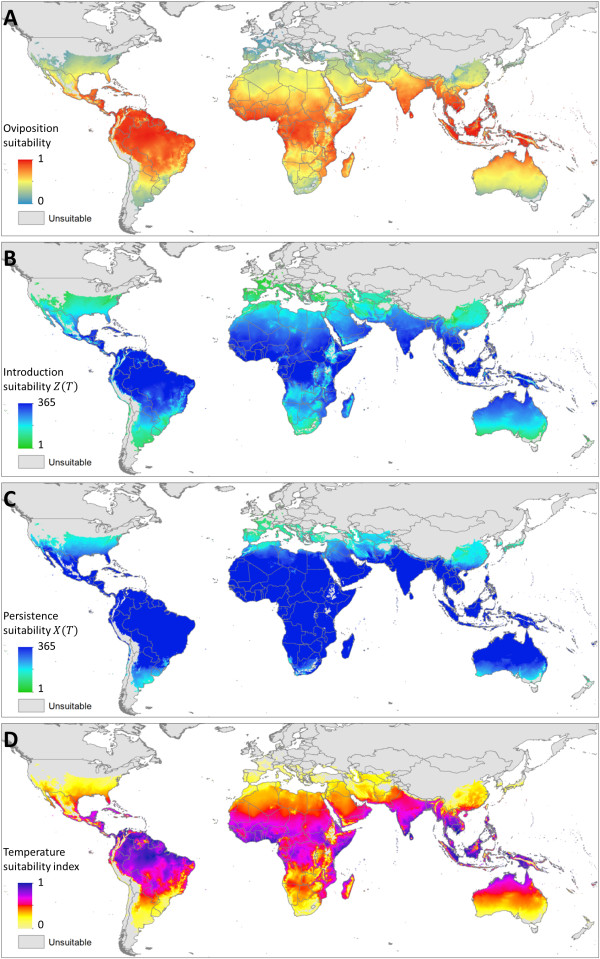
***Ae. albopictus *temperature suitability for persistence and DENV transmission.** Panels correspond directly to those described for *Ae. aegypti* in Figure 3, but constraints are expanded to areas that permit oviposition for 365 days in the year.

The thermal limits of persistence for each species were defined by comparing the predicted number of days in a year where *X*_*ovi*_(*T*)_*i*_ > 0 at the occurrence points where each species has been reported. For *Ae. aegypti,* 95% of occurrences had 219 or more days in an average year suitable for oviposition. The equivalent value for persistence of *Ae. albopictus* was 365 days, suggesting that temperature does not limit the persistence of *Ae. Albopictus* through inability of the adults to complete oviposition. The limits defined by these thresholds were used to constrain the geographic extent of all the predictions in Figures 3 and 4.

Within these limits, temperature suitability for oviposition (relative values of *X*_*ovi*_(*T*)) varies considerably, both geographically and between species. For *Ae. aegypti* oviposition, temperature suitability peaks in the warmest tropical environments in the northern Amazon, sub-Saharan Africa and South East Asia but drops by up to 100 fold as conditions become more temperate. The distribution of oviposition suitability for *Ae. albopictus* is similar, but the hottest regions are predicted to be less suitable due to the vector’s reduced survival at higher temperatures relative to *Ae. aegypti*. The geographic extent of *Ae. albopictus* is also increased relative to that of *Ae. aegypti*, although often with low suitability, such as in the USA. The temperature suitability index also predicts high suitability in many arid areas where neither species has been observed, such as the Sahara desert and the interior of Australia. In these areas, factors other than temperature are clearly more likely to be the limiting factors defining the absolute extent of oviposition suitability.

### Seasonal profile of temperature suitability

To summarise global intra-annual variation in temperature suitability two measures are presented in Figures 3B-C and 4B-C. The first summarises the number of days in an average year where *Z*(*T*)_*i*_ > 0. This summarises the number of days in a year where if an infectious human was introduced temperature would permit subsequent transmission, hereafter called introduction suitability, shown in Figures 3B and 4B. The latter of these indices summarises the number of days in which *X*(*T*)_*i*_ > 0, or if an infectious human population is omnipresent, the number of days in the year where temperature would permit an infectious mosquito population would be present, hereafter called persistence suitability, shown in Figures 3C and 4C. The former measure is more representative of a transmission free environment, while the latter is more representative of an endemic environment.

For both species, there are considerable differences between the introduction suitability and persistence suitability maps, indicating that temperature plays an important role in limiting not only the absolute geographic limits, but also in enabling different levels of endemicity. Surprisingly few environments were receptive to DENV introduction on every day of the year, which is consistent with the seasonal nature of reported dengue illness in many regions, such as southern Brazil, Mexico and China (Figures 3B-C and 4B-C). The map of introduction suitability does, however, highlight the prolonged periods of suitability in areas where dengue is presently underreported, such as sub-Saharan Africa [2,22,56], or has yet to be reported, such as the eastern Arabian peninsula and Australia’s Northern Territory. In such areas, if suitable mosquito breeding habitat and overlapping susceptible human populations were present, our results indicate that the potential for DENV introduction would be high (Figures 3D and 4D) and distributed throughout the year (Figures 3B and 4B). By contrast, many temperate environments have a limited window of transmission, particularly in many areas of North America and Europe. The predicted distribution of persistence suitability is far less heterogeneous with large portions of the world where onward transmission would be enabled on every day of the year given sufficient reservoirs of infectious humans. At the fringes of the distribution, although the window of transmission is expected to be longer than that of introduction suitability, the transmission season is still predicted to be significantly restricted. This indicates that even if mosquitoes in these areas were frequently exposed to infectious humans through either autochthonous or imported cases, we expect temperature would prevent year-round transmission and thus prevent the area from becoming endemic. The limits temperature places on endemicity can be identified by areas that allow transmission for 365 days minus the incubation period in humans and the duration of human infectiousness (typically 3-10 days [11] and 2-5 days [57] respectively).

The maps of both introduction and persistence suitability for *Ae. aegypti* and *Ae. albopictus* show a similar distribution. This is consistent with DENV EIP being a main limiting factor to the seasonal extent of DENV transmission. Although, DENV EIP is slightly longer in *Ae. albopictus*, adult females are predicted to live longer leading to similar seasonal profiles. The only place in which the two predictions differ is at the fringes of transmission where the wider predicted distribution of *Ae. albopictus* could allow a very limited transmission season at higher latitudes.

### Total suitability throughout the year

To summarise total suitability across the year *Z*(*T*) values for each time step were summed then normalised relative to the maximum global pixel value. This index provides discrimination of the effects of suitability between environments that are suitable throughout the year and also highlights the contribution of environments that have shorter, but much more pronounced periods of suitability. Temperature suitability for *Ae. aegypti* peaks in the warmest regions of the world, particularly in the sub-Saharan region and southern India. By contrast, *Ae. albopictus* temperature suitability peaks in northern South America where the diurnal temperature fluctuation is less pronounced and thus more favourable to *Ae. albopictus* survival at extreme temperatures. For both species, temperature suitability in the most suitable regions (typically tropical regions) is 100-1000 times the value in the least suitable regions (typically temperate regions). Alternatively, given equivalent conditions temperate regions would need 100-1000 times the number of mosquitoes to sustain equivalent levels of transmission. Predicted weekly changes in the temperature and oviposition suitability indices throughout an average year are also shown in animations in Additional files 3, 4, 5, 6, 7 and 8.

### Prediction uncertainty

Additional files 9 and 10 show the interquartile range in predictions for the temperature suitability index and oviposition suitability for both species. Uncertainty predictions of the temperature suitability index for *Ae. aegypti* (Additional file 9A) scales approximately linearly with the initial prediction (Figure 3D), while in the corresponding measure for *Ae. albopictus* (Additional file 10A) uncertainty values level off at high and low temperature suitability index extremes (Figure 4D). Uncertainty in predicting oviposition suitability for *Ae. aegypti* (Additional file 9B) is high in all but low suitability regions (Figure 3A), while uncertainty is highest in the mid oviposition suitability (Additional file 10B, Figure 4A) regions in the equivalent measure for *Ae. albopictus*. Average uncertainty in predicting the temperature suitability index for both species is similar, however uncertainty for oviposition suitability is lower for *Ae. albopictus* likely due to the longer lifespan reducing the overall effect of uncertainty in FGC length.

### Relative role of *Ae. aegypti* and *Ae. albopictus*

**Figure 5:**
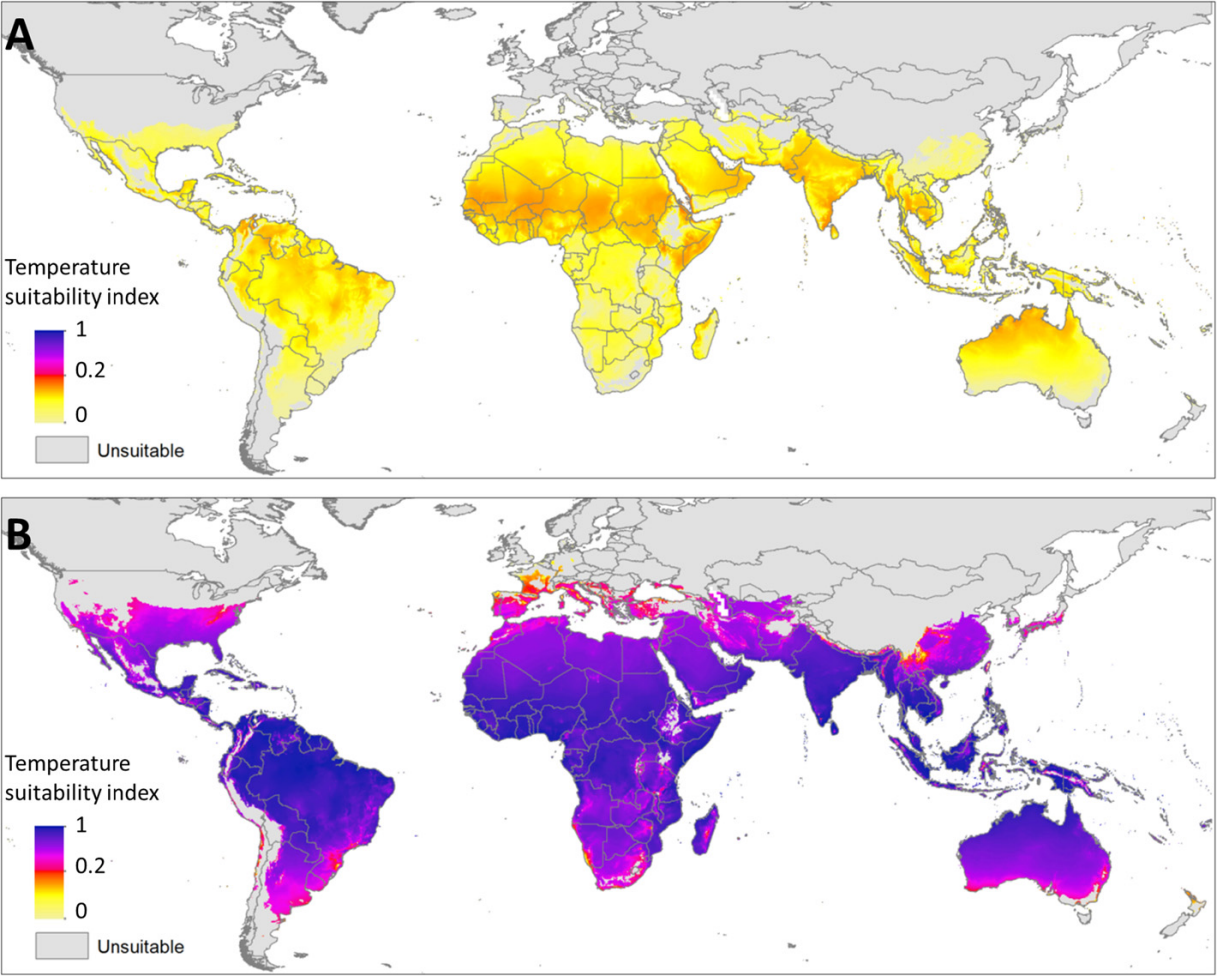
**Comparative temperature suitability of *Ae. aegypti *(A) and *Ae. albopictus *(B).** The annualised temperature suitability index (*X*(*T*)) normalised relative to the maximum value of both species and plotted on a logarithmic scale.

Figure 5 shows the temperature suitability index displayed in Figures 3D and 4D, but with pixel values normalised to the maximum value of the temperature suitability index for *Ae. albopictus* and re-plotted on a log-scale. On average the temperature suitability index for *Ae. albopictus* is around 42 times higher than for *Ae. aegypti*, meaning that suitability is predicted to be far higher for *Ae. albopictus*. Even the most suitable regions for *Ae. aegypti* DENV transmission have equivalent temperature suitability values as relatively marginal areas for *Ae. albopictus* DENV transmission, for example temperature suitability for *Ae. aegypti* in the tropical regions of Brazil is equivalent to temperature suitability for *Ae. albopictus* in northern France (Figure 5). This difference is due to the much longer survival times of adult female *Ae. albopictus* which far offsets the minor differences in vector competence. The relative values of the temperature suitability for oviposition index also show the same degree of disparity between the two species with *Ae. albopictus* able to produce on average 33 times the population of parous females.

## Discussion

In this study we used a range of biologically relevant temperature-based relationships in a dynamic model framework to evaluate the effects of temperature on DENV transmission on a global scale. For the first time we examined the global limits temperature places on DENV transmission taking into account seasonal and diurnal variations in temperature and their cumulative effects on mosquito cohorts and present explicit high spatial and temporal resolution predictions. We also show that temperature has the potential to limit different levels of DENV endemicity and map the predicted temperature constraints in both transmission free environments and highly endemic environments and suggest that the true limits for other endemicity settings are likely to lie between the two. Finally, we conclude that when considering the currently available data from existing competence experiments, our analysis of temperature and its effects on DENV development and mosquito survival indicates that DENV transmission in *Ae. albopictus* is only marginally less efficient than in *Ae. aegypti* and when considering the increased longevity of the former, we predict that *Ae. albopictus* has the potential to be a more efficient DENV vector, even if this potential is not fully realised in its current niche.

The prediction that *Ae. albopictus* has a higher potential for DENV transmission than *Ae. aegypti* is at odds with the current understanding of the global epidemiology of DENV where sustained high incidence and explosive outbreaks are rarely observed in locations where *Ae. albopictus* is the sole vector. This was previously thought to be at least partly due to reduced competence of *Ae. albopictus* (reduced rates of virus dissemination from the midgut to the salivary glands) [15,58]. Our results indicate, however, that by incorporating the full breadth of data from previous experiments over a wide range of mosquito and virus strains and experimental designs, competence of *Ae. albopictus* was similar to that of *Ae. aegypti*. This small difference becomes even less meaningful when incorporated into a DENV transmission model due to the longer lifespan, and thus longer time to become and remain infectious, of *Ae. albopictus*. By assuming a normally distributed probability of EIP completion we assumed that given enough time, every infected mosquito would progress to becoming infectious and the inter-species difference is associated with the efficiency with which DENV can cross the mid-gut barrier and disseminate to the salivary glands. Although the existence of species-specific immune responses [59], protective gut microbiota [59,60], protective extreme temperatures [32] or *Wolbachia*[61] may block transmission, this would be challenging to demonstrate experimentally and full recovery from infection before onward transmission seems an unlikely outcome. Irrespective of whether this phenomenon occurs, the modelling approach effectively accounts for recovered mosquitoes in the data by predicting an EIP that exceeds their lifespan, therefore modelling the progress of DENV infection in the mosquito through EIP has the ability to incorporate a variety of species-specific aspects of competence.

Assuming EIP and adult survival are modelled correctly, species-specific differences in vectorial capacity can be accounted for by differences in i) the ratio of mosquitoes to humans, which seems unlikely to be driven by inter-species differences in mosquito population size given similar survey rates [62-64], but may be affected by shifts in habitat preferences that increase the degree of mosquito-human population overlap, ii) features of vector competence that are not accounted for in current study designs [65-67] or iii) differences in the human blood feeding rate. Differences in blood feeding behaviour and the degree of mosquito-human population overlap are perhaps the most plausible explanations, given that *Ae. albopictus* typically either inhabits more rural environments (or in urban environments often in much less close human association than the highly peridomestic *Ae. aegypti*[26,64]) and that *Ae. aegypti* has a well documented tendency to feed frequently on human hosts [68,69]. This has the effect of reducing the mosquito-human ratio, but also affects the source of potential blood meals, with *Ae. albopictus* known to feed opportunistically on a variety of non-human hosts [24]. It has been shown, however, that *Ae. albopictus* is capable of both inhabiting high density urban environments and will sometimes feed frequently and almost exclusively on human blood [23,70,71]. Although specifically designed comparative studies between *Ae. aegypti* and *Ae. albopictus* that control for the underlying mechanisms are needed to further clarify differences in their DENV vector competence [65-67], our results would suggest that the capacity of *Ae. albopictus* to transmit DENV is primarily driven by some aspect of its ecology that affects how closely *Ae. albopictus* habitats align with human population densities and how frequently they feed on human blood.

Further experiments need to be conducted on temporal and environmental variation in the ratio of mosquitoes to humans and human blood feeding habits of *Ae. albopictus* to explore these hypotheses further. Given the second order effect of human blood feeding rate on vectorial capacity, and given a conservative crude estimate that the mosquito-human proportion is halved for *Ae. albopictus,* we would expect an *Ae. albopictus* human blood feeding rate of less than $\frac{2}{\sqrt{42}}=0.31$ times that of *Ae. aegypti*. This has been observed in some settings [23,72]. We do not mean to assert that *Ae. albopictus* is an unrecognised and underemphasised vector of DENV, but rather suggest that its involvement in transmission may be locally dependent and that further entomological investigation into human blood feeding rate and the overlap between mosquito and human populations needs to be undertaken to better understand its relative role in DENV transmission.

The results presented here apply generalised relationships to long-term average temperature data to predict the constraint temperature places on DENV transmission. While our predictions are likely to be representative of the majority of global environments most of the time, local adaptations and abnormal weather patterns may explain temporary deviations from predicted temperature constraints. It has been suggested that *Ae. albopictus* has adapted its immature stages to local environments to allow it to persist long inhospitable winters in Europe and the USA [73,74], however, when considering the temperature constraints solely on the adult forms, we found no difference between the temperature suitability for oviposition values attributed to *Ae. albopictus* occurrence points in its native Asian range and those in its newer invasive range. It has also been hypothesised that vector competence in *Ae. aegypti* varies geographically [75,76] and may even be influenced by co-evolution of local vector and virus populations [65,66,77]. Although we found the study effect to be highly significant in both EIP models, we were unable to distinguish the effects of experimental design from the effects of mosquito or viral strains. Previous work did not detect significant differences in EIP between DENV serotypes [11] and wide-scale differences in competence between different strains of *Ae. aegypti* are difficult to demonstrate considering the degree of variability in vector competence studies (Figure 1). Although there is some evidence of fine scale variation in competence in both mosquito [78-80] and virus [81,82] populations, more research is needed to determine if such structure exists at a wider scale considering human assisted rapid movement of vector and viruses may minimise opportunities for evolutionary interactions [83,84]. Until wider spatial scale structuring of mosquito-virus interaction can be demonstrated, a generalised prediction using vector and virus strains from a variety of different conditions is most likely to be representative of the majority of transmission environments. It is possible that behavioural adaptations to the micro-climates of the places where they rest or avoid extremes may allow adult mosquitoes to minimise the effects of harsh temperatures. While this may be possible in some local settings [52], protection is likely to be limited and environmental stochasticity is likely to minimise opportunities for permanent population establishment and subsequent DENV transmission. As such, we find no strong evidence that any local adaptations of the adult forms are directly temperature dependent and as a result, the predictions made here are likely to be indicative of the long-term environmental average.

The predictions of the limits temperature places on persistence and transmission broadly align with the known distribution of each species and the global distribution of DENV transmission (see Additional file 2, [1,2]). Discontinuities between the two most commonly occur in extremely arid environments, such as Australia and the Arabian Peninsula, where limited immature development habitat (through either low precipitation or minimal open human water storage) prevents the establishment of permanent vector populations and where humidity may be an important additional limiting factor [85]. We also predict brief periods of low risk in some temperate environments that have yet to experience DENV transmission. This approach predicts the absolute limits temperature places on DENV transmission, however, in some low temperature suitability environments, the mosquito population size required to reach a certain threshold vectorial capacity may be biologically implausibly high, thus further limiting the constraints temperature places on global DENV transmission. Alternatively, DENV transmission may have yet to expand to these new areas to realise its full niche, or additional factors other than temperature, particularly humidity, may be providing additional limits to transmission. To fully delineate the temperature-permitted niche, the effects of temperature on aspects of immature mosquito population dynamics and other aspects of transmission would need to be predicted.

In this manuscript we predict the absolute limits temperature places on global expansion of DENV, but we do not evaluate what is driving its expansion. Further analyses need to be conducted to identify how temperature interacts with other environmental and genetic factors to limit mosquito vector and virus distributions. This kind of analysis could be done by studying areas that have undergone recent DENV expansion and test whether this coincides with peak periods of risk as determined by temperature suitability along with relevant intra-annual changes in other candidate covariates. This would bring about a greater quantitative understanding of global DENV expansion and allow preventative measures to be put in place in areas and times of peak or elevated risk.

By comparison with the global distribution of dengue we have revealed that while temperature provides key constraints on global DENV transmission it is not the only factor limiting DENV transmission on a global scale. Despite this, many attempts to model the current and future global distribution of dengue and its vectors have predominantly or exclusively relied on temperature as a predictor [86-91]. Through a detailed biological consideration of the way in which temperature acts, it is possible to better inform statistical approaches in two key ways: i) constraining predictions to eliminate areas where temperature does not permit transmission and ii) including this information as a more biologically relevant covariate than standard temperature measures, but always in addition to other covariates to explain the complex epidemiology of DENV transmission. Combining mechanistic and statistical approaches will allow us to utilise the wealth of theoretical and experimental knowledge of the epidemiology of DENV transmission in generalised analyses [1,40,92].

While in this manuscript the temperature suitability index has been summarised in a broadly spatial analysis, the same index could be used in models that analyse time series data of vectors or dengue cases using a similar method. Such biological consideration is essential if we are to extrapolate past or current conditions to make predictions about the future, such as in projections of the future distribution of dengue or in dengue early warning systems. Temperature suitability is just one example of this and consideration of further biological limitations, such as geographic spread of dengue’s vectors, the role of other limiting factors such as humidity and the rate of dengue expansion need to be considered if projections of the future distribution of dengue are to be more reliable and useful.

The predictions from this analysis suggest that current day temperature regimes permit both vector establishment and DENV transmission beyond their current ranges. While factors other than temperature are likely to further limit the distribution of dengue and its vectors, if expansion is to occur, temperature suitability can elucidate the regions and suitable seasons most at risk. For *Ae. aegypti* and/or *Ae. albopictus* free areas, the introduction suitability (*Z*_*ovi*_(*T*)) maps and annual profiles can be used to direct surveillance to the regions and times of the year where a single mosquito introduction would carry the highest risk of population persistence. For areas with year-round populations of *Ae. aegypti,* but without persistent DENV transmission, the DENV introduction (*Z*(*T*)) maps and annual profiles can be used to guide vector control to limit periods and areas of peak risk. For areas with persistent dengue transmission (endemic) the annual profiles of persistence suitability (*X*(*T*)) can be used to direct control towards the time of year that is most likely to break the on-going transmission cycle. Finally, in areas with established *Ae. albopictus* populations, their potential for DENV transmission can be evaluated through a combination of *Z*(*T*) and *X*(*T*) indices that elucidate areas and times of peak risk which can then guide further entomological investigation regarding human blood feeding rate and mosquito-human population overlap, thereby further improving estimates of DENV vectorial capacity of this species. This combination of high spatial and temporal resolutions of risk estimates coupled with guided local entomological investigation is likely to give a more complete understanding of future DENV expansion risk than a consideration of the risk imposed by extrapolated niche approaches alone [86,87,89-91].

## Conclusions

Here we used a range of entomological and virological experimental data to parameterise temperature-based relationships in a dynamic transmission model to assess the limits that changing temperature regimes place on *Ae. aegypti* and *Ae. albopictus* persistence as well as the global distribution of DENV transmission. The model predictions were used to map the absolute and seasonal temperature limits to vector persistence and DENV transmission on a global scale. These maps reveal that as well as constraining the absolute extent of transmission, temperature may also limit levels of DENV endemicity. A comparison of temperature suitability for DENV transmission by *Ae. aegypti* vs. *Ae. albopictus* revealed that given similar population sizes and human blood feeding rates, *Ae. albopictus* has a higher vectorial capacity. This finding contradicts existing explanations and suggests that ecological factors, such as blood feeding patterns and the degree of mosquito-human population overlap, may explain why areas with established *Ae. albopictus* populations do not experience sustained dengue outbreaks. The resulting models can inform statistical approaches to model DENV transmission and persistence of its vectors by replacing raw temperature variables to include known biological understanding of transmission. The risk of dengue expansion into novel areas and at novel times of the year can be estimated by using the seasonal profiles presented here to target entomological investigation of human biting rate and the degree of mosquito-human population overlap. As well as being informative for surveillance purposes, this further investigation will help identify and understand additional factors limiting DENV transmission and will inform and support new ways to limit the expansion of the geographic extent and range of endemicity of dengue.

## Abbreviations

EIP: Extrinsic incubation period; DENV: Dengue virus; FGC: First gonotrophic cycle.

## Competing interests

The authors declare that they have no competing interests.

## Authors’ contributions

OJB, DLS, PWG and SIH designed the experiment. OJB, wrote the manuscript and collected and analysed the data. MUK collected additional data. NG, DLS and MUK helped with data analysis. All authors helped with data and results interpretation and were involved in drafting, revising and approving the final version of the manuscript.

## Supplementary Material

Additional file 1 Priors used for Bayesian Inter-species EIP comparison model. Click here for file

Additional file 2 **Assembly of the supplementary *Ae. aegypti *** **and *Ae. albopictus *** **database.** Click here for file

Additional file 3 **Animation of *Ae. aegypti *** **temperature suitability for oviposition (*X***_***ovi***_**(*T*)) throughout an average year.** Click here for file

Additonal file 4 **Animation of *Ae. albopictus *** **temperature suitability for oviposition (*X***_***ovi***_**(*T*)) throughout an average year.** Click here for file

Additional file 5 **Animation of *Ae. aegypti *** **temperature suitability for introduction of dengue virus transmission (*Z*** **(*T*)) throughout an average year.** Click here for file

Additional file 6 **Animation of *Ae. albopictus *** **temperature suitability for introduction of dengue virus transmission (*Z*** **(*T*)) throughout an average year.** Click here for file

Additional file 7 **Animation of *Ae. aegypti *** **temperature suitability for persistence of dengue virus transmission (*X*** **(*T*)) throughout an average year.** Click here for file

Additional file 8 **Animation of *Ae. albopictus *** **temperature suitability for persistence of dengue virus transmission (*X*** **(*T*)) throughout an average year.** Click here for file

Additional file 9 ***Ae. aegypti *model prediction uncertainty for the temperature suitability index (A) and oviposition suitability (B).** The output shows the interquartile range in predictions presented in maps 3A and 3D. Click here for file

Additional file 10 ***Ae. albopictus *model prediction uncertainty for the temperature suitability index (A) and oviposition suitability (B).** The output shows the interquartile range in predictions presented in maps 4A and 4D. Click here for file
