# Supplementary material for: Global temperature constraints on Aedes aegypti and Ae. albopictus persistence and competence for dengue virus transmission
Source: Parasit Vectors. 2014 Jul 22;7:338. doi: 10.1186/1756-3305-7-338 (PMC4148136; doi:10.1186/1756-3305-7-338)
Supplement: Additional file 1 — Priors used for Bayesian Inter-species EIP comparison model. [file 1756-3305-7-338-S1.docx]

**Additional file 1: Priors used for Bayesian Inter-species EIP comparison model**

$$\alpha_{aegypti} \sim N(0, 0.001)$$

$$\alpha_{albopictus} \sim N(0, 0.001)$$

$${\sigma_{EIP}}_{T, i, j} \sim lnN\left( \mu_{assay}, {\sigma_{assay}}^{2} \right)$$

$${\sigma_{EIP}}_{T, i, aegypti} \sim lnN\left( 2.14, 0.270 \right)$$

$${\sigma_{EIP}}_{T, i, albopictus} \sim lnN\left( 2.14, 0.270 \right)$$

$${\sigma_{study}}^{2}\sim Gam(9, 0.9)$$
