# Supplementary material for: Global temperature constraints on Aedes aegypti and Ae. albopictus persistence and competence for dengue virus transmission
Source: Parasit Vectors. 2014 Jul 22;7:338. doi: 10.1186/1756-3305-7-338 (PMC4148136; doi:10.1186/1756-3305-7-338)
Supplement: Additional file 2 — Assembly of the supplementary Ae. aegypti and Ae. albopictus database. [file 1756-3305-7-338-S2.docx]

**Additional File 2: Assembly of the supplementary *Aedes aegypti* and *Aedes albopictus* database**

**AF2.1 Overview**

The *Aedes* (Stegomyia) *aegypti* and *Ae.* (Stegomyia) *albopictus* database comprises occurrence data (presence, abundance and absence) linked to point or polygon locations, derived from peer-reviewed literature. Data sources are described in full here. To collate the peer-reviewed database, literature searches were undertaken using the major search engines and the resulting articles were manually reviewed. Geo-spatial information for 5966 occurrences of *Ae. aegypti* (4,397) and *Ae. albopictus* (1,569) were extracted using site descriptions from 1,556 articles. Locations were identified within the articles by searching for specific coordinates or, if not available, small administrative units. All data entries for both point and polygon data were manually checked by the authors and then underwent a series of routine quality control procedures to ensure correct geo-positioning.

**AF2.2 Peer-reviewed Literature Search**

PubMed (<http://www.ncbi.nlm.nih.gov/entrez/query.fcgi>) 1960 to 2009 was searched using term “Aedes”, “aegypti” and “albopictus”. The MESH term technology used in the PubMed citation archive ensured all pseudonyms were automatically included (<http://www.nlm.nih.gov/mesh/2008/MBrowser.html>) in the searches. The same process was repeated for ISI Web of Science ([http://wok.mimas.ac.uk](http://wok.mimas.ac.uk/)) and ProMED (records since 1994; [http://www.promedmail.org](http://www.promedmail.org/)). The searches were last updated on 3^th^ November 2009. No language restrictions were placed on these searches, however, only those citations with a full title and abstract were downloaded into Endnote version 10 (Thomson ResearchSoft, USA, <http://www.endnote.com>). 10,314 original citations were scanned and a total of 3,704 references were retrieved with 1,556 unique articles identified as potentially containing useable location data.

In-house language skills allowed processing of all English, French, Portuguese and Spanish articles. Russian articles with data were translated and included in the database. We were unable to extract information from a small number of Turkish, Polish, Hebrew, Italian, German and Chinese articles.

**AF2.3 Disease vector data and species type**

Confirmed occurrences of *Ae.* *aegypti* and *Ae.* *albopictus* found within the peer reviewed literature were recorded as an occurrence datapoint. Species names reported as collected in the site were entered in the database. If both species have been collected at the same site during the same time period, a second data point/polygon has been entered with all respective information. Occurrence points for *Ae.* *aegypti* were classified in presence, abundance and absence (4,080, 130, 187 respectively) and *Ae.* a*lbopictus* in presence and absence (1,469, 100 respectively). No additional information on ‘sub-species’ or other such as information given in the source pdf e.g. ‘temporate’ or ‘tropical’ have been recorded. Beginning and end year of survey was included in the data abstraction. In this analysis only presence and abundance points from any time period were used as they represent a known unique geographic occurrence of the disease.

Both point and polygon was entered into a bespoke PostgreSQL database that links vector data to spatial data. A link to the PDF library was also included in the database.

**AF2.4 Geo-positioning occurrence points**

All available location information in the peer-reviewed literature was extracted from each article. The site name and contextual information were used together to locate the study site using Microsoft Encarta (Microsoft Corporation, Redmond, WA) and Google Earth 5.1. Place names are often duplicated within a country so the contextual information was used to ensure the right site was selected. Where the site name was not found, the contextual information was used to scan sites in the approximate area to check for names that had been transliterated in Microsoft Encarta/Google Earth in a different way to the published article (e.g. Imichli and Imishly). If the site was not located using Microsoft Encarta or Google Earth, a range of online georeferencing resources were tried including Getty Thesaurus (<http://www.getty.edu/research/conducting_research/vocabularies/tgn/index.html>), Alexandria (<http://middleware.alexandria.ucsb.edu/client/gaz/adl/index.jsp>), Falling Rain (<http://www.fallingrain.com/world/>), National geospatial-intelligence agency (<http://geonames.nga.mil/ggmaviewer>), and Maplandia (<http://maplandia.com/>). If the study site could be georeferenced to a specific place, it was recorded as a point location. In total, 5,430 point locations from the peer-reviewed literature were included in the database at this stage. If the study site could only be identified at an administrative area level (e.g. province or district, etc), it was recorded as a polygon and the centroid (mean centre) was derived to give a latitude and longitude for the site. A total of 536 polygon locations from the peer-reviewed literature were recorded in the database. All point locations and polygon centroids were converted to decimal degrees before entering the quality control process described below. To define the absolute limits of *Aedes* persistence only the point data was used to improve geographic precision.

**AF2.5 Automatic Validation and Quality control**

All datapoints were crosschecked and validated using a standardised validation procedure. First a multi-polygon shapefile defining land/water boundaries was created as a template to validate geopositioned points. This land/water shape file was rasterised to spatially overlapping raster pixels (the polygon boundaries must cover the centre of the raster pixel) at a 5 x 5 km resolution and was used to ensure all disease occurrence points were positioned on a valid land pixel.

Any points that met the following criteria were excluded from the database:

1. Points found further than 10km from a land boundary. Points located less than 10km from a land boundary were repositioned to the nearest land raster cell.
2. Points representing the centroid of an administrative division having an area greater than 111km^2^ (1 degree at the equator).

Once the quality control procedure was complete, the final database contained 5,966 occurrence datapoints (including 5,430 point locations and 536 small polygon centroids) covering a period from 1960 to 2009. Maps displaying the *Ae.* *aegypti* and *Ae.* *albopictus* locations are provided in Figures AF2A and AF2B.


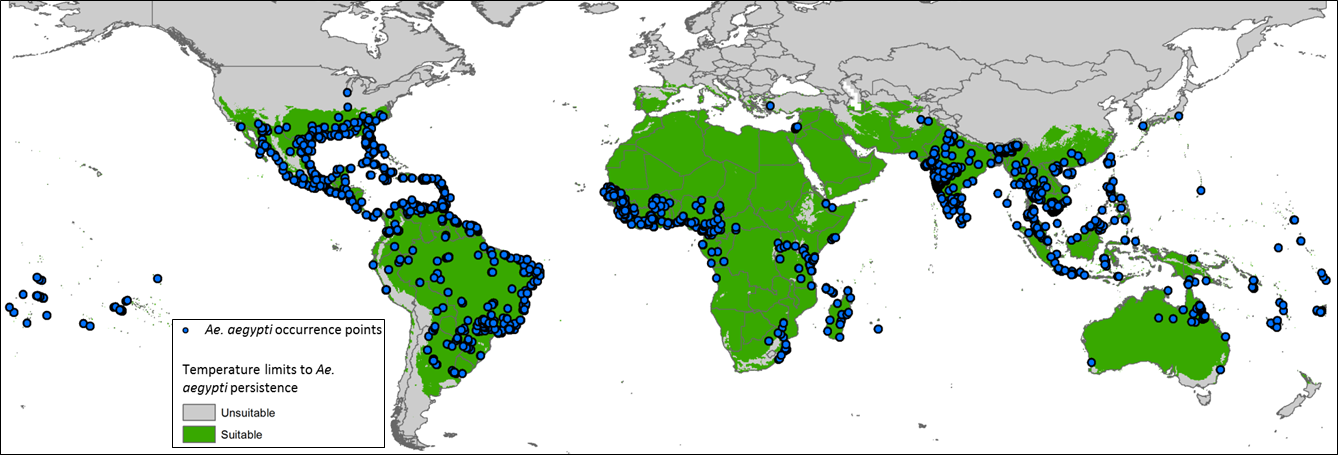


**Figure AF2A. The distribution of *Ae. aegypti* occurrence points in the database. The background shows the temperature limits to *Ae. aegypti* persistence where 283 or more days in an average year are suitable for parous females.**

**
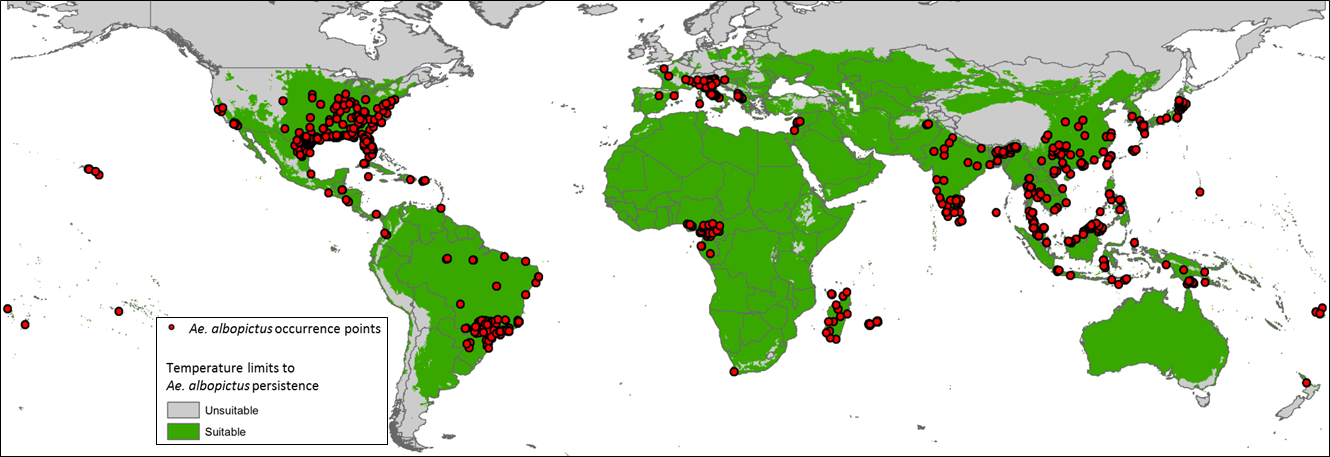
**

**Figure AF2B. The distribution of *Ae. albopictus* occurrence points in the database. The background shows the temperature limits to *Ae. albopictus* persistence where 165 or more days in an average year are suitable for parous females.**

**S2.6 Bibliography of final endnote library**

1. Abe, M., et al., *The Buen Pastor cemetery in Trujillo, Venezuela: measuring dengue vector output from a public area.* Tropical Medicine & International Health, 2005. **10**(6): p. 597-603.

2. AbuHassan, A., C.R. Adanan, and W.A. Rahman, *Patterns in* Aedes albopictus *(Skuse) population density, host-seeking, and oviposition behavior in Penang, Malaysia.* Journal of Vector Ecology, 1996. **21**(1): p. 17-21.

3. Addy, P.A.K., R.K. Esena, and S.K.N. Atuahene, *Possible contributing factors to the paucity of yellow fever epidemics in the Ashanti region of Ghana, West Africa.* East African Medical Journal, 1996. **73**(1): p. 3-9.

4. Adeleke, M.A., et al., *Mosquito larval habitats and public health implications in Abeokuta, Ogun State, Nigeria.* Tanzania Health Research Bulletin, 2008. **10**(2): p. 103-107.

5. Adhami, J. and P. Reiter, *Introduction and establishment of* Aedes (Stegomyia) albopictus *Skuse (Diptera : Culicidae) in Albania.* Journal of the American Mosquito Control Association, 1998. **14**(3): p. 340-343.

6. Aditya, G., et al., *Bamboo stumps as mosquito larval habitats in Darjeeling Himalayas, India: A spatial scale analysis.* Insect Science, 2008. **15**(3): p. 245-249.

7. Aguilera, L., et al., *[Incidence of* Aedes (S) aegypti *and other Culicidae in the municipality of Playa, La Habana City].* Revista Cubana de Medicina Tropical, 2000. **52**(3): p. 174-179.

8. Aguilera, L., et al., *[The ecological succession of mosquito species in the town of Boyeros, Ciudad de la Habana 1994-1996].* Revista Cubana de Medicina Tropical, 2000. **52**(2): p. 138-144.

9. Ahid, S.M. and R. Lourenco-De-Oliveira, *[Mosquitoes potential vectors of canine heartworm in the Northeast Region from Brazil].* Revista de Saúde Pública, 1999. **33**(6): p. 560-565.

10. Ahmad, I., S. Astari, and M. Tan, *Resistance of* Aedes aegypti *(Diptera: Culicidae) in 2006 to pyrethroid insecticides in Indonesia and its association with oxidase and esterase levels.* Pakistan Journal of Biological Science, 2007. **10**(20): p. 3688-3692.

11. Ahmad, R., et al., *Detection of dengue virus from field* Aedes aegypti *and* Aedes albopictus *adults and larvae.* Southeast Asian Journal of Tropical Medicine and Public Health, 1997. **28**(1): p. 138-142.

12. Akoua-Koffi, C., et al., *[Investigation surrounding a fatal case of yellow fever in Cote d'Ivoire in 1999].* Bulletin de la Societe de Pathologie Exotique, 2001. **94**(3): p. 227-230.

13. Akoua-Koffi, C., et al., *[Detection and management of the yellow fever epidemic in the Ivory Coast, 2001].* Medecine Tropicale, 2002. **62**(3): p. 305-309.

14. Akram, W. and J.J. Lee, *Effect of habitat characteristics on the distribution and behavior of* Aedes albopictus*.* Journal of Vector Ecology, 2004. **29**(2): p. 379-382.

15. Akstein, E., *Chromosomes of* Aedes Aegypti*, and of Some Other Species of Mosquitoes.* Bulletin of the Research Council of Israel, 1962. **B 11**(3): p. 146-155.

16. Ali, A., J.K. Nayar, and R.D. Xue, *Comparative toxicity of selected larvicides and insect growth regulators to a Florida laboratory population of* Aedes albopictus*.* Journal of the American Mosquito Control Association, 1995. **11**(1): p. 72-76.

17. Ali, S.R. and L.E. Rozeboom, *Cross-Insemination Frequencies between Strains of* Aedes-Albopictus *and Members of* Aedes-Scutellaris *Group.* Journal of Medical Entomology, 1971. **8**(3): p. 263-265.

18. Ali, S.R. and L.E. Rozeboom, *Cross-Mating between* Aedes-(S)-Polynesiensis *Marks and* Aedes-(S)-Albopictus *Skuse in a Large Cage.* Mosquito News, 1971. **31**(1): p. 80-84.

19. Almiron, W.R. and R. Asis, *[Abundance indices of larvae and pupae of* Aedes aegypti *(Diptera: Culicidae) in Cordoba City].* Revista de la Facultad de Ciencias Médicas, 2003. **60**(1): p. 37-41.

20. Alto, B.W. and S.A. Juliano, *Precipitation and temperature effects on populations of* Aedes albopictus *(Diptera : Culicidae): Implications for range expansion.* Journal of Medical Entomology, 2001. **38**(5): p. 646-656.

21. Alto, B.W. and S.A. Juliano, *Temperature effects on the dynamics of* Aedes albopictus *(Diptera : Culicidae) populations in the laboratory.* Journal of Medical Entomology, 2001. **38**(4): p. 548-556.

22. Alto, B.W., et al., *Larval competition differentially affects arbovirus infection in* Aedes *mosquitoes.* Ecology, 2005. **86**(12): p. 3279-3288.

23. Alves, M.C.G.P. and N.N. da Silva, *[Simplifying the sampling method for evaluating the larval density of* Aedes aegypti *in Brazil].* Revista de Saude Publica, 2001. **35**(5): p. 467-473.

24. Alves, M.C.G.P., S.D. Gurgel, and M.D.R.R. Dealmeida, *[Sampling Desing for Larval Density Computation of* Aedes-Aegypti *and* Aedes-Albopictus *in the State of Sao-Paulo, Brazil].* Revista de Saude Publica, 1991. **25**(4): p. 251-256.

25. Amerasinghe, F.P. and T.S.B. Alagoda, *Mosquito Oviposition in Bamboo Traps, with Special Reference to* Aedes-Albopictus*,* Aedes-Novalbopictus *and* Armigeres-Subalbatus*.* Insect Science and Its Application, 1984. **5**(6): p. 493-500.

26. Amusan, A.A., A.B. Idowu, and F.S. Arowolo, *Comparative toxicity effect of bush tea leaves (*Hyptis suaveolens*) and orange peel (*Citrus sinensis*) oil extract on larvae of the yellow fever mosquito* Aedes aegypti*.* Tanzania Health Research Bulletin, 2005. **7**(3): p. 174-178.

27. Amusan, A.A., et al., *Sampling mosquitoes with CDC light trap in rice field and plantation communities in Ogun State, Nigeria.* Tanzania Health Research Bulletin, 2005. **7**(3): p. 111-116.

28. Anderson, C.R., K.R.P. Singh, and J.K. Sarkar, *Isolation of Chikungunya Virus from* Aedes Aegypti *Fed on Naturally Infected Humans in Calcutta.* Current Science, 1965. **34**(20): p. 579-580.

29. Andis, M.D., et al., *Strategies for the emergency control of arboviral epidemics in New Orleans.* Journal of the American Mosquito Control Association, 1987. **3**(2): p. 125-130.

30. Andrade, C.S., et al., *Reappearance of* Aedes aegypti *(Diptera: Culicidae) in Lima, Peru.* Memorias Do Instituto Oswaldo Cruz, 2001. **96**(5): p. 657-658.

31. Andreadis, T.G., *A survey of mosquitoes breeding in used tire stockpiles in Connecticut.* Journal of the American Mosquito Control Association, 1988. **4**(3): p. 256-260.

32. Angel, B. and V. Joshi, *Distribution and seasonality of vertically transmitted dengue viruses in* Aedes *mosquitoes in arid and semi-arid areas of Rajasthan, India.* Journal of Vector Borne Diseases, 2008. **45**(1): p. 56-59.

33. Angel, B. and V. Joshi, *Distribution of dengue virus types in* Aedes aegypti *in dengue endemic districts of Rajasthan, India.* Indian Journal of Medical Research, 2009. **129**(6): p. 665-668.

34. Angel, B., K. Sharma, and V. Joshi, *Association of ovarian proteins with transovarial transmission of dengue viruses by* Aedes *mosquitoes in Rajasthan, India.* Indian Journal of Medical Research, 2008. **128**(3): p. 320-323.

35. Annis, B., *Comparison of the effectiveness of two deet formulations against* Aedes albopictus *in the Philippines.* Journal of the American Mosquito Control Association, 1991. **7**(4): p. 543-546.

36. Annis, B., et al., *Suppression of larval* Aedes aegypti *populations in household water storage containers in Jakarta, Indonesia, through releases of first-instar* Toxorhynchites splendens *larvae.* Journal of the American Mosquito Control Association, 1989. **5**(2): p. 235-238.

37. Anonymous, *Update:* Aedes albopictus *infestation--United States, Mexico.* MMWR Morbidity and Mortality Weekly Report, 1989. **38**(25): p. 440, 445-446.

38. Anonymous, Aedes albopictus *introduction into continental Africa, 1991.* MMWR Morbidity and Mortality Weekly Report, 1991. **40**(48): p. 836-838.

39. Anonymous, *Dengue epidemic--Peru, 1990.* Canada Diseases Weekly Report, 1991. **17**(40): p. 217-218.

40. Anonymous, *From the Centers for Disease Control and Prevention. Dengue type 3 infection--Nicaragua and Panama, October-November 1994.* JAMA - the Journal of the American Medical Association, 1995. **273**(11): p. 840-841.

41. Anonymous, *Dengue type 3 infection. Nicaragua and Panama, October-November 1994.* Weekly Epidemiological Record, 1995. **70**(6): p. 41-43.

42. Anonymous, *Dengue fever at the U.S.-Mexico border, 1995-1996.* MMWR. Morbidity and Mortality Weekly Report, 1996. **45**(39): p. 841-844.

43. Anonymous, *Dengue fever at the U.S.-Mexico border, 1995-1996.* MMWR Morbidity and Mortality Weekly Report, 1996. **45**(39): p. 841-844.

44. Anonymous *DENGUE - U.S./MEXICO BORDER, 1995-1996*. PROMED, 1996.

45. Anonymous, *From the Centers for Disease Control and Prevention. Dengue fever at the US-Mexico border, 1995-1996.* JAMA - the Journal of the American Medical Association, 1996. **276**(18): p. 1464-1465.

46. Anonymous. *DENGUE ADVISORY - CUBA (HAVANA)*. 1999 [cited 1999 17 May]; El Nuevo Dia, Puerto Rico, 5? May 1999 [in Spanish].

47. Anonymous, *Update: outbreak of Rift Valley Fever--Saudi Arabia, August-November 2000.* MMWR. Morbidity and Mortality Weekly Report, 2000. **49**(43): p. 982-985.

48. Anonymous. *DENGUE - VENEZUELA: ALERT*. 2000 [cited 2000 19 January]; EFE News Services (U.S.) Inc. Tue 18 Jan 2000.

49. Anonymous *YELLOW FEVER - BRAZIL (SAO PAULO): CONFIRMED* PROMED, 2000.

50. Anonymous. *DENGUE/DHF - PANAMA*. 2001 [cited 2001 4 August]; La Nacion (Costa Rica), ACAN-EFE, Fri 3 Aug 2001 (trans. by Mod.MPP) [edited].

51. Anonymous. *DENGUE/DHF UPDATES (01): 8 JAN 2001 [Costa Rica]*. 2001 [cited 2001 8 January]; Xinhua 30 Dec 2000 [edited].

52. Anonymous. *DENGUE/DHF UPDATES (06): 24 MAY 2001 [Peru]*. 2001 [cited 2001 25 May]; El Comerico (Quito) 8 May 2001 (translated by MPP) [edited].

53. Anonymous. *DENGUE/DHF - PANAMA (02)*. 2001 [cited 2001 10 November]; Terra Online-Domingo, 4 de novembro de 2001 [edited].

54. Anonymous. *DENGUE - BRAZIL*. 2001 [cited 2001 18 October]; Agencia EFE (via COMTEX), Wed 17 Oct 2001 [edited].

55. Anonymous AEDES ALBOPICTUS*, IMPORTED - USA (CALIFORNIA*. PROMED, 2001.

56. Anonymous. *DENGUE/DHF UPDATES (24): 25 JUN 2002 [Philippines/ Vietnam/ Brazil]*. 2002 [cited 2002 25 June]; The Sun Star, Mon 17 Jun 2002 [edited].

57. Anonymous *Brazil: Dengue Epidemic Under Control, but Threat Remains* PROMED, 2002.

58. Anonymous AEDES ALBOPICTUS *- PANAMA* PROMED, 2002.

59. Anonymous AEDES ALBOPICTUS *- PANAMA*. PROMED, 2002.

60. Anonymous. *DENGUE/DHF UPDATE 2003 (29) [Indonesia/ China]*. 2003 [cited 2003 21 July]; Jakarta Post, Fri 18 Jul 2003 [edited].

61. Anonymous. *DENGUE/DHF UPDATE 2003 (10) [Indonesia/ Paraguay/ Australia/ Ecuador]*. 2003 [cited 2003 10 March]; The Jakarta Post, Tue 5 Mar 2003 [edited].

62. Anonymous. *DENGUE/DHF UPDATE 2003 (43) [India]*. 2003 [cited 2003 6 November]; Times of India 6 Nov 2003 [edited].

63. Anonymous. *DENGUE/DHF UPDATE 2003 (37) [India/ Indonesia/ Philippines]*. 2003 [cited 2003 22 september]; Times of India 22 Sep 2003 [edited].

64. Anonymous *UNDIAGNOSED FEBRILE ILLNESS - INDONESIA (BOGOR): RFI*. PROMED, 2003.

65. Anonymous *DENGUE/DHF UPDATE 2003 (37)*. PROMED, 2003.

66. Anonymous *DENGUE/DHF UPDATE 2003 (39)*. PROMED, 2003.

67. Anonymous *DENGUE/DHF UPDATE 2003 (48)* PROMED, 2003.

68. Anonymous. *DENGUE/DHF UPDATE 2004 (04) [Peru/ Honduras/ Bolivia]*. 2004 [cited 2004 31 January]; Xinhua News Agency 29 Jan 2004 [edited].

69. Anonymous. *DENGUE/DHF UPDATE 2004 (18) [Sri Lanka/ Vietnam]*. 2004 [cited 2004 20 June]; ColomboPage News Desk, Sri Lanka Sat 19 Jun 2004 [edited].

70. Anonymous *DENGUE FEVER - AUSTRALIA (NORTHERN TERRITORY): MOSQUITO CONTROL* PROMED, 2004.

71. Anonymous. *DENGUE/DHF UPDATE 2005 (08)*. 2005 [cited 2005 23rd Apr].

72. Anonymous *DENGUE/DHF UPDATE 2005 (08)* PROMED, 2005.

73. Anonymous *DENGUE/DHF UPDATE 2005 (22)* PROMED, 2005.

74. Anonymous *DENGUE/DHF UPDATE 2006 (28)* PROMED, 2006.

75. Anonymous *DENGUE/DHF UPDATE 2006 (30)*. PROMED, 2006.

76. Anonymous *DENGUE/DHF UPDATE 2006 (33)* PROMED, 2006.

77. Anonymous *Dengue/DHF update 2006 (29)* PROMED, 2006.

78. Anonymous *Dengue/DHF update 2006 (33)*. PROMED, 2006.

79. Anonymous *DENGUE/DHF UPDATE 2007 (22)*. PROMED, 2007.

80. Anonymous *DENGUE/DHF UPDATE 2007 (26)* PROMED, 2007.

81. Anonymous *DENGUE/DHF UPDATE 2007 (34)* PROMED, 2007.

82. Anonymous *DENGUE/DHF UPDATE 2007 (43)*. PROMED, 2007.

83. Anonymous *DENGUE/DHF UPDATE 2007 (44)*. PROMED, 2007.

84. Anonymous *CHIKUNGUNYA - ITALY (EMILIA ROMAGNA) (04)*. PROMED, 2007.

85. Anonymous *CHIKUNGUNYA - ITALY (EMILIA ROMAGNA) (07)*. PROMED, 2007.

86. Anonymous *DENGUE/DHF UPDATE 2007 (50)*. PROMED, 2007.

87. Anonymous *DENGUE/DHF UPDATE 2007 (48)*. PROMED, 2007.

88. Anonymous *DENGUE/DHF UPDATE 2007 (43)*. PROMED, 2007.

89. Anonymous *DENGUE/DHF UPDATE 2007 (42)*. PROMED, 2007.

90. Anonymous *DENGUE/DHF UPDATE 2007 (39)*. PROMED, 2007.

91. Anonymous *DENGUE/DHF UPDATE 2007 (31)*. PROMED, 2007.

92. Anonymous *DENGUE/DHF UPDATE 2007 (29)*. PROMED, 2007.

93. Anonymous *DENGUE/DHF UPDATE 2007 (26)*. PROMED, 2007.

94. Anonymous *DENGUE/DHF UPDATE 2007 (22)*. PROMED, 2007.

95. Anonymous *YELLOW FEVER - BRAZIL (06)*. PROMED, 2008.

96. Anonymous *DENGUE/DHF UPDATE 2008 (03)* PROMED, 2008.

97. Anonymous *CHIKUNGUNYA (11): INDIA (KERALA)* PROMED, 2008.

98. Anonymous *DENGUE/DHF UPDATE 2008 (22)* PROMED, 2008.

99. Anonymous *DENGUE/DHF UPDATE 2008 (24)* PROMED, 2008.

100. Anonymous *YELLOW FEVER - SOUTH AMERICA (29): BRAZIL (SAO PAULO), MONKEYS, SUSPECTED*. PROMED, 2008.

101. Anonymous *DENGUE/DHF UPDATE 2008 (45)*. PROMED, 2008.

102. Anonymous *DENGUE/DHF UPDATE 2008 (56): SINGAPORE, AUSTRALIA*. PROMED, 2008.

103. Anonymous *DENGUE/DHF UPDATE 2008 (47)*. PROMED, 2008.

104. Anonymous *DENGUE/DHF UPDATE 2008 (45)*. PROMED, 2008.

105. Anonymous *DENGUE/DHF UPDATE 2008 (44)*. PROMED, 2008.

106. Anonymous *DENGUE/DHF UPDATE 2008 (40)*. PROMED, 2008.

107. Anonymous *DENGUE/DHF UPDATE 2008 (39)*. PROMED, 2008.

108. Anonymous *DENGUE/DHF UPDATE 2008 (31)*. PROMED, 2008.

109. Anonymous *DENGUE/DHF UPDATE 2008 (29)*. PROMED, 2008.

110. Anonymous *DENGUE/DHF UPDATE 2008 (28)*. PROMED, 2008.

111. Anonymous *DENGUE/DHF UPDATE 2008 (26)*. PROMED, 2008.

112. Anonymous *DENGUE/DHF UPDATE 2008 (23)*. PROMED, 2008.

113. Anonymous *DENGUE/DHF UPDATE 2008 (22)*. PROMED, 2008.

114. Anonymous *DENGUE/DHF UPDATE 2008 (20)*. PROMED, 2008.

115. Anonymous *DENGUE/DHF UPDATE 2008 (18)*. PROMED, 2008.

116. Anonymous *DENGUE/DHF UPDATE 2008 (17)*. PROMED, 2008.

117. Anonymous *DENGUE/DHF UPDATE 2008 (16)*. PROMED, 2008.

118. Anonymous *DENGUE/DHF UPDATE 2008 (15)*. PROMED, 2008.

119. Anonymous *DENGUE/DHF UPDATE 2008 (12)*. PROMED, 2008.

120. Anonymous *DENGUE/DHF UPDATE 2008 (09)*. PROMED, 2008.

121. Anonymous *DENGUE/DHF UPDATE 2008 (08)*. PROMED, 2008.

122. Anonymous *DENGUE/DHF UPDATE 2009 (02)* PROMED, 2009.

123. Anonymous *DENGUE/DHF UPDATE 2009 (03)*. PROMED, 2009.

124. Anonymous *DENGUE/DHF UPDATE 2009 (04)* PROMED, 2009.

125. Anonymous *DENGUE/DHF UPDATE 2009 (05)*. PROMED, 2009.

126. Anonymous *DENGUE/DHF UPDATE 2009 (23)* PROMED, 2009.

127. Anonymous *DENGUE/DHF UPDATE 2009*. PROMED, 2009.

128. Anonymous *DENGUE/DHF UPDATE 2009 (43)*. PROMED, 2009.

129. Anonymous *DENGUE/DHF UPDATE 2009 (41)*. PROMED, 2009.

130. Anonymous *DENGUE/DHF UPDATE 2009 (40)*. PROMED, 2009.

131. Anonymous *DENGUE/DHF UPDATE 2009 (39)*. PROMED, 2009.

132. Anonymous AEDES ALBOPICTUS *- FRANCE: FIRST REPORT*. PROMED, 2009.

133. Anonymous *DENGUE/DHF UPDATE 2009 (35)*. PROMED, 2009.

134. Anonymous *DENGUE/DHF UPDATE 2009 (33)*. PROMED, 2009.

135. Anonymous *DENGUE/DHF UPDATE 2009 (28)*. PROMED, 2009.

136. Anonymous *DENGUE/DHF UPDATE 2009 (23)*. PROMED, 2009.

137. Anonymous *DENGUE/DHF UPDATE 2009 (21)*. PROMED, 2009.

138. Anonymous *DENGUE/DHF UPDATE 2009 (14)*. PROMED, 2009.

139. Anonymous *DENGUE/DHF UPDATE 2009 (12)*. PROMED, 2009.

140. Anonymous *DENGUE/DHF UPDATE 2009 (11)*. PROMED, 2009.

141. Anonymous *DENGUE/DHF UPDATE 2009 (09)*. PROMED, 2009.

142. Anonymous *DENGUE/DHF UPDATE 2009 (07)*. PROMED, 2009.

143. Anonymous *DENGUE/DHF UPDATE 2009 (06)*. PROMED, 2009.

144. Anonymous *DENGUE/DHF UPDATE 2009 (05)*. PROMED, 2009.

145. Anonymous *DENGUE/DHF UPDATE 2009 (04)*. PROMED, 2009.

146. Anonymous *DENGUE/DHF UPDATE 2009 (03)*. PROMED, 2009.

147. Anosike, J.C., et al., *Epidemiology of tree-hole breeding mosquitoes in the tropical rainforest of Imo State, South-East Nigeria.* Annals of Agricultural and Environmental Medicine, 2007. **14**(1): p. 31-38.

148. Ansari, M.A. and R.K. Razdan, *Concurrent control of mosquitoes and domestic pests by use of deltamethrin-treated curtains in the New Delhi Municipal Committee, India.* Journal of the American Mosquito Control Association, 2001. **17**(2): p. 131-136.

149. Ansari, M.A., R.K. Razdan, and U. Sreehari, *Laboratory and field evaluation of Hilmilin against mosquitoes.* Journal of the American Mosquito Control Association, 2005. **21**(4): p. 432-436.

150. Ansari, M.A., et al., *Bioefficacy of Olyset (R) nets against mosquitoes in India.* Journal of the American Mosquito Control Association, 2006. **22**(1): p. 102-106.

151. Anyanwu, I.N., et al., *The incrimination of* Aedes (stegomyia) aegypti *as the vector of Dirofilaria repens in Nigeria.* Veterinary Parasitology, 2000. **92**(4): p. 319-327.

152. Apostol, B.L., et al., *Population genetics with RAPD-PCR markers: The breeding structure of* Aedes aegypti *in Puerto Rico.* Heredity, 1996. **76**: p. 325-334.

153. Apostol, B.L., et al., *Use of randomly amplified polymorphic DNA amplified by polymerase chain reaction markers to estimate the number of* Aedes aegypti *families at oviposition sites in San Juan, Puerto Rico.* American Journal of Tropical Medicine and Hygiene, 1994. **51**(1): p. 89-97.

154. Appawu, M., et al., *Surveillance of viral haemorrhagic fevers in ghana: entomological assessment of the risk of transmission in the northern regions.* Ghana Medical Journal, 2006. **40**(4): p. 137-141.

155. Apperson, C.S., B. Engber, and J.F. Levine, *Relative Suitability of* Aedes-Albopictus *and* Aedes-Aegypti *in North-Carolina to Support Development of* Dirofilaria-Immitis*.* Journal of the American Mosquito Control Association, 1989. **5**(3): p. 377-382.

156. Aranda, C., R. Eritja, and D. Roiz, *First record and establishment of the mosquito* Aedes albopictus *in Spain.* Medical and Veterinary Entomology, 2006. **20**(1): p. 150-152.

157. Armstrong, P.M. and R. Rico-Hesse, *Differential susceptibility of* Aedes aegypti *to infection by the American and Southeast Asian genotypes of dengue type 2 virus.* Vector Borne and Zoonotic Diseases, 2001. **1**(2): p. 159-168.

158. Armstrong, P.M. and R. Rico-Hesse, *Efficiency of dengue serotype 2 virus strains to infect and disseminate in* Aedes aegypti*.* American Journal of Tropical Medicine and Hygiene, 2003. **68**(5): p. 539-544.

159. Arostegui, J., et al., *Impact of evidence-based community derived interventions for the control of the dengue virus vector* Aedes aegypti *in Managua, Nicaragua.* American Journal of Tropical Medicine and Hygiene, 2006. **75**(5): p. 108.

160. Arredondo-Jimenez, J.I. and K.M. Valdez-Delgado, Aedes aegypti *pupal/demographic surveys in southern Mexico: consistency and practicality.* Annals of Tropical Medicine and Parasitology, 2006. **100 Suppl 1**: p. S17-S32.

161. Arrivillaga, J. and R. Barrera, *Food as a limiting factor for* Aedes aegypti *in water-storage containers.* Journal of Vector Ecology

, 2004. **29**(1): p. 11-20.

162. Arunachalam, N., et al., *Studies on dengue in rural areas of Kurnool District, Andhra Pradesh, India.* Journal of the American Mosquito Control Association, 2004. **20**(1): p. 87-90.

163. Arunachalam, N., et al., *Studies on dengue in rural areas of Kurnool District, Andhra Pradesh, India.* Journal of the American Mosquito Control Association, 2004. **20**(1): p. 87-90.

164. Arunachalam, N., et al., *Natural vertical transmission of dengue viruses by* Aedes aegypti *in Chennai, Tamil Nadu, India.* Indian Journal of Medical Research, 2008. **127**(4): p. 395-397.

165. Atmosoed.S, et al., *Man-Biting Activity of* Aedes-Aegypti *in Djakarta, Indonesia.* Mosquito News, 1972. **32**(3): p. 467-469.

166. Avila Montes, G.A., et al., *[Evaluation of an educational module on dengue and* Aedes Aegypti *for schoolchildren in Honduras].* Revista Panamericana de Salud Publica, 2004. **16**(2): p. 84-94.

167. Avila Montes, G.A., et al., *[Evaluation of an educational module on dengue and* Aedes Aegypti *for schoolchildren in Honduras].* Revista Panamericana de Salud Pública, 2004. **16**(2): p. 84-94.

168. Aviles, G., et al., Aedes Aegypti *in Cordoba Province, Argentina.* Journal of the American Mosquito Control Association, 1997. **13**(3): p. 255-258.

169. Aviles, G., et al., *[Outbreak of Dengue-2 virus in Salta, Argentina, 1988].* Medicina, 2000. **60**(6): p. 875-879.

170. Ayres, C., et al., *Genetic diversity in Brazilian Populations of* Aedes albopictus*.* Memorias Do Instituto Oswaldo Cruz, 2002. **97**(6): p. 871-875.

171. Ayres, C.F., et al., *Genetic differentiation of* Aedes Aegypti *(Diptera: Culicidae), the major dengue vector in Brazil.* Journal of Medical Entomology, 2003. **40**(4): p. 430-435.

172. Babu, C.J., K.N. Panicker, and P.K. Das, *Breeding of* Aedes-Aegypti *in Closed Septic Tanks.* Indian Journal of Medical Research, 1983. **77**(May): p. 637-637.

173. Badolo, A., et al., *Evaluation of the sensitivity of* Aedes Aegypti *and* Anopheles gambiae *complex mosquitoes to two insect repellents: DEET and KBR 3023.* Tropical Medicine & International Health, 2004. **9**(3): p. 330-334.

174. Badrinath, P. *YELLOW FEVER THREAT TO INDIA*. PROMED, 2001.

175. Bagny, L., et al., Aedes *(Diptera: Culicidae) vectors of arboviruses in Mayotte (Indian Ocean): distribution area and larval habitats.* Journal of Medical Entomology, 2009. **46**(2): p. 198-207.

176. Bailey, D.L., R.G. Jones, and P.R. Simmonds, *Effects of Indigenous Toxorhynchites-Rutilus-Rutilus on* Aedes-Aegypti *Breeding in Tire Dumps.* Mosquito News, 1983. **43**(1): p. 33-37.

177. Balakrishnan, N., S. Venkatesh, and S. Lal, *An entomological study on the dengue vectors during outbreak of dengue in Tiruppur town and its surroundings, Tamil Nadu, India.* Journal of Communicable Diseases, 2006. **38**(2): p. 164-168.

178. Balestra, R.A., et al., *[Occurrence of* Aedes (Stegomyia) albopictus *(Skuse) in urban area of Tocantins state, Brazil].* Neotropical Entomology, 2008. **37**(2): p. 233-235.

179. Ballard, J.W. and I.D. Marshall, *An investigation of the potential of* Aedes camptorhynchus *(Thom.) as a vector of Ross River virus.* Australian Journal of Experimental Biology and Medical Science, 1986. **64 ( Pt 2)**: p. 197-200.

180. Baly, A., et al., *Cost effectiveness of* Aedes Aegypti *control programmes: participatory versus vertical.* Transactions of the Royal Society of Tropical Medicine and Hygiene, 2007. **101**(6): p. 578-586.

181. Banerjee, K., D.T. Mourya, and A.S. Malunjkar, *Susceptibility and Transmissibility of Different Geographical Strains of* Aedes-Aegypti *Mosquitos to Chikungunya Virus.* Indian Journal of Medical Research, 1988. **87**: p. 134-138.

182. Bang, Y.H. and C.P. Pant, *Field Trial of Abate Larvicide for Control of* Aedes-Aegypti *in Bangkok, Thailand.* Bulletin of the World Health Organization, 1972. **46**(3): p. 416-425.

183. Bang, Y.H., C.P. Pant, and N. Gratz, *Suppression of a Field Population of* Aedes-Aegypti *by Malathion Thermal Fogs and Abate Larvicide.* Bulletin of the World Health Organization, 1972. **46**(4): p. 554-558.

184. Bang, Y.H. and R.J. Tonn, *Effectiveness of Different Insecticides and Formulations against* Aedes-Aegypti *Larvae in Ant Traps in Bangkok, Thailand.* Bulletin of the World Health Organization, 1969. **41**(2): p. 320-324.

185. Bang, Y.H., R.J. Tonn, and Jatanase.S, *Development and Reversion of DDT Resistance in an* Aedes-Aegypti *Population in Bangkok, Thailand.* Bulletin of the World Health Organization, 1971. **45**(3): p. 404-410.

186. Banks, A. *DENGUE/DHF UPDATES (34): 1 SEP 2002 [Bangladesh/Nicaragua/Cuba/ Mexico]*. 2002 [cited 2002 1 September]; The Daily Star, Tue 27 Aug 2002 [edited].

187. Banks, A. *DENGUE/DHF UPDATE 2006 (01)*. PROMED, 2006.

188. Banks, A.-L. *Dengue/DHF update 2006 (30)* PROMED, 2006.

189. Banks, A.-L. *Dengue/DHF update 2007* PROMED, 2007.

190. Banks, A.-L., Barrett, B. and M. Hopp *Dengue/DHF update 2007 (08)* PROMED, 2007.

191. Banks, A.-L. and K.A. Bryant *Dengue/DHF update 2007 (16)* PROMED, 2007.

192. Banks, A.-L. and J. Dudley *Dengue/DHF update 2007 (06)*. PROMED, 2007.

193. Banks, A.-L., Dudley, J. and D. Duffy *Dengue/DHF update 2006 (32)* PROMED, 2006.

194. Banks, A.-L., Marshall, M. and D. Silver *Dengue/DHF Update 2007 (04)* PROMED, 2007.

195. Banks, A.-L., Marshall, M. and D. Silver *Dengue/DHF Update 2007 (04)* PROMED, 2007.

196. Banks, A.-L. and A. Rodriguez *Dengue/DHF update 2006 (21)* PROMED, 2006.

197. Banks, A.-L. and T. Schmidt *Dengue/DHF update 2007 (02)* PROMED, 2007.

198. Banks, A.L. *DENGUE/DHF UPDATE 2006 (12)*. 2006 [cited 2006 28th March].

199. Bansal, S.K. and K.V. Singh, *Relative susceptibility of some common mosquito vector larvae to synthetic insecticidal compounds in north-western Rajasthan.* Journal of Environmental Biology, 2007. **28**(4): p. 829-832.

200. Barata, E., et al., *Capture of culicids in urban areas: evaluation of the resting box method.* Revista de Saude Publica, 2007. **41**(3): p. 375-382.

201. Barata, E.A.M.D., et al., *[*Aedes Aegypti *(I.) population in an endemic area of dengue in the Southeast Brazil].* Revista de Saude Publica, 2001. **35**(3): p. 237-242.

202. Barbazan, P., et al., *Assessment of a new strategy, based on* Aedes Aegypti *(L.) pupal productivity, for the surveillance and control of dengue transmission in Thailand.* Annals of Tropical Medicine and Parasitology, 2008. **102**(2): p. 161-171.

203. Barbosa, M.G., et al., *[Record of epidemiologically important Culicidae in the rural area of Manaus, Amazonas].* Revista da Sociedade Brasileira de Medicina Tropical, 2008. **41**(6): p. 658-663.

204. Barcellos, C., et al., *[Identification of places with potential transmission of dengue fever in Porto Alegre using Geographical Information Systems].* Revista da Sociedade Brasileira de Medicina Tropical, 2005. **38**(3): p. 246-250.

205. Barcellos, C., et al., *[Identification of places with potential transmission of dengue fever in Porto Alegre using Geographical Information Systems].* Revista da Sociedade Brasileira de Medicina Tropical, 2005. **38**(3): p. 246-250.

206. Barennes, H., et al., *[An epidemic risk of yellow fever in Burkina Faso despite a rapid immunisation riposte: role of a multidisciplinary investigation team].* Sante, 2002. **12**(3): p. 323-329.

207. Barker, C.M., et al., *Habitat preferences and phenology of Ochlerotatus triseriatus and* Aedes albopictus *(Diptera : Culicidae) in southwestern Virginia.* Journal of Medical Entomology, 2003. **40**(4): p. 403-410.

208. Barkerhudson, P., R. Jones, and B.H. Kay, *Categorization of Domestic Breeding Habitats of* Aedes-Aegypti *(Diptera, Culicidae) in Northern Queensland, Australia.* Journal of Medical Entomology, 1988. **25**(3): p. 178-182.

209. Barreau, C., F.X. Jousset, and M. Bergoin, *Venereal and vertical transmission of the* Aedes albopictus *parvovirus in* Aedes Aegypti *mosquitoes.* American Journal of Tropical Medicine and Hygiene, 1997. **57**(2): p. 126-131.

210. Barrera, R., *Competition and resistance to starvation in larvae of container-inhabiting* Aedes *mosquitoes.* Ecological Entomology, 1996. **21**(2): p. 117-127.

211. Barrera, R., M. Amador, and G.G. Clark, *Ecological factors influencing* Aedes Aegypti *(Diptera: Culicidae) productivity in artificial containers in Salinas, Puerto Rico.* Journal of Medical Entomology, 2006. **43**(3): p. 484-492.

212. Barrera, R., M. Amador, and G.G. Clark, *Sample-size requirements for developing strategies, based on the pupal/demographic survey, for the targeted control of dengue.* Annals of Tropical Medicine and Parasitology, 2006. **100 Suppl 1**: p. S33-S43.

213. Barrera, R., M. Amador, and G.G. Clark, *Use of the pupal survey technique for measuring* Aedes Aegypti *(Diptera: Culicidae) productivity in Puerto Rico.* American Journal of Tropical Medicine and Hygiene, 2006. **74**(2): p. 290-302.

214. Barrera, R., et al., *Unusual productivity of* Aedes Aegypti *in septic tanks and its implications for dengue control.* Medical and Veterinary Entomology, 2008. **22**(1): p. 62-69.

215. Barrera, R., J. Avila, and S. Gonzalez-Tellez, *Unreliable supply of potable water and elevated* Aedes Aegypti *larval indices: a causal relationship?* Journal of the American Mosquito Control Association, 1993. **9**(2): p. 189-195.

216. Barrera, R., et al., *[Stratification of a hyperendemic city in hemorrhagic dengue].* Revista Panamericana de Salud Publica, 2000. **8**(4): p. 225-233.

217. Barrera, R., C.E. Machadoallison, and L.A. Bulla, *Breeding Places Persistence, Succession and Population Regulation in 3 Urban Culicidae (Culex-Fatigans Wied., C-Corniger Theo. And* Aedes-Aegypti *L).* Acta Cientifica Venezolana, 1981. **32**(5): p. 386-393.

218. Barrera, R., et al., *Public service deficiencies and* Aedes Aegypti *breeding sites in Venezuela.* Bulletin of the Pan American Health Organization, 1995. **29**(3): p. 193-205.

219. Barrera, R.R., R. Machadoallison, and L.A. Bulla, *[Breeding Places, Larval Density and Niche Segregation in 3 Urban Culicidae (*Culex-Fatigans *Wied,* Culex-Corniger *Theo, and* Aedes-Aegypti *L) at Caracas Cemetery].* Acta Cientifica Venezolana, 1979. **30**(4): p. 418-424.

220. Barrett, W.L., *Damage Caused by* Lankesteria Culicis *(Ross) to* Aedes Aegypti *(L).* Mosquito News, 1968. **28**(3): p. 441-444.

221. Barrett, W.L., F.M. Miller, and J.W. Kliewer, *Distribution in Texas of* Lankesteria-Culicis *(Ross), a Parasite of* Aedes-Aegypti *(L).* Mosquito News, 1971. **31**(1): p. 23-27.

222. Bartley, L.M., C.A. Donnelly, and G.P. Garnett, *The seasonal pattern of dengue in endemic areas: mathematical models of mechanisms.* Transactions of the Royal Society of Tropical Medicine and Hygiene, 2002. **96**(4): p. 387-397.

223. Bassole, I.H., et al., *Ovicidal and larvicidal activity against* Aedes Aegypti *and* Anopheles gambiae *complex mosquitoes of essential oils extracted from three spontaneous plants of Burkina Faso.* Parassitologia, 2003. **45**(1): p. 23-26.

224. Batra, C.P., P.K. Mittal, and T. Adak, *Control of* Aedes Aegypti *breeding in desert coolers and tires by use of* Bacillus thuringiensis *var.* Israelensis *formulation.* Journal of the American Mosquito Control Association, 2000. **16**(4): p. 321-323.

225. Bayard, V., E. Quiroz, and J. Mojica, *[Re-emergence of dengue in Panama].* Revista Medica de Panama, 1996. **21**(3): p. 85-92.

226. Beard, C.B., et al., *Response of Domestic and Peridomestic Strains of* Aedes-Aegypti *(Diptera, Culicidae) in New-Orleans, Louisiana, USA, to Organo-Phosphate, Organochlorine, and Pyrethroid Insecticides.* Journal of Medical Entomology, 1985. **22**(3): p. 276-280.

227. Beckett, E.B. and H. Townson, *Variability in the Flight Muscles of Field and Laboratory Strains of the Mosquito,* Aedes-Aegypti *(L) (Diptera, Culicidae).* International Journal of Insect Morphology & Embryology, 1982. **11**(5-6): p. 319-325.

228. Beebe, N.W., et al., *Genetic diversity of the dengue vector* Aedes Aegypti *in Australia and implications for future surveillance and mainland incursion monitoring.* Communicable Diseases Intelligence, 2005. **29**(3): p. 299-304.

229. Beebe, N.W., et al., *A polymerase chain reaction-based diagnostic to identify larvae and eggs of container mosquito species from the Australian region.* Journal of Medical Entomology, 2007. **44**(2): p. 376-380.

230. Behbahani, A., et al., *Population differentiation and Wolbachia phylogeny in mosquitoes of the* Aedes scutellaris *group.* Medical and Veterinary Entomology, 2005. **19**(1): p. 66-71.

231. Bell, D.D. and J.L. Benach, Aedes-Aegypti *in Southeastern New-York-State.* Mosquito News, 1973. **33**(2): p. 248-250.

232. Bellini, R., et al., *Efficacy of different ovitraps and binomial sampling in* Aedes albopictus *surveillance activity.* Journal of the American Mosquito Control Association, 1996. **12**(4): p. 632-636.

233. Bellini, R., et al., Aedes albopictus *(Diptera:Culicidae) is incompetent as a vector of hepatitis C virus.* Apmis, 1997. **105**(4): p. 299-302.

234. Bennett, J.K., et al., *New state record for the Asian tiger mosquito,* Aedes albopictus *(skuse).* Journal of the American Mosquito Control Association, 2005. **21**(4): p. 341-343.

235. Bennett, K.E., et al., *Variation in vector competence for dengue 2 virus among 24 collections of* Aedes Aegypti *from Mexico and the United States.* American Journal of Tropical Medicine and Hygiene, 2002. **67**(1): p. 85-92.

236. Bernhardt, S.A., et al., *Variation in vector competence for dengue 2 virus among collections of* Aedes Aegypti *from the Yucatan and Vera Cruz regions of Mexico.* American Journal of Tropical Medicine and Hygiene, 2006. **75**(5): p. 212.

237. Beserra, E.B. and F.P. Castro, Jr., *[Compared biology of populations of* Aedes (Stegomyia) aegypti *(L.) (Diptera: Culicidae) of Paraiba state, Brazil].* Neotropical Entomology, 2008. **37**(1): p. 81-85.

238. Beserra, E.B., et al., *[Biology and thermal exigency of* Aedes Aegypti *(L.) (Diptera : Culicidae) from four bioclimatic localities of Paraiba].* Neotropical Entomology, 2006. **35**(6): p. 853-860.

239. Beserra, E.B., et al., *[Resistance of* Aedes Aegypti *(L.) (Diptera : Culicidae) populations to organophosphates ternephos in the Paraiba State, Brazil].* Neotropical Entomology, 2007. **36**(2): p. 303-307.

240. Bharaj, P., et al., *Concurrent infections by all four dengue virus serotypes during an outbreak of dengue in 2006 in Delhi, India.* Virology Journal, 2008. **5**: p. -.

241. Biber, P.A., et al., *Laboratory evaluation of susceptibility of natural subpopulations of* Aedes Aegypti *larvae to temephos.* Journal of the American Mosquito Control Association, 2006. **22**(3): p. 408-411.

242. Birungi, J. and L.E. Munstermann, *Genetic structure of* Aedes albopictus *(Diptera : Culicidae) populations based on mitochondrial ND5 sequences: Evidence for an independent invasion into Brazil and United States.* Annals of the Entomological Society of America, 2002. **95**(1): p. 125-132.

243. Bishop, S. *YELLOW FEVER - USA EX VENEZUELA (02)*. PROMED, 1999.

244. Bisset, J.A., et al., *[Status of resistance to insecticides and resistance mechanisms in larvae from Playa municipality collected during the intensive campaign against* Aedes Aegypti *in Havana City, 2001-2002].* Revista Cubana de Medicina Tropical, 2004. **56**(1): p. 61-66.

245. Bisset, J.A., M.M. Rodriguez, and L. Caceres, *[Levels of resistance to insecticides and their mechanisms in 2 strains of* Aedes Aegypti *from Panama].* Revista Cubana de Medicina Tropical, 2003. **55**(3): p. 191-195.

246. Bisset, J.A., et al., *[High esterases as mechanism of resistance to organophosphate insecticides in* Aedes Aegypti *strains].* Revista Cubana de Medicina Tropical, 2001. **53**(1): p. 37-43.

247. Biswas, D., et al., *Observations on the breeding habitats of* Aedes Aegypti *in Calcutta following an episode of dengue haemorrhagic fever.* Indian Journal of Medical Research, 1993. **97**: p. 44-46.

248. Black, W.C., et al., *Breeding Structure of a Colonizing Species -* Aedes-Albopictus *(Skuse) in the United-States.* Heredity, 1988. **60**: p. 173-181.

249. Black, W.C., et al., *Breeding Structure of a Colonizing Species -* Aedes-Albopictus *(Skuse) in Peninsular Malaysia and Borneo.* Heredity, 1988. **61**: p. 439-446.

250. Black, W.C., D.K. Mclain, and K.S. Rai, *Patterns of Variation in the Rdna Cistron within and among World Populations of a Mosquito,* Aedes-Albopictus *(Skuse).* Genetics, 1989. **121**(3): p. 539-550.

251. Boisier, P., et al., *[Dengue 1 epidemic in the Grand Comoro Island (Federal Islamic Republic of the Comores). March-May 1993].* Annales de la Societe Belge de Medecine Tropicale, 1994. **74**(3): p. 217-229.

252. Bond, H.A., G.B. Craig, and R.W. Fay, *Field Mating and Movement of Aedes-Aegypti.* Mosquito News, 1970. **30**(3): p. 394-402.

253. Bond, H.A. and R.W. Fay, *Factors Influencing* Aedes Aegypti *Occurrence in Containers.* Mosquito News, 1969. **29**(1): p. 113-116.

254. Bond, J.G., C.F. Marina, and T. Williams, *The naturally derived insecticide spinosad is highly toxic to* Aedes *and* Anopheles *mosquito larvae.* Medical and Veterinary Entomology, 2004. **18**(1): p. 50-56.

255. Bonilauri, P., et al., *Chikungunya virus in* Aedes albopictus*, Italy.* Emerging Infectious Diseases, 2008. **14**(5): p. 852-854.

256. Bosio, C.F., et al., *Genetic structure of* Aedes Aegypti *populations in Thailand using mitochondrial DNA.* American Journal of Tropical Medicine and Hygiene, 2005. **72**(4): p. 434-442.

257. Bosio, C.F., et al., *Variation in the efficiency of vertical transmission of dengue-1 virus by strains of* Aedes albopictus *(Diptera: Culicidae).* Journal of Medical Entomology, 1992. **29**(6): p. 985-989.

258. Bouldouyre, M.A., et al., *Factors of severity at admission during an epidemic of dengue 1 in New Caledonia (South Pacific) in 2003.* Scandinavian Journal of Infectious Diseases, 2006. **38**(8): p. 675-681.

259. Bracco, J.E., et al., *Genetic variability of* Aedes Aegypti *in the Americas using a mitochondrial gene: evidence of multiple introductions.* Memorias Do Instituto Oswaldo Cruz, 2007. **102**(5): p. 573-580.

260. Bracco, J.E. and A.L. Dalfabbro, *[Single-Larva Sampling for* Aedes-Aegypti *Surveillance].* Revista de Saude Publica, 1995. **29**(2): p. 144-146.

261. Braga, I.A., et al., *[Comparative study between larval surveys and ovitraps to monitor populations of* Aedes aegypti*].* Revista da Sociedade Brasileira de Medicina Tropical, 2000. **33**(4): p. 347-353.

262. Braga, I.A., et al., Aedes Aegypti *resistance to temephos during 2001 in several municipalities in the states of Rio de Janeiro, Sergipe, and Alagoas, Brazil.* Memorias Do Instituto Oswaldo Cruz, 2004. **99**(2): p. 199-203.

263. Braga, I.A., et al., *Effectiveness of methoprene, an insect growth regulator, against temephos-resistant* Aedes Aegypti *populations from different Brazilian localities, under laboratory conditions.* Journal of Medical Entomology, 2005. **42**(5): p. 830-837.

264. Braks, M.A.H., et al., *Interspecific competition between two invasive species of container mosquitoes,* Aedes Aegypti *and* Aedes albopictus *(Diptera : Culicidae), in Brazil.* Annals of the Entomological Society of America, 2004. **97**(1): p. 130-139.

265. Braks, M.A.H., et al., *Convergent habitat segregation of* Aedes Aegypti *and* Aedes albopictus *(Diptera : Culicidae) in southeastern Brazil and Florida.* Journal of Medical Entomology, 2003. **40**(6): p. 785-794.

266. Brengues, C., et al., *Pyrethroid and DDT cross-resistance in* Aedes Aegypti *is correlated with novel mutations in the voltage-gated sodium channel gene.* Medical and Veterinary Entomology, 2003. **17**(1): p. 87-94.

267. Brown, M.D., et al., *Chlorine Tolerance of* Mesocyclops *(Cyclopoida - Cyclopidae) Copepods and 3 Container-Breeding Species of Mosquitos.* Environmental Entomology, 1994. **23**(5): p. 1245-1249.

268. Bryant, J.E., et al., *Size heterogeneity in the 3' noncoding region of south American isolates of yellow fever virus.* Journal of Virology, 2005. **79**(6): p. 3807-3821.

269. Buerano, C.C., et al., *IgM-capture ELISA of serum samples collected from Filipino dengue patients.* Southeast Asian Journal of Tropical Medicine and Public Health, 2000. **31**(3): p. 524-529.

270. Burkett-Cadena, N.D. and G.R. Mullen, *Field comparison of Bermuda-hay infusion to infusions of emergent aquatic vegetation for collecting female mosquitoes.* Journal of the American Mosquito Control Association, 2007. **23**(2): p. 117-123.

271. Burkett-Cadena, N.D. and G.R. Mullen, *Comparison of infusions of commercially available garden products for collection of container-breeding mosquitoes.* Journal of the American Mosquito Control Association, 2008. **24**(2): p. 236-243.

272. Burkot, T.R., et al., *Productivity of natural and artificial containers for* Aedes polynesiensis *and* Aedes Aegypti *in four American Samoan villages.* Medical and Veterinary Entomology, 2007. **21**(1): p. 22-29.

273. Burton, G.J., *Coastal Survey of Aedes Aegypti-Breeding in British Guiana.* Annals of Tropical Medicine and Parasitology, 1963. **57**(4): p. 446-451.

274. Cabrera, B.D. and F. Valeza, *Distribution and density of mosquitoes in two endemic areas for bancroftian filariasis in Sorsogon, Philippines.* Southeast Asian Journal of Tropical Medicine and Public Health, 1978. **9**(3): p. 398-405.

275. Cabrera, M. and K. Jaffe, *An aggregation pheromone modulates lekking behavior in the vector mosquito* Aedes Aegypti *(Diptera: Culicidae).* Journal of the American Mosquito Control Association, 2007. **23**(1): p. 1-10.

276. Calado, D.C. and M.A. Silva, *[Evaluation of the temperature influence on the development of* Aedes albopictus*].* Revista de Saúde Pública, 2002. **36**(2): p. 173-179.

277. Calheiros, C.M.L., et al., *Experimental infection of* Culex (Culex) quinquefasciatus *and* Aedes (Stegomyia) aegypti *with* Wuchereria bancrofti*.* Memorias Do Instituto Oswaldo Cruz, 1998. **93**(6): p. 855-860.

278. Camacho, D.E., et al., *[Laboratory diagnosis of dengue virus infections in Aragua State, Venezuela: October 1997-December 1998].* Investigacion Clinica 2003. **44**(2): p. 91-103.

279. Camargo-Neves, V.L., et al., *Entomological investigation of a sylvatic yellow fever area in Sao Paulo State, Brazil.* Cadernos de Saúde Pública, 2005. **21**(4): p. 1278-1286.

280. Campos, J. and C.F. Andrade, *[Larval susceptibility to chemical insecticides of two* Aedes Aegypti *populations].* Revista de Saúde Pública, 2001. **35**(3): p. 232-236.

281. Campos, J. and C.F. Andrade, *[Larval susceptibility of* Aedes Aegypti *and* Culex quinquefasciatus *populations to chemical insecticides].* Revista de Saúde Pública, 2003. **37**(4): p. 523-527.

282. Cancrini, G., et al., Aedes albopictus *is a natural vector of* Dirofilaria immitis *in Italy.* Veterinary Parasitology, 2003. **118**(3-4): p. 195-202.

283. Cancrini, G., et al., *Development of* Dirofilaria and Setaria nematodes *in* Aedes albopictus*.* Parassitologia, 1995. **37**(2-3): p. 141-145.

284. Cancrini, G., et al., Aedes albopictus *and* Culex pipiens *implicated as natural vectors of* Dirofilaria repens *in central Italy.* Journal of Medical Entomology, 2007. **44**(6): p. 1064-1066.

285. Canyon, D.V. and J.L. Hii, *The Mossie-Buster: a hose-driven insecticide delivery tool for the control of container-breeding mosquitoes.* Journal of the American Mosquito Control Association, 1997. **13**(4): p. 389-394.

286. Carbajo, A.E., et al., *[Spatio-temporal variability in the transmission of dengue in Buenos Aires City].* Medicina 2004. **64**(3): p. 231-234.

287. Carbajo, A.E., et al., *[Spatio-temporal variability in the transmission of dengue in Buenos Aires City].* Medicina (B Aires), 2004. **64**(3): p. 231-234.

288. Cardenas, V. *DENGUE/DHF - COLOMBIA*. IQCB-Biweekly Report of Cases and Outbreaks 1995 [cited 1995 3 September].

289. Cardoso Junior, R.P., et al., *[Detection of* Aedes Aegypti *and* Aedes albopictus*, in an urban zone of the municipality of Catanduva, SP, after control of a Dengue epidemic].* Revista da Sociedade Brasileira de Medicina Tropical, 1996. **30**(1): p. 37-40.

290. Cardoso Junior, R.P., et al., *[Detection of* Aedes Aegypti *and* Aedes albopictus*, in an urban zone of the municipality of Catanduva, SP, after control of a Dengue epidemic].* Revista da Sociedade Brasileira de Medicina Tropical, 1996. **30**(1): p. 37-40.

291. Carles, G., H. Peiffer, and A. Talarmin, *Effects of dengue fever during pregnancy in French Guiana.* Clinical Infectious Diseases 1999. **28**(3): p. 637-640.

292. Carlos, M. and A.-L. Banks *Dengue/DHF update 2007 (13)*. PROMED, 2007.

293. Carrieri, M., et al., *On the competition occurring between* Aedes albopictus *and* Culex pipiens *(Diptera : Culicidae) in Italy.* Environmental Entomology, 2003. **32**(6): p. 1313-1321.

294. Carrieri, M., et al., *Tolerance thresholds for* Aedes albopictus *and* Aedes caspius *in Italian urban areas.* Journal of the American Mosquito Control Association, 2008. **24**(3): p. 377-386.

295. Carvalho Mdo, S., et al., *Susceptibility of* Aedes Aegypti *larvae to the insecticide temephos in the Federal District, Brazil.* Revista de Saúde Pública, 2004. **38**(5): p. 623-629.

296. Casas-Martinez, M. and J. Torres-Estrada, *First evidence of* Aedes albopictus *(Skuse) in Southern Chiapas, Mexico.* Emerging Infectious Diseases, 2003. **9**(5): p. 606-607.

297. Castle, T., et al., *Absence of impact of aerial malathion treatment on* Aedes Aegypti *during a dengue outbreak in Kingston, Jamaica.* Revista Panamericana de Salud Publica, 1999. **5**(2): p. 100-105.

298. Castle, T., et al., *Absence of impact of aerial malathion treatment on* Aedes Aegypti *during a dengue outbreak in Kingston, Jamaica.* Revista Panamericana de Salud Pública, 1999. **5**(2): p. 100-105.

299. Castro, M., N. Quintana, and P.M. Quinones, *[Evaluating two pyrethroids in dengue vector control in Putumayo, Colombia].* Revista de Saude Publica (Bogota), 2007. **9**(1): p. 106-116.

300. Castro, M.G., et al., *Dengue virus detection by using reverse transcription-polymerase chain reaction in saliva and progeny of experimentally infected* Aedes albopictus *from Brazil.* Memorias do Instituto Oswaldo Cruz, 2004. **99**(8): p. 809-814.

301. Cebret, A. and R. Desire, *[Anti-*Aedes Aegypti *campaign in French Guiana].* Bulletin De La Societe De Pathologie Exotique, 1996. **89**(2): p. 148-152; discussion 153.

302. Cecilio, A.B., et al., *Natural vertical transmission by* Stegomyia albopicta *as dengue vector in Brazil.* Brazilian Journal of Biology, 2009. **69**(1): p. 123-127.

303. Cha, S.J., D.D. Chadee, and D.W. Severson, *Population dynamics of an endogenous meiotic drive system in* Aedes Aegypti *in Trinidad.* American Journal of Tropical Medicine and Hygiene, 2006. **75**(1): p. 70-77.

304. Chabasse, D., et al., *[Encephalitic syndrome caused by flavivirus diagnosed after return from a voyage to South India: probably dengue with neurological manifestations].* Bulletin de la Societe de Pathologie Exotique et de ses Filiales, 1986. **79**(4): p. 531-538.

305. Chadee, D. AEDES ALBOPICTUS *- TRINIDAD (02)*. PROMED, 2004.

306. Chadee, D.D., *Effects of 'closed' houses on the* Aedes Aegypti *eradication programme in Trinidad.* Medical and Veterinary Entomology, 1988. **2**(2): p. 193-198.

307. Chadee, D.D., *Landing periodicity of the mosquito* Aedes Aegypti *in Trinidad in relation to the timing of insecticidal space-spraying.* Medical and Veterinary Entomology, 1988. **2**(2): p. 189-192.

308. Chadee, D.D., *[Methods for evaluating* Aedes Aegypti *populations and insecticide treatment in a town of Trinidad, West Indies].* Boletín de la Oficina Sanitaria Panamericana, 1990. **109**(4): p. 350-359.

309. Chadee, D.D., *Seasonal incidence and vertical distribution patterns of oviposition by* Aedes Aegypti *in an urban environment in Trinidad, W. I.* Journal of the American Mosquito Control Association, 1991. **7**(3): p. 383-386.

310. Chadee, D.D., *Seasonal incidence and horizontal distribution patterns of oviposition by* Aedes Aegypti *in an urban environment in Trinidad, west Indies.* Journal of the American Mosquito Control Association, 1992. **8**(3): p. 281-284.

311. Chadee, D.D., *Surveillance for the dengue vector* Aedes Aegypti *in Tobago, West Indies.* Journal of the American Mosquito Control Association, 2003. **19**(3): p. 199-205.

312. Chadee, D.D., *Observations on the seasonal prevalence and vertical distribution patterns of oviposition by* Aedes Aegypti *(L.) (Diptera: Culicidae) in urban high-rise apartments in Trinidad, West Indies.* Journal of Vector Ecology

, 2004. **29**(2): p. 323-330.

313. Chadee, D.D., *Key premises, a guide to* Aedes Aegypti *(Diptera: Culicidae) surveillance and control.* Bulletin of Entomological Research, 2004. **94**(3): p. 201-207.

314. Chadee, D.D., *Impact of pre-seasonal focal treatment on population densities of the mosquito* Aedes Aegypti *in Trinidad, West Indies: a preliminary study.* Acta Tropica, 2009. **109**(3): p. 236-240.

315. Chadee, D.D., *Dengue cases and* Aedes Aegypti *indices in Trinidad, West Indies.* Acta Tropica, 2009. **112**(2): p. 174-180.

316. Chadee, D.D. and J.C. Beier, *Natural variation in blood-feeding kinetics of four mosquito vectors.* Journal of Vector Ecology, 1996. **21**(2): p. 150-155.

317. Chadee, D.D. and J.C. Beier, *Factors influencing the duration of blood-feeding by laboratory-reared and wild* Aedes Aegypti *(Diptera: Culicidae) from Trinidad, West Indies.* Annals of Tropical Medicine and Parasitology, 1997. **91**(2): p. 199-207.

318. Chadee, D.D. and P.S. Corbet, *Seasonal incidence and diel patterns of oviposition in the field of the mosquito,* Aedes Aegypti *(L.) (Diptera:Culicidae) in Trinidad, West Indies: a preliminary study.* Annals of Tropical Medicine and Parasitology, 1987. **81**(2): p. 151-161.

319. Chadee, D.D. and P.S. Corbet, *Diel pattern of oviposition in the laboratory of the mosquito* Aedes albopictus *(Skuse) (Diptera: Culicidae).* Annals of Tropical Medicine and Parasitology, 1989. **83**(4): p. 423-429.

320. Chadee, D.D. and P.S. Corbet, *A night-time role of the oviposition site of the mosquito,* Aedes Aegypti *(L.) (Diptera: Culicidae).* Annals of Tropical Medicine and Parasitology, 1990. **84**(5): p. 429-433.

321. Chadee, D.D. and P.S. Corbet, *Diel patterns of oviposition indoors of the mosquito,* Aedes Aegypti *(L.) (Diptera: Culicidae) in Trinidad, W.I.: a preliminary study.* Annals of Tropical Medicine and Parasitology, 1990. **84**(1): p. 79-84.

322. Chadee, D.D. and P.S. Corbet, *The gonotrophic status of female* Aedes Aegypti *(L.) overnight at the oviposition site (Diptera: Culicidae).* Annals of Tropical Medicine and Parasitology, 1991. **85**(4): p. 461-466.

323. Chadee, D.D. and P.S. Corbet, *The gonotrophic status and diel pattern of entry to outdoor oviposition sites of female* Aedes Aegypti *(L.) (Diptera: Culicidae).* Annals of Tropical Medicine and Parasitology, 1993. **87**(3): p. 263-268.

324. Chadee, D.D., P.S. Corbet, and H. Talbot, *Proportions of eggs laid by* Aedes Aegypti *on different substrates within an ovitrap in Trinidad, West Indies.* Medical and Veterinary Entomology, 1995. **9**(1): p. 66-70.

325. Chadee, D.D., R. Doon, and D.W. Severson, *Surveillance of dengue fever cases using a novel* Aedes Aegypti *population sampling method in Trinidad, West Indies: the cardinal points approach.* Acta Tropica, 2007. **104**(1): p. 1-7.

326. Chadee, D.D., F.H. Fat, and R.C. Persad, *First record of* Aedes albopictus *from Trinidad, West Indies.* Journal of the American Mosquito Control Association, 2003. **19**(4): p. 438-439.

327. Chadee, D.D., et al., *Oviposition response of* Aedes Aegypti *mosquitoes to different concentrations of hay infusion in Trinidad, West Indies.* Journal of the American Mosquito Control Association, 1993. **9**(3): p. 346-348.

328. Chadee, D.D. and R. Martinez, *Landing periodicity of* Aedes Aegypti *with implications for dengue transmission in Trinidad, West Indies.* Journal of Vector Ecology

, 2000. **25**(2): p. 158-163.

329. Chadee, D.D., F.L.R. Williams, and U.D. Kitron, *Impact of vector control on a dengue fever outbreak in Trinidad, West Indies, in 1998.* Tropical Medicine & International Health, 2005. **10**(8): p. 748-754.

330. Chambers, G.M. and M.J. Klowden, *Nutritional reserves of autogenous and anautogenous selected strains of* Aedes albopictus *(Diptera: Culicidae).* Journal of Medical Entomology, 1994. **31**(4): p. 554-560.

331. Chan, A.S.T., et al., *Development of an indicator to evaluate the impact, on a community-based* Aedes Aegypti *control intervention, of improved cleaning of water-storage containers by householders.* Annals of Tropical Medicine and Parasitology, 1998. **92**(3): p. 317-329.

332. Chan, K.L., B.C. Ho, and Y.C. Chan, Aedes-Aegypti *(L.) and* Aedes-Albopictus *(Skuse) in Singapore City .2. Larval Habitats.* Bulletin of the World Health Organization, 1971. **44**(5): p. 629-633.

333. Chan, Y.C., K.L. Chan, and B.C. Ho, Aedes-Aegypti *(L.) and* Aedes-Albopictus *(Skuse) in Singapore-City .1. Distribution and Density.* Bulletin of the World Health Organization, 1971. **44**(5): p. 617-627.

334. Chan, Y.C. and S. Lam *CHIKUNGUNYA - MALAYSIA (PORT KLANG)*. PROMED, 1999.

335. Chang, A.Y., et al., *Combining Google Earth and GIS mapping technologies in a dengue surveillance system for developing countries.* International Journal of Health Geographics, 2009. **8**: p. 49.

336. Chang, L.H., et al., *Differential survival of* Aedes Aegypti *and* Aedes albopictus *(Diptera: Culicidae) larvae exposed to low temperatures in Taiwan.* Journal of Medical Entomology, 2007. **44**(2): p. 205-210.

337. Chang, M.S., et al., *Changes in abundance and behaviour of vector mosquitoes induced by land use during the development of an oil palm plantation in Sarawak.* Transactions of the Royal Society of Tropical Medicine and Hygiene, 1997. **91**(4): p. 382-386.

338. Chang, M.S., S. Lian, and N. Jute, *A small scale field trial with expanded polystyrene beads for mosquito control in septic tanks.* Transactions of the Royal Society of Tropical Medicine and Hygiene, 1995. **89**(2): p. 140-141.

339. Chansang, C. and P. Kittayapong, *Application of mosquito sampling count and geospatial methods to improve dengue vector surveillance.* American Journal of Tropical Medicine and Hygiene, 2007. **77**(5): p. 897-902.

340. Chareonviriyaphap, T., et al., *Allozyme patterns of* Aedes albopictus*, a vector of dengue in Thailand.* Journal of Medical Entomology, 2004. **41**(4): p. 657-663.

341. Chareonviriyaphap, T., et al., *Larval habitats and distribution patterns of* Aedes Aegypti *(Linnaeus) and* Aedes albopictus *(Skuse), in Thailand.* Southeast Asian Journal of Tropical Medicine and Public Health, 2003. **34**(3): p. 529-535.

342. Chareonviriyaphap, T. and K. Lerdthusnee, *Genetic differentiation of* Aedes Aegypti *mainland and island populations from southern Thailand.* Journal of the American Mosquito Control Association, 2002. **18**(3): p. 173-177.

343. Cheah, W.L., M.S. Chang, and Y.C. Wang, *Spatial, environmental and entomological risk factors analysis on a rural dengue outbreak in Lundu District in Sarawak, Malaysia.* Tropical Biomedicine, 2006. **23**(1): p. 85-96.

344. Chen, C.D., et al., *Preliminary study on the effectiveness of mosquito repelling lamp, E Da.* Tropical Biomedicine, 2007. **24**(2): p. 89-91.

345. Chen, C.D., et al., *Dengue vector surveillance in urban residential and settlement areas in Selangor, Malaysia.* Tropical Biomedicine, 2005. **22**(1): p. 39-43.

346. Chen, C.D., et al., *Mixed breeding of* Aedes Aegypti *(L.) and* Aedes albopictus *Skuse in four dengue endemic areas in Kuala Lumpur and Selangor, Malaysia.* Tropical Biomedicine, 2006. **23**(2): p. 224-227.

347. Chen, C.D., et al., *Weekly variation on susceptibility status of* Aedes *mosquitoes against temephos in Selangor, Malaysia.* Tropical Biomedicine, 2005. **22**(2): p. 195-206.

348. Chen, H. and H.B. Chen, *[Changes in element content in* Aedes albopictus *infected by dengue virus type II].* Chinese Journal of Parasitology & Parasitic Diseases - Zhongguo Ji Sheng Chong Xue Yu Ji Sheng Chong Bing Za Zhi, 2002. **20**(3): p. 171-173.

349. Chen, H. and H.B. Chen, *[Sequences analysis of cytochrome C oxidase subunit I gene in* Aedes albopictus *from different geographic strains in China].* Chinese Journal of Epidemiology - Zhonghua Liu Xing Bing Xue Za Zhi 2003. **24**(6): p. 491-493.

350. Chen, W.J., et al., *[A study on transovarial transmission of dengue type 1 virus in* Aedes aegypti*].* Chinese Journal of Microbiology and Immunology - Zhonghua Min Guo Wei Sheng Wu Ji Mian Yi Xue Za Zhi 1990. **23**(4): p. 259-270.

351. Chen, W.J., et al., *Vector competence of* Aedes albopictus *and* Ae. aegypti *(Diptera: Culicidae) to dengue 1 virus on Taiwan: development of the virus in orally and parenterally infected mosquitoes.* Journal of Medical Entomology 1993. **30**(3): p. 524-530.

352. Cheong, C.Y. *DENGUE - MALAYSIA: CONTROL*. 1999 [cited 1999 19 February]; New Straits Times, Tuesday, 2 Feb 1999 [edited].

353. Chin, J. *YELLOW FEVER - BRAZIL (03)*. PROMED, 1998.

354. Chippaux, A., et al., *[Several yellow fever cases in an endemic area in Ivory Coast: serological and epidemiological evidence (author's transl)].* Medecine Tropicale, 1981. **41**(1): p. 53-61.

355. Chow, V.T., et al., *Monitoring of dengue viruses in field-caught* Aedes Aegypti *and* Aedes albopictus *mosquitoes by a type-specific polymerase chain reaction and cycle sequencing.* American Journal of Tropical Medicine and Hygiene, 1998. **58**(5): p. 578-586.

356. Chowdhury, M. *DENGUE/DHF - BANGLADESH (02)*. 2000 [cited 2000 31 January].

357. Chowdhury, M.A. *Dengue in Dhaka up-date, 20 Jul 2000* PROMED, 2000.

358. Chung, Y.K. and F.Y. Pang, *Dengue virus infection rate in field populations of female* Aedes Aegypti *and* Aedes albopictus *in Singapore.* Tropical Medicine & International Health, 2002. **7**(4): p. 322-330.

359. Chungue, E., et al., *Dengue 1 epidemic in French Polynesia, 1988-1989: surveillance and clinical, epidemiological, virological and serological findings in 1752 documented clinical cases.* Transactions of the Royal Society of Tropical Medicine and Hygiene, 1992. **86**(2): p. 193-197.

360. Cianchi, R., et al., *Isocitrate dehydrogenase in* Aedes aegypti*: formal genetics, preliminary linkage data and study of natural populations.* Parassitologia, 1978. **20**(1-3): p. 47-58.

361. Clark, G.G., H. Seda, and D.J. Gubler, *Use of the "CDC backpack aspirator" for surveillance of* Aedes Aegypti *in San Juan, Puerto Rico.* Journal of the American Mosquito Control Association, 1994. **10**(1): p. 119-124.

362. Coder, D. *DENGUE - VENEZUELA (12)*. 1997 [cited 1997 2 December]; El Nacional; Caracas, Venezuela; 29 Nov 1997.

363. Coder, D. *DENGUE, HEMORRHAGIC - COSTA RICA*. 1997 [cited 1997 23 September].

364. Coder, D. *DENGUE - VENEZUELA (12)* PROMED, 1997.

365. Coelho, G.E., et al., *Dynamics of the 2006/2007 dengue outbreak in Brazil.* Memorias Do Instituto Oswaldo Cruz, 2008. **103**(6): p. 535-U7.

366. Coffinet, T., et al., *First record of* Aedes albopictus *in Gabon.* Journal of the American Mosquito Control Association, 2007. **23**(4): p. 471-472.

367. Colton, Y.M., D.D. Chadee, and D.W. Severson, *Natural skip oviposition of the mosquito* Aedes Aegypti *indicated by codominant genetic markers.* Medical and Veterinary Entomology, 2003. **17**(2): p. 195-204.

368. Comiskey, N. and D.M. Wesson, *Dirofilaria (Filarioidea, Onchocercidae) Infection in* Aedes-Albopictus *(Diptera, Culicidae) Collected in Louisiana.* Journal of Medical Entomology, 1995. **32**(5): p. 734-737.

369. Comiskey, N.M., R.C. Lowrie, and D.M. Wesson, *Role of habitat components on the dynamics of* Aedes albopictus *(Diptera : Culicidae) from New Orleans.* Journal of Medical Entomology, 1999. **36**(3): p. 313-320.

370. Conway, G.R., M. Trpis, and Mcclella.Ga, *Population Parameters of Mosquito* Aedes-Aegypti *(L) Estimated by Mark-Release-Recapture in a Suburban Habitat in Tanzania.* Journal of Animal Ecology, 1974. **43**(2): p. 289-304.

371. Cook, S., et al., *Isolation of a new strain of the flavivirus cell fusing agent virus in a natural mosquito population from Puerto Rico.* Journal of General Virology, 2006. **87**(Pt 4): p. 735-748.

372. Cookman, J.E. and R.A. Lebrun, Aedes-Aegypti *Larvae in Portsmouth, Rhode-Island.* Journal of the American Mosquito Control Association, 1986. **2**(1): p. 96-97.

373. Cooper, R.D., et al., Aedes-Albopictus *(Skuse) (Diptera, Culicidae) in the Western Province of Papua-New-Guinea and the Threat of Its Introduction to Australia.* Journal of the Australian Entomological Society, 1994. **33**: p. 115-116.

374. Corbet, P.S. and D.D. Chadee, *Incidence and diel pattern of oviposition outdoors of the mosquito,* Aedes Aegypti *(L.) (Diptera: Culicidae) in Trinidad, W.I. in relation to solar aspect.* Annals of Tropical Medicine and Parasitology, 1990. **84**(1): p. 63-78.

375. Corbet, P.S. and D.D. Chadee, *The diel pattern of entry to outdoor oviposition sites by female* Aedes Aegypti *(L.) (Diptera: Culicidae) that then laid eggs there: a preliminary study.* Annals of Tropical Medicine and Parasitology, 1992. **86**(5): p. 523-528.

376. Corbet, P.S. and S.M. Smith, *Diel Periodicities of Landing of Nulliparous and Parous* Aedes-Aegypti *(L) at Dar Es Salaam, Tanzania(Diptera, Culicidae).* Bulletin of Entomological Research, 1974. **64**(1): p. 111-121.

377. Cordellier, R., *[The epidemiology of yellow fever in Western Africa].* Bulletin of the World Health Organization, 1991. **69**(1): p. 73-84.

378. Cornel, A.J. and R.H. Hunt, Aedes albopictus *in Africa? First records of live specimens in imported tires in Cape Town.* Journal of the American Mosquito Control Association, 1991. **7**(1): p. 107-108.

379. Correa, P.R.L., E. Franca, and T.F. Bogutchi, *[*Aedes Aegypti *infestation and occurrence of dengue in the city of Belo Horizonte, Brazil].* Revista de Saude Publica, 2005. **39**(1): p. 33-40.

380. Cosgriff, M. *DENGUE/DHF: UPDATES, 30 SEP 2000*. PROMED, 2000.

381. Costa, F.S., et al., *[Population dynamics of* Aedes Aegypti *(L) in an urban area with high incidence of dengue].* Revista da Sociedade Brasileira de Medicina Tropical, 2008. **41**(3): p. 309-312.

382. Costanzo, K.S., K. Mormann, and S.A. Juliano, *Asymmetrical competition and patterns of abundance of* Aedes albopictus *and* Culex pipiens *(Diptera : Culicidae).* Journal of Medical Entomology, 2005. **42**(4): p. 559-570.

383. Costero, A., et al., *Survival of starved* Aedes Aegypti *(Diptera: Culicidae) in Puerto Rico and Thailand.* Journal of Medical Entomology, 1999. **36**(3): p. 272-276.

384. Costero, A., et al., *Life table study of* Aedes Aegypti *(Diptera: Culicidae) in Puerto Rico fed only human blood versus blood plus sugar.* Journal of Medical Entomology, 1998. **35**(5): p. 809-813.

385. Craig, G.B. and W.A. Hickey, *Genetic Variability in* Aedes Aegypti *(Diptera - Culicidae) .4. Mutation Load in Some African Populations.* Annals of the Entomological Society of America, 1966. **59**(6): p. 1228-1234.

386. Craig, S., et al., *Diverse dengue type 2 virus populations contain recombinant and both parental viruses in a single mosquito host.* Journal of Virology, 2003. **77**(7): p. 4463-4467.

387. Crans, W.J., et al., *First record of* Aedes albopictus *from New Jersey.* Journal of the American Mosquito Control Association, 1996. **12**(2): p. 307-309.

388. Cuddehe, M., *Mexico fights rise in dengue fever.* Lancet, 2009. **374**(9690): p. 602.

389. Cuellar-Jimenez, M.E., et al., *[Detection of* Aedes albopictus *(Skuse) (Diptera: Culicidae) in the city of Cali, Valle del Cauca, Colombia.].* Biomedica, 2007. **27**(2): p. 273-279.

390. Cui, K.L., *[The Autogeny of* Aedes-Albopictus *in Guangzhou Area].* Acta Entomologica Sinica, 1982. **25**(3): p. 256-259.

391. Cully, J.F., Jr., et al., *Antibodies to La Crosse virus in eastern chipmunks in Indiana near an* Aedes albopictus *population.* Journal of the American Mosquito Control Association, 1991. **7**(4): p. 651-653.

392. Cupp, E.W., et al., *West Nile virus infection in mosquitoes in the Mid-South USA, 2002-2005.* Journal of Medical Entomology, 2007. **44**(1): p. 117-125.

393. Curtin, T.J., *Status of* Aedes Aegypti *in Eastern Mediterranean.* Journal of Medical Entomology, 1967. **4**(1): p. 48-50.

394. Curtis, C.F., et al., *Comparative field cage tests of the population suppressing efficiency of three genetic control systems for* Aedes Aegypti*.* Heredity, 1976. **36**(1): p. 11-29.

395. da Costa-da-Silva, A.L., M.L. Capurro, and J.E. Bracco, *Genetic lineages in the yellow fever mosquito* Aedes (Stegomyia) aegypti *(Diptera: Culicidae) from Peru.* Memorias Do Instituto Oswaldo Cruz, 2005. **100**(6): p. 539-544.

396. da Costa-Ribeiro, M.C.V., R. Lourenco-de-Oliveira, and A.B. Failloux, *Higher genetic variation estimated by microsatellites compared to isoenzyme markers in* Aedes Aegypti *from Rio de Janeiro.* Memorias Do Instituto Oswaldo Cruz, 2006. **101**(8): p. 917-921.

397. da Costa-Ribeiro, M.C.V., R. Lourenco-De-Oliveira, and A.B. Failloux, *Geographic and temporal genetic patterns of* Aedes Aegypti *populations in Rio de Janeiro, Brazil.* Tropical Medicine & International Health, 2006. **11**(8): p. 1276-1285.

398. da Costa-Ribeiro, M.C.V., R. Lourenco-De-Oliveira, and A.B. Failloux, *Low gene flow of* Aedes Aegypti *between dengue-endemic and dengue-free areas in southeastern and southern Brazil.* American Journal of Tropical Medicine and Hygiene, 2007. **77**(2): p. 303-309.

399. da Cunha, R.V., et al., *Dengue infection in Paracambi, State of Rio de Janeiro, 1990-1995.* Revista da Sociedade Brasileira de Medicina Tropical, 1997. **30**(5): p. 379-383.

400. da Cunha, R.V., et al., *Dengue infection in Paracambi, State of Rio de Janeiro, 1990-1995.* Revista da Sociedade Brasileira de Medicina Tropical, 1997. **30**(5): p. 379-383.

401. da Cunha, R.V., et al., *Retrospective study on dengue in Fortaleza, state of Ceara, Brazil.* Memorias do Instituto Oswaldo Cruz, 1998. **93**(2): p. 155-159.

402. da Silva, A.M., *[Domestic water reservoir as breeding site of* Aedes aegypti*].* Revista de Saude Publica, 2004. **38**(1): p. 139-140.

403. da Silva, V.C., et al., *[Diversity of oviposition containers and buildings where* Aedes albopictus *and* Aedes Aegypti *can be found].* Revista de Saúde Pública, 2006. **40**(6): p. 1106-1011.

404. da-Cunha, M.P., et al., *Monitoring of resistance to the pyrethroid cypermethrin in Brazilian* Aedes Aegypti *(Diptera : Culicidae) populations collected between 2001 and 2003.* Memorias Do Instituto Oswaldo Cruz, 2005. **100**(4): p. 441-444.

405. Dalla Pozza, G. and G. Majori, *First record of* Aedes albopictus *establishment in Italy.* Journal of the American Mosquito Control Association, 1992. **8**(3): p. 318-320.

406. Dalla Pozza, G.L., R. Romi, and C. Severini, *Source and spread of* Aedes albopictus *in the Veneto region of Italy.* Journal of the American Mosquito Control Association, 1994. **10**(4): p. 589-592.

407. Danis-Lozano, R., M.H. Rodriguez, and M. Hernandez-Avila, *Gender-related family head schooling and* Aedes Aegypti *larval breeding risk in southern Mexico.* Salud Pública de México, 2002. **44**(3): p. 237-242.

408. Dantes, H.G., et al., *Dengue epidemics on the Pacific Coast of Mexico.* International Journal of Epidemiology, 1988. **17**(1): p. 178-186.

409. Darsie, R.F., *Description of the pupa of* Aedes cretinus *Edwards, a key to the pupae of the albopictus subgroup, subgenus Stegomyia Theobald, genus Aedes Meigen, and characters to separate the European Stegomyia species (Diptera : Culicidae).* Proceedings of the Entomological Society of Washington, 1999. **101**(3): p. 614-618.

410. Das, P.K. and P.K. Rajagopalan, *Susceptibility of Larvae of* Culex-Fatigans *(Wiedmann),* Anopheles-Stephensi *(Liston) and* Aedes-Aegypti *(Linn) to Insecticides in Pondicherry.* Indian Journal of Medical Research, 1979. **70**(Sep): p. 412-416.

411. Daza, E., et al. *VENEZUELAN EQUINE ENCEPHALITIS - COLOMBIA (2)*. PROMED, 1995.

412. de Albuquerque, C.M., et al., *[First report of* Aedes albopictus *in areas of Mata Atlantica, Recife, PE, Brazil].* Revista de Saúde Pública, 2000. **34**(3): p. 314-315.

413. de Almeida, P.S., et al., *[Spatial distribution of* Aedes albopictus *in the southern area of Mato Grosso do Sul State, Brazil].* Revista de Saúde Pública, 2006. **40**(6): p. 1094-1100.

414. de Andrande, C.F. and M. Modolo, *Susceptibility of* Aedes Aegypti *larvae to temephos and Bacillus thuringiensis var israelensis in integrated control.* Revista de Saúde Pública, 1991. **25**(3): p. 184-187.

415. De Benedictis, J., et al., *Identification of the people from whom engorged* Aedes Aegypti *took blood meals in Florida, Puerto Rico, using polymerase chain reaction-based DNA profiling.* American Journal of Tropical Medicine and Hygiene, 2003. **68**(4): p. 437-446.

416. de Brito, M. and S.P. Forattini, *[Productivity of* Aedes albopictus*' breeding containers in Paraiba Valley, Brazil].* Revista de Saude Publica, 2004. **38**(2): p. 209-215.

417. de Brito, M., et al., *[1st Finding of* Aedes (Stegomyia) albopictus *(Skuse) in the Sao-Paulo State (Brazil)].* Revista de Saude Publica, 1986. **20**(6): p. 489-489.

418. de Carvalho, G.A., C.F.S. Andrade, and M.T. Ueta, *Experimental infection of* Aedes albopictus *(Diptera : Culicidae) larvae with the xiphidiocercariae of a hematolechid.* Memorias Do Instituto Oswaldo Cruz, 2002. **97**(4): p. 573-578.

419. de Castro, M.G., et al., *Dengue virus detection by using reverse transcription-polymerase chain reaction in saliva and progeny of experimentally infected* Aedes albopictus *from Brazil.* Memorias Do Instituto Oswaldo Cruz, 2004. **99**(8): p. 809-814.

420. De Cock, K.M., et al., *Epidemic yellow fever in eastern Nigeria, 1986.* Lancet, 1988. **331**(8586): p. 630-633.

421. De Figueiredo, R.M., et al., *[Exanthematous diseases and the first epidemic of dengue to occur in Manaus, Amazonas State, Brazil, during 1998-1999].* Revista da Sociedade Brasileira de Medicina Tropical, 2004. **37**(6): p. 476-9.

422. de Filippis, A.M., et al., *Isolation and characterization of wild type yellow fever virus in cases temporally associated with 17DD vaccination during an outbreak of yellow fever in Brazil.* Vaccine, 2004. **22**(9-10): p. 1073-1078.

423. de Garin, A.B., et al., *Atmospheric control of* Aedes Aegypti *populations in Buenos Aires (Argentina) and its variability.* International Journal of Biometeorology, 2000. **44**(3): p. 148-156.

424. de la Cruz, A.M., A. Mesa, and J.L. San Martin, *[The community and the control of* Aedes aegypti*: perception and behavior regarding temephos larvicide].* Revista Cubana de Medicina Tropical, 2001. **53**(1): p. 44-47.

425. de Lima-Camara, T.N., N.A. Honorio, and R. Lourenco-de-Oliveira, *Parity and ovarian development of* Aedes Aegypti *and Ae. albopictus (Diptera: Culicidae) in metropolitan Rio de Janeiro.* Journal of Vector Ecology

, 2007. **32**(1): p. 34-40.

426. de Oliveira, R.L., et al., *Large genetic differentiation and low variation in vector competence for dengue and yellow fever viruses of* Aedes albopictus *from Brazil, the United States, and the Cayman Islands.* American Journal of Tropical Medicine and Hygiene, 2003. **69**(1): p. 105-114.

427. de Sousa, G.B., G. Aviles, and C.N. Gardenal, *Allozymic polymorphism in* Aedes Aegypti *populations from Argentina.* Journal of the American Mosquito Control Association, 2000. **16**(3): p. 206-209.

428. de Sousa, G.B., A. Blanco, and C.N. Gardenal, *Genetic relationships among* Aedes Aegypti *(Diptera: Culicidae) populations from Argentina using random amplified polymorphic DNA polymerase chain reaction markers.* Journal of Medical Entomology, 2001. **38**(3): p. 371-375.

429. de Sousa, R.D. and H.E.M.D. Bicudo, *Heterochromatic banding pattern in two Brazilian populations of* Aedes aegypti*.* Genetica, 1999. **105**(1): p. 93-99.

430. de Valdez, M.R.W., *Parasitoid-induced behavioral alterations of* Aedes Aegypti *mosquito larvae infected with mermithid nematodes (Nematoda : Mermithidae).* Journal of Vector Ecology, 2006. **31**(2): p. 344-354.

431. Degallier, N., et al., *First isolation of dengue 1 virus from* Aedes Aegypti *in Federal District, Brazil.* Revista da Sociedade Brasileira de Medicina Tropical, 2000. **33**(1): p. 95-96.

432. Degallier, N., et al., *First isolation of dengue 1 virus from* Aedes Aegypti *in Federal District, Brazil.* Revista da Sociedade Brasileira de Medicina Tropical, 2000. **33**(1): p. 95-96.

433. Degallier, N., et al., Aedes albopictus *may not be vector of dengue virus in human epidemics in Brazil.* Revista de Saude Publica, 2003. **37**(3): p. 386-387.

434. Degallier, N., et al., *People's knowledge and practice about dengue, its vectors, and control means in Brasilia (DF), Brazil: Its relevance with entomological factors.* Journal of the American Mosquito Control Association, 2000. **16**(2): p. 114-123.

435. Delatte, H., et al., *Geographic distribution and developmental sites of* Aedes albopictus *(Diptera : Culicidae) during a Chikungunya epidemic event.* Vector Borne and Zoonotic Diseases, 2008. **8**(1): p. 25-34.

436. Delatte, H., et al., *Influence of temperature on immature development, survival, longevity, fecundity, and gonotrophic cycles of* Aedes albopictus*, vector of chikungunya and dengue in the Indian Ocean.* Journal of Medical Entomology, 2009. **46**(1): p. 33-41.

437. Delatte, H., et al., *[*Aedes albopictus*, vector of chikungunya and dengue viruses in Reunion Island: biology and control].* Parasite-Journal De La Societe Francaise De Parasitologie, 2008. **15**(1): p. 3-13.

438. Dell Chism, B. and C.S. Apperson, *Horizontal transfer of the insect growth regulator pyriproxyfen to larval microcosms by gravid* Aedes albopictus *and Ochlerotatus triseriatus mosquitoes in the laboratory.* Medical and Veterinary Entomology, 2003. **17**(2): p. 211-220.

439. della Torre, A., C. Bomboi, and G. Cancrini, *[Extension of the area of* Aedes albopictus *in Italy. 1st report of the species in central Italy].* Parassitologia, 1992. **34**(1-3): p. 143-146.

440. Desena, M.L., et al., Aedes Aegypti *(Diptera: Culicidae) age determination by cuticular hydrocarbon analysis of female legs.* Journal of Medical Entomology, 1999. **36**(6): p. 824-830.

441. Deubel, V., et al., *Genetic heterogeneity of yellow fever virus strains from Africa and the Americas.* Journal of General Virology, 1986. **67 ( Pt 1)**: p. 209-213.

442. Deubel, V., et al., *Homogeneity among Senegalese strains of yellow fever virus.* American Journal of Tropical Medicine and Hygiene, 1985. **34**(5): p. 976-983.

443. Devine, G.J., et al., *The use of pyriproxyfen as a control agent for* Aedes Aegypti *in Peru.* American Journal of Tropical Medicine and Hygiene, 2006. **75**(5): p. 200-200.

444. Di Luca, M., et al., *[*Aedes albopictus *in Rome: monitoring in the 3-year period of 1998-2000].* Annali dell'Istituto Superiore di Sanità, 2001. **37**(2): p. 249-254.

445. Diallo, M., et al., *Vector competence of* Aedes Aegypti *populations from Senegal for sylvatic and epidemic dengue 2 virus isolated in West Africa.* Transactions of the Royal Society of Tropical Medicine and Hygiene, 2008. **102**(5): p. 493-498.

446. Diallo, M., et al., *Amplification of the sylvatic cycle of dengue virus type 2, Senegal, 1999-2000: entomologic findings and epidemiologic considerations.* Emerging Infectious Dieseases, 2003. **9**(3): p. 362-367.

447. Diallo, M., et al., *Amplification of the sylvatic cycle of dengue virus type 2, Senegal, 1999-2000: entomologic findings and epidemiologic considerations.* Emerging Infectious Diseases, 2003. **9**(3): p. 362-367.

448. Diallo, M., et al., *Potential role of sylvatic and domestic African mosquito species in dengue emergence.* American Journal of Tropical Medicine and Hygiene, 2005. **73**(2): p. 445-449.

449. Diallo, M., J. Thonnon, and D. Fontenille, *Vertical transmission of the yellow fever virus by* Aedes Aegypti *(Diptera, Culicidae): dynamics of infection in F1 adult progeny of orally infected females.* American Journal of Tropical Medicine and Hygiene, 2000. **62**(1): p. 151-156.

450. Diallo, M., J. Thonnon, and D. Fontenille, *Vertical transmission of the yellow fever virus by* Aedes Aegypti *(Diptera, Culicidae): Dynamics of infection in F-1 adult progeny of orally infected females.* American Journal of Tropical Medicine and Hygiene, 2000. **62**(1): p. 151-156.

451. Diallo, M., et al., *Vectors of Chikungunya virus in Senegal: current data and transmission cycles.* American Journal of Tropical Medicine and Hygiene, 1999. **60**(2): p. 281-286.

452. Diarrassouba, S. and J. Dossou-Yovo, *[Atypical activity rhythm in* Aedes Aegypti *in a sub-sudanian savannah zone of Cote d'Ivoire].* Bulletin De La Societe De Pathologie Exotique, 1997. **90**(5): p. 361-363.

453. Diaz, C., et al., *[An inter-sector participatory strategy in Cuba using an ecosystem approach to prevent dengue transmission at the local level].* Cadernos de Saúde Pública, 2009. **25 Suppl 1**: p. S59-70.

454. Dibo, M.R., et al., *Identification of the best ovitrap installation sites for gravid* Aedes (Stegomyia) aegypti *in residences in Mirassol, State of Sao Paulo, Brazil.* Memorias Do Instituto Oswaldo Cruz, 2005. **100**(4): p. 339-343.

455. Dibo, M.R., et al., *Study of the relationship between* Aedes (Stegomyia) aegypti *egg and adult densities, dengue fever and climate in Mirassol, state of Sao Paulo, Brazil.* Memorias Do Instituto Oswaldo Cruz, 2008. **103**(6): p. 554-560.

456. Dieng, H., et al., *A laboratory and field evaluation of Macrocyclops distinctus, Megacyclops viridis and Mesocyclops pehpeiensis as control agents of the dengue vector* Aedes albopictus *in a peridomestic area in Nagasaki, Japan.* Medical and Veterinary Entomology, 2002. **16**(3): p. 285-291.

457. Dinardo-Miranda, L.L. and E.P. Contel, *Enzymatic variability in natural populations of* Aedes Aegypti *(Diptera:Culicidae) from Brazil.* Journal of Medical Entomology, 1996. **33**(5): p. 726-733.

458. Djam, J.C. and D.A. Focks, *Susceptibility of* Toxorhynchites-Amboinensis *and* Aedes-Aegypti *to Several Adulticides Currently Used for Mosquito-Control.* Mosquito News, 1983. **43**(4): p. 471-473.

459. Domingues, R.B., et al., *Headache features in patients with dengue virus infection.* Cephalalgia, 2006. **26**(7): p. 879-882.

460. Domingues, R.B., et al., *Involvement of the central nervous system in patients with dengue virus infection.* Journal of the Neurological Sciences, 2008. **267**(1-2): p. 36-40.

461. Donatti, J.E. and A.D. Gomes, *[Adultrap: Description of adult trap of* Aedes Aegypti *(Diptera, Culicidae)].* Revista Brasileira De Entomologia, 2007. **51**(2): p. 255-256.

462. Donnelly, J.W., Aedes-Aegypti *in New-Jersey.* Journal of the American Mosquito Control Association, 1993. **9**(2): p. 238-238.

463. Donpedro, K.N. and T.O. Adegbite, *Nuvan Resistance in a Field Strain of* Aedes-Aegypti *(L) in Lagos, Nigeria.* Environmental Pollution Series a-Ecological and Biological, 1985. **38**(1): p. 19-29.

464. dos Passos, R.A. and W.P. Tadei, *Parasitism of* Ascogregarina taiwanensis *and* Ascogregarina culicis *(Apicomplexa : Lecudinidae) in larvae of* Aedes albopictus *and* Aedes Aegypti *(Diptera : Culicidae) from Manaus, Amazon region, Brazil.* Journal of Invertebrate Pathology, 2008. **97**(3): p. 230-236.

465. dos Santos, R.L., *[Updating of the distribution of* Aedes albopictus *in Brazil (1997-2002)].* Revista de Saude Publica, 2003. **37**(5): p. 671-673.

466. dos Santos, S.O. and J.C. do Nascimento, *[First register of* Aedes albopictus *presence in Mato Grosso do Sul, Brazil].* Revista de Saude Publica, 1998. **32**(5): p. 486-486.

467. dos Santos, V.M., et al., *Analysis of genetic relatedness between populations of* Aedes Aegypti *from different geographic regions of Sao Paulo state, Brazil.* Revista do Instituto de Medicina Tropical de São Paulo, 2003. **45**(2): p. 99-101.

468. Dottori, M. *CHIKUNGUNYA - ITALY (EMILIA ROMAGNA) (06)* PROMED, 2007.

469. Doyle, P. AEDES AEGYPTI *- USA (ARIZONA)*. PROMED, 2001.

470. Dua, V.K., et al., *Repellency of* Lantana camara *(Verbenaceae) flowers against Aedes mosquitoes.* Journal of the American Mosquito Control Association, 1996. **12**(3 Pt 1): p. 406-408.

471. Dua, V.K., B.N. Nagpal, and V.P. Sharma, *Repellent action of neem cream against mosquitoes.* Indian Journal of Malariology, 1995. **32**(2): p. 47-53.

472. Dudley, J., et al. *Dengue/DHF update 2007 (12)* PROMED, 2007.

473. Duenas, J.C.R., et al., *Restriction fragment-length polymorphism of the mtDNA A+T-rich region as a genetic marker in* Aedes Aegypti *(Diptera : Culicidae).* Annals of the Entomological Society of America, 2002. **95**(3): p. 352-358.

474. Duhrkopf, R.E., *A Survey of Container-Bredding Mosquitos in Mclennan County, Texas.* Texas Journal of Science, 1994. **46**(2): p. 127-132.

475. Duhrkopf, R.E. and H. Benny, *Differences in the larval alarm reaction in populations of* Aedes Aegypti *and* Aedes albopictus*.* Journal of the American Mosquito Control Association, 1990. **6**(3): p. 411-414.

476. Durand, J.P. *DENGUE/DHF UPDATE 2004 (03) [French Antilles/ Indonesia/ Tonga]*. 2004 [cited 2004 22 January].

477. Dutary, B.E., J.E. Rozette, and C. Campos, *[Current situation of the* Aedes Aegypti *mosquito in the metropolitan area of Panama city].* Revista Médica de Panamá, 1989. **14**(2): p. 67-78.

478. Dutta, P., et al., *Entomological observations on dengue vector mosquitoes following a suspected outbreak of dengue in certain parts of Nagaland with a note on their susceptibility to insecticides.* Journal of Environmental Biology, 2004. **25**(2): p. 209-212.

479. Dutta, P., et al., *Distribution of potential dengue vectors in major townships along the national highways and trunk roads of northeast India.* Southeast Asian Journal of Tropical Medicine and Public Health, 1998. **29**(1): p. 173-176.

480. Dye, C., *Competition Amongst Larval* Aedes-Aegypti *- the Role of Interference.* Ecological Entomology, 1984. **9**(3): p. 355-357.

481. Eamchan, P., et al., *Epidemiology and control of dengue virus infections in Thai villages in 1987.* American Journal of Tropical Medicine and Hygiene, 1989. **41**(1): p. 95-101.

482. Easton, E.R., *Urbanization and its effects on the ecology of mosquitoes in Macau, Southeast Asia.* Journal of the American Mosquito Control Association, 1994. **10**(4): p. 540-544.

483. Echevers, G., et al., *Results of spraying with ultra-low-volume malathion at ground level in Panama City.* Bulletin of the Pan American Health Organization, 1975. **9**(3): p. 232-237.

484. Edgerly, J.S., M.S. Willey, and T.P. Livdahl, *The Community Ecology of Aedes Egg Hatching - Implications for a Mosquito Invasion.* Ecological Entomology, 1993. **18**(2): p. 123-128.

485. Edman, J., et al., *Attractant resting boxes for rapid collection and surveillance of* Aedes Aegypti *(L.) inside houses.* Journal of the American Mosquito Control Association, 1997. **13**(1): p. 24-27.

486. Egger, J.R. and P.G. Coleman, *Age and clinical dengue illness.* Emerging Infectious Diseases, 2007. **13**(6): p. 924-925.

487. Eiras, A.E. and M.C. Resende, *Preliminary evaluation of the 'Dengue-MI' technology for* Aedes Aegypti *monitoring and control.* Cadernos de Saúde Pública, 2009. **25 Suppl 1**: p. S45-58.

488. Eliason, D.A., et al., *Apparent influence of the stage of blood meal digestion on the efficacy of ground applied ULV aerosols for the control of urban Culex mosquitoes. II. Laboratory evidence.* Journal of the American Mosquito Control Association, 1990. **6**(3): p. 371-375.

489. Engelthaler, D. *DENGUE RUMOR FALSE - ARIZONA, USA*. PROMED, 1996.

490. Engelthaler, D.M., et al., *The reemergence of* Aedes Aegypti *in Arizona.* Emerging Infectious Diseases, 1997. **3**(2): p. 241-242.

491. Espinoza Gomez, F., C.M. Hernandez Suarez, and R. Coll Cardenas, *[Factors that modify the larval indices of* Aedes Aegypti *in Colima, Mexico].* Revista Panamericana de Salud Pública, 2001. **10**(1): p. 6-12.

492. Espinoza-Gomez, F., C.M. Hernandez-Suarez, and R. Coll-Cardenas, *Educational campaign versus malathion spraying for the control of* Aedes Aegypti *in Colima, Mexico.* Journal of Epidemiology and Community Health, 2002. **56**(2): p. 148-152.

493. Essam Abdel, S.S., et al., *Efficacy of botanical extracts from* Callitris glaucophylla*, against* Aedes Aegypti *and* Culex annulirostris *mosquitoes.* Tropical Biomedicine, 2006. **23**(2): p. 180-185.

494. Evans, B.R. and G.A. Bevier, *Measurement of Field Populations of* Aedes Aegypti *with Ovitrap in 1968.* Mosquito News, 1969. **29**(3): p. 347-353.

495. Evans, D. *DENGUE - AUSTRALIA*. 1995 [cited 1995 22 August].

496. Evans, D. *DENGUE - AUSTRALIA* PROMED, 1995.

497. Facchinelli, L., et al., *Evaluation of a sticky trap for collecting* Aedes (Stegomyia) *adults in a dengue-endemic area in Thailand.* American Journal of Tropical Medicine and Hygiene, 2008. **78**(6): p. 904-909.

498. Facchinelli, L., et al., *Development of a novel sticky trap for container-breeding mosquitoes and evaluation of its sampling properties to monitor urban populations of* Aedes albopictus*.* Medical and Veterinary Entomology, 2007. **21**(2): p. 183-195.

499. Failloux, A.B., H. Darius, and N. Pasteur, *Genetic differentiation of* Aedes aegypti*, the vector of dengue virus in French Polynesia.* Journal of the American Mosquito Control Association, 1995. **11**(4): p. 457-462.

500. Failloux, A.B., et al., *Isoenzyme differentiation of* Aedes Aegypti *populations in French Guiana.* Medical and Veterinary Entomology, 2002. **16**(4): p. 456-460.

501. Failloux, A.B., P. Tuhiti, and Y. Sechan, *[Evaluation of larvicide susceptibility of Culicidae in French Polynesia].* Bulletin De La Societe De Pathologie Exotique, 1990. **83**(3): p. 399-405.

502. Failloux, A.B., et al., *[Genetic control of vectorial competence in* Aedes *mosquitoes].* Bulletin De La Societe De Pathologie Exotique, 1999. **92**(4): p. 266-273.

503. Fajardo, P., et al., *[Popular notions regarding "dengue" and "rompehuesos", 2 models of the disease in Colombia].* Revista Panamericana de Salud Publica, 2001. **10**(3): p. 161-168.

504. Fantinatti, E.C., et al., *[Abundance and aggregation egg of* Aedes Aegypti *L. and* Aedes albopictus *(Skuse) (Diptera: Culicidae) in the north and northwest of the State of Parana, Brazil].* Neotropical Entomology, 2007. **36**(6): p. 960-965.

505. Farrell, K.T., *An epidemic of dengue fever in Wewak.* Papua New Guinea Medical Journal 1978. **21**(2): p. 191-196.

506. Faulde, M.K., J.J. Scharninghausen, and M. Tisch, *Fire fighting truck-based emergency mosquito biolarviciding to prevent outbreaks of malaria and arboviral disease in Kabul, Afghanistan.* Journal of Pest Science, 2008. **81**(2): p. 71-77.

507. Fauran, P., M. Laille, and J.P. Moreau, *[Study on the vertical transmission of the dengue virus in the South Pacific].* Bulletin de la Societe de Pathologie Exotique, 1990. **83**(3): p. 311-316.

508. Fauran, P., M. Laille, and J.P. Moreau, *[Study on the vertical transmission of the dengue virus in the South Pacific].* Bulletin De La Societe De Pathologie Exotique, 1990. **83**(3): p. 311-316.

509. Fauran, P., G. Le Gonidec, and F. Rodhain, *[Research on viral infections in South Pacific mosquitoes under natural conditions].* Bulletin De La Société De Pathologie Exotique Et De Ses Filiales, 1984. **77**(5): p. 628-636.

510. Fauran, P. and R. Taylor, *[Monitoring of mosquito vectors at international airports of the South Pacific region].* Bulletin De La Société De Pathologie Exotique Et De Ses Filiales, 1988. **81**(1): p. 125-135.

511. Favaro, E.A., et al., *Physiological state of* Aedes (Stegomyia) aegypti *mosquitoes captured with MosquiTRAPs in Mirassol, Sao Paulo, Brazil.* Journal of Vector Ecology 2006. **31**(2): p. 285-291.

512. Favaro, E.A., et al., *Assessment of entomological indicators of* Aedes Aegypti *(L.) from adult and egg collections in Sao Paulo, Brazil.* Journal of Vector Ecology, 2008. **33**(1): p. 8-16.

513. Fay, R.W. and G.B. Craig, *Genetically Marked* Aedes Aegypti *in Studies of Field Populations.* Mosquito News, 1969. **29**(1): p. 121-127.

514. Fe, N.F., et al., *[Culicidae insect fauna from rural zone in Amazonas State with incidence of sylvatic yellow fever].* Revista da Sociedade Brasileira de Medicina Tropical, 2003. **36**(3): p. 343-348.

515. Fe, N.F., et al., *Registration of the occurrence of* Aedes albopictus *in an urban zone in Manaus, Amazonas, Brazil.* Revista de Saúde Pública, 2003. **37**(5): p. 674-675.

516. Feres, V.C., et al., *Laboratory surveillance of dengue virus in Central Brazil, 1994-2003.* Journal of Clinical Virology, 2006. **37**(3): p. 179-183.

517. Fergussonlaguna, A. and C.E. Machadoallison, *[Inbreeding Effect of Several Generations of Full-Sib Mating in* Aedes-Aegypti *(L)].* Acta Cientifica Venezolana, 1979. **30**(5): p. 507-510.

518. Fernandez, E.A., et al., *Trial of a community-based intervention to decrease infestation of* Aedes Aegypti *mosquitoes in cement washbasins in El Progreso, Honduras.* Acta Tropica, 1998. **70**(2): p. 171-183.

519. Fernandez, R., et al., *[Study of the relationship dengue-pregnancy in a group of cuban-mothers].* Revista Cubana de Medicina Tropical, 1994. **46**(2): p. 76-78.

520. Fernandez, Z. and O.P. Forattini, *[Survival of* Aedes albopictus*: physiological age and reproductive history].* Revista de Saúde Pública, 2003. **37**(3): p. 285-291.

521. Fernandez, Z., et al., *Susceptibility of urban and rural populations of* Aedes albopictus *from Sao Paulo State, Brazil, to infection by dengue-1 and -2 viruses.* Journal of Medical Entomology, 2004. **41**(5): p. 961-964.

522. Fernandez, Z., et al., *Vector competence of rural and urban strains of* Aedes (Stegomyia) albopictus *(Diptera : Culicidae) from Sao Paulo State, Brazil for IC, ID, and IF subtypes of Venezuelan equine encephalitis virus.* Journal of Medical Entomology, 2003. **40**(4): p. 522-527.

523. Ferreira, A.C. and F. Chiaravalloti Neto, *[Infestation of an urban area by* Aedes Aegypti *and relation with socioeconomic levels].* Revista de Saude Publica, 2007. **41**(6): p. 915-922.

524. Figueiredo, L.B., et al., *Dengue virus 3 genotype I associated with dengue fever and dengue hemorrhagic fever, Brazil.* Emerging Infectious Diseases, 2008. **14**(2): p. 314-316.

525. Figueiredo, L.T., S.M. Cavalcante, and M.C. Simoes, *Dengue serologic survey of schoolchildren in Rio de Janeiro, Brazil, in 1986 and 1987.* Bulletin of the Pan American Health Organization, 1990. **24**(2): p. 217-225.

526. Figueiredo, L.T., et al., *Dengue serologic survey in Ribeirao Preto, Sao Paulo, Brazil.* Bulletin of the Pan American Health Organization, 1995. **29**(1): p. 59-69.

527. Fink, T.M., et al., Aedes Aegypti *in Tucson, Arizona.* Emerging Infectious Diseases, 1998. **4**(4): p. 703-704.

528. Flisser, A., et al., *Infectious diseases in Mexico. A survey from 1995-2000.* Archives of Medical Research, 2002. **33**(4): p. 343-350.

529. Flores, A.E., et al., *Elevated alpha-esterase levels associated with permethrin tolerance in* Aedes Aegypti *(L.) from Baja California, Mexico.* Pesticide Biochemistry and Physiology, 2005. **82**(1): p. 66-78.

530. Flores-Mendoza, C., *Surveillance of* Aedes Aegypti *in Comas district, Lima, Peru.* American Journal of Tropical Medicine and Hygiene, 2006. **75**(5): p. 268.

531. Focks, D.A. and D.D. Chadee, *Pupal survey: an epidemiologically significant surveillance method for* Aedes aegypti*: an example using data from Trinidad.* American Journal of Tropical Medicine and Hygiene, 1997. **56**(2): p. 159-167.

532. Focks, D.A., S.R. Sackett, and D.L. Bailey, *Field Experiments on the Control of* Aedes-Aegypti *and Culex-Quinquefasciatus by Toxorhynchites-Rutilus Rutilus (Diptera, Culicidae).* Journal of Medical Entomology, 1982. **19**(3): p. 336-339.

533. Focks, D.A., et al., *Observations on container-breeding mosquitoes in New Orleans, Louisiana, with an estimate of the population density of* Aedes Aegypti *(L.).* American Journal of Tropical Medicine and Hygiene, 1981. **30**(6): p. 1329-1335.

534. Focks, D.A., et al., *Effect of Weekly Releases of Toxorhynchites-Amboinensis (Doleschall) on* Aedes-Aegypti *(L) (Diptera, Culicidae) in New-Orleans, Louisiana.* Journal of Economic Entomology, 1985. **78**(3): p. 622-626.

535. Focks, D.A., et al., *The Integrated Use of* Toxorhynchites-Amboinensis *and Ground-Level Ulv Insecticide Application to Suppress* Aedes-Aegypti *(Diptera, Culicidae).* Journal of Medical Entomology, 1986. **23**(5): p. 513-519.

536. Fontenille, D., *[New and unusual locations of* Aedes (Stegomyia) aegypti*, Linne 1762 (Diptera, Culicidae) in Madagascar].* Bulletin De La Société De Pathologie Exotique Et De Ses Filiales, 1986. **79**(4): p. 525-530.

537. Fontenille, D., et al., *First evidence of natural vertical transmission of yellow fever virus in* Aedes aegypti*, its epidemic vector.* Transactions of the Royal Society of Tropical Medicine and Hygiene, 1997. **91**(5): p. 533-535.

538. Fontenille, D., et al., *First evidence of natural vertical transmission of yellow fever virus in* Aedes aegypti*, its epidemic vector.* Transactions of the Royal Society of Tropical Medicine and Hygiene, 1997. **91**(5): p. 533-535.

539. Fontenille, D., et al., *[Arbovirus infections on the island of Nosy-Be; serologic and entomologic findings].* Archives de l'Institut Pasteur de Madagascar, 1988. **54**(1): p. 101-115.

540. Fontenille, D., et al., *[Arboviroses in the region of Nosy-Be, Madagascar. Serologic and entomologic data].* Bulletin De La Société De Pathologie Exotique Et De Ses Filiales, 1988. **81**(1): p. 58-70.

541. Fontenille, D. and F. Rodhain, *Biology and distribution of* Aedes albopictus *and* Aedes Aegypti *in Madagascar.* Journal of the American Mosquito Control Association, 1989. **5**(2): p. 219-225.

542. Fontenille, D., et al., *New vectors of Rift Valley fever in West Africa.* Emerging Infectious Dieseases, 1998. **4**(2): p. 289-293.

543. Fontenille, D., et al., *Short report: Rift Valley fever in western Africa: isolations from Aedes mosquitoes during an interepizootic period.* American Journal of Tropical Medicine and Hygiene, 1995. **52**(5): p. 403-404.

544. Forattini, O.P., et al., *[Adult* Aedes albopictus *and Ae. scapularis behavior (Diptera: Culicidae in Southeastern Brazil].* Revista de Saúde Pública, 2000. **34**(5): p. 461-467.

545. Forattini, O.P., et al., *[Epidemiologic significance of* Aedes albopictus *breeding places in bromeliads].* Revista de Saúde Pública, 1998. **32**(2): p. 186-188.

546. Fornells, L.A. *DENGUE/DHF UPDATE 2004 (31) [Brazil]*. 2004 [cited 2004 7 October]; Diario do Nordeste 2 Oct 2004 [in Portuguese, summarized by Mod.JW;

edited].

547. Fornells, L.A. *DENGUE/DHF UPDATE 2004 (31)*. 2004 [cited 2004 7th Oct].

548. Fornells, L.A. *DENGUE/DHF UPDATE 2005 (01)*. 2004 [cited 2005 5th Jan].

549. Fornells, L.A. *DENGUE/DHF UPDATE 2004 (31)* PROMED, 2004.

550. Fortaleza, C. *HEMORRHAGIC FEVER - BRAZIL (CEARA)*. PROMED, 1998.

551. Foster, B.E., Aedes-Albopictus *larvae collected from tree holes in southern Indiana.* Journal of the American Mosquito Control Association, 1989. **5**(1): p. 95-95.

552. Fouque, F., et al., Aedes Aegypti *in French Guiana: susceptibility to a dengue virus.* Tropical Medicine & International Health, 2001. **6**(1): p. 76-82.

553. Fox, I., Aedes-Aegypti *Reared from Dry Artificial Habitats during Drought in Puerto-Rico in 1974.* Mosquito News, 1975. **35**(2): p. 202-203.

554. Fox, I., *Evaluation of Ultralow Volume Aerial and Ground Applications of Malathion against Natural-Populations of* Aedes-Aegypti *in Puerto-Rico.* Mosquito News, 1980. **40**(2): p. 280-283.

555. Fox, I. and I.G. Bayona, *Malathion Resistant Strains of* Aedes-Aegypti *in Puerto-Rico in 1969.* Mosquito News, 1972. **32**(2): p. 157-160.

556. Fox, I. and P. Specht, *Evaluating ultra-low volume ground applications of malathion against* Aedes Aegypti *using landing counts in Puerto Rico, 1980-84.* Journal of the American Mosquito Control Association, 1988. **4**(2): p. 163-167.

557. Francy, D.B., et al., *A new arbovirus from* Aedes albopictus*, an Asian mosquito established in the United States.* Science, 1990. **250**(4988): p. 1738-1740.

558. Freier, J.E. and D.B. Francy, *A duplex cone trap for the collection of adult* Aedes albopictus*.* Journal of the American Mosquito Control Association, 1991. **7**(1): p. 73-79.

559. Fulmali, P.V., A. Walimbe, and P.V.M. Mahadev, *Spread, establishment & prevalence of dengue vector* Aedes Aegypti *(L.) in Konkan region, Maharashtra, India.* Indian Journal of Medical Research, 2008. **127**(6): p. 589-601.

560. Furlow, B.M. and W.W. Young, *Larval Surveys Compared to Ovitrap Surveys for Detecting* Aedes-Aegypti *and Aedes-Triseriatus.* Mosquito News, 1970. **30**(3): p. 468-470.

561. Gad, A.M., et al., *Host feeding of mosquitoes (Diptera: Culicidae) associated with the recurrence of Rift Valley fever in Egypt.* Journal of Medical Entomology 1999. **36**(6): p. 709-714.

562. Gama, R.A., et al., *Evaluation of the sticky MosquiTRAP for detecting* Aedes (Stegomyia) aegypti *(L.) (Diptera: Culicidae) during the dry season in Belo Horizonte, Minas Gerais, Brazil.* Neotropical Entomology, 2007. **36**(2): p. 294-302.

563. Garcia, J.J., T. Fukuda, and J.J. Becnel, *Seasonality, prevalence and pathogenicity of the gregarine* Ascogregarina taiwanensis *(Apicomplexa: Lecudinidae) in mosquitoes from Florida.* Journal of the American Mosquito Control Association, 1994. **10**(3): p. 413-418.

564. Garcia-Franco, F., et al., *Large genetic distances among* Aedes Aegypti *populations along the south Pacific coast of Mexico.* American Journal of Tropical Medicine and Hygiene, 2002. **66**(5): p. 594-598.

565. Garcia-Rejon, J., et al., *Dengue Virus-Infected* Aedes Aegypti *in the Home Environment.* American Journal of Tropical Medicine and Hygiene, 2008. **79**(6): p. 940-950.

566. Gargan, T.P., P.G. Jupp, and R.J. Novak, *Panveld oviposition sites of floodwater Aedes mosquitoes and attempts to detect transovarial transmission of Rift Valley fever virus in South Africa.* Medical and Veterinary Entomology, 1988. **2**(3): p. 231-236.

567. Gaunt, C.M., J.P. Mutebi, and L.E. Munstermann, *Biochemical taxonomy and enzyme electrophoretic profiles during development, for three morphologically similar* Aedes *species (Diptera: Culicidae) of the subgenus* Stegomyia*.* Journal of Medical Entomology, 2004. **41**(1): p. 23-32.

568. Geevarghese, G., et al., *Field Trials for Control of* Aedes-Aegypti *with Abate in Poona City and Suburbs.* Indian Journal of Medical Research, 1977. **65**(4): p. 466-473.

569. Geevarghese, G., H.N. Kaul, and V. Dhanda, *Observations on Re-Establishment of* Aedes-Aegypti *Population in Poona-City and Suburbs, Maharashtra-State, India.* Indian Journal of Medical Research, 1975. **63**(8): p. 1155-1163.

570. George, R. and G. Duraisamy, *Bleeding manifestations of dengue haemorrhagic fever in Malaysia.* Acta Tropica, 1981. **38**(1): p. 71-78.

571. Gerade, B.B., et al., *Field validation of* Aedes Aegypti *(Diptera: Culicidae) age estimation by analysis of cuticular hydrocarbons.* Journal of Medical Entomology, 2004. **41**(2): p. 231-238.

572. Gerhardt, R.R., et al., *First isolation of La Crosse virus from naturally infected* Aedes albopictus*.* Emerging Infectious Diseases, 2001. **7**(5): p. 807-811.

573. Germain, M., et al., *Yellow fever in the Gambia, 1978--1979: entomological aspects and epidemiological correlations.* American Journal of Tropical Medicine and Hygiene, 1980. **29**(5): p. 929-940.

574. Germain, M., et al., *Yellow fever in the Gambia, 1978--1979: entomological aspects and epidemiological correlations.* American Journal of Tropical Medicine and Hygiene, 1980. **29**(5): p. 929-940.

575. Getis, A., et al., *Characteristics of the spatial pattern of the dengue vector,* Aedes aegypti*, in Iquitos, Peru.* American Journal of Tropical Medicine and Hygiene, 2003. **69**(5): p. 494-505.

576. Gianella, A. *DENGUE SURVEILLANCE, SANTA CRUZ - BOLIVIA*. 1996 [cited 1996 10 September].

577. Gianella, A. *YELLOW FEVER - BOLIVIA (SANTA CRUZ) (06)*. PROMED, 1999.

578. Gianella, A., et al., *[Epidemic outbreak of dengue virus 2/Jamaica genotype in Bolivia].* Salud Publica de Mexico 1998. **40**(6): p. 469-473.

579. Gionar, Y.R., S. Atmosoedjono, and M.J. Bangs, *Mesocyclops brevisetosus (Cyclopoida: Cyclopoidae) as a potential biological control agent against mosquito larvae in Indonesia.* Journal of the American Mosquito Control Association, 2006. **22**(3): p. 437-443.

580. Gionar, Y.R., et al., *Use of a funnel trap for collecting immature* Aedes Aegypti *and copepods from deep wells in Yogyakarta, Indonesia.* Journal of the American Mosquito Control Association, 1999. **15**(4): p. 576-580.

581. Giraldo-Calderon, G.I., et al., *[Evaluation of the triflumuron and the mixture of Bacillus thuringiensis plus Bacillus sphaericus for control of the immature stages of* Aedes Aegypti *and* Culex quinquefasciatus *(Diptera: Culicidae) in catch basins].* Biomedica, 2008. **28**(2): p. 224-233.

582. Goettel, M.S., M.K. Toohey, and J.S. Pillai, *The urban mosquitoes of Suva, Fiji: seasonal incidence and evaluation of environmental sanitation and ULV spraying for their control.* Journal of Tropical Medicine and Hygiene, 1980. **83**(4): p. 165-171.

583. Gokhale, M.D., P.G. Jacob, and D.T. Mourya, *Dengue virus and insecticide susceptibility status of* Aedes Aegypti *mosquitoes from Belagola village, Mandya District, Karnataka state: during and post-epidemic investigations.* Journal of Communicable Diseases, 2000. **32**(4): p. 247-253.

584. Gomes, A.C., et al., *Host-feeding patterns of potential human disease vectors in the Paraiba Valley Region, State of Sao Paulo, Brazil.* Journal of Vector Ecology, 2003. **28**(1): p. 74-78.

585. Gomes, A.D., et al., *Anthropophilic activity of* Aedes Aegypti *and of* Aedes albopictus *in area under control and surveillance.* Revista de Saude Publica, 2005. **39**(2): p. 206-210.

586. Gomes, A.D., et al., *Duration of Larval and Pupal Development Stages of* Aedes-Albopictus *in Natural and Artificial Containers.* Revista de Saude Publica, 1995. **29**(1): p. 15-19.

587. Gomes Ade, C., et al., *[*Aedes albopictus *in rural zone of Brazil and its implication in the wild yellow fever transmission].* Revista de Saude Publica, 1999. **33**(1): p. 95-97.

588. Goncalves Neto, V.S. and J.M. Rebelo, *[Epidemiological characteristics of dengue in the Municipality of Sao Luis, Maranhao, Brazil, 1997-2002].* Cadernos de Saude Publica, 2004. **20**(5): p. 1424-1431.

589. Gonzalez, J. *DENGUE/DHF UPDATE 2005 (39)* PROMED, 2005.

590. Gonzalez, L.A., M.O. Araujo, and A.B. Azuje, *[Evaluation of Temephos 50% CE on populations of* Aedes Aegypti *(Diptera: Culicidae) in Trujillo, Venezuela].* Revista Colombiana De Entomologia, 2008. **34**(2): p. 188-191.

591. Gora, D., et al., *The potential role of rodents in the enzootic cycle of Rift Valley fever virus in Senegal.* Microbes and Infection, 2000. **2**(4): p. 343-346.

592. Gorrochotegui-Escalante, N., et al., *Breeding structure of* Aedes Aegypti *populations in Mexico varies by region.* American Journal of Tropical Medicine and Hygiene, 2002. **66**(2): p. 213-222.

593. Gorrochotegui-Escalante, N., et al., *Association mapping of segregating sites in the early trypsin gene and susceptibility to dengue-2 virus in the mosquito Aedes aegypti.* Insect Biochemistry and Molecular Biology, 2005. **35**(7): p. 771-788.

594. Gorrochotegui-Escalante, N., et al., *Genetic isolation by distance among* Aedes Aegypti *populations along the northeastern coast of Mexico.* American Journal of Tropical Medicine and Hygiene, 2000. **62**(2): p. 200-209.

595. Gottfried, K.L., et al., *Temporal abundance, parity, survival rates, and arbovirus isolation of field-collected containerinhabiting mosquitoes in eastern Tennessee.* Journal of the American Mosquito Control Association, 2002. **18**(3): p. 164-172.

596. Gouck, H.K., *Host Preferences of Various Strains of* Aedes-Aegypti *and Aedes-Simpsoni as Determined by an Olfactometer.* Bulletin of the World Health Organization, 1972. **47**(5): p. 680-683.

597. Gould, D.J., et al., *Dengue Control on an Island in Gulf of Thailand .1. Results of an* Aedes-Aegypti *Control Program.* American Journal of Tropical Medicine and Hygiene, 1971. **20**(5): p. 705-714.

598. Grill, C.P. and S.A. Juliano, *Predicting species interactions based on behaviour: Predation and competition in container-dwelling mosquitoes.* Journal of Animal Ecology, 1996. **65**(1): p. 63-76.

599. Grimstad, P.R., et al., *Recently introduced* Aedes albopictus *in the United States: potential vector of La Crosse virus (Bunyaviridae: California serogroup).* Journal of the American Mosquito Control Association, 1989. **5**(3): p. 422-427.

600. Grover, K.K. and H.V. Agarwal, *Chemo-Sterilization of* Aedes-Aegypti *(L) .3. Control of a Simulated Natural-Population in a Field Cage by Releases of Thiotepa-Sterilized Males.* Indian Journal of Experimental Biology, 1979. **17**(2): p. 139-143.

601. Guard, R.W., N.D. Stallman, and M.A. Wiemers, *Dengue in the northern region of Queensland, 1981-1982.* Medical Journal of Australia 1984. **140**(13): p. 765-769.

602. Gubler, D.J., et al., *Variation in susceptibility to oral infection with dengue viruses among geographic strains of* Aedes aegypti*.* American Journal of Tropical Medicine and Hygiene, 1979. **28**(6): p. 1045-1052.

603. Gubler, D.J. and L. Rosen, *Variation among geographic strains of* Aedes albopictus *in susceptibility to infection with dengue viruses.* American Journal of Tropical Medicine and Hygiene, 1976. **25**(2): p. 318-325.

604. Gubler, D.J., et al., *Epidemic dengue 3 in central Java, associated with low viremia in man.* American Journal of Tropical Medicine and Hygiene, 1981. **30**(5): p. 1094-1099.

605. Guerra, H.L., et al., *[Effectiveness of the yellow fever vaccine 17D: an epidemiologic evaluation in health services].* Revista Panamericana de Salud Publica, 1997. **2**(2): p. 115-120.

606. Gunther, J., et al., *Evidence of vertical transmission of dengue virus in two endemic localities in the state of Oaxaca, Mexico.* Intervirology, 2007. **50**(5): p. 347-352.

607. Gupta, D.K., et al., *Intradomestic mosquito breeding sources and their management.* Indian Journal of Malariology, 1992. **29**(1): p. 41-46.

608. Gurtler, R.E., F.M. Garelli, and H.D. Coto, *Effects of a Five-Year Citywide Intervention Program To Control* Aedes Aegypti *and Prevent Dengue Outbreaks in Northern Argentina.* Plos Neglected Tropical Diseases, 2009. **3**(4): p. -.

609. Gusmao, D.S., et al., *Derris (Lonchocarpus) urucu (Leguminosae) extract modifies the peritrophic matrix structure of* Aedes Aegypti *(Diptera:Culicidae).* Memorias Do Instituto Oswaldo Cruz, 2002. **97**(3): p. 371-375.

610. Guzman, M.G., et al., *[Final characterization of and lessons learned from the dengue 3 epidemic in Cuba, 2001-2002].* Revista Panamericana De Salud Publica-Pan American Journal of Public Health, 2006. **19**(4): p. 282-289.

611. Guzman, M.G., et al., *[The estimation of the economic damages caused as a consequence of the epidemic of hemorrhagic dengue in Cuba in 1981].* Revista Cubana de Medicina Tropical, 1992. **44**(1): p. 13-17.

612. Guzman Tirado, M.G., et al., *[Retrospective sero-epidemiologic survey on dengue virus in the municipalities of Cienfuegos and Palmira].* Revista Cubana de Medicina Tropical, 1989. **41**(3): p. 321-332.

613. Gwadz, R.W. and G.B. Craig, *Sexual Receptivity in Female Aedes Aegypti.* Mosquito News, 1968. **28**(4): p. 586-593.

614. Ha, D.Q. *DENGUE - VIETNAM (02)*. 1996 [cited 1996 6 November].

615. Haber, W.A. and C.G. Moore, Aedes-Aegypti *in Puerto-Rican Rain-Forest - Results of a One-Year Survey.* Mosquito News, 1973. **33**(4): p. 576-578.

616. Hadad, M. *DENGUE/DHF UPDATE 2004 (24) [Afghanistan/ Vietnam/ Philippines/ Saudi Arabia]*. 2004 [cited 2004 7 August]; Globe and Mail 4 Aug 2004 [edited].

617. Haddad, N., et al., *Presence of* Aedes albopictus *in Lebanon and Syria.* Journal of the American Mosquito Control Association, 2007. **23**(2): p. 226-228.

618. Hafkin, B., et al., *Reintroduction of dengue fever into the continental United States. I. Dengue surveillance in Texas, 1980.* American Journal of Tropical Medicine and Hygiene, 1982. **31**(6): p. 1222-1228.

619. Hamdan, H., et al., *Insecticide resistance development in Culex quinquefasciatus (Say),* Aedes Aegypti *(L.) and* Aedes albopictus *(Skuse) larvae against malathion, permethrin and temephos.* Tropical Biomedicine, 2005. **22**(1): p. 45-52.

620. Hammond, S.N., et al., *Characterization of* Aedes Aegypti *(Diptera : Culcidae) production sites in urban Nicaragua.* Journal of Medical Entomology, 2007. **44**(5): p. 851-860.

621. Hanna, J.N., et al., *Two contiguous outbreaks of dengue type 2 in north Queensland.* Medical Journal of Australia, 1998. **168**(5): p. 221-225.

622. Hanna, J.N., et al., *An epidemic of dengue 3 in far north Queensland, 1997-1999.* Medical Journal of Australia 2001. **174**(4): p. 178-182.

623. Hanna, J.N., et al., *An epidemic of dengue 3 in far north Queensland, 1997-1999.* Medical Journal of Australia, 2001. **174**(4): p. 178-182.

624. Hanna, J.N., et al., *Multiple outbreaks of dengue serotype 2 in north Queensland, 2003/04.* Australian and New Zealand Journal of Public Health, 2006. **30**(3): p. 220-225.

625. Hanson, S.M. and G.B. Craig, Jr., *Cold acclimation, diapause, and geographic origin affect cold hardiness in eggs of* Aedes albopictus *(Diptera: Culicidae).* Journal of Medical Entomology, 1994. **31**(2): p. 192-201.

626. Hanson, S.M. and G.B. Craig, Jr., *Relationship between cold hardiness and supercooling point in* Aedes albopictus *eggs.* Journal of the American Mosquito Control Association, 1995. **11**(1): p. 35-38.

627. Hanson, S.M. and G.B. Craig, Jr., Aedes albopictus *(Diptera: Culicidae) eggs: field survivorship during northern Indiana winters.* Journal of Medical Entomology, 1995. **32**(5): p. 599-604.

628. Hanson, S.M., et al., *Reducing the overwintering ability of* Aedes albopictus *by male release.* Journal of the American Mosquito Control Association, 1993. **9**(1): p. 78-83.

629. Haq, S., et al., *Field evaluation of biolarvicides in Surat city, India.* Journal of Vector Borne Diseases, 2004. **41**(3-4): p. 61-66.

630. Harley, D., et al., *Mosquito isolates of Ross River virus from Cairns, Queensland, Australia.* American Journal of Tropical Medicine and Hygiene, 2000. **62**(5): p. 561-565.

631. Harrington, L.C., et al., *Analysis of survival of young and old* Aedes Aegypti *(Diptera : Culicidae) from Puerto Rico and Thailand.* Journal of Medical Entomology, 2001. **38**(4): p. 537-547.

632. Harrington, L.C. and J.D. Edman, *Indirect evidence against delayed "skip-oviposition" behavior by* Aedes Aegypti *(Diptera: Culicidae) in Thailand.* Journal of Medical Entomology, 2001. **38**(5): p. 641-645.

633. Harrington, L.C., et al., *Influence of container size, location, and time of day on oviposition patterns of the dengue vector,* Aedes aegypti*, in Thailand.* Vector Borne and Zoonotic Diseases, 2008. **8**(3): p. 415-423.

634. Harrington, L.C., et al., *Dispersal of the dengue vector* Aedes Aegypti *within and between rural communities.* American Journal of Tropical Medicine and Hygiene, 2005. **72**(2): p. 209-220.

635. Harrington, L.C., et al., *Age-dependent survival of the dengue vector* Aedes Aegypti *(Diptera : Culicidae) demonstrated by simultaneous release-recapture of different age cohorts.* Journal of Medical Entomology, 2008. **45**(2): p. 307-313.

636. Harrington, M.A., et al., *Genetic-Variation in an Urban-Population of* Aedes-Aegypti *(Diptera, Culicidae).* Journal of Medical Entomology, 1984. **21**(6): p. 706-710.

637. Harrison, B. *DENGUE VIRUSES, TRANSMISSION BY* AEDES ALBOPICTUS *(08) [Thailand]*. 1998 [cited 1998 17 May].

638. Harrison, B.A., et al., *An Efficient Floating Larval Trap for Sampling* Aedes-Aegypti *Populations (Diptera, Culicidae).* Journal of Medical Entomology, 1982. **19**(6): p. 722-727.

639. Harrison, B.A., et al., *Mosquitoes (Diptera : Culicidae) collected near the Great Dismal Swamp: New state records, notes on certain species, and a revised checklist for Virginia.* Proceedings of the Entomological Society of Washington, 2002. **104**(3): p. 655-662.

640. Hartberg, W.K., *Observations on Mating Behavior of* Aedes-Aegypti *in Nature.* Bulletin of the World Health Organization, 1971. **45**(6): p. 847-850.

641. Hartberg, W.K., *Palp-Extended, a Sex-Linked and Sex-Limited Mutant of Aedes-Aegypti.* Mosquito News, 1975. **35**(1): p. 34-40.

642. Hartberg, W.K. and G.B. Craig, *Gene-Controlled Morphological Differences in Male Genitalia of* Aedes-Aegypti *and* Aedes-Mascarensis *(Diptera-Culicidae).* Mosquito News, 1973. **33**(2): p. 206-214.

643. Hashim, N.A., et al., *Life tables study of immature* Aedes albopictus *(Skuse) (Diptera : Culicidae) during the wet and dry seasons in Penang, Malaysia.* Southeast Asian Journal of Tropical Medicine and Public Health, 2008. **39**(1): p. 39-47.

644. Hauserma.W, R.W. Fay, and C.S. Hacker, *Dispersal of Genetically Marked Female* Aedes-Aegypti *in Mississippi.* Mosquito News, 1971. **31**(1): p. 37-51.

645. Hawley, W.A., et al., *Overwintering survival of* Aedes albopictus *(Diptera: Culicidae) eggs in Indiana.* Journal of Medical Entomology, 1989. **26**(2): p. 122-129.

646. Hayes, G.R. and Haverfie.Le, *Distribution and Density of* Aedes-Aegypti *(L) and Lankesteria-Culicis (Ross) in Louisiana and Adjoining Areas.* Mosquito News, 1971. **31**(1): p. 28-32.

647. Hayes, J.M., et al., *Risk factors for infection during a severe dengue outbreak in El Salvador in 2000.* American Journal of Tropical Medicine and Hygiene, 2003. **69**(6): p. 629-633.

648. Heard, P.B., et al., *Transmission of a newly recognized virus (Bunyaviridae, Bunyavirus) isolated from* Aedes albopictus *(Diptera: Culicidae) in Potosi, Missouri.* Journal of Medical Entomology, 1991. **28**(5): p. 601-605.

649. Hecker, H. and R. Brun, *Morphometric differences in midgut epithelial cells between strains of female* Aedes Aegypti *(L.) (Insecta, Diptera).* Cell Tissue Research, 1975. **159**(1): p. 91-99.

650. Hemingway, J., et al., *Mechanisms of Insecticide Resistance in* Aedes-Aegypti *(L) (Diptera, Culicidae) from Puerto-Rico.* Bulletin of Entomological Research, 1989. **79**(1): p. 123-130.

651. Herbert, E.W. and P.V. Perkins, *Comparative Tests of 5 Insecticides against* Aedes-Albopictus *Larvae from South-Vietnam.* Mosquito News, 1973. **33**(1): p. 76-78.

652. Herrera, F., et al., *Population genetic structure of the dengue mosquito* Aedes Aegypti *in Venezuela.* Memorias Do Instituto Oswaldo Cruz, 2006. **101**(6): p. 625-633.

653. Herrera-Basto, E., et al., *First reported outbreak of classical dengue fever at 1,700 meters above sea level in Guerrero State, Mexico, June 1988.* American Journal of Tropical Medicine and Hygiene, 1992. **46**(6): p. 649-653.

654. Heukelbach, J., et al., *Risk factors associated with an outbreak of dengue fever in a favela in Fortaleza, north-east Brazil.* Tropical Medicine & International Health, 2001. **6**(8): p. 635-642.

655. Hickey, W.A. and G.B. Craig, *Distortion of Sex Ratio in Populations of Aedes Aegypti.* Canadian Journal of Genetics and Cytology, 1966. **8**(2): p. 260-278.

656. Higgs, S., et al., *Growth characteristics of ChimeriVax-Den vaccine viruses in* Aedes Aegypti *and* Aedes albopictus *from Thailand.* American Journal of Tropical Medicine and Hygiene, 2006. **75**(5): p. 986-993.

657. Hill, L.A., et al., *Rapid Identification of* Aedes albopictus*,* Aedes scutellaris*, and* Aedes Aegypti *Life Stages Using Real-time Polymerase Chain Reaction Assays.* American Journal of Tropical Medicine and Hygiene, 2008. **79**(6): p. 866-875.

658. Hills, S.L., et al., *A focal, rapidly-controlled outbreak of dengue fever in two suburbs in Townsville, north Queensland, 2001.* Communicable Diseases Intelligence, 2002. **26**(4): p. 596-600.

659. Ho, B.C., K.L. Chan, and Y.C. Chan, Aedes-Aegypti *(L.) and* Aedes-Albopictus *(Skuse) in Singapore City .3. Population Fluctuations.* Bulletin of the World Health Organization, 1971. **44**(5): p. 635-641.

660. Ho, B.C., A. Ewert, and L.M. Chew, *Interspecific competition among* Aedes aegypti*,* Ae. albopictus*, and* Ae. triseriatus *(Diptera: Culicidae): larval development in mixed cultures.* Journal of Medical Entomology, 1989. **26**(6): p. 615-623.

661. Hobbs, J.H., E.A. Hughes, and B.H. Eichold, *Replacement of* Aedes-Aegypti *by* Aedes-Albopictus *in Mobile, Alabama.* Journal of the American Mosquito Control Association, 1991. **7**(3): p. 488-489.

662. Hoeck, P.A., et al., *Population and parity levels of* Aedes Aegypti *collected in Tucson.* Journal of Vector Ecology

, 2003. **28**(1): p. 65-73.

663. Hoel, D.F., et al., *Evaluation of carbon dioxide, 1-octen-3-ol, and lactic acid as baits in Mosquito Magnet (TM) Pro traps for* Aedes albopictus *in North Central Florida.* Journal of the American Mosquito Control Association, 2007. **23**(1): p. 11-17.

664. Holick, J., et al., *Discovery of* Aedes albopictus *infected with West Nile virus in southeastern Pennsylvania.* Journal of the American Mosquito Control Association, 2002. **18**(2): p. 131-131.

665. Honorio, N.A., et al., *Preliminary data on the performance of* Aedes Aegypti *and* Aedes albopictus *immatures developing in water-filled tires in Rio de Janeiro.* Memorias Do Instituto Oswaldo Cruz, 2006. **101**(2): p. 225-228.

666. Honorio, N.A., et al., *The spatial distribution of* Aedes Aegypti *and* Aedes albopictus *in a transition zone, Rio de Janeiro, Brazil.* Cadernos de Saúde Pública, 2009. **25**(6): p. 1203-1214.

667. Honorio, N.A., et al., *Occurrence of Toxorhynchites guadeloupensis (Dyar & knab) in oviposition trap of* Aedes Aegypti *(L.) (Diptera : Culicidae).* Neotropical Entomology, 2007. **36**(5): p. 809-811.

668. Honorio, N.A. and R. Lourenco-De-Oliveira, *[Frequency of* Aedes Aegypti *and* Aedes albopictus *larvae and pupae in traps, Brazil].* Revista de Saúde Pública, 2001. **35**(4): p. 385-391.

669. Honorio, N.A., et al., *Dispersal of* Aedes Aegypti *and* Aedes albopictus *(Diptera : Culicidae) in an urban endemic dengue area in the State of Rio de Janeiro, Brazil.* Memorias Do Instituto Oswaldo Cruz, 2003. **98**(2): p. 191-198.

670. Hooper, G.H.S., *Susceptibility to Insecticides of Populations of* Aedes Aegypti *from Queensland Australia.* Annals of Tropical Medicine and Parasitology, 1967. **61**(4): p. 451-457.

671. Hopp, M. *PRO/EDR> Yellow fever - Senegal (05)*. PROMED, 2002.

672. Hopp, M. *DENGUE/DHF UPDATE 2007 (17)*. PROMED, 2007.

673. Hopp, M., M. Marshall, and J. Dudley *Dengue/DHF update 2007 (05)* PROMED, 2007.

674. Hopp, M., M. Marshall, and J. Dudley *Dengue/DHF update 2007 (05)* PROMED, 2007.

675. Hribar, L.J., et al., *Survey of container-breeding mosquitoes from the Florida Keys, Monroe County, Florida.* Journal of the American Mosquito Control Association, 2001. **17**(4): p. 245-248.

676. Hribar, L.J., et al., *Mosquito larvae (Culicidae) and other Diptera associated with containers, storm drains, and sewage treatment plants in the Florida Keys, Monroe County, Florida.* Florida Entomologist, 2004. **87**(2): p. 199-203.

677. Huang, Z.H. and J.F. Wang, *[Cloning and sequencing of cytochrome c oxidase II(COII) gene of three species of mosquitoes].* Chinese Journal of Parasitology & Parasitic Diseases - Zhongguo Ji Sheng Chong Xue Yu Ji Sheng Chong Bing Za Zhi, 2001. **19**(2): p. 90-92.

678. Huber, K., et al., Aedes Aegypti *in Senegal: Genetic diversity and genetic structure of domestic and sylvatic populations.* American Journal of Tropical Medicine and Hygiene, 2008. **79**(2): p. 218-229.

679. Huber, K., et al., *Human transportation influences* Aedes Aegypti *gene flow in Southeast Asia.* Acta Tropica, 2004. **90**(1): p. 23-29.

680. Huber, K., et al., *Genetic differentiation of the dengue vector,* Aedes Aegypti *(Ho Chi Minh City, Vietnam) using microsatellite markers.* Molecular Ecology, 2002. **11**(9): p. 1629-1635.

681. Huber, K., et al., *[*Aedes Aegypti *in Vietnam: Ecology, genetic structure, vectorial competence and resistance to insecticides.].* Annales De La Societe Entomologique De France, 2000. **36**(2): p. 109-120.

682. Huber, K., et al., Aedes Aegypti *in south Vietnam: ecology, genetic structure, vectorial competence and resistance to insecticides.* Southeast Asian Journal of Tropical Medicine and Public Health, 2003. **34**(1): p. 81-86.

683. Huber, K., et al., *Temporal genetic variation in* Aedes Aegypti *populations in Ho Chi Minh City (Vietnam).* Heredity, 2002. **89**(1): p. 7-14.

684. Huber, K., et al., *[*Aedes aegypti*, vector of the dengue virus: spatio-temporal structure of its genetic variation].* Bulletin de l Academie Nationale de Medecine 2002. **186**(7): p. 1237-1248; discussion 1248-1250.

685. Huber, K., et al., *[*Aedes aegypti*, vector of the dengue virus: spatio-temporal structure of its genetic variation].* Bulletin de l'Académie Nationale de Médecine, 2002. **186**(7): p. 1237-1248; discussion 1248-1250.

686. Hull, B., et al., *Natural transovarial transmission of dengue 4 virus in* Aedes Aegypti *in Trinidad.* American Journal of Tropical Medicine and Hygiene, 1984. **33**(6): p. 1248-1250.

687. Hull, B., et al., *Natural transovarial transmission of dengue 4 virus in* Aedes Aegypti *in Trinidad.* American Journal of Tropical Medicine and Hygiene, 1984. **33**(6): p. 1248-1250.

688. Husbands, B.S. *DENGUE/DHF - PHILIPPINES (LUZON)*. 1999 [cited 1999 31 July]; Philippine Daily Inquirer [edited].

689. Hutamai, S., et al., *A survey of dengue viral infection in* Aedes Aegypti *and* Aedes albopictus *from re-epidemic areas in the north of Thailand using nucleic acid sequence based amplification assay.* Southeast Asian Journal of Tropical Medicine and Public Health, 2007. **38**(3): p. 448-454.

690. Ibanez-Bernal, S., et al., *First record in America of* Aedes albopictus *naturally infected with dengue virus during the 1995 outbreak at Reynosa, Mexico.* Medical and Veterinary Entomology, 1997. **11**(4): p. 305-309.

691. Ibanez-Bernal, S. and C. Martinez-Campos, Aedes albopictus *in Mexico.* Journal of the American Mosquito Control Association, 1994. **10**(2 Pt 1): p. 231-232.

692. Ilkal, M.A., et al., *Entomological investigations during outbreaks of dengue fever in certain villages in Maharashtra state.* Indian Journal of Medical Research, 1991. **93**: p. 174-178.

693. Ilkal, M.A., et al., *Entomological investigations during outbreaks of dengue fever in certain villages in Maharashtra state.* Indian Journal of Medical Research, 1991. **93**: p. 174-178.

694. Impoinvil, D.E., et al., *Abundance of immature* Anopheles *and culicines (Diptera : Culicidae) in different water body types in the urban environment of Malindi, Kenya.* Journal of Vector Ecology, 2008. **33**(1): p. 107-116.

695. Inwang, E.E., M.A.Q. Khan, and A.W.A. Brown, *DDT-Resistance in West African and Asian Strains of* Aedes Aegypti *(L).* Bulletin of the World Health Organization, 1967. **36**(3): p. 409-421.

696. Irving-Bell, R.J., et al., *Septic tank mosquitoes: competition between species in central Nigeria.* Medical and Veterinary Entomology, 1987. **1**(3): p. 243-250.

697. Ishak, H., et al., *Breeding habitats of* Aedes Aegypti *(L) and* Aedes. albopictus *(Skuse) in villages of Barru, South Sulawesi, Indonesia.* Southeast Asian Journal of Tropical Medicine and Public Health, 1997. **28**(4): p. 844-850.

698. Itoh, T., et al., *Utilization of bloodfed females of* Aedes Aegypti *as a vehicle for the transfer of the insect growth regulator pyriproxyfen to larval habitats.* Journal of the American Mosquito Control Association, 1994. **10**(3): p. 344-347.

699. Jakob, W.L. and G.A. Bevier, *Application of Ovitraps in Us* Aedes Aegypti *Eradication Program.* Mosquito News, 1969. **29**(1): p. 55-62.

700. Jakob, W.L. and G.A. Bevier, *Evaluation of Ovitraps in Us* Aedes-Aegypti *Eradication Program.* Mosquito News, 1969. **29**(4): p. 650-653.

701. Jakob, W.L., R.W. Fay, and D.P. Wilton, *Field Trails of an Amine Ovicide against* Aedes-Aegypti *(L).* Mosquito News, 1970. **30**(2): p. 191-194.

702. Jardina, B.J., *The eradication of* Aedes albopictus *in Indianapolis, Indiana.* Journal of the American Mosquito Control Association, 1990. **6**(2): p. 310-311.

703. Jatanase.S, *Report on a Pilot Study of* Aedes Aegypti *Control in Bangkok.* Bulletin of the World Health Organization, 1966. **35**(1): p. 91-92.

704. Jatanasen, S., *Environmental Manipulation and Health Education in* Aedes Aegypti *Control in Thailand.* Bulletin of the World Health Organization, 1967. **36**(4): p. 636-638.

705. Jennings, C.D., et al., Aedes Aegypti *control in the Lao People's Democratic Republic, with reference to copepods.* American Journal of Tropical Medicine and Hygiene, 1995. **53**(4): p. 324-330.

706. Jensen, T., et al., *Comparison of bi-directional Fay, omni-directional, CDC, and duplex cone traps for sampling adult* Aedes albopictus *and* Aedes Aegypti *in north Florida.* Journal of the American Mosquito Control Association, 1994. **10**(1): p. 74-78.

707. Jinfu, W. and H. Chaohui, *Molecular divergence of the mitochondrial cytochrome oxidase II gene in three mosquitoes.* Journal of the American Mosquito Control Association, 2002. **18**(4): p. 301-306.

708. Jirakanjanakit, N., et al., *Insecticide susceptible/resistance status in* Aedes (Stegomyia) aegypti *and* Aedes (Stegomyia) albopictus *(Diptera : Culicidae) in Thailand during 2003-2005.* Journal of Economic Entomology, 2007. **100**(2): p. 545-550.

709. Jirakanjanakit, N., et al., *Trend of temephos resistance in* Aedes (Stegomyia) *mosquitoes in Thailand during 2003-2005.* Environmental Entomology, 2007. **36**(3): p. 506-511.

710. Johnson, B.W., et al., *Vector competence of Brazilian* Aedes Aegypti *and Ae. albopictus for a Brazilian yellow fever virus isolate.* Transactions of the Royal Society of Tropical Medicine and Hygiene, 2002. **96**(6): p. 611-613.

711. Joshi, P.T., A.P. Pandya, and J.K. Anjan, *Epidemiological and entomological investigation in dengue outbreak area of Ahmedabad district.* Journal of Communicable Diseases, 2000. **32**(1): p. 22-27.

712. Joshi, P.T., A.P. Pandya, and J.K. Anjan, *Epidemiological and entomological investigation in dengue outbreak area of Ahmedabad district.* Journal of Communicable Diseases, 2000. **32**(1): p. 22-27.

713. Joshi, V., et al., *Entomological studies in a dengue endemic area, Jalore, Rajasthan.* Indian Journal of Medical Research, 1996. **104**: p. 161-165.

714. Joshi, V., D.T. Mourya, and R.C. Sharma, *Persistence of dengue-3 virus through transovarial transmission passage in successive generations of* Aedes Aegypti *mosquitoes.* American Journal of Tropical Medicine and Hygiene, 2002. **67**(2): p. 158-161.

715. Joshi, V., et al., *Importance of socioeconomic status and tree holes in distribution of* Aedes *mosquitoes (Diptera : Culicidae) in Jodhpur, Rajasthan, India.* Journal of Medical Entomology, 2006. **43**(2): p. 330-336.

716. Jousset, F.X., et al., *A parvo-like virus persistently infecting a C6/36 clone of* Aedes albopictus *mosquito cell line and pathogenic for* Aedes Aegypti *larvae.* Virus Research, 1993. **29**(2): p. 99-114.

717. Jueco, N.L. and B.D. Cabrera, *Ecology and Biology of* Aedes-Aegypti*,* Aedes-Albopictus*, and* Culex Fatigans *- Breeding Distance and Oviposition Preference.* Kalikasan-the Philippine Journal of Biology, 1976. **5**(3): p. 301-308.

718. Juliano, S.A., *Species introduction and replacement among mosquitoes: Interspecific resource competition or apparent competition?* Ecology, 1998. **79**(1): p. 255-268.

719. Juliano, S.A., L.P. Lounibos, and G.F. O'Meara, *A field test for competitive effects of* Aedes albopictus *on* A-aegypti *in South Florida: differences between sites of coexistence and exclusion?* Oecologia, 2004. **139**(4): p. 583-593.

720. Juliano, S.A., et al., *Desiccation and thermal tolerance of eggs and the coexistence of competing mosquitoes.* Oecologia, 2002. **130**(3): p. 458-469.

721. Jumali, et al., *Epidemic dengue hemorrhagic fever in rural Indonesia. III. Entomological studies.* American Journal of Tropical Medicine and Hygiene, 1979. **28**(4): p. 717-724.

722. Jumali, S., et al., *Epidemic dengue hemorrhagic fever in rural Indonesia. III. Entomological studies.* American Journal of Tropical Medicine and Hygiene, 1979. **28**(4): p. 717-724.

723. Jupp, P.G. and A. Kemp, Aedes albopictus *and other mosquitoes imported in tires into Durban, South Africa.* Journal of the American Mosquito Control Association, 1992. **8**(3): p. 321-322.

724. Jupp, P.G. and A. Kemp, *The potential for dengue in South Africa: vector competence tests with dengue 1 and 2 viruses and 6 mosquito species.* Transactions of the Royal Society of Tropical Medicine and Hygiene, 1993. **87**(6): p. 639-643.

725. Jupp, P.G. and A. Kemp, *Laboratory vector competence experiments with yellow fever virus and five South African mosquito species including* Aedes aegypti*.* Transactions of the Royal Society of Tropical Medicine and Hygiene, 2002. **96**(5): p. 493-498.

726. Jupp, P.G., et al., *The 2000 epidemic of Rift Valley fever in Saudi Arabia: mosquito vector studies.* Medical and Veterinary Entomology, 2002. **16**(3): p. 245-252.

727. Jupp, P.G. and B.M. McIntosh, Aedes furcifer *and other mosquitoes as vectors of chikungunya virus at Mica, northeastern Transvaal, South Africa.* Journal of the American Mosquito Control Association, 1990. **6**(3): p. 415-420.

728. Jupp, P.G. and J.I. Phillips, *An electron microscopical study of Rift Valley fever and Sindbis viral infection in mosquito salivary glands (Diptera : Culicidae).* African Entomology, 1998. **6**(1): p. 75-81.

729. Kabilan, L., et al., *Field- and laboratory-based active dengue surveillance in Chennai, Tamil Nadu, India: observations before and during the 2001 dengue epidemic.* American Journal of Infection Control 2004. **32**(7): p. 391-396.

730. Kabilan, L., et al., *Field- and laboratory-based active dengue surveillance in Chennai, Tamil Nadu, India: observations before and during the 2001 dengue epidemic.* American Journal of Infection Control, 2004. **32**(7): p. 391-396.

731. Kalyanasundaram, M., et al., *Efficacy of two organophosphorus insecticides, Reldan & Dursban against the larvae of* Culex quinquefasciatus*.* Indian Journal of Medical Research, 2003. **117**: p. 25-29.

732. Kambhampati, S., W.C. Black, and K.S. Rai, *Geographic Origin of the United-States and Brazilian* Aedes-Albopictus *Inferred from Allozyme Analysis.* Heredity, 1991. **67**(Part 1): p. 85-94.

733. Kambhampati, S., et al., *Temporal variation in genetic structure of a colonising species:* Aedes albopictus *in the United States.* Heredity, 1990. **64 ( Pt 2)**: p. 281-287.

734. Kambhampati, S. and K.S. Rai, *Temporal variation in the ribosomal DNA nontranscribed spacer of* Aedes albopictus *(Diptera: Culicidae).* Genome, 1991. **34**(2): p. 293-297.

735. Kambhampati, S. and K.S. Rai, *Mitochondrial DNA variation within and among populations of the mosquito* Aedes albopictus*.* Genome, 1991. **34**(2): p. 288-292.

736. Kambhampati, S. and K.S. Rai, *Patterns of Morphometric and Allozyme Variation in* Aedes-Albopictus*.* Entomologia Experimentalis Et Applicata, 1991. **60**(2): p. 193-201.

737. Kaplan, J.E., et al., *Epidemiologic investigations of dengue infection in Mexico, 1980.* American Journal of Epidemiology, 1983. **117**(3): p. 335-343.

738. Kar, I., A. Eapen, and K.J. Ravindran, *Domestic breeding sources and their contribution in* Anopheles stephensi *breeding in Dindigul, Tamil Nadu.* Indian Journal of Malariology, 1996. **33**(4): p. 191-199.

739. Karch, S., et al., *[The culicidian fauna and its nuisance in Kinshasha (Zaire)].* Bulletin De La Societe De Pathologie Exotique, 1993. **86**(1): p. 68-75.

740. Karunaratne, S.H.P.P. and J. Hemingway, *Malathion resistance and prevalence of the malathion carboxylesterase mechanism in populations of mosquito vectors of disease in Sri Lanka.* Bulletin of the World Health Organization, 2001. **79**(11): p. 1060-1064.

741. Kasule, F.K. and L.M. Cook, *Phenotypic variability and heterozygosity at an esterase locus in the mosquito Aedes aegypti.* Heredity, 1988. **61 ( Pt 3)**: p. 427-431.

742. Katsuda, Y., et al., *Control of mosquito vectors of tropical infectious diseases: (1) bioefficacy of mosquito coils containing several pyrethroids and a synergist.* Southeast Asian Journal of Tropical Medicine and Public Health, 2008. **39**(1): p. 48-54.

743. Katsuda, Y., et al., *Control of mosquito vectors of tropical infectious diseases: (2) pyrethroid susceptibility of* Aedes Aegypti *( l) collected from different sites in Thailand.* Southeast Asian Journal of Tropical Medicine and Public Health, 2008. **39**(2): p. 229-234.

744. Kaul, H.N., et al., *Insecticide Susceptibility Studies in Populations of* Aedes-Aegypti *from Maharashtra-State, India.* Indian Journal of Medical Research, 1976. **64**(12): p. 1760-1768.

745. Kaul, H.N. and G. Geevarghese, *Comparative-Study of Ovitrap and Single Larva Survey Methods for the Surveillance of* Aedes Aegypti *in Pune City.* Indian Journal of Medical Research, 1979. **69**(Jan): p. 71-74.

746. Kawada, H., S. Honda, and M. Takagi, *Comparative laboratory study on the reaction of* Aedes Aegypti *and* Aedes albopictus *to different attractive cues in a mosquito trap.* Journal of Medical Entomology, 2007. **44**(3): p. 427-432.

747. Kawada, H., et al., *Field evaluation of spatial repellency of metofluthrin-impregnated latticework plastic strips against* Aedes Aegypti *(L.) and analysis of environmental factors affecting its efficacy in My Tho City, Tien Giang, Vietnam.* American Journal of Tropical Medicine and Hygiene, 2006. **75**(6): p. 1153-1157.

748. Kawada, H., et al., *Field evaluation of spatial repellency of metofluthrin impregnated plastic strips against mosquitoes in Hai Phong city, Vietnam.* American Journal of Tropical Medicine and Hygiene, 2005. **73**(2): p. 350-353.

749. Kay, B. and V.S. Nam, *New strategy against* Aedes Aegypti *in Vietnam.* Lancet, 2005. **365**(9459): p. 613-617.

750. Kay, B.H., et al., *Dengue fever. Reappearance in northern Queensland after 26 years.* Medical Journal of Australia, 1984. **140**(5): p. 264-268.

751. Kay, B.H., et al., *Point source inoculation of* mesocyclops *(Copepoda: Cyclopidae) gives widespread control of* Ochlerotatus *and* Aedes *(Diptera: Culicidae) immatures in service manholes and pits in north Queensland, Australia.* Journal of Medical Entomology, 2002. **39**(3): p. 469-474.

752. Kay, B.H., et al., *Control of* Aedes *vectors of dengue in three provinces of Vietnam by use of* Mesocyclops *(Copepoda) and community-based methods validated by entomologic, clinical, and serological surveillance.* American Journal of Tropical Medicine and Hygiene, 2002. **66**(1): p. 40-48.

753. Kay, B.H., G. Prakash, and R.G. Andre, Aedes albopictus *and other* Aedes (Stegomyia) *species in Fiji.* Journal of the American Mosquito Control Association, 1995. **11**(2 Pt 1): p. 230-234.

754. Kay, B.H., G. Prakash, and R.G. Andre, *Operational and Scientific Notes -* Aedes-Albopictus *and Other* Aedes (Stegomyia) *Species in Fiji.* Journal of the American Mosquito Control Association, 1995. **11**(2): p. 230-234.

755. Kay, B.H., et al., *Winter intervention against* Aedes Aegypti *(Diptera: Culicidae) larvae in subterranean habitats slows surface recolonization in summer.* Journal of Medical Entomology, 2002. **39**(2): p. 356-361.

756. Kay, B.H., et al., *The importance of subterranean mosquito habitat to arbovirus vector control strategies in north Queensland, Australia.* Journal of Medical Entomology, 2000. **37**(6): p. 846-853.

757. Kay, B.H., K.A. Sutton, and B.M. Russell, *A sticky entry-exit trap for sampling mosquitoes in subterranean habitats.* Journal of the American Mosquito Control Association, 2000. **16**(3): p. 262-265.

758. Keirans, J.E., *Larval Development of* Aedes Aegypti *(L) in Used Auto Tires.* Mosquito News, 1969. **29**(1): p. 43-46.

759. Kemp, A. and P.G. Jupp, *Potential for dengue in South Africa: mosquito ecology with particular reference to* Aedes aegypti*.* Journal of the American Mosquito Control Association, 1991. **7**(4): p. 574-583.

760. Kesavaraju, B., et al., *Behavioural responses of larval container mosquitoes to a size-selective predator.* Ecological Entomology, 2007. **32**(3): p. 262-272.

761. Kesavaraju, B. and S.A. Juliano, *Differential Behavioral Responses to Water-Borne Cues to Predation in Two Container-Dwelling Mosquitoes.* Annals of the Entomological Society of America, 2004. **97**(1): p. 194-201.

762. Kesavaraju, B. and S.A. Juliano, *Behavioral Responses of* Aedes albopictus *to a Predator Are Correlated with Size-Dependent Risk of Predation.* Annals of the Entomological Society of America, 2008. **101**(6): p. 1150-1153.

763. Kesavaraju, B., D.A. Yee, and S.A. Juliano, *Interspecific and intraspecific differences in foraging preferences of container-dwelling mosquitoes.* Journal of Medical Entomology, 2007. **44**(2): p. 215-221.

764. Khan, A.R., *Studies on the breeding habitats and seasonal prevalence of larval population of* Aedes Aegypti *(L.) and* Aedes albopictus *(skuse) in Dacca city.* Bangladesh Medical Research Council Bulletin, 1980. **6**(2): p. 45-52.

765. Khin, M.M. and K.A. Than, *Transovarial transmission of dengue 2 virus by* Aedes Aegypti *in nature.* American Journal of Tropical Medicine and Hygiene, 1983. **32**(3): p. 590-594.

766. Kilpatrick, J.W., R.J. Tonn, and Jatanase.S, *Evaluation of Ultra-Low-Volume Insecticide Dispensing Systems for Use in Single-Engined Aircraft and Their Effectiveness against* Aedes-Aegypti *Populations in South-East-Asia.* Bulletin of the World Health Organization, 1970. **42**(1): p. 1-14.

767. Kitron, U., et al., *Introduction of* Aedes albopictus *into a La Crosse virus - Enzootic site in Illinois.* Emerging Infectious Diseases, 1998. **4**(4): p. 627-630.

768. Kittayapong, P., et al., *Community participation and appropriate technologies for dengue vector control at transmission foci in Thailand.* Journal of the American Mosquito Control Association, 2006. **22**(3): p. 538-546.

769. Kittayapong, P., et al., *Host age effect and expression of cytoplasmic incompatibility in field populations of Wolbachia-superinfected* Aedes albopictus*.* Heredity, 2002. **88**(4): p. 270-274.

770. Kittayapong, P. and D. Strickman, *Distribution of container-inhabiting* Aedes *larvae (Diptera: Culicidae) at a dengue focus in Thailand.* Journal of Medical Entomology, 1993. **30**(3): p. 601-606.

771. Kittayapong, P., et al., *Suppression of dengue transmission by application of integrated vector control strategies at sero-positive GIS-Based foci.* American Journal of Tropical Medicine and Hygiene, 2008. **78**(1): p. 70-76.

772. Kittrayapong, P., V. Baimai, and S.L. O'Neill, *Field prevalence of Wolbachia in the mosquito vector* Aedes albopictus*.* American Journal of Tropical Medicine and Hygiene, 2002. **66**(1): p. 108-111.

773. Klassen, W. and A.W.A. Brown, *Genetics of Insecticide-Resistance + Several Visible Mutants in Aedes Aegypti.* Canadian Journal of Genetics and Cytology, 1964. **6**(1): p. 61-73.

774. Kline, D.L., et al., *Evaluation of the enantiomers of 1-octen-3-ol and 1-octyn-3-ol as attractants for mosquitoes associated with a freshwater swamp in Florida, USA.* Medical and Veterinary Entomology, 2007. **21**(4): p. 323-331.

775. Klobucar, A., et al., *First record of* Aedes albopictus *in Croatia.* Journal of the American Mosquito Control Association, 2006. **22**(1): p. 147-148.

776. Kloter, K.O., D.D. Bowman, and M.K. Carroll, *Evaluation of Some Ovitrap Materials Used for* Aedes-Aegypti *Surveillance.* Mosquito News, 1983. **43**(4): p. 438-441.

777. Kloter, K.O., et al., *An Experimental Evaluation of 6 Different Suction Traps for Attracting and Capturing Aedes-Aegypti.* Mosquito News, 1983. **43**(3): p. 297-301.

778. Knight, J.C. and S.A. Corbet, *Compounds affecting mosquito oviposition: structure-activity relationships and concentration effects.* Journal of the American Mosquito Control Association, 1991. **7**(1): p. 37-41.

779. Knio, K.M., et al., *A two-year survey on mosquitoes of Lebanon.* Parasite, 2005. **12**(3): p. 229-235.

780. Knox, T.B., et al., *Enhanced vector competence of* Aedes Aegypti *(Diptera: Culicidae) from the Torres Strait compared with mainland Australia for dengue 2 and 4 viruses.* Journal of Medical Entomology, 2003. **40**(6): p. 950-956.

781. Knudsenb *DENGUE - BANGLADESH: BACKGROUND*. PROMED, 2000.

782. knudsenb, B. *DENGUE - BANGLADESH: BACKGROUND*. 2000 [cited 2000 4 february].

783. Ko, Y.C., M.J. Chen, and S.M. Yeh, *The predisposing and protective factors against dengue virus transmission by mosquito vector.* American Journal of Epidemiology, 1992. **136**(2): p. 214-220.

784. Kobayashi, M., N. Nihei, and T. Kurihara, *Analysis of northern distribution of* Aedes albopictus *(Diptera: Culicidae) in Japan by geographical information system.* Journal of Medical Entomology, 2002. **39**(1): p. 4-11.

785. Koenraadt, C.J., et al., *Dengue knowledge and practices and their impact on* Aedes Aegypti *populations in Kamphaeng Phet, Thailand.* American Journal of Tropical Medicine and Hygiene, 2006. **74**(4): p. 692-700.

786. Koenraadt, C.J.M., et al., *Spatial and temporal patterns in pupal and adult production of the dengue vector* Aedes Aegypti *in Kamphaeng Phet, Thailand.* American Journal of Tropical Medicine and Hygiene, 2008. **79**(2): p. 230-238.

787. Koenraadt, C.J.M. and L.C. Harrington, *Flushing effect of rain on container-inhabiting mosquitoes* Aedes Aegypti *and* Culex pipiens *(Diptera : Culicidae).* Journal of Medical Entomology, 2008. **45**(1): p. 28-35.

788. Koenraadt, C.J.M., et al., *Standardizing container classification for immature* Aedes Aegypti *surveillance in Kamphaeng Phet, Thailand.* Journal of Medical Entomology, 2007. **44**(6): p. 938-944.

789. Kohn, M., *Susceptibility of adult* Aedes Aegypti *(L.) and* Culex quinquefasciatus *Say (Diptera; Culicidae) to DDT in Kampuchea.* Folia Parasitologica (Praha), 1991. **38**(3): p. 269-274.

790. Konishi, E., *Size of blood meals of* Aedes albopictus *and* Culex tritaeniorhynchus *(Diptera: Culicidae) feeding on an unrestrained dog infected with* Dirofilaria immitis *(Spirurida: Filariidae).* Journal of Medical Entomology, 1989. **26**(6): p. 535-538.

791. Konishi, E., *Susceptibility of* Aedes albopictus *and* Culex tritaeniorhynchus *(Diptera: Culicidae) collected in Miki City, Japan, to* Dirofilaria immitis *(Spirurida: Filariidae).* Journal of Medical Entomology, 1989. **26**(5): p. 420-424.

792. Konishi, E., Culex tritaeniorhynchus *and* Aedes albopictus *(Diptera: Culicidae) as natural vectors of* Dirofilaria immitis *(Spirurida: Filariidae) in Miki City, Japan.* Journal of Medical Entomology, 1989. **26**(4): p. 294-300.

793. Korgaonkar, N.S., et al., *Sampling of adult mosquito vectors with Mosquito Magnet Pro in Panaji, Goa, India.* Journal of the American Mosquito Control Association, 2008. **24**(4): p. 604-607.

794. Kow, C.Y., L.L. Koon, and P.F. Yin, *Detection of dengue viruses in field caught male* Aedes Aegypti *and* Aedes albopictus *(Diptera: Culicidae) in Singapore by type-specific PCR.* Journal of Medical Entomology 2001. **38**(4): p. 475-479.

795. Kroeger, A., et al., *Effective control of dengue vectors with curtains and water container covers treated with insecticide in Mexico and Venezuela: cluster randomised trials.* British Medical Journal, 2006. **332**(7552): p. 1247-1252.

796. Krueger, A. and R.M. Hagen, *Short communication: First record of* Aedes albopictus *in Gabon, Central Africa.* Tropical Medicine & International Health, 2007. **12**(9): p. 1105-1107.

797. Kumar, A. and K.S. Rai, *Organization of a cloned repetitive DNA fragment in mosquito genomes (Diptera: Culicidae).* Genome, 1991. **34**(6): p. 998-1006.

798. Kumar, A., et al., *An outbreak of dengue fever in rural areas of northern India.* Journal of Communicable Diseases, 2001. **33**(4): p. 274-281.

799. Kumar, K., et al., *Investigation of an outbreak of chikungunya in Malegaon Municipal areas of Nasik district, Maharashtra (India) and its control.* Journal of Vector Borne Diseases, 2008. **45**(2): p. 157-163.

800. Kumar, N.P., et al., *A226V mutation in virus during the 2007 chikungunya outbreak in Kerala, India.* Journal of General Virology, 2008. **89**: p. 1945-1948.

801. Kumar, R.R., et al., *Breeding habitats and larval indices of* Aedes Aegypti *(L) in residential areas of Rajahmundry town, Andhra Pradesh.* Journal of Communicable Diseases, 2002. **34**(1): p. 50-58.

802. Kumarasamy, V., et al., *Re-emergence of Chikungunya virus in Malaysia.* Medical Journal of Malaysia, 2006. **61**(2): p. 221-225.

803. Kusriastuti , R. *CHIKUNGUNYA - INDONESIA (JAVA) (02)* PROMED, 2004.

804. Labarthe, N., et al., *Mosquito frequency and feeding habits in an enzootic canine dirofilariasis area in Niteroi, state of Rio de Janeiro, Brazil.* Memorias Do Instituto Oswaldo Cruz, 1998. **93**(2): p. 145-154.

805. Lagrotta, M.T., W.D. Silva, and R. Souza-Santos, *Identification of key areas for* Aedes Aegypti *control through geoprocessing in Nova Iguacu, Rio de Janeiro state, Brazil.* Cadernos de Saúde Pública, 2008. **24**(1): p. 70-80.

806. Lai, C.H., et al., *Susceptibility of mosquitoes in central Taiwan to natural infections of* Dirofilaria immitis*.* Medical and Veterinary Entomology, 2001. **15**(1): p. 64-67.

807. Laille, M., P. Fauran, and F. Rodhain, *[The presence of* Aedes (Stegomyia) albopictus *in the Fiji Islands].* Bulletin De La Societe De Pathologie Exotique, 1990. **83**(3): p. 394-398.

808. Laille, M., P. Fauran, and F. Rodhain, *[Note on the Presence of* Aedes-(Stegomyia)-Albopictus *in Fiji Islands].* Bulletin De La Societe De Pathologie Exotique, 1990. **83**(3): p. 394-398.

809. Laille, M., et al., *[Dengue epidemic in New Caledonia (1989). Environmental factors and prevention].* Bulletin De La Societe De Pathologie Exotique, 1993. **86**(5 Pt 2): p. 442-449.

810. Laird, M., et al., *Japanese* Aedes albopictus *among four mosquito species reaching New Zealand in used tires.* Journal of the American Mosquito Control Association, 1994. **10**(1): p. 14-23.

811. Lamont, P. *DENGUE/DHF UPDATES (34)*. PROMED, 2002.

812. Lampman, R., S. Hanson, and R. Novak, *Seasonal abundance and distribution of mosquitoes at a rural waste tire site in Illinois.* Journal of the American Mosquito Control Association, 1997. **13**(2): p. 193-200.

813. Lan, N.T.P., et al., *Protective and Enhancing HLA Alleles, HLA-DRB1*0901 and HLA-A*24, for Severe Forms of Dengue Virus Infection, Dengue Hemorrhagic Fever and Dengue Shock Syndrome.* Plos Neglected Tropical Diseases, 2008. **2**(10): p. -.

814. LaPointe, D.A., M.L. Goff, and C.T. Atkinson, *Comparative susceptibility of introduced forest dwelling mosquitoes in Hawai'i to avian malaria, Plasmodium relictum.* Journal of Parasitology, 2005. **91**(4): p. 843-849.

815. Laranja, A.T., A.J. Manzato, and H.E.M.D. Bicudo, *Caffeine effect on mortality and oviposition in successive generations of* Aedes aegypti*.* Revista de Saude Publica, 2006. **40**(6): p. 1112-1117.

816. Laras, K., et al., *Tracking the re-emergence of epidemic chikungunya virus in Indonesia.* Transactions of the Royal Society of Tropical Medicine and Hygiene, 2005. **99**(2): p. 128-141.

817. Lardeux, F.J., *Biological control of Culicidae with the copepod* Mesocyclops aspericornis *and larvivorous fish (Poeciliidae) in a village of French Polynesia.* Medical and Veterinary Entomology, 1992. **6**(1): p. 9-15.

818. Larghi, O. *DENGUE - ARGENTINA, BRAZIL, PARAGUAY: ALERT*. PROMED, 2000.

819. Lazcano, J.A.B., et al., *[Ecological factors linked to the presence of* Aedes Aegypti *larvae in highly infested areas of Playa, a municipality belonging to Ciudad de La Habana, Cuba].* Revista Panamericana de Salud Publica, 2006. **19**(6): p. 379-384.

820. Le Gonidec, G., J.P. Quene, and P. Fauran, *[On an outbreak caused by dengue type 4 virus, in Thio, New Caledonia. Epidemiological and clinical aspects (author's transl)].* Bulletin De La Société De Pathologie Exotique Et De Ses Filiales, 1982. **75**(2): p. 141-150.

821. Lee, D. and C.G. Moore, *Mosquito Studies during an Interepidemic Outbreak of Dengue in Puerto-Rico.* Mosquito News, 1973. **33**(4): p. 506-509.

822. Lee, H.L., et al., *Impact of larviciding with a* Bacillus thuringiensis israelensis *formulation, VectoBac WG, on dengue mosquito vectors in a dengue endemic site in Selangor State, Malaysia.* Southeast Asian Journal of Tropical Medicine and Public Health, 2008. **39**(4): p. 601-609.

823. Lee, J.J. and M.J. Klowden, *A male accessory gland protein that modulates female mosquito (Diptera: Culicidae) host-seeking behavior.* Journal of the American Mosquito Control Association, 1999. **15**(1): p. 4-7.

824. Lee, Y.W. and J. Zairi, *Susceptibility of laboratory and field-collected* Aedes Aegypti *and* Aedes albopictus *to* Bacillus thuringiensis israelensis *H-14.* Journal of the American Mosquito Control Association, 2006. **22**(1): p. 97-101.

825. Leisnham, P.T. and S.A. Juliano, *Spatial and temporal patterns of coexistence between competing* Aedes *mosquitoes in urban Florida.* Oecologia, 2009. **160**(2): p. 343-352.

826. Leisnham, P.T., L.M. Sala, and S.A. Juliano, *Geographic variation in adult survival and reproductive tactics of the mosquito* Aedes albopictus*.* Journal of Medical Entomology, 2008. **45**(2): p. 210-221.

827. Lenhart, A., et al., *Insecticide-treated bednets to control dengue vectors: preliminary evidence from a controlled trial in Haiti.* Tropical Medicine & International Health, 2008. **13**(1): p. 56-67.

828. Lenhart, A.E., et al., *Use of the pupal/demographic-survey technique to identify the epidemiologically important types of containers producing* Aedes Aegypti *(L.) in a dengue-endemic area of Venezuela.* Annals of Tropical Medicine and Parasitology, 2006. **100 Suppl 1**: p. S53-S59.

829. Leontsini, E., et al., *Effect of a community-based* Aedes Aegypti *control programme on mosquito larval production sites in El Progreso, Honduras.* Transactions of the Royal Society of Tropical Medicine and Hygiene, 1993. **87**(3): p. 267-271.

830. Lerdthusnee, K. and T. Chareonviriyaphap, *Comparison of isozyme patterns of* Aedes Aegypti *populations collected from pre- and post-*Bacillus thuringiensis israelensis *treatment sites in Thailand.* Journal of the American Mosquito Control Association, 1999. **15**(1): p. 48-52.

831. Leroy, E.M., et al., *Concurrent chikungunya and dengue virus infections during simultaneous outbreaks, Gabon, 2007.* Emerging Infectious Diseases, 2009. **15**(4): p. 591-593.

832. Levi, J.E., et al., *Evaluation of a commercial real-time PCR kit for detection of dengue virus in samples collected during an outbreak in Goiania, central Brazil, in 2005.* Journal of Clinical Microbiology, 2007. **45**(6): p. 1893-1897.

833. Li, C.F., et al., *Rainfall, abundance of* Aedes Aegypti *and dengue infection in Selangor, Malaysia.* Southeast Asian Journal of Tropical Medicine and Public Health, 1985. **16**(4): p. 560-568.

834. Li, F.S., et al., *Etiologic and serologic investigations of the 1980 epidemic of dengue fever on Hainan Island, China.* American Journal of Tropical Medicine and Hygiene, 1986. **35**(5): p. 1051-1054.

835. Li, F.S., et al., *Etiologic and serologic investigations of the 1980 epidemic of dengue fever on Hainan Island, China.* American Journal of Tropical Medicine and Hygiene, 1986. **35**(5): p. 1051-1054.

836. Lien, J.C. and Y.N. Lin, *[The pathogens of Taiwan mosquitoes--Coelomomyces species].* Kaohsiung Journal of Medical Sciences - Gaoxiong Yi Xue Ke Xue Za Zhi, 1990. **6**(7): p. 350-359.

837. Lima, E.P., et al., *[*Aedes Aegypti *resistance to temefos in counties of Ceara State].* Revista da Sociedade Brasileira de Medicina Tropical, 2006. **39**(3): p. 259-263.

838. Lima, R.S. and V.M. Scarpassa, *Evidence of two lineages of the dengue vector* Aedes Aegypti *in the Brazilian Amazon, based on mitochondrial DNA ND4 gene sequences.* Genetics and Molecular Biology, 2009. **32**(2): p. 414-422.

839. Lin, H.M., et al., *[Dengue vector density survey in Liuchiu, Pintung, Taiwan].* Chinese Journal of Microbiology and Immunology - Zhonghua Min Guo Wei Sheng Wu Ji Mian Yi Xue Za Zhi 1986. **19**(3): p. 218-223.

840. Linthicum, K.J., et al., *Rift Valley fever virus (family Bunyaviridae, genus Phlebovirus). Isolations from Diptera collected during an inter-epizootic period in Kenya.* Journal of Hygiene, 1985. **95**(1): p. 197-209.

841. Linthicum, K.J., et al., *Introduction and potential establishment of* Aedes albopictus *in California in 2001.* Journal of the American Mosquito Control Association, 2003. **19**(4): p. 301-308.

842. Liu, H.W., et al., *Clinical observations of virologically confirmed dengue fever in the 1987 outbreak in southern Taiwan.* Gaoxiong Yi Xue Ke Xue Za Zhi, 1989. **5**(1): p. 42-49.

843. Lloyd, L.S., et al., *The design of a community-based health education intervention for the control of Aedes aegypti.* American Journal of Tropical Medicine and Hygiene, 1994. **50**(4): p. 401-411.

844. Lofgren, C.S., et al., *Effectiveness of Ultra-Low-Volume Applications of Malathion at a Rate of 3 Us Fluid Ounces Per Acre in Controlling* Aedes-Aegypti *in Thailand.* Bulletin of the World Health Organization, 1970. **42**(1): p. 27-35.

845. Lofgren, C.S., et al., *Effectiveness of Ultra-Low-Volume Applications of Malathion at a Rate of 6 Us Fluid Ounces Per Acre in Controlling* Aedes-Aegypti *in a Large-Scale Test at Nakhon-Sawan, Thailand.* Bulletin of the World Health Organization, 1970. **42**(1): p. 15-25.

846. Lok, C.K., N.S. Kiat, and T.K. Koh, *An autocidal ovitrap for the control and possible eradication of Aedes aegypti.* Southeast Asian Journal of Tropical Medicine and Public Health, 1977. **8**(1): p. 56-62.

847. Lopes, J., et al., *[*Aedes (Stegomyia) aegypti *L. and associated culicidae fauna in a urban area of southern Brazil].* Revista de Saúde Pública, 1993. **27**(5): p. 326-333.

848. Lopes, J., et al., *Dispersion of* Aedes Aegypti *(Linnaeus, 1762) and* Aedes albopictus *(Skuse, 1894) in the rural zone of North Parana State.* Brazilian Archives of Biology and Technology, 2004. **47**(5): p. 739-746.

849. Lopez, C., M.M.D. Ibanez, and C.E. Machadoallison, *[Larval Density and Population-Dynamics of* Aedes-Aegypti *(L) in Laboratory Conditions].* Acta Cientifica Venezolana, 1976. **27**(6): p. 317-320.

850. Lopez Lastra, C.C., et al., *Host range and specificity of an Argentinean isolate of the aquatic fungus Leptolegnia chapmanii (Oomycetes: Saprolegniales), a pathogen of mosquito larvae (Diptera: Culicidae).* Mycopathologia, 2004. **158**(3): p. 311-315.

851. Lorenz, L., et al., *The effect of colonization upon* Aedes Aegypti *susceptibility to oral infection with yellow fever virus.* American Journal of Tropical Medicine and Hygiene, 1984. **33**(4): p. 690-694.

852. Lorimer, N., L.P. Lounibos, and J.L. Petersen, *Field Trials with a Translocation Homozygote in* Aedes-Aegypti *Diptera-Culicidae for Population Replacement.* Journal of Economic Entomology, 1976. **69**(3): p. 405-409.

853. Lorono Pino, M.A., et al., *Epidemic dengue 4 in the Yucatan, Mexico, 1984.* Revista do Instituto de Medicina Tropical de Sao Paulo, 1993. **35**(5): p. 449-455.

854. Lounibos, L.P. and R.L. Escher, *Sex ratios of mosquitoes from long-term censuses of Florida tree holes.* Journal of the American Mosquito Control Association, 2008. **24**(1): p. 11-15.

855. Lounibos, L.P., R.L. Escher, and R. Lourenco-de-Oliveria, *Asymmetric evolution of photoperiodic diapause in temperate and tropical invasive populations of* Aedes albopictus *(Diptera : Culicidae).* Annals of the Entomological Society of America, 2003. **96**(4): p. 512-518.

856. Lourenco de Oliveira, R., et al., *Large genetic differentiation and low variation in vector competence for dengue and yellow fever viruses of* Aedes albopictus *from Brazil, the United States, and the Cayman Islands.* American Journal of Tropical Medicine and Hygiene, 2003. **69**(1): p. 105-114.

857. Lourenco-de-Oliveira, R., et al., *The invasion of urban forest by dengue vectors in Rio de Janeiro.* Journal of Vector Ecology

, 2004. **29**(1): p. 94-100.

858. Lourenco-de-Oliveira, R., et al., *Dengue virus type 3 isolation from* Aedes Aegypti *in the municipality of Nova Iguacu, State of Rio de Janeiro.* Memorias do Instituto Oswaldo Cruz, 2002. **97**(6): p. 799-800.

859. Lourenco-de-Oliveira, R., et al., *Dengue virus type 3 isolation from* Aedes Aegypti *in the municipality of Nova Iguacu, State of Rio de Janeiro.* Memorias Do Instituto Oswaldo Cruz, 2002. **97**(6): p. 799-800.

860. Lourenco-de-Oliveira, R., et al., *Comparison of different uses of adult traps and ovitraps for assessing dengue vector infestation in endemic areas.* Journal of the American Mosquito Control Association, 2008. **24**(3): p. 387-392.

861. Lourenco-de-Oliveira, R., et al., Aedes Aegypti *in Brazil: genetically differentiated populations with high susceptibility to dengue and yellow fever viruses.* Transactions of the Royal Society of Tropical Medicine and Hygiene, 2004. **98**(1): p. 43-54.

862. Lourenco-de-Oliveira, R., et al., *Oral susceptibility to yellow fever virus of* Aedes Aegypti *from Brazil.* Memorias Do Instituto Oswaldo Cruz, 2002. **97**(3): p. 437-439.

863. Lowenberg-Neto, P. and M.A. Navarro-Silva, *[First report of* Aedes albopictus *in the state of Santa Catarina, Brazil].* Revista de Saúde Pública, 2002. **36**(2): p. 246-247.

864. Lucia, A., et al., *Efficacy of a new combined larvicidal-adulticidal ultralow volume formulation against* Aedes Aegypti *(Diptera: Culicidae), vector of dengue.* Parasitology Research, 2009. **104**(5): p. 1101-1107.

865. Lugo, E.D., et al., *Identification of* Aedes albopictus *in urban Nicaragua.* Journal of the American Mosquito Control Association, 2005. **21**(3): p. 325-327.

866. Macdonald, W.W. and Rajapaks.N, *Survey of Distribution and Relative Prevalence of* Aedes-Aegypti *in Ssabah, Brunei, and Sarawak.* Bulletin of the World Health Organization, 1972. **46**(2): p. 203-209.

867. Machado-Allison, C.E. and G.B. Craig, *Geographic Variation in Resistance to Desiccation in* Aedes-Aegypti *and* a-Atropalpus *(Diptera - Culicidae).* Annals of the Entomological Society of America, 1972. **65**(3): p. 542-547.

868. Maciel-de-Freitas, R., et al., *Variation in* Aedes Aegypti *(Diptera: Culicidae) container productivity in a slum and a suburban district of Rio de Janeiro during dry and wet seasons.* Memorias Do Instituto Oswaldo Cruz, 2007. **102**(4): p. 489-496.

869. Maciel-de-Freitas, R., et al., *Mosquito traps designed to capture* Aedes Aegypti *(Diptera: Culicidae) females: preliminary comparison of Adultrap, MosquiTRAP and backpack aspirator efficiency in a dengue-endemic area of Brazil.* Memorias Do Instituto Oswaldo Cruz, 2008. **103**(6): p. 602-605.

870. Macoris, M.D., et al., *Resistance of* Aedes Aegypti *from the State of Sao Paulo, Brazil, to organophosphates insecticides.* Memorias Do Instituto Oswaldo Cruz, 2003. **98**(5): p. 703-708.

871. Macoris, M.D.D., et al., *Association of insecticide use and alteration on* Aedes Aegypti *susceptibility status.* Memorias Do Instituto Oswaldo Cruz, 2007. **102**(8): p. 895-900.

872. Macoris, M.L., et al., *Factors favoring houseplant container infestation with* Aedes Aegypti *larvae in Marilia, Sao Paulo, Brazil.* Revista Panamericana de Salud Pública, 1997. **1**(4): p. 280-286.

873. Madeira, N.G., C.A. Macharelli, and L.R. Carvalho, *Variation of the oviposition preferences of* Aedes Aegypti *in function of substratum and humidity.* Memorias Do Instituto Oswaldo Cruz, 2002. **97**(3): p. 415-420.

874. Madhukar, B.V. and M.K.K. Pillai, *Insecticide Susceptibility Studies in Indian Strains of* Aedes Aegypti *L.* Mosquito News, 1968. **28**(2): p. 222-225.

875. Madhukar, B.V. and M.K.K. Pillai, *Development of Organophosphorus Resistance in Indian Strains of* Aedes-Aegypti *(L).* Bulletin of the World Health Organization, 1970. **43**(5): p. 735-742.

876. Maestre-Serrano, R., et al., *[Presence of Haemagogus equinus Theobald, 1903 (Diptera: Culicidae), in Soledad and Malambo, in the Province of Atlantico, Colombia].* Biomedica, 2008. **28**(1): p. 99-107.

877. Maguire, M., et al., *Simulation modelling of* Aedes Aegypti *prevalence, an environmental hazard surveillance tool for the control of dengue epidemics.* International Journal of Environmental Health Research, 1999. **9**(4): p. 253-259.

878. Mahadev, P.V., et al., *Dengue in Gujarat state, India during 1988 & 1989.* Indian Journal of Medical Research, 1993. **97**: p. 135-144.

879. Mahadev, P.V., et al., *Activity of dengue-2 virus and prevalence of* Aedes Aegypti *in the Chirimiri colliery area, Madhya Pradesh, India.* Southeast Asian Journal of Tropical Medicine and Public Health, 1997. **28**(1): p. 126-137.

880. Mahadev, P.V., et al., *Activity of dengue-2 virus and prevalence of* Aedes Aegypti *in the Chirimiri colliery area, Madhya Pradesh, India.* Southeast Asian Journal of Tropical Medicine and Public Health, 1997. **28**(1): p. 126-137.

881. Mahadev, P.V.M., *A Case-Study of* Aedes-Aegypti *Prevalence by Settlement Types in Dehu Town Group of Maharashtra State.* Indian Journal of Medical Research, 1983. **78**(Oct): p. 537-546.

882. Mahadev, P.V.M., V. Dhanda, and P.S. Shetty, Aedes-Aegypti *(L) in Maharashtra State - Distribution and Larval Habitats.* Indian Journal of Medical Research, 1978. **67**(Apr): p. 562-580.

883. Mahadev, P.V.M., P.V. Fulmali, and A.C. Mishra, *A preliminary study of multilevel geographic distribution & prevalence of* Aedes Aegypti *(Diptera : Culicidae) in the state of Goa, India.* Indian Journal of Medical Research, 2004. **120**(3): p. 173-182.

884. Mahadev, P.V.M. and G. Geevarghese, *Comparison of Single Larva and Conventional Pool Methods for the Study of* Aedes-Aegypti *in Tyre Dumps.* Indian Journal of Medical Research, 1978. **68**(Dec): p. 934-939.

885. Mahadev, P.V.M., et al., *Dengue in Gujarat State, India during 1988 and 1989.* Indian Journal of Medical Research Section a-Infectious Diseases, 1993. **97**: p. 135-144.

886. Mahilum, M.M., et al., *Evaluation of the present dengue situation and control strategies against* Aedes Aegypti *in Cebu City, Philippines.* Journal of Vector Ecology 2005. **30**(2): p. 277-83.

887. Mahmood, F., *Susceptibility of geographically distinct* Aedes Aegypti *L. from Florida to Dirofilaria immitis (Leidy) infection.* Journal of Vector Ecology 2000. **25**(1): p. 36-47.

888. Mahmood, F. and J.K. Nayar, *Effects of* Dirofilaria-Immitis *(Nematoda, Filarioidea) Infection on Life Table Characteristics of Susceptible and Refractory Strains of* Aedes-Aegypti *(Vero-Beach) (Diptera, Culicidae).* Florida Entomologist, 1989. **72**(4): p. 567-578.

889. Mahmood, F. and J.K. Nayar, *Effect of* Dirofilaria-Immitis *(Nematoda, Filarioidea) Infection on Rate of Diuresis in Susceptible and Refractory Strains of* Aedes-Aegypti *(Vero Beach) (Diptera, Culicidae).* Florida Entomologist, 1989. **72**(4): p. 579-585.

890. Mahsinah, S.A. *DENGUE/DHF UPDATE 2004 (20) [Malaysia/ Sri Lanka/ Indonesia]*. 2004 [cited 2004 3 July]; New Straits Times, 3 Jul 2004 [edited].

891. Mallet, E.C., et al., *[Hemorrhagic dengue with shock in children in French Polynesia].* Bulletin de la Societe de Pathologie Exotique, 1993. **86**(5 Pt 2): p. 450-454.

892. Mangara, S.G., et al., *The risk of dengue hemorrhagic fever (DHF) outbreak based on vector density in Kurau, Riau province, Indonesia.* Southeast Asian Journal of Tropical Medicine and Public Health, 2000. **31 Suppl 1**: p. 134-139.

893. Manrique-Saide, P., et al., Ochlerotatus taeniorhynchus*: A probable vector of* Dirofilaria immitis *in coastal areas of Yucatan, Mexico.* Journal of Medical Entomology, 2008. **45**(1): p. 169-171.

894. Manrique-Saide, P., et al., *Pupal surveys for* Aedes Aegypti *surveillance and potential targeted control in residential areas of Merida, Mexico.* Journal of the American Mosquito Control Association, 2008. **24**(2): p. 289-298.

895. Mariappan, T., R. Srinivasan, and P. Jambulingam, *Defective rainwater harvesting structure and dengue vector productivity compared with peridomestic habitats in a coastal town in southern India.* Journal of Medical Entomology, 2008. **45**(1): p. 148-156.

896. Markon, C. *DENGUE/DHF - INDONESIA (03)*. 1998 [cited 1998 18 April].

897. Markon, C. *DENGUE - INDIA (MAHARASHTRA) (03)*. 1998 [cited 1998 5 September]; Deccan Chronicle 3 September.

898. Markon, C. *DENGUE/DHF - INDONESIA (03)*. PROMED, 1998.

899. Markon, C. *DENGUE - PHILIPPINES (02)*. PROMED, 1998.

900. Markon, C. *DENGUE - INDIA (MUMBAI)*. 1999 [cited 1999 11 October]; Times of India 11 Oct 1999 [edited].

901. Marques, G.R. and O.P. Forattini, *[*Aedes albopictus *in soil bromeliads in Ilhabela, coastal area of Southeastern Brazil].* Revista de Saúde Pública, 2005. **39**(4): p. 548-552.

902. Marques, G.R., R.L. Santos, and O.P. Forattini, *[*Aedes albopictus *in bromeliads of anthropic environment in Sao Paulo State, Brazil].* Revista de Saúde Pública, 2001. **35**(3): p. 243-248.

903. Marquetti, M.C., et al., *[Proportional abundance of culicidae in the urban ecosystem of Havana City].* Revista Cubana de Medicina Tropical, 1999. **51**(3): p. 181-184.

904. Marquetti, M.C., et al., *[Ecologic indexes in the surveillance system of* Aedes Aegypti *(Diptera: Culicidae) in Cuba].* Revista Cubana de Medicina Tropical, 1999. **51**(2): p. 79-82.

905. Marquetti, M.C., et al., *[The incidence of Culicidae in an urban area of Ciudad de La Habana during 1995].* Revista Cubana de Medicina Tropical, 1998. **50**(2): p. 138-142.

906. Marquetti Mdel, C., et al., *[The performance of the* Aedes Aegypti *eradication program in 2 towns of Ciudad de La Habana province, 1990-1992].* Revista Cubana de Medicina Tropical, 1996. **48**(3): p. 174-177.

907. Marquetti Mdel, C., et al., *[The effect of abiotic factors on the incidence of* Aedes Aegypti *in the town of 10 de Octobre, Ciudad de la Habana Province, 1982-1992].* Revista Cubana de Medicina Tropical, 1995. **47**(2): p. 88-92.

908. Marshall, M., A.-L. Banks, and B. Barrett *Dengue/DHF update 2007 (11)* PROMED, 2007.

909. Marten, G.G., *Elimination of* Aedes albopictus *from tire piles by introducing Macrocyclops albidus (Copepoda, Cyclopidae).* Journal of the American Mosquito Control Association, 1990. **6**(4): p. 689-693.

910. Marti, G.A., et al., *Evaluation of* Mesocyclops annulatus *(Copepoda: Cyclopoidea) as a control agent of* Aedes Aegypti *(Diptera: Culicidae) in Argentina.* Memorias Do Instituto Oswaldo Cruz, 2004. **99**(5): p. 535-540.

911. Martinez-Ibarra, J.A., et al., *Indigenous fish species for the control of* Aedes Aegypti *in water storage tanks in Southern Mexico.* Biocontrol, 2002. **47**(4): p. 481-486.

912. Martinez-Ibarra, J.A., et al., *Influence of plant abundance on nectar feeding by* Aedes Aegypti *(Diptera: Culicidae) in southern Mexico.* Journal of Medical Entomology, 1997. **34**(6): p. 589-593.

913. Martins, A.J., et al., *Chitin synthesis inhibitor effect on* Aedes Aegypti *populations susceptible and resistant to organophosphate temephos.* Pest Management Science, 2008. **64**(6): p. 676-680.

914. Martins, V.E., et al., *[First report of* Aedes (Stegomyia) albopictus *in the state of Ceara, Brazil].* Revista de Saúde Pública, 2006. **40**(4): p. 737-739.

915. Massad, E., et al., *The risk of yellow fever in a dengue-infested area.* Transactions of the Royal Society of Tropical Medicine and Hygiene, 2001. **95**(4): p. 370-374.

916. Masuh, H., et al., *Field evaluation of a smoke-generating formulation containing beta-cypermethrin against the dengue vector in Argentina.* Journal of the American Mosquito Control Association, 2003. **19**(1): p. 53-57.

917. Masuh, H., et al., Aedes Aegypti *(Diptera : Culicidae): monitoring of populations to improve control strategies in Argentina.* Parasitology Research, 2008. **103**(1): p. 167-170.

918. Mathiot, C.C., et al., *An outbreak of human Semliki Forest virus infections in Central African Republic.* American Journal of Tropical Medicine and Hygiene, 1990. **42**(4): p. 386-393.

919. Mayers, P.J., *Recent Introduction of* Aedes-Aegypti *in Bermuda.* Mosquito News, 1983. **43**(3): p. 361-362.

920. Mazine, C.A., et al., *Disposable containers as larval habitats for* Aedes Aegypti *in a city with regular refuse collection: a study in Marilia, Sao Paulo State, Brazil.* Acta Tropica, 1996. **62**(1): p. 1-13.

921. Mazzarri, M.B. and G.P. Georghiou, *Characterization of resistance to organophosphate, carbamate, and pyrethroid insecticides in field populations of* Aedes Aegypti *from Venezuela.* Journal of the American Mosquito Control Association, 1995. **11**(3): p. 315-322.

922. McBride, W.J.H., *Deaths associated with dengue haemorrhagic fever: the first in Australia in over a century.* Medical Journal of Australia, 2005. **183**(1): p. 35-37.

923. Mcclelland, G.A., *Field Observations on Periodicity and Site Preference in Oviposition by* Aedes Aegypti *(L) and Related Mosquitoes (Diptera - Culicidae) in Kenya.* Proceedings of the Royal Entomological Society of London Series a-General Entomology, 1968. **43**: p. 147-154.

924. Mcclelland, G.A., W.K. Hartberg, and C.M. Courtois, *Aedes-Mascarensis Macgregor on Mauritius .1. Ecology in Relation to* Aedes-Albopictus *(Skuse) (Diptera-Culicidae).* Journal of Medical Entomology, 1973. **10**(6): p. 570-577.

925. McCrae, A.W. and B.G. Kirya, *Yellow fever and Zika virus epizootics and enzootics in Uganda.* Transactions of the Royal Society of Tropical Medicine and Hygiene, 1982. **76**(4): p. 552-562.

926. McHugh, C.P., *Distributional records from the U.S. Air Force ovitrapping program--1990.* Journal of the American Mosquito Control Association, 1991. **7**(3): p. 499-501.

927. McHugh, C.P., *Distributional records from the U.S. Air Force ovitrapping program--1991.* Journal of the American Mosquito Control Association, 1992. **8**(2): p. 198-199.

928. McHugh, C.P., *Distributional records for Aedes mosquitoes from the U.S. Air Force ovitrapping program-1992.* Journal of the American Mosquito Control Association, 1993. **9**(3): p. 352-355.

929. McHugh, C.P. and P.A. Hanny, *Records of* Aedes albopictus*, Ae. aegypti and Ae. triseriatus from the U.S. Air Force ovitrapping program--1989.* Journal of the American Mosquito Control Association, 1990. **6**(3): p. 549-551.

930. Mchugh, C.P. and A.M. Vandeberg, *Records of* Aedes-Albopictus*,* Aedes-Aegypti *and* Aedes-Triseriatus *from the United-States-Air-Force Ovitrapping Program 1988.* Journal of the American Mosquito Control Association, 1989. **5**(3): p. 440-443.

931. McLain, D.K., K.S. Rai, and M.J. Fraser, *Intraspecific and interspecific variation in the sequence and abundance of highly repeated DNA among mosquitoes of the* Aedes albopictus *subgroup.* Heredity, 1987. **58 ( Pt 3)**: p. 373-381.

932. Medronho, R.A., et al., Aedes Aegypti *immature forms distribution according to type of breeding site.* American Journal of Tropical Medicine and Hygiene, 2009. **80**(3): p. 401-404.

933. Meeraus, W.H., J.S. Armistead, and J.R. Arias, *Field comparison of novel and gold,standard traps for collecting* Aedes albopictus *in Northern Virginia.* Journal of the American Mosquito Control Association, 2008. **24**(2): p. 244-248.

934. Mehendale, S.M., et al., *Outbreak of dengue fever in rural areas of Parbhani district of Maharashtra (India).* Indian Journal of Medical Research, 1991. **93**: p. 6-11.

935. Melluso, C. *WEST NILE VIRUS,* AEDES ALBOPICTUS *- USA (MARYLAND)*. PROMED, 2002.

936. Mendez, F., et al., *Human and mosquito infections by dengue viruses during and after epidemics in a dengue-endemic region of Colombia.* American Journal of Tropical Medicine and Hygiene, 2006. **74**(4): p. 678-83.

937. Mendez, F., et al., *Human and mosquito infections by dengue viruses during and after epidemics in a dengue-endemic region of Colombia.* American Journal of Tropical Medicine and Hygiene, 2006. **74**(4): p. 678-683.

938. Mendoza, F., S. Ibanez-Bernal, and F.J. Cabrero-Sanudo, *A standardized sampling method to estimate mosquito richness and abundance for research and public health surveillance programmes.* Bulletin of Entomological Research, 2008. **98**(4): p. 323-332.

939. Mercado-Hernandez, R., et al., *The association of* Aedes Aegypti *and Ae. albopictus in Allende, Nuevo Leon, Mexico.* Journal of the American Mosquito Control Association, 2006. **22**(1): p. 5-9.

940. Mercado-Hernandez, R., I. Fernandez-Salas, and H. Villarreal-Martinez, *Spatial distribution of the larval indices of* Aedes Aegypti *in Guadalupe, Nuevo Leon, Mexico, with circular distribution analysis.* Journal of the American Mosquito Control Association, 2003. **19**(1): p. 15-18.

941. Merdic, E., et al., *Mosquitoes of Istria, a contribution to the knowledge of Croatian mosquito fauna (Diptera, Culicidae).* Periodicum Biologorum, 2008. **110**(4): p. 351-360.

942. Merlin, M., et al., *[Evaluation of immunological and entomotological indices of yellow fever in Pointe-Noire, People's Republic of Congo].* Bulletin de la Societe de Pathologie Exotique et de ses Filiales, 1986. **79**(2): p. 199-206.

943. Merrill, S.A., F.B. Ramberg, and H.H. Hagedorn, *Phylogeography and population strucure of* Aedes Aegypti *in Arizona.* American Journal of Tropical Medicine and Hygiene, 2005. **72**(3): p. 304-310.

944. Meynard, J.B., et al., *First description of a dengue fever outbreak in the interior of French Guiana, February 2006.* European Journal of Public Health, 2009. **19**(2): p. 183-188.

945. Mi Mi, K. and T. khin Aye, *Isolation of dengue type 3 from mosquitoes in Rangoon.* Southeast Asian Journal of Tropical Medicine and Public Health, 1976. **7**(4): p. 507-512.

946. Miagostovich, M.P., et al., *Dengue epidemic in the state of Rio de Janeiro, Brazil: virological and epidemiological aspects.* Revista do Instituto de Medicina Tropical de Sao Paulo, 1993. **35**(2): p. 149-154.

947. Micieli, M.V. and R.E. Campos, *Oviposition activity and seasonal pattern of a population of* Aedes (Stegomyia) aegypti *(L.) (Diptera: Culicidae) in subtropical Argentina.* Memorias Do Instituto Oswaldo Cruz, 2003. **98**(5): p. 659-663.

948. Micieli, M.V., et al., *[Population dynamics of the immature stages of Aedes aegjpti (Diptera : Culicidae), vector of dengue: a longitudinal study (1996-2000).].* Revista de Biologia Tropical, 2006. **54**(3): p. 979-983.

949. Micks, D.W. and W.B. Moon, Aedes Aegypti *in a Texas coastal county as an index of dengue fever receptivity and control.* American Journal of Tropical Medicine and Hygiene, 1980. **29**(6): p. 1382-1388.

950. Midega, J.T., et al., *Application of the pupal/demographic-survey methodology to identify the key container habitats of* Aedes Aegypti *(L.) in Malindi district, Kenya.* Annals of Tropical Medicine and Parasitology, 2006. **100 Suppl 1**: p. S61-S72.

951. Milagres, M. *DENGUE/DHF UPDATE 2003 (31) [Brazil]*. 2003 [cited 2003 4 August]; Informativo noolhar, Sat 26 Jul 2003 (translated by Maria Jacobs,

ProMED-ESP) [edited].

952. Milagres, M. *DENGUE/DHF UPDATE 2003 (04)*. PROMED, 2003.

953. Miller, B.R., et al., *Isolation and genetic characterization of Rift Valley fever virus from* Aedes vexans arabiensis*, Kingdom of Saudi Arabia.* Emerging Infectious Dieseases, 2002. **8**(12): p. 1492-1494.

954. Miller, B.R., C.J. Mitchell, and M.E. Ballinger, *Replication, tissue tropisms and transmission of yellow fever virus in* Aedes albopictus*.* Transactions of the Royal Society of Tropical Medicine and Hygiene, 1989. **83**(2): p. 252-255.

955. Miller, B.R., et al., *Epidemic yellow fever caused by an incompetent mosquito vector.* Tropical Medicine and Parasitology, 1989. **40**(4): p. 396-399.

956. Mireji, P.O., et al., *Heavy metals in mosquito larval habitats in urban Kisumu and Malindi, Kenya, and their impact.* Ecotoxicology and Environmental Safety, 2008. **70**(1): p. 147-153.

957. Mitchell, C.J. and B.R. Miller, *Vertical transmission of dengue viruses by strains of* Aedes albopictus *recently introduced into Brazil.* Journal of the American Mosquito Control Association, 1990. **6**(2): p. 251-253.

958. Mitchell, C.J., et al., *Isolation of eastern equine encephalitis virus from* Aedes albopictus *in Florida.* Science, 1992. **257**(5069): p. 526-527.

959. Mitchell, C.J., et al., *Isolations of Potosi virus from mosquitoes collected in the United States, 1989-94.* Journal of the American Mosquito Control Association, 1996. **12**(1): p. 1-7.

960. Mitchell, C.J., G.C. Smith, and B.R. Miller, *Vector competence of* Aedes albopictus *for a newly recognized Bunyavirus from mosquitoes collected in Potosi, Missouri.* Journal of the American Mosquito Control Association, 1990. **6**(3): p. 523-527.

961. Mittal, P.K., T. Adak, and C.P. Batra, *Comparative toxicity of selected larvicidal formulations against Anopheles stephensi Liston and* Aedes Aegypti *Linn.* Journal of Communicable Diseases, 2001. **33**(2): p. 116-120.

962. Mogi, M., *Variation in Oviposition, Hatch Rate and Setal Morphology in Laboratory Strains of* Aedes-Albopictus*.* Mosquito News, 1982. **42**(2): p. 196-201.

963. Mogi, M., et al., *Applicability of presence-absence and sequential sampling for ovitrap surveillance of Aedes (Diptera: Culicidae) in Chiang Mai, northern Thailand.* Journal of Medical Entomology, 1990. **27**(4): p. 509-514.

964. Mogi, M., et al., *Scale pattern variations of* Aedes Aegypti *in Chiang Mai, northern Thailand.* Journal of the American Mosquito Control Association, 1989. **5**(4): p. 529-533.

965. Mogi, M., et al., *Ovitrap surveys of dengue vector mosquitoes in Chiang Mai, northern Thailand: seasonal shifts in relative abundance of* Aedes albopictus *and Ae. aegypti.* Medical and Veterinary Entomology, 1988. **2**(4): p. 319-324.

966. Mogi, M., et al., *Inter- and intraspecific variation in resistance to desiccation by adult Aedes (Stegomyia) spp. (Diptera: Culicidae) from Indonesia.* Journal of Medical Entomology, 1996. **33**(1): p. 53-57.

967. Mogi, M., et al., *Occurrence of* Aedes (Stegomyia) *spp. mosquitoes (Diptera: Culicidae) in Halmahela villages, Indonesia.* Journal of Medical Entomology, 1996. **33**(1): p. 169-172.

968. Mogi, M., et al., *Variation in Abdominal Color Pattern in 8 Populations of* Aedes-Aegypti *from the Philippines.* Mosquito News, 1984. **44**(1): p. 60-65.

969. Mogi, M. and N. Yamamura, *Estimation of the Attraction Range of a Human Bait for* Aedes-Albopictus *(Diptera, Culicidae) Adults and Its Absolute Density by a New Removal Method Applicable to Populations with Immigrants.* Researches on Population Ecology, 1981. **23**(2): p. 328-343.

970. Mohammed, A. and D.D. Chadee, *An evaluation of some Trinidadian plant extracts against larvae of* Aedes Aegypti *mosquitoes.* Journal of the American Mosquito Control Association, 2007. **23**(2): p. 172-176.

971. Moloney, J.M., et al., *Domestic* Aedes Aegypti *breeding site surveillance: limitations of remote sensing as a predictive surveillance tool.* American Journal of Tropical Medicine and Hygiene, 1998. **59**(2): p. 261-264.

972. Mondet, B., *[Yellow fever epidemiology in Brazil].* Bulletin de la Societe de Pathologie Exotique, 2001. **94**(3): p. 260-267.

973. Montada, D., A.R. Rajavel, and V. Vasuki, *Use of hexaflumuron, an insect growth regulator in the control of* Aedes albopictus *(Skuse).* Southeast Asian Journal of Tropical Medicine and Public Health, 1994. **25**(2): p. 374-377.

974. Montella, I.R., et al., *Insecticide Resistance Mechanisms of Brazilian* Aedes Aegypti *Populations from 2001 to 2004.* American Journal of Tropical Medicine and Hygiene, 2007. **77**(3): p. 467-477.

975. Monzon, R.B., et al., *Larvicidal potential of five Philippine plants against* Aedes Aegypti *(Linnaeus) and Culex quinquefasciatus (Say).* Southeast Asian Journal of Tropical Medicine and Public Health, 1994. **25**(4): p. 755-759.

976. Moore, C.G., *Insecticide Avoidance by Ovipositing Aedes-Aegypti.* Mosquito News, 1977. **37**(2): p. 291-293.

977. Moore, C.G., et al., Aedes Aegypti *in Puerto Rico: environmental determinants of larval abundance and relation to dengue virus transmission.* American Journal of Tropical Medicine and Hygiene, 1978. **27**(6): p. 1225-1231.

978. Moore, C.G., et al., Aedes albopictus *and other container-inhabiting mosquitoes in the United States: results of an eight-city survey.* Journal of the American Mosquito Control Association, 1990. **6**(2): p. 173-178.

979. Moore, C.G. and D.M. Whitacre, *Competition in Mosquitos .2. Production of* Aedes-Aegypti *Diptera-Culicidae Larval Growth Retardant at Various Densities and Nutrition Levels.* Annals of the Entomological Society of America, 1972. **65**(4): p. 915-918.

980. Moore, J.P., *New Nebraska mosquito distribution records.* Journal of the American Mosquito Control Association, 2001. **17**(4): p. 262-264.

981. Morales, A., et al., *Recovery of Dengue-2 Virus from* Aedes-Aegypti *in Colombia.* American Journal of Tropical Medicine and Hygiene, 1973. **22**(6): p. 785-787.

982. Morato, V.C.G., et al., *Infestation of* Aedes Aegypti *estimated by oviposition traps in Brazil.* Revista de Saude Publica, 2005. **39**(4): p. 553-558.

983. Morens, D.M., et al., *Dengue in Puerto Rico, 1977: public health response to characterize and control an epidemic of multiple serotypes.* American Journal of Tropical Medicine and Hygiene, 1986. **35**(1): p. 197-211.

984. Morrison, A.C., et al., *Evaluation of a sampling methodology for rapid assessment of* Aedes Aegypti *infestation levels in Iquitos, Peru.* Journal of Medical Entomology, 2004. **41**(3): p. 502-510.

985. Morrison, A.C., et al., *Increased fecundity of* Aedes Aegypti *fed human blood before release in a mark-recapture study in Puerto Rico.* Journal of the American Mosquito Control Association, 1999. **15**(2): p. 98-104.

986. Morrison, A.C., et al., *Temporal and geographic patterns of* Aedes Aegypti *(Diptera : Culicidae) production in Iquitos, Peru.* Journal of Medical Entomology, 2004. **41**(6): p. 1123-1142.

987. Morrison, A.C., et al., Aedes Aegypti *(Diptera: Culicidae) production from non-residential sites in the Amazonian city of Iquitos, Peru.* Annals of Tropical Medicine and Parasitology, 2006. **100 Suppl 1**: p. S73-S86.

988. Morvan, J., et al., *Rift Valley fever on the east coast of Madagascar.* Research in Virology, 1991. **142**(6): p. 475-482.

989. Mou, R., H.E. Bao, and J.H. Li, *[Change of lipid, esterase and lipase in mosquito larvae infected with Lagenidium giganteum].* Chinese Journal of Parasitology & Parasitic Diseases - Zhongguo Ji Sheng Chong Xue Yu Ji Sheng Chong Bing Za Zhi, 2004. **22**(5): p. 280-282.

990. Mouchet, J., *[*Aedes-Aegypti *and Potential Carriers of Yellow-Fever in Democratic Republic of Somalia and in French Territory of Afars and Issas].* Bulletin of the World Health Organization, 1971. **45**(3): p. 383-394.

991. Mourya, D.T., M.D. Gokhale, and A.C. Mishra, *Biochemical basis of DDT-resistance in* Aedes Aegypti *population from a dengue affected area in Shahjahanpur city.* Indian Journal of Medical Research, 1994. **99**: p. 212-215.

992. Mourya, D.T., et al., *Isolation of Chikungunya virus from* Aedes Aegypti *mosquitoes collected in the town of Yawat, Pune District, Maharashtra State, India.* Acta Virologica, 2001. **45**(5-6): p. 305-309.

993. Mousson, L., et al., *Phylogeography of* Aedes (Stegomyia) aegypti *(L.) and* Aedes (Stegomyia) albopictus *(Skuse) (Diptera: Culicidae) based on mitochondrial DNA variations.* Genetic Research, 2005. **86**(1): p. 1-11.

994. Mousson, L., et al., *Genetic structure of* Aedes Aegypti *populations in Chiang Mai (Thailand) and relation with dengue transmission.* Tropical Medicine & International Health, 2002. **7**(10): p. 865-872.

995. Munstermann, L.E. and D.M. Wesson, *First record of Ascogregarina taiwanensis (Apicomplexa: Lecudinidae) in North American* Aedes albopictus*.* Journal of the American Mosquito Control Association, 1990. **6**(2): p. 235-243.

996. Murrell, E.G. and S.A. Juliano, *Detritus type alters the outcome of interspecific competition between* Aedes Aegypti *and* Aedes albopictus *(Diptera : Culicidae).* Journal of Medical Entomology, 2008. **45**(3): p. 375-383.

997. Mwangangi, J., et al., *Dynamics of immature stages of* Anopheles *arabiensis and other mosquito species (Diptera : Culicidae) in relation to rice cropping in a rice agro-ecosystem in Kenya.* Journal of Vector Ecology, 2006. **31**(2): p. 245-251.

998. Nakhapakorn, K. and N.K. Tripathi, *An information value based analysis of physical and climatic factors affecting dengue fever and dengue haemorrhagic fever incidence.* International journal of health geographics 2005. **4**: p. 13.

999. Nalim, S., et al., *Studies on the susceptibility of a large urban population of* Aedes Aegypti *to infection with dengue viruses.* Southeast Asian Journal of Tropical Medicine and Public Health, 1978. **9**(4): p. 494-500.

1000. Nam, V.S., et al., *Eradication of* Aedes Aegypti *from a village in Vietnam, using copepods and community participation.* American Journal of Tropical Medicine and Hygiene, 1998. **59**(4): p. 657-660.

1001. Nart, P. *DENGUE/DHF UPDATES (20): 28 NOV 2001 [Panama]*. 2001 [cited 2001 29 November]; Agencia EFE (via COMTEX), Wed 21 Nov 2001 [edited].

1002. Nart, P. *DENGUE/DHF UPDATES (22): 10 JUN 2002 [El Salvador]*. 2002 [cited 2002 10 June]; Agence France Press, via Newsline, Tue 4 Jun 2002 [edited].

1003. Nart, P. *DENGUE/DHF UPDATES (23): 17 JUN 2002 [Cambodia]*. 2002 [cited 2002 25 June]; Xinhua News Agency, Tue 11 June 2002 [edited].

1004. Nart, P. *DENGUE/DHF UPDATES (41): 21 OCT 2002* PROMED, 2002.

1005. Nart, P. AEDES ALBOPICTUS *- NICARAGUA* PROMED, 2003.

1006. Nart, P. *DENGUE/DHF UPDATE 2003 (15)* PROMED, 2003.

1007. Nart, P. AEDES ALBOPICTUS *- SPAIN* PROMED, 2004.

1008. Nasci, R.S., *The size of emerging and host-seeking* Aedes Aegypti *and the relation of size to blood-feeding success in the field.* Journal of the American Mosquito Control Association, 1986. **2**(1): p. 61-62.

1009. Nasci, R.S., G.B. Wright, and F.S. Willis, *Control of* Aedes albopictus *larvae using time-release larvicide formulations in Louisiana.* Journal of the American Mosquito Control Association, 1994. **10**(1): p. 1-6.

1010. Natal, D., et al., *[*Aedes (Stegomyia) albopictus *(Skuse) breeding in Bromeliaceae in the outskirts of an urban area of the city of Sao Paulo, Brazil].* Revista de Saúde Pública, 1997. **31**(5): p. 517-518.

1011. Nathan, N., et al., *Shortage of vaccines during a yellow fever outbreak in Guinea.* Lancet, 2001. **358**(9299): p. 2129-2130.

1012. Nathin, M.A., S.R. Harun, and Sumarmo, *Dengue haemorrhagic fever and Japanese B encephalitis in Indonesia.* Southeast Asian Journal of Tropical Medicine and Public Health, 1988. **19**(3): p. 475-481.

1013. Nayar, J.K., et al., *Mosquito hosts of arboviruses from Indian River County, Florida, during 1998.* Florida Entomologist, 2001. **84**(3): p. 376-379.

1014. Nayar, J.K. and J.W. Knight, Aedes albopictus *(Diptera : Culicidae): an experimental and natural host of* Dirofilaria immitis *(Filarioidea : Onchocercidae) in Florida, USA.* Journal of Medical Entomology, 1999. **36**(4): p. 441-448.

1015. Neff, J.M., et al., *Dengue fever in a Puerto Rican community.* American Journal of Epidemiology, 1967. **86**(1): p. 162-184.

1016. Nelson, M.J., et al., *Observations on the breeding habitats of* Aedes Aegypti *(L.) in Jakarta, Indonesia.* Southeast Asian Journal of Tropical Medicine and Public Health, 1976. **7**(3): p. 424-429.

1017. Nelson, M.J., et al., *Diurnal Periodicity of Attraction to Human Bait of* Aedes-Aegypti *(Diptera Culicidae) in Jakarta, Indonesia.* Journal of Medical Entomology, 1978. **14**(5): p. 504-510.

1018. Neng, W., et al., *Susceptibility of* Aedes albopictus *from China to insecticides, and mechanism of DDT resistance.* Journal of the American Mosquito Control Association, 1992. **8**(4): p. 394-397.

1019. Neto, P.L. and M.A. Navarro-Silva, *Development, longevity, gonotrophic cycle and oviposition of* Aedes albopictus *Skuse (Diptera : Culicidae) under cyclic temperatures.* Neotropical Entomology, 2004. **33**(1): p. 29-33.

1020. Neves-Souza, P.C.F., et al., *Inducible nitric oxide synthase (iNOS) expression in monocytes during acute Dengue Fever in patients and during in vitro infection.* Bmc Infectious Diseases, 2005. **5**: p. -.

1021. Newton, M.E., R.J. Wood, and D.I. Southern, *Cytological Mapping of M and D Loci in Mosquito,* Aedes-Aegypti *(L).* Genetica, 1978. **48**(2): p. 137-143.

1022. Niebylski, M.L. and G.B. Craig, Jr., *Dispersal and survival of* Aedes albopictus *at a scrap tire yard in Missouri.* Journal of the American Mosquito Control Association, 1994. **10**(3): p. 339-343.

1023. Niebylski, M.L., et al., *Blood hosts of* Aedes albopictus *in the United States.* Journal of the American Mosquito Control Association, 1994. **10**(3): p. 447-450.

1024. Nino, L., *[Use of the function semivariogram and kriging estimation in the spacial analysis of* Aedes Aegypti *(Diptera: Culicidae) distributions].* Biomedica, 2008. **28**(4): p. 578-586.

1025. Nisha, V., et al., *Geographical information system (GIS) in investigation of an outbreak.* Journal of Communicable Diseases, 2005. **37**(1): p. 39-43.

1026. Nogueira, L.A., et al., *Short report: Application of an alternative Aedes species (Diptera : Culicidae) surveillance method in Botucatu City, Sao Paulo, Brazil.* American Journal of Tropical Medicine and Hygiene, 2005. **73**(2): p. 309-311.

1027. Nogueira, R.M., et al., *Dengue type 2 outbreak in the south of the state of Bahia, Brazil: laboratorial and epidemiological studies.* Revista do Instituto de Medicina Tropical de Sao Paulo, 1995. **37**(6): p. 507-510.

1028. Nogueira, R.M., et al., *Virological study of a dengue type 1 epidemic at Rio de Janeiro.* Memorias do Instituto Oswaldo Cruz, 1988. **83**(2): p. 219-225.

1029. Nogueira, R.M., et al., *Virological study of a dengue type 1 epidemic at Rio de Janeiro.* Memorias Do Instituto Oswaldo Cruz, 1988. **83**(2): p. 219-225.

1030. Noridah, O., et al., *Outbreak of chikungunya due to virus of Central/East African genotype in Malaysia.* Medical Journal of Malaysia, 2007. **62**(4): p. 323-328.

1031. Novak, R.J., *A North American model to contain the spread of* Aedes albopictus *through tire legislation.* Parassitologia, 1995. **37**(2-3): p. 129-139.

1032. Novak, R.J., et al., *Penetration rate of two pesticide carriers at a large used-tire storage facility in Chicago, Illinois.* Journal of the American Mosquito Control Association, 1990. **6**(2): p. 188-196.

1033. Nwoke, B.E., et al., *Sustainable urban development and human health: septic tank as a major breeding habitat of mosquito vectors of human diseases in south-eastern Nigeria.* Applied Parasitology, 1993. **34**(1): p. 1-10.

1034. O'Leary, D.R., et al., *Assessment of dengue risk in relief workers in Puerto Rico after Hurricane Georges, 1998.* American Journal of Tropical Medicine and Hygiene, 2002. **66**(1): p. 35-39.

1035. O'Meara, G.F., M.M. Cutwa, and L.F. Evans, Jr., *Bromeliad-inhabiting mosquitoes in south Florida: native and exotic plants differ in species composition.* Journal of Vector Ecology 2003. **28**(1): p. 37-46.

1036. O'Meara, G.F., et al., *Invasion of cemeteries in Florida by* Aedes albopictus*.* Journal of the American Mosquito Control Association, 1992. **8**(1): p. 1-10.

1037. Obando, R.G., et al., *[Experience of an entomological analysis of the breeding sites of* Aedes Aegypti *and* Culex quinquefasciatus *in Cali, Colombia].* Revista Colombiana De Entomologia, 2007. **33**(2): p. 148-156.

1038. Ocampo, C.B. and D.M. Wesson, *Population dynamics of* Aedes Aegypti *from a dengue hyperendemic urban setting in Colombia.* American Journal of Tropical Medicine and Hygiene, 2004. **71**(4): p. 506-513.

1039. Ogata, K. and A.L. Samayoa, *Discovery of* Aedes albopictus *in Guatemala.* Journal of the American Mosquito Control Association, 1996. **12**(3): p. 503-506.

1040. Omardeen, T.A., *Susceptibility Tests in Trinidad with Anopheles Aquasalis Curry,* Aedes Aegypti *(L) and* Culex Pipiens *Fatigans Wiedemann, Using Standard Who Mosquito Adult Test Kit.* Bulletin of the World Health Organization, 1961. **24**(4-5): p. 495-507.

1041. OMeara, G.F., et al., *Exotic tank bromeliads harboring immature* Aedes albopictus *and* Aedes bahamensis *(Diptera: Culicidae) in Florida.* Journal of Vector Ecology, 1995. **20**(2): p. 216-224.

1042. OMeara, G.F., L.F. Evans, and M.L. Womack, *Colonization of rock holes by* Aedes albopictus *in the southeastern United States.* Journal of the American Mosquito Control Association, 1997. **13**(3): p. 270-274.

1043. Orta-Pesina, H., R. Mercado-Hernandez, and J.F. Elizondo-Leal, *[Distribution of* Aedes albopictus *(Skuse) in Nuevo Leon, Mexico, 2001-2004.].* Salud Pública de México, 2005. **47**(2): p. 163-165.

1044. Ortiz, D.I., W.L. Kang, and S.C. Weaver, *Susceptibility of* Ae. aegypti *(Diptera: Culicidae) to Infection with Epidemic (Subtype IC) and Enzootic (Subtypes ID, IIIC, IIID) Venezuelan Equine Encephalitis Complex Alphaviruses.* Journal of Medical Entomology, 2008. **45**(6): p. 1117-1125.

1045. Otero, M., H.G. Solari, and N. Schweigmann, *A stochastic population dynamics model for* Aedes aegypti*: formulation and application to a city with temperate climate.* Bulletin of Mathematical Biology, 2006. **68**(8): p. 1945-1974.

1046. OwusuDaaku, K.O., R.J. Wood, and R.D. Butler, *Selected lines of* Aedes Aegypti *with persistently distorted sex ratios.* Heredity, 1997. **79**: p. 388-393.

1047. Padbidri, V.S., et al., *The 1993 epidemic of dengue fever in Mangalore, Karnataka state, India.* Southeast Asian Journal of Tropical Medicine and Public Health, 1995. **26**(4): p. 699-704.

1048. Padbidri, V.S., et al., *The 1993 epidemic of dengue fever in Mangalore, Karnataka state, India.* Southeast Asian Journal of Tropical Medicine and Public Health, 1995. **26**(4): p. 699-704.

1049. Paduan, K.D., J.P. Araujo, and P.E.M. Ribolla, *Genetic variability in geographical populations of* Aedes Aegypti *(Diptera, Culicidae) in Brazil elucidated by molecular markers.* Genetics and Molecular Biology, 2006. **29**(2): p. 391-395.

1050. Paduan, K.D. and P.E.M. Ribolla, *Mitochondrial DNA polymorphism and heteroplasmy in populations of* Aedes Aegypti *in Brazil.* Journal of Medical Entomology, 2008. **45**(1): p. 59-67.

1051. Paeporn, P., et al., *Temephos resistance in two forms of* Aedes Aegypti *and its significance for the resistance mechanism.* Southeast Asian Journal of Tropical Medicine and Public Health, 2003. **34**(4): p. 786-792.

1052. Paeporn, P., et al., *Biochemical detection of pyrethroid resistance mechanism in* Aedes Aegypti *in Ratchaburi province, Thailand.* Tropical Biomedicine, 2004. **21**(2): p. 145-151.

1053. Paeporn, P., et al., *Insecticide susceptibility and selection for resistance in a population of* Aedes Aegypti *from Ratchaburi province, Thailand.* Tropical Biomedicine, 2004. **21**(2): p. 1-6.

1054. Page, E. *CHIKUNGUNYA - AFRICA (GABON) (03)*. PROMED, 2007.

1055. Pages, F., et al., Aedes albopictus *mosquito: the main vector of the 2007 Chikungunya outbreak in Gabon.* PLoS One, 2009. **4**(3): p. e4691.

1056. Paige, C.J. and G.B. Craig, *Variation in Filarial Susceptibility among East-African Populations of Aedes-Aegypti.* Journal of Medical Entomology, 1975. **12**(5): p. 485-493.

1057. Paily, K.P., et al., *Infectivity of a mermithid nematode Romanomermis iyengari (Welch) in different conductivity levels under laboratory and field conditions.* Indian Journal of Experimental Biology, 1991. **29**(6): p. 579-581.

1058. Panagos, A., et al., *Dengue in Grenada.* Revista Panamericana de Salud Publica - Pan American Journal of Public Health, 2005. **17**(4): p. 225-229.

1059. Pandey, B.D., et al., *First case of Dengue virus infection in Nepal.* Nepal Medical College journal 2004. **6**(2): p. 157-9.

1060. Pandian, R.S. and S.K. Dwarakanath, *The Biting Activity Rhythm in* Aedini *Mosquitos of Madurai.* Comparative Physiology and Ecology, 1992. **17**(2): p. 66-70.

1061. Panicker, K.N., M.G. Bai, and M. Kalyanasundaram, *Well Breeding-Behavior of* Aedes-Aegypti*.* Indian Journal of Medical Research, 1982. **76**(Nov): p. 689-691.

1062. Pant, C.P., et al., *Large-Scale Field Trial of Ultralow-Volume Fenitrothion Applied by a Portable Mist Blower for Control of* Aedes-Aegypti*.* Bulletin of the World Health Organization, 1974. **51**(4): p. 409-415.

1063. Pant, C.P., M.J. Nelson, and H.L. Mathis, *Sequential Application of Ultralow-Volume Ground Aerosols of Fenitrothion for Sustained Control of Aedes-Aegypti.* Bulletin of the World Health Organization, 1973. **48**(4): p. 455-459.

1064. Pant, C.P. and M. Yasuno, *Field Studies on Gonotropic Cycle of* Aedes-Aegypti *in Bangkok, Thailand.* Journal of Medical Entomology, 1973. **10**(2): p. 219-223.

1065. Paramasivan, R., et al., *Seroepidemiology of a focal outbreak of dengue in Tamil Nadu.* Indian Journal of Medical Research, 2006. **124**(6): p. 718-720.

1066. Parc, F., et al., *[Dengue outbreak by virus type 4 in French Polynesia. I. General epidemiology - clinical specific aspects (author's transl)].* Medecine Tropicale, 1981. **41**(1): p. 93-96.

1067. Parker, A.G., et al., *Rock Hole Habitats of a Feral Population of* Aedes-Aegypti *on the Island of Anguilla, West-Indies.* Mosquito News, 1983. **43**(1): p. 79-81.

1068. Passos, A.D., E.M. Rodrigues, and A.L. Dal-Fabbro, *Dengue control in Ribeirao Preto, Sao Paulo, Brazil.* Cadernos de Saúde Pública, 1998. **14 Suppl 2**: p. 123-128.

1069. Paupy, C., et al., *Gene flow between domestic and sylvan populations of* Aedes Aegypti *(Diptera : Culicidae) in North Cameroon.* Journal of Medical Entomology, 2008. **45**(3): p. 391-400.

1070. Paupy, C., et al., *Influence of breeding sites features on genetic differentiation of* Aedes Aegypti *populations analyzed on a local scale in Phnom Penh Municipality of Cambodia.* American Journal of Tropical Medicine and Hygiene, 2004. **71**(1): p. 73-81.

1071. Paupy, C., et al., *Factors influencing the population structure of* Aedes Aegypti *from the main cities in Cambodia.* Heredity, 2005. **95**(2): p. 144-147.

1072. Paupy, C., et al., *Variation over space and time of* Aedes Aegypti *in Phnom Penh (Cambodia): genetic structure and oral susceptibility to a dengue virus.* Genetical Research, 2003. **82**(3): p. 171-182.

1073. Paupy, C., et al., *Variation over space and time of* Aedes Aegypti *in Phnom Penh (Cambodia): genetic structure and oral susceptibility to a dengue virus.* Genetic Research, 2003. **82**(3): p. 171-182.

1074. Paupy, C., et al., *Population structure of* Aedes albopictus *from La Reunion Island (Indian Ocean) with respect to susceptibility to a dengue virus.* Heredity, 2001. **87**: p. 273-283.

1075. Paupy, C., et al., *Comparisons of amplified fragment length polymorphism (AFLP), microsatellite, and isoenzyme markers: population genetics of* Aedes Aegypti *(Diptera: Culicidae) from Phnom Penh (Cambodia).* Journal of Medical Entomology, 2004. **41**(4): p. 664-671.

1076. Paupy, C., et al., Aedes Aegypti *in Tahiti and Moorea (French Polynesia): isoenzyme differentiation in the mosquito population according to human population density.* American Journal of Tropical Medicine and Hygiene, 2000. **62**(2): p. 217-224.

1077. Pavan, W. *CHIKUNGUNYA - ITALY (EMILIA ROMAGNA): SUSPECTED* PROMED, 2006.

1078. Pearson, A.M. and R.J. Wood, *Combining the Meiotic Drive Gene-D and the Translocation-T1 in the Mosquito,* Aedes-Aegypti *(L) .1. Sex-Ratio Distortion and Fertility.* Genetica, 1980. **51**(3): p. 203-210.

1079. Pelaez, O., et al., *Dengue 3 epidemic, Havana, 2001.* Emerging Infectious Dieseases, 2004. **10**(4): p. 719-722.

1080. Pena, C.J., G. Gonzalvez, and D.D. Chadee, *Seasonal prevalence and container preferences of* Aedes albopictus *in Santo Domingo City, Dominican Republic.* Journal of Vector Ecology 2003. **28**(2): p. 208-212.

1081. Pener, H. AEDES ALBOPICTUS *- ISRAEL: FIRST RECORD* PROMED, 2002.

1082. Penersalomon, H. and A. Vardi, *Reoccurrence of* Aedes-Aegypti *(Insecta-Diptera-Culicidae) in Israel.* Israel Journal of Zoology, 1975. **24**(3-4): p. 193-193.

1083. Peng, Y.F., et al., *Field evaluations of Romanomermis yunanensis (Nematoda : Mermithidae) for control of Culicinae mosquitoes in China.* Fundamental and Applied Nematology, 1998. **21**(3): p. 227-232.

1084. Pereira, E.D., et al., *Trichomycete fungi (Zygomycota) associated with mosquito larvae (Diptera : Culicidae) in natural and artificial habitats in Manaus, AM Brazil.* Neotropical Entomology, 2005. **34**(2): p. 325-329.

1085. Perez, C.M., et al., *Spinosad, a naturally derived insecticide, for control of* Aedes Aegypti *(Diptera: Culicidae): efficacy, persistence, and elicited oviposition response.* Journal of Medical Entomology, 2007. **44**(4): p. 631-638.

1086. Perez, D., et al., *Community participation in* Aedes Aegypti *control: a sociological perspective on five years of research in the health area ''26 de Julio'', Havana, Cuba.* Tropical Medicine & International Health, 2007. **12**(5): p. 664-672.

1087. Perez Diaz, R. and O. Fuentes Gonzalez, *[Analysis of the surveillance system of larval traps in the municipality of Mariel (1984-1987)].* Revista Cubana de Medicina Tropical, 1990. **42**(2): p. 254-260.

1088. Perez-Pacheco, R., et al., *Parasitism of* Romanomermis iyengari *in larvae of three species of mosquito in laboratory, and of* Anopheles pseudopunctipennis *in the field.* Agrociencia, 2004. **38**(4): p. 413-421.

1089. Perich, M.J., et al., *Penetration of ultra-low volume applied insecticide into dwellings for dengue vector control.* Journal of the American Mosquito Control Association, 1992. **8**(2): p. 137-142.

1090. Perich, M.J., et al., *Behavior of resting* Aedes Aegypti *(Culicidae: Diptera) and its relation to ultra-low volume adulticide efficacy in Panama City, Panama.* Journal of Medical Entomology, 2000. **37**(4): p. 541-546.

1091. Perich, M.J., et al., *Field evaluation of a lethal ovitrap against dengue vectors in Brazil.* Medical and Veterinary Entomology, 2003. **17**(2): p. 205-210.

1092. Perich, M.J., et al., *Evaluation of the efficacy of lambda-cyhalothrin applied by three spray application methods for emergency control of* Aedes Aegypti *in Costa Rica.* Journal of the American Mosquito Control Association, 2003. **19**(1): p. 58-62.

1093. Perich, M.J., et al., *Evaluation of the efficacy of lambda-cyhalothrin applied as ultra-low volume and thermal fog for emergency control of* Aedes Aegypti *in Honduras.* Journal of the American Mosquito Control Association, 2001. **17**(4): p. 221-224.

1094. Perich, M.J., et al., *Comparison of ground and aerial ultra-low volume applications of malathion against* Aedes Aegypti *in Santo Domingo, Dominican Republic.* Journal of the American Mosquito Control Association, 1990. **6**(1): p. 1-6.

1095. Pesina, H.O., R.M. Hernandez, and M.A.V. Rodriguez, Aedes albopictus *in Allende City, Nuevo Leon, Mexico.* Journal of the American Mosquito Control Association, 2001. **17**(4): p. 260-261.

1096. Petersen, J.L., L.P. Lounibos, and N. Lorimer, *Field Trials of Double Translocation Heterozygote Males for Genetic-Control of* Aedes-Aegypti *(L) (Diptera-Culicidae).* Bulletin of Entomological Research, 1977. **67**(2): p. 313-324.

1097. Pethuan, S., et al., *Biochemical studies of insecticide resistance in* Aedes (Stegomyia) aegypti *and* Aedes (Stegomyia) albopictus *(Diptera: Culicidae) in Thailand.* Tropical Biomedicine, 2007. **24**(1): p. 7-15.

1098. Phillips, I., et al., *First documented outbreak of dengue in the Peruvian Amazon region.* Bulletin of the Pan American Health Organization, 1992. **26**(3): p. 201-207.

1099. Phuanukoonnon, S., I. Mueller, and J.H. Bryan, *Effectiveness of dengue control practices in household water containers in Northeast Thailand.* Tropical Medicine & International Health, 2005. **10**(8): p. 755-763.

1100. Pillai, J.S. and I.M. Rakai, *Mosquito-Borne Infections in Fiji .6. Diel Periodicity in Landing of* Aedes-Aegypti *on Man.* Mosquito News, 1976. **36**(2): p. 186-189.

1101. Pillai, J.S. and J. Urdang, *Discovery of the Mosquito* Aedes-Aegypti *on Tokelau Group.* New Zealand Medical Journal, 1979. **90**(643): p. 212-213.

1102. Pinheiro, V.C. and W.P. Tadei, *Evaluation of the residual effect of temephos on* Aedes Aegypti *(Diptera, Culicidae) larvae in artificial containers in Manaus, Amazonas State, Brazil.* Cadernos de Saúde Pública, 2002. **18**(6): p. 1529-1536.

1103. Pinheiro, V.C. and W.P. Tadei, *Frequency, diversity, and productivity study on the* Aedes Aegypti *most preferred containers in the city of Manaus, Amazonas, Brazil.* Revista do Instituto de Medicina Tropical de São Paulo, 2002. **44**(5): p. 245-250.

1104. Pinheiro, V.C.S., et al., *Detection of dengue virus serotype 3 by reverse transcription-polymerase chain reaction in* Aedes Aegypti *(Diptera, Culicidae) captured in Manaus, Amazonas.* Memorias do Instituto Oswaldo Cruz, 2005. **100**(8): p. 833-839.

1105. Pinheiro, V.C.S., et al., *Detection of dengue virus serotype 3 by reverse transcription-polymerase chain reaction in* Aedes Aegypti *(Diptera, Culicidae) captured in Manaus, Amazonas.* Memorias Do Instituto Oswaldo Cruz, 2005. **100**(8): p. 833-839.

1106. Pisano, M.R., J. Nicoli, and H. Tolou, *Homogeneity of yellow fever virus strains isolated during an epidemic and a post-epidemic period in West Africa.* Virus Genes, 1997. **14**(3): p. 225-234.

1107. Pollack, M. *DENGUE/DHF - COSTA RICA (02)*. PROMED, 1999.

1108. Pollack, M.P. *DENGUE/DHF - COSTA RICA (04)*. 1999 [cited 1999 21 August]; La Nacion, San Jose, Costa Rica Thu 19 Aug 1999 [edited].

1109. Pollack, M.P. *DENGUE/DHF - SRI LANKA (02)*. 1999 [cited 1999 9 August]; Sunday Observer (Sri Lanka), 8 Aug 1999 [edited].

1110. Pollack, M.P. *DENGUE/DHF - COSTA RICA (02)*. 1999 [cited 1999 11 July]; La Nacion, San Jose, Costa Rica, 9 Jul 1999.

1111. Pollack, M.P. *DENGUE/DHF - CENTRAL AMERICA (02)*. 2000 [cited 2000 24 August]; Agencia EFE (vua COMTEX): filed: Fri Aug 18, 2000 5:51 PM EST [edited].

1112. Polsomboon, S., et al., *Behavioral responses of catnip (Nepeta cataria) by two species of mosquitoes,* Aedes Aegypti *and* Anopheles harrisoni*, in Thailand.* Journal of the American Mosquito Control Association, 2008. **24**(4): p. 513-519.

1113. Polsomboon, S., et al., *Effects of physiological conditioning on behavioral avoidance by using a single age group of* Aedes Aegypti *exposed to deltamethrin and DDT.* Journal of Medical Entomology, 2008. **45**(2): p. 251-259.

1114. Pombi, M., C. Costantini, and A. della Torre, *[*Aedes albopictus *(Diptera: Culicidae) in Rome: experimental study of relevant control strategy parameters].* Parassitologia, 2003. **45**(2): p. 97-102.

1115. Ponce, G., et al., *Laboratory evaluation of Vectobac (R) as against* Aedes Aegypti *in Monterrey, Nuevo Leon, Mexico.* Journal of the American Mosquito Control Association, 2002. **18**(4): p. 341-343.

1116. Ponlanwat, A. and L.C. Harrington, *Blood feeding patterns of* Aedes Aegypti *and* Aedes albopictus *in Thailand.* Journal of Medical Entomology, 2005. **42**(5): p. 844-849.

1117. Ponlawat, A. and L.C. Harrington, *Factors associated with male mating success of the dengue vector mosquito,* Aedes aegypti*.* American Journal of Tropical Medicine and Hygiene, 2009. **80**(3): p. 395-400.

1118. Ponlawat, A., J.G. Scott, and L.C. Harrington, *Insecticide susceptibility of* Aedes Aegypti *and* Aedes albopictus *across Thailand.* Journal of Medical Entomology, 2005. **42**(5): p. 821-825.

1119. Pontes, R.J., et al., *[Dengue epidemic in Ribeirao Preto, SP, Brazil: a preliminary note].* Revista de Saude Publica, 1991. **25**(4): p. 315-317.

1120. Pontes, R.J.S., et al., *Vector densities that potentiate Dengue outbreaks in a Brazilian city.* American Journal of Tropical Medicine and Hygiene, 2000. **62**(3): p. 378-383.

1121. Powell, J.R., W.J. Tabachnick, and J. Arnold, *Genetics and the origin of a vector population: Aedes aegypti, a case study.* Science, 1980. **208**(4450): p. 1385-1387.

1122. Powers, N.R., et al., *The reintroduction and possible establishment of* Aedes albopictus *in New Mexico.* Journal of the American Mosquito Control Association, 2006. **22**(4): p. 756-757.

1123. Pramanik, M.K., G. Aditya, and S.K. Raut, *Seasonal prevalence of* Aedes Aegypti *immatures in Kolkata, India.* Southeast Asian Journal of Tropical Medicine and Public Health, 2007. **38**(3): p. 442-447.

1124. Preslar, D. *MYSTERY DISEASE - NICARAGUA* PROMED, 1995.

1125. Putnam, J.L., G.G. Clark, and T.W. Scott, *Failure of immune sera to neutralize dengue-2 virus in intrathoracically inoculated* Aedes aegypti*.* Journal of the American Mosquito Control Association, 1995. **11**(3): p. 372-374.

1126. Putnam, J.L. and T.W. Scott, *Blood-feeding behavior of dengue-2 virus-infected Aedes aegypti.* American Journal of Tropical Medicine and Hygiene, 1995. **52**(3): p. 225-227.

1127. Qiu, F.X., et al., *The first epidemic of dengue hemorrhagic fever in the People's Republic of China.* American Journal of Tropical Medicine and Hygiene, 1991. **44**(4): p. 364-370.

1128. Qiu, F.X., et al., *The first epidemic of dengue hemorrhagic fever in the People's Republic of China.* American Journal of Tropical Medicine and Hygiene, 1991. **44**(4): p. 364-370.

1129. Qiu, F.X., et al., *Dengue in China: a clinical review.* Bulletin of the World Health Organization, 1993. **71**(3-4): p. 349-359.

1130. Qualls, W.A. and G.R. Mullen, *Evaluation of the Mosquito Magnet Pro trap with and without 1-octen-3-ol for collecting* Aedes albopictus *and other urban mosquitoes.* Journal of the American Mosquito Control Association, 2007. **23**(2): p. 131-136.

1131. Quintero, J., et al., *An ecosystemic approach to evaluating ecological, socioeconomic and group dynamics affecting the prevalence of* Aedes Aegypti *in two Colombian towns.* Cadernos de Saúde Pública, 2009. **25 Suppl 1**: p. S93-103.

1132. Quiroz, E., et al., *[Dengue in Panama, 1993].* Revista Cubana de Medicina Tropical, 1997. **49**(2): p. 86-93.

1133. Rafael, M.S., et al., *Potential control of* Aedes Aegypti *(Diptera: Culicidae) with* Piper aduncum *L. (Piperaceae) extracts demonstrated by chromosomal biomarkers and toxic effects on interphase nuclei.* Genetics and Molecular Research, 2008. **7**(3): p. 772-781.

1134. Raineri, V., et al., *[Further data on the spread to Genoa of* Aedes albopictus*].* Parassitologia, 1991. **33**(2-3): p. 183-185.

1135. Rajatileka, S., et al., *Development and application of a simple colorimetric assay reveals widespread distribution of sodium channel mutations in Thai populations of Aedes aegypti.* Acta Tropica, 2008. **108**(1): p. 54-57.

1136. Rajavel, A.R., R. Natarajan, and K. Vaidyanathan, *Mosquitoes of the mangrove forests of India: part six--Kundapur, Karnataka and Kannur, Kerala.* Journal of the American Mosquito Control Association, 2006. **22**(4): p. 582-585.

1137. Rajendram, G.F. and N.R. Antony, *Survey of peridomestic mosquito species of Jaffna peninsula in Sri Lanka.* Southeast Asian Journal of Tropical Medicine and Public Health, 1991. **22**(4): p. 637-642.

1138. Ramos, M.M., et al., *Epidemic dengue and dengue hemorrhagic fever at the Texas-Mexico border: Results of a household-based seroepiderniologic survey, December 2005.* American Journal of Tropical Medicine and Hygiene, 2008. **78**(3): p. 364-369.

1139. Rao, T.R., Pannicke.Kn, and R. Reuben, *Tree-Hole Breeding of* Aedes-Aegypti *in Southern India - a Preliminary Report.* Bulletin of the World Health Organization, 1970. **42**(2): p. 333-334.

1140. Rao, T.R., S.D. Paul, and K.R.P. Singh, *Experimental Studies on Mechanical Transmission of Chikungunya Virus by Aedes Aegypti.* Mosquito News, 1968. **28**(3): p. 406-408.

1141. Rao, T.R., et al., *Breeding Places and Seasonal Incidence of Aedes-Aegypti, as Assessed by Single-Larva Survey Method.* Bulletin of the World Health Organization, 1973. **48**(5): p. 615-622.

1142. Ratsitorahina, M., et al., *Outbreak of dengue and chikungunya fevers, Toamasina, Madagascar, 2006.* Emerging Infectious Diseases, 2008. **14**(7): p. 1135-1137.

1143. Ravel, S., et al., *Microsatellite markers for population genetic studies in* Aedes Aegypti *(Diptera: Culicidae) from Cote d'Ivoire: evidence for a microgeographic genetic differentiation of mosquitoes from Bouake.* Acta Tropica, 2002. **82**(1): p. 39-49.

1144. Ravel, S., et al., *A preliminary study of the population genetics of* Aedes Aegypti *(Diptera : Culicidae) from Mexico using microsatellite and AFLP markers.* Acta Tropica, 2001. **78**(3): p. 241-250.

1145. Rawlings, J. *DENGUE - U.S./MEXICO BORDER, 1995-1996*. 1996 [cited 1996 5 October].

1146. Rawlins, S.C., G.G. Clark, and R. Martinez, *Effects of single introduction of Toxorhynchites moctezuma upon* Aedes Aegypti *on a Caribbean Island.* Journal of the American Mosquito Control Association, 1991. **7**(1): p. 7-10.

1147. Rawlins, S.C., et al., *Evaluation of Caribbean strains of Macrocyclops and Mesocyclops (Cyclopoida:Cyclopidae) as biological control tools for the dengue vector Aedes aegypti.* Journal of the American Mosquito Control Association, 1997. **13**(1): p. 18-23.

1148. Rawlins, S.C., et al., *A comparison of surveillance systems for the dengue vector* Aedes Aegypti *in Port of Spain, Trinidad.* Journal of the American Mosquito Control Association, 1998. **14**(2): p. 131-136.

1149. Rawlins, S.C., et al., *A comparison of surveillance systems for the dengue vector* Aedes Aegypti *in Port of Spain, Trinidad.* Journal of the American Mosquito Control Association, 1998. **14**(2): p. 131-136.

1150. Rawlins, S.C. and R. Ragoonansingh, *Comparative organophosphorus insecticide susceptibility in Caribbean populations of* Aedes Aegypti *and Toxorhynchites moctezuma.* Journal of the American Mosquito Control Association, 1990. **6**(2): p. 315-317.

1151. Rawlins, S.C. and J.O. Wan, *Resistance in some Caribbean populations of* Aedes Aegypti *to several insecticides.* Journal of the American Mosquito Control Association, 1995. **11**(1): p. 59-65.

1152. Ray, S. and N. Tandon, *Breeding habitats & seasonal variation in the larval density of* Aedes Aegypti *(L) & Ae. albopictus (Skuse) in an urban garden in Calcutta city.* Indian Journal of Medical Research, 1999. **109**: p. 221-224.

1153. Rebora, A., F. Rongioletti, and V. Raineri, Aedes albopictus *in Europe: a new challenge for dermatologists.* Dermatology, 1993. **187**(1): p. 6-8.

1154. Reed, D., T. Maguire, and J. Mataika, *Type 1 dengue with hemorrhagic disease in Fiji: epidemiologic findings.* American Journal of Tropical Medicine and Hygiene, 1977. **26**(4): p. 784-791.

1155. Reed, D., T. Maguire, and J. Mataika, *Type 1 dengue with hemorrhagic disease in Fiji: epidemiologic findings.* American Journal of Tropical Medicine and Hygiene, 1977. **26**(4): p. 784-791.

1156. Reeves, W.K., et al., *Hematophagous and parasitic Diptera (Insecta) in the Great Smoky Mountains National Park, USA.* Zootaxa, 2004(483): p. 1-44.

1157. Regis, L., et al., *Developing new approaches for detecting and preventing* Aedes Aegypti *population outbreaks: basis for surveillance, alert and control system.* Memorias Do Instituto Oswaldo Cruz, 2008. **103**(1): p. 50-59.

1158. Regnier, A.V., et al., *Field Studies of Gardona against* Aedes-Aegypti *in Puerto-Rico.* Mosquito News, 1971. **31**(3): p. 360-370.

1159. Reiter, P. AEDES ALBOPICTUS *- HONDURAS (3)*. PROMED, 1995.

1160. Reiter, P., et al., *Short report: dispersal of* Aedes Aegypti *in an urban area after blood feeding as demonstrated by rubidium-marked eggs.* American Journal of Tropical Medicine and Hygiene, 1995. **52**(2): p. 177-179.

1161. Reiter, P., M.A. Amador, and N. Colon, *Enhancement of the CDC ovitrap with hay infusions for daily monitoring of* Aedes Aegypti *populations.* Journal of the American Mosquito Control Association, 1991. **7**(1): p. 52-55.

1162. Reiter, P., et al., *First recorded outbreak of yellow fever in Kenya, 1992-1993. II. Entomologic investigations.* American Journal of Tropical Medicine and Hygiene, 1998. **59**(4): p. 650-656.

1163. Reiter, P., et al., *Evaluation of the CDC gravid trap for the surveillance of St. Louis encephalitis vectors in Memphis, Tennessee.* Journal of the American Mosquito Control Association, 1986. **2**(2): p. 209-211.

1164. Remoue, F., et al., *IgE and IgG4 antibody responses to Aedes saliva in African children.* Acta Tropica, 2007.

1165. Reuben, R., *Artificial Breeding Places for Study of* Aedes Aegypti *Breeding in a South Indian Town.* Indian Journal of Medical Research, 1968. **56**(7): p. 1019-1022.

1166. Reuben, R., *Factors in Biology of* Aedes-Aegypti *of Importance to a Genetic Control Programme.* Journal of Communicable Diseases, 1974. **6**(2): p. 117-120.

1167. Reuben, R., et al., *Estimation of Daily Emergence of* Aedes-Aegypti *(Diptera-Culicidae) in Sonepat, India.* Journal of Medical Entomology, 1978. **14**(6): p. 705-714.

1168. Reuben, R., et al., *New Paddle for Black Jar Ovitrap for Surveillance of Aedes-Aegypti.* Indian Journal of Medical Research, 1977. **65**: p. 115-119.

1169. Rey, J.R., et al., *Factors affecting mosquito production from stormwater drains and catch basins in two Florida cities.* Journal of Vector Ecology, 2006. **31**(2): p. 334-343.

1170. Rey, J.R., et al., *Mosquito production from four constructed treatment wetlands in peninsular Florida.* Journal of the American Mosquito Control Association, 2006. **22**(2): p. 198-205.

1171. Reyes-Villanueva, F., *Egg development may require multiple bloodmeals among small* Aedes Aegypti *(Diptera : Culicidae) field collected in Northeastern Mexico.* Florida Entomologist, 2004. **87**(4): p. 630-632.

1172. Reyes-Villanueva, F., H. de la Garza-Garza, and J.A. Flores-Leal, *[Effect of sublethal concentrations of abate on biological parameters of* Aedes aegypti*].* Salud Pública de México, 1992. **34**(4): p. 406-412.

1173. Reyes-Villanueva, F., M. Juarez-Eguia, and A. Flores-Leal, *Effects of sublethal dosages of Abate upon adult fecundity and longevity of* Aedes aegypti*.* Journal of the American Mosquito Control Association, 1990. **6**(4): p. 739-741.

1174. Reyes-Villanueva, F. and M.A. Rodriguez-Perez, *The logistic model for predicting the non-gonoactive* Aedes Aegypti *females.* Salud Pública de México, 2004. **46**(3): p. 234-240.

1175. Reynes, J.M., et al., *The first epidemic of dengue hemorrhagic fever in French Guiana.* American Journal of Tropical Medicine and Hygiene, 1994. **51**(5): p. 545-553.

1176. Ribeiro, H., *Control of* Aedes-Aegypti *during Yellow-Fever Epidemic in Luanda, Angola, in 1971.* Bulletin of the World Health Organization, 1973. **48**(4): p. 504-505.

1177. Richards, S.L., et al., *Spatial analysis of* Aedes albopictus *(Diptera : Culicidae) oviposition in suburban neighborhoods of a piedmont community in North Carolina.* Journal of Medical Entomology, 2006. **43**(5): p. 976-989.

1178. Richards, S.L., et al., *Impact of source reduction on the spatial distribution of larvae and pupae of* Aedes albopictus *(Diptera : Culicidae) in suburban neighborhoods of a Piedmont community in North Carolina.* Journal of Medical Entomology, 2008. **45**(4): p. 617-628.

1179. Richards, S.L., et al., *Host-feeding patterns of* Aedes albopictus *(Diptera : Culicidae) in relation to availability of human and domestic animals in suburban landscapes of central north Carolina.* Journal of Medical Entomology, 2006. **43**(3): p. 543-551.

1180. Richardson, J., et al., *Quantitative analysis of dengue-2 virus RNA during the extrinsic incubation period in individual Aedes aegypti.* American Journal of Tropical Medicine and Hygiene, 2006. **74**(1): p. 132-141.

1181. Richardson, J.H., W.E. Barton, and D.C. Williams, *Survey of container-inhabiting mosquitoes in Clemson, South Carolina, with emphasis on* Aedes albopictus*.* Journal of the American Mosquito Control Association, 1995. **11**(4): p. 396-400.

1182. Rios-Velasquez, C.M., et al., *Distribution of dengue vectors in neighborhoods with different urbanization types of Manaus, state of Amazonas, Brazil.* Memorias Do Instituto Oswaldo Cruz, 2007. **102**(5): p. 617-623.

1183. Ritchie, S.A., *Efficacy of Australian quarantine procedures against the mosquito* Aedes *aegypti.* Journal of the American Mosquito Control Association, 2001. **17**(2): p. 114-117.

1184. Ritchie, S.A., *Effect of some animal feeds and oviposition substrates on* Aedes *oviposition in ovitraps in Cairns, Australia.* Journal of the American Mosquito Control Association, 2001. **17**(3): p. 206-208.

1185. Ritchie, S.A. and S. Long, *Does S-methoprene affect oviposition by* Aedes Aegypti *in an ovitrap?* Journal of the American Mosquito Control Association, 2003. **19**(2): p. 170-171.

1186. Ritchie, S.A., et al., *An adulticidal sticky ovitrap for sampling container-breeding mosquitoes.* Journal of the American Mosquito Control Association, 2003. **19**(3): p. 235-242.

1187. Ritchie, S.A., et al., *Entomological investigations in a focus of dengue transmission in Cairns, Queensland, Australia, by using the sticky ovitraps.* Journal of Medical Entomology 2004. **41**(1): p. 1-4.

1188. Ritchie, S.A., et al., *Entomological investigations in a focus of dengue transmission in Cairns, Queensland, Australia, by using the sticky ovitraps.* Journal of Medical Entomology, 2004. **41**(1): p. 1-4.

1189. Ritchie, S.A., et al., *A biodegradable lethal ovitrap for control of container-breeding Aedes.* Journal of the American Mosquito Control Association, 2008. **24**(1): p. 47-53.

1190. Ritchie, S.A., et al., *Discovery of a widespread infestation of* Aedes albopictus *in the Torres Strait, Australia.* Journal of the American Mosquito Control Association, 2006. **22**(3): p. 358-365.

1191. Robert, L.L. and J.K. Olson, *Susceptibility of Female* Aedes-Albopictus *from Texas to Commonly Used Adulticides.* Journal of the American Mosquito Control Association, 1989. **5**(2): p. 251-253.

1192. Robert, V., et al., *[Yellow fever virus, dengue 2 and other arboviruses isolated from mosquitos, in Burkina Faso, from 1983 to 1986. Entomological and epidemiological considerations].* Bulletin de la Societe de Pathologie Exotique, 1993. **86**(2): p. 90-100.

1193. Robert, V., et al., *[Yellow fever v, dengue 2 and other arboviruses isolated from mosquitos, in Burkina Faso, from 1983 to 1986. Entomological and epidemiological considerationsirus].* Bulletin De La Societe De Pathologie Exotique, 1993. **86**(2): p. 90-100.

1194. Robin, Y. and J. Mouchet, *[Serological and entomological study on yellow fever in Sierra Leone].* Bulletin de la Societe de Pathologie Exotique et de ses Filiales, 1975. **68**(3): p. 249-258.

1195. Robin, Y. and J. Mouchet, *[Serological and entomological study on yellow fever in Sierra Leone].* Bulletin De La Société De Pathologie Exotique Et De Ses Filiales, 1975. **68**(3): p. 249-258.

1196. Rodhain, F., *[Preliminary results of an entomological survey of the potential arbovirus vectors in the French Territory of Afars and Issas].* Bulletin De La Société De Pathologie Exotique Et De Ses Filiales, 1976. **69**(2): p. 169-174.

1197. Rodhain, F., *[Results of a survey of potential dengue vectors in the Neo-Caledonian archipelago].* Bulletin De La Société De Pathologie Exotique Et De Ses Filiales, 1976. **69**(1): p. 21-27.

1198. Rodier, G.R., et al., *Epidemic dengue 2 in the city of Djibouti 1991-1992.* Transactions of the Royal Society of Tropical Medicine and Hygiene, 1996. **90**(3): p. 237-240.

1199. Rodier, G.R., et al., *Epidemic dengue 2 in the city of Djibouti 1991-1992.* Transactions of the Royal Society of Tropical Medicine and Hygiene, 1996. **90**(3): p. 237-240.

1200. Rodrigues, E.M., et al., *[Epidemiology of dengue infection in Ribeirao Preto, SP, Brazil].* Revista de Saude Publica, 2002. **36**(2): p. 160-165.

1201. Rodriguez, A. *DENGUE/DHF UPDATE 2006 (03)*. 2006 [cited 2006 27th Jan].

1202. Rodriguez, A. *DENGUE/DHF UPDATE 2006 (05)*. PROMED, 2006.

1203. Rodriguez, A. *DENGUE/DHF UPDATE 2006 (15)* PROMED, 2006.

1204. Rodriguez, A.J. *DENGUE/DHF UPDATE 2005 (14)*. 2005 [cited 2005 2nd June].

1205. Rodriguez, A.J. *DENGUE/DHF UPDATE 2006 (12)*. 2006 [cited 2006 28th March].

1206. Rodriguez, A.J. *DENGUE/DHF UPDATE 2006 (13)*. 2006 [cited 2006 3rd April].

1207. Rodriguez , A.J. and A.-L. Banks *Dengue/DHF update 2006 (26)* PROMED, 2006.

1208. Rodriguez, H. and F. de la Hoz, *Dengue and dengue and vector behaviour in Caqueza, Colombia, 2004.* Revista de Salud Pública (Bogotá, Colombia)(Bogota), 2005. **7**(1): p. 1-15.

1209. Rodriguez, M.M., et al., *Cross-resistance to pyrethroid and organophosphorus insecticides induced by selection with temephos in* Aedes Aegypti *(Diptera: Culicidae) from Cuba.* Journal of Medical Entomology, 2002. **39**(6): p. 882-888.

1210. Rodriguez, M.M., et al., *Pyrethroid insecticide-resistant strain of* Aedes Aegypti *from Cuba induced by deltamethrin selection.* Journal of the American Mosquito Control Association, 2005. **21**(4): p. 437-445.

1211. Rodriguez, M.M., et al., *[Cross resistance to pyrethroids in* Aedes Aegypti *from Cuba induced by the selection with organophosphate malathion].* Revista Cubana de Medicina Tropical, 2003. **55**(2): p. 105-111.

1212. Rodriguez, P.H. and G.B. Craig, *Susceptibility to Brugia-Pahangi in Geographic Strains of Aedes-Aegypti.* American Journal of Tropical Medicine and Hygiene, 1973. **22**(1): p. 53-61.

1213. Rodriguez Tovar, M.L. and M.G. Ortega Martinez, Aedes albopictus *in Muzquiz city, Coahuila, Mexico.* Journal of the American Mosquito Control Association, 1994. **10**(4): p. 587.

1214. Rodriguez-Figueroa, L., et al., *Risk factors for dengue infection during an outbreak in Yanes, Puerto Rico in 1991.* American Journal of Tropical Medicine and Hygiene, 1995. **52**(6): p. 496-502.

1215. Roesel, T., et al. *Dengue/DHF update 2006 (19)* PROMED, 2006.

1216. Roesel, T., A. Rodriguez , and J. Dudley *Dengue/DHF update 2006 (28)* PROMED, 2006.

1217. Roiz, D., et al., *Initial distribution assessment of* Aedes albopictus *(Diptera : Culicidae) in the Barcelona, Spain, area.* Journal of Medical Entomology, 2008. **45**(3): p. 347-352.

1218. Rojas-Gil, Y. and H. Brochero, *[New record of* Aedes Aegypti *(Linnaeus, 1762), in the urban area of La Pedrera, Amazonas, Colombia].* Biomedica, 2008. **28**(4): p. 587-596.

1219. Romero-Vivas, C.M. and A.K. Falconar, *Investigation of relationships between* Aedes Aegypti *egg, larvae, pupae, and adult density indices where their main breeding sites were located indoors.* Journal of the American Mosquito Control Association, 2005. **21**(1): p. 15-21.

1220. Romero-Vivas, C.M., C.J. Leake, and A.K. Falconar, *Determination of dengue virus serotypes in individual* Aedes Aegypti *mosquitoes in Colombia.* Medical and Veterinary Entomology, 1998. **12**(3): p. 284-288.

1221. Romero-Vivas, C.M., C.J. Sutherland, and A.K. Falconar, *The use of direct sequencing of dengue virus cDNA from individual field-collected* Aedes Aegypti *for surveillance and epidemiological studies.* Medical and Veterinary Entomology, 2000. **14**(1): p. 89-94.

1222. Romero-Vivas, C.M.E., C.J. Leake, and A.K.I. Falconar, *Determination of dengue virus serotypes in individual* Aedes Aegypti *mosquitoes in Colombia.* Medical and Veterinary Entomology, 1998. **12**(3): p. 284-288.

1223. Romi, R., *History and updating on the spread of* Aedes albopictus *in Italy.* Parassitologia, 1995. **37**(2-3): p. 99-103.

1224. Romi, R., et al., *Laboratory and field evaluation of metallic copper on* Aedes albopictus *(Diptera : Culicidae) larval development.* Journal of Medical Entomology, 2000. **37**(2): p. 281-285.

1225. Romi, R., et al., *Potential vectors of West Nile Virus following an equine disease outbreak in Italy.* Medical and Veterinary Entomology, 2004. **18**(1): p. 14-19.

1226. Romi, R., et al., *Susceptibility of Italian populations of* Aedes albopictus *to temephos and to other insecticides.* Journal of the American Mosquito Control Association, 2003. **19**(4): p. 419-423.

1227. Rosen, L., et al., *Comparative susceptibility of mosquito species and strains to oral and parenteral infection with dengue and Japanese encephalitis viruses.* American Journal of Tropical Medicine and Hygiene, 1985. **34**(3): p. 603-615.

1228. Rosen, L., et al., *Transovarial transmission of dengue viruses by mosquitoes:* Aedes albopictus *and Aedes aegypti.* American Journal of Tropical Medicine and Hygiene, 1983. **32**(5): p. 1108-1119.

1229. Rossi, G.C., N.T. Pascual, and F.J. Krsticevic, *First record of* Aedes albopictus *(Skuse) from Argentina.* Journal of the American Mosquito Control Association, 1999. **15**(3): p. 422-422.

1230. Roux, J., et al., *[Epidemic of yellow fever in the southeastern region of Upper Volta (October-December, 1983). Epidemiological study. Preliminary results].* Medecine Tropicale, 1984. **44**(4): p. 303-309.

1231. Rozeboom, L.E., *Relative Densities of Freely Breeding Populations of* Aedes-(S) Polynesiensis *Marks and* a-(S)-Albopictus *Skuse - Large Cage Experiment.* American Journal of Tropical Medicine and Hygiene, 1971. **20**(2): p. 356-362.

1232. Rozilawati, H., et al., *Field bioefficacy of deltamethrin residual spraying against dengue vectors.* Tropical Biomedicine, 2005. **22**(2): p. 143-148.

1233. Rozilawati, H., J. Zairi, and C.R. Adanan, *Seasonal abundance of* Aedes albopictus *in selected urban and suburban areas in Penang, Malaysia.* Tropical Biomedicine, 2007. **24**(1): p. 83-94.

1234. Russell, B.M., et al., *Surveillance of the mosquito* Aedes Aegypti *and its biocontrol with the copepod* Mesocyclops aspericornis *in Australian wells and gold mines.* Medical and Veterinary Entomology, 1996. **10**(2): p. 155-160.

1235. Russell, R.C., *The relative attractiveness of carbon dioxide and octenol in CDC- and EVS-type light traps for sampling the mosquitoes* Aedes Aegypti *(L.),* Aedes polynesiensis *Marks, and* Culex quinquefasciatus *say in Moorea, French Polynesia.* Journal of Vector Ecology 2004. **29**(2): p. 309-314.

1236. Russell, R.C. and J.H. Bryan, *A Survey of Domestic Container-Breeding Mosquitos in New-South-Wales for the Presence of* Aedes-Aegypti *(L), the Vector of Dengue Fever.* Journal of the Australian Entomological Society, 1985. **24**: p. 193-194.

1237. Russell, R.C. and S.A. Ritchie, *Surveillance and behavioral investigations of* Aedes Aegypti *and* Aedes polynesiensis *in Moorea, French Polynesia, using a sticky ovitrap.* Journal of the American Mosquito Control Association, 2004. **20**(4): p. 370-375.

1238. Russell, R.C., C.E. Webb, and N. Davies, Aedes Aegypti *(L.) and* Aedes polynesiensis *marks (Diptera : culicidae) in Moorea, French Polynesia: A study of adult population structures and pathogen (*Wuchereria bancrofti *and* Dirofilaria immitis*) infection rates to indicate regional and seasonal epidemiological risk for dengue and filariasis.* Journal of Medical Entomology, 2005. **42**(6): p. 1045-1056.

1239. Saavedra-Rodriguez, K., et al., *Quantitative trait loci mapping of genome regions controlling permethrin resistance in the mosquito* Aedes aegypti*.* Genetics, 2008. **180**(2): p. 1137-1152.

1240. Sabatini, A., et al., *[*Aedes albopictus *in Italy and possible diffusion of the species into the Mediterranean area].* Parassitologia, 1990. **32**(3): p. 301-304.

1241. Saelim, V., et al., *Bottle and biochemical assays on temephos resistance in* Aedes Aegypti *in Thailand.* Southeast Asian Journal of Tropical Medicine and Public Health, 2005. **36**(2): p. 417-425.

1242. Salas-Luevano, M.A. and F. Reyes-Villanueva, *[Seasonal variations in* Aedes Aegypti *populations in Monterrey, Mexico].* Salud Pública de México, 1994. **36**(4): p. 385-392.

1243. Samarawickrema, W.A., et al., *The relative importance and distribution of* Aedes polynesiensis *and Ae. aegypti larval habitats in Samoa.* Medical and Veterinary Entomology, 1993. **7**(1): p. 27-36.

1244. Sames, W.J., et al., *Insecticide susceptibility of* Aedes Aegypti *and* Aedes albopictus *in the lower Rio Grande Valley of Texas and Mexico.* Journal of the American Mosquito Control Association, 1996. **12**(3): p. 487-490.

1245. Samuel, P.P., V. Thenmozhi, and B.K. Tyagi, *A focal outbreak of dengue fever in a rural area of Tamil Nadu.* Indian Journal of Medical Research, 2007. **125**(2): p. 179-181.

1246. Samui, K.L., et al., *Mosquitoes captured in a horse-baited stable trap in southeast Louisiana.* Journal of the American Mosquito Control Association, 2003. **19**(2): p. 139-147.

1247. Sanchez, L., et al., *[A community education strategy to promote participation in dengue prevention in Cuba].* Revista Panamericana de Salud Publica-Pan American Journal of Public Health, 2008. **24**(1): p. 61-69.

1248. Sanchez, L., et al., *Intersectoral coordination in* Aedes Aegypti *control. A pilot project in Havana City, Cuba.* Tropical Medicine & International Health, 2005. **10**(1): p. 82-91.

1249. Sanchez, L., et al., Aedes Aegypti *larval indices and risk for dengue epidemics.* Emerging Infectious Diseases, 2006. **12**(5): p. 800-806.

1250. Sanders, E.J., et al., *First recorded outbreak of yellow fever in Kenya, 1992-1993. I. Epidemiologic investigations.* American Journal of Tropical Medicine and Hygiene, 1998. **59**(4): p. 644-649.

1251. Sang, R.C., et al., *Entomologic investigations of a chikungunya virus epidemic in the union of the Comoros, 2005.* American Journal of Tropical Medicine and Hygiene, 2008. **78**(1): p. 77-82.

1252. Sant'Ana, A.L., *[First recorded occurrence of* Aedes (Stegomyia) albopictus *(Skuse) in the southeastern region of Brazil].* Revista de Saúde Pública, 1996. **30**(4): p. 392-393.

1253. Santamarina Mijares, A. and M.C. Perez Pacheco, *[Pathogenic effect of the nematode parasite Romanomermis iyengari(Nematoda: Mermithidae) in* Aedes Aegypti *mosquito larvae (Diptera: Culicidae) under laboratory conditions in the state of Oaxaca, Mexico].* Revista Cubana de Medicina Tropical, 1998. **50**(1): p. 8-11.

1254. Santamarina Mijares, A., R. Perez Pacheco, and S. Honorio Martinez, *[Susceptibility of* Aedes Aegypti *larvae to parasitism by Romanomermis culicivorax in laboratory and field conditions in Oaxaca, Mexico].* Revista Panamericana de Salud Pública, 2000. **8**(5): p. 299-304.

1255. Santos, L.U., F.S. Andrade, and G.A. Carvalho, *Biological control of* Aedes albopictus *(Diptera: Culicidae) larvae in trap tyres by* Mesocyclops longisetus *(Copepoda: Cyclopidae) in two field trials.* Memorias Do Instituto Oswaldo Cruz, 1996. **91**(2): p. 161-162.

1256. Santos-Torres, S., et al., *[The immune state against yellow fever vaccinal virus (17D) in 2 populations of Bahia State, Brazil].* Revista da Sociedade Brasileira de Medicina Tropical, 2000. **33**(1): p. 39-46.

1257. Sardelis, M.R., et al., *Vector competence of three North American strains of* Aedes albopictus *for West Nile virus.* Journal of the American Mosquito Control Association, 2002. **18**(4): p. 284-289.

1258. Sarthou, J.L., et al., *Isolation of Rift Valley fever virus from human peripheral blood mononuclear cells: Mauritanian epidemic.* Research in Virology, 1989. **140**(3): p. 263-270.

1259. Saul, S.H., P. Guptavanij, and G.B. Craig, *Genetic-Variability at an Esterase Locus in* Aedes-Aegypti *(Diptera-Culicidae).* Annals of the Entomological Society of America, 1976. **69**(1): p. 73-79.

1260. Saul, S.H., R.J. Novak, and Q.E. Ross, *The Role of the Preadult Stages in the Ecological Separation of 2 Subspecies of Aedes-Aegypti.* American Midland Naturalist, 1980. **104**(1): p. 118-134.

1261. Savage, H.M., et al., *First record of breeding populations of* Aedes albopictus *in continental Africa: implications for arboviral transmission.* Journal of the American Mosquito Control Association, 1992. **8**(1): p. 101-103.

1262. Savage, H.M., et al., *Host-feeding patterns of* Aedes albopictus *(Diptera: Culicidae) at a temperate North American site.* Journal of Medical Entomology, 1993. **30**(1): p. 27-34.

1263. Savage, H.M., et al., *Vector competence of* Aedes albopictus *from Pine Bluff, Arkansas, for a St. Louis encephalitis virus strain isolated during the 1991 epidemic.* Journal of the American Mosquito Control Association, 1994. **10**(4): p. 501-506.

1264. Savage, H.M., et al., *Entomologic investigations of an epidemic of St. Louis encephalitis in Pine Bluff, Arkansas, 1991.* American Journal of Tropical Medicine and Hygiene, 1993. **49**(1): p. 38-45.

1265. Sawabe, K. and M. Mogi, *Differences in energy metabolism and adult desiccation resistance among three* Aedes (Stegomyia) *species (Diptera: Culicidae) from South Sulawesi, Indonesia.* Journal of Medical Entomology, 1999. **36**(1): p. 101-107.

1266. Scarpassa, V.M., T.B. Cardoza, and R.P. Cardoso, *Population genetics and phylogeography of* Aedes aegipti *(Diptera : Culicidae) from Brazil.* American Journal of Tropical Medicine and Hygiene, 2008. **78**(6): p. 895-903.

1267. Scat, Y., O. Moreau, and V. Fougere, *[The value of an immunocapture method for the study of serum IgM in the surveillance of dengue in Martinique].* Bulletin de la Societe de Pathologie Exotique et de ses Filiales, 1989. **82**(2): p. 173-184.

1268. Schaffner, F. and S. Karch, *[First report of* Aedes albopictus *(Skuse, 1984) in metropolitan France].* Comptes Rendus de l'Académie des Sciences. Série III, Sciences de la vie, 2000. **323**(4): p. 373-375.

1269. Schioler, K.L. and C.N. Macpherson, *Dengue Transmission in the Small-Island Setting: Investigations from the Caribbean Island of Grenada.* American Journal of Tropical Medicine and Hygiene, 2009. **81**(2): p. 280-286.

1270. Schneider, J.R., et al., *Adult size and distribution of* Aedes Aegypti *(Diptera: Culicidae) associated with larval habitats in Iquitos, Peru.* Journal of Medical Entomology, 2004. **41**(4): p. 634-642.

1271. Schoeler, G.B., et al., *Evaluation of surveillance devices for monitoring* Aedes Aegypti *in an urban area of northeastern Peru.* Journal of the American Mosquito Control Association, 2004. **20**(1): p. 6-11.

1272. Schreck, C.E., *Permethrin and dimethyl phthalate as tent fabric treatments against Aedes aegypti.* Journal of the American Mosquito Control Association, 1991. **7**(4): p. 533-535.

1273. Schultz, G.W., *Cemetery vase breeding of dengue vectors in Manila, Republic of the Philippines.* Journal of the American Mosquito Control Association, 1989. **5**(4): p. 508-513.

1274. Schweigmann, N., et al., Aedes albopictus *in an area of Misiones, Argentina.* Revista de Saude Publica, 2004. **38**(1): p. 136-138.

1275. Scott, T.W., et al., *Longitudinal studies of* Aedes Aegypti *(Diptera: Culicidae) in Thailand and Puerto Rico: blood feeding frequency.* Journal of Medical Entomology, 2000. **37**(1): p. 89-101.

1276. Scott, T.W., et al., *Blood-feeding patterns of* Aedes Aegypti *(Diptera: Culicidae) collected in a rural Thai village.* Journal of Medical Entomology, 1993. **30**(5): p. 922-927.

1277. Scott, T.W., et al., *Longitudinal studies of* Aedes Aegypti *(Diptera: Culicidae) in Thailand and Puerto Rico: population dynamics.* Journal of Medical Entomology, 2000. **37**(1): p. 77-88.

1278. Seawright, J.A., P.E. Kaiser, and D.A. Dame, *Mating Competitiveness of Chemosterilized Hybrid Males of* Aedes-Aegypti *(L) in Outdoor Cage Studies.* Mosquito News, 1975. **35**(3): p. 308-314.

1279. Seawright, J.A., P.E. Kaiser, and D.A. Dame, *Mating Competitiveness of Chemosterilized Hybrid Males of* Aedes-Aegypti *(L) in Field-Tests.* Mosquito News, 1977. **37**(4): p. 615-619.

1280. Seawright, J.A., et al., *Field Competitiveness of Males of* Aedes-Aegypti *(L) Heterozygous for a Translocation.* Mosquito News, 1975. **35**(1): p. 30-33.

1281. Seawright, J.A., et al., *Field Competitiveness of Double Translocation Heterozygote Males of* Aedes-Aegypti *(L).* Journal of Medical Entomology, 1976. **13**(2): p. 208-211.

1282. Seawright, J.A., et al., *Reciprocal Translocations in* Aedes-Aegypti *(L).* Mosquito News, 1975. **35**(3): p. 384-388.

1283. Sebastian, A., et al., *Suppression of* Aedes-Aegypti *(Diptera, Culicidae) Using Augmentative Release of Dragonfly Larvae (Odonata, Libellulidae) with Community Participation in Yangon, Myanmar.* Bulletin of Entomological Research, 1990. **80**(2): p. 223-232.

1284. Seed, C.R., et al., *The risk of dengue transmission by blood during a 2004 outbreak in Cairns, Australia.* Transfusion, 2009. **49**(7): p. 1482-1487.

1285. Seet, R.C.S., et al., *Oxidative damage in dengue fever.* Free Radical Biology and Medicine, 2009. **47**(4): p. 375-380.

1286. Seet, R.C.S., A.M.L. Quek, and E.C.H. Lim, *Symptoms and risk factors of ocular complications following dengue infection.* Journal of Clinical Virology, 2007. **38**(2): p. 101-105.

1287. Segura Mde, N., et al., *[Occurrence of* Aedes albopictus *in the state of Para, Brazil].* Revista de Saúde Pública, 2003. **37**(3): p. 388-389.

1288. Self, L.S., et al., *Reduction in Hospitalized Cases of Dengue Hemorrhagic-Fever in Menado (Sulawesi), Indonesia after Aerial Spraying with Ulv Malathion to Control* Aedes-Aegypti*.* Journal of the Medical Association of Thailand, 1977. **60**(10): p. 482-492.

1289. Seng, C.M. and N. Jute, *Breeding of* Aedes Aegypti *(L.) and* Aedes albopictus *(Skuse) in urban housing of Sibu town, Sarawak.* Southeast Asian Journal of Tropical Medicine and Public Health, 1994. **25**(3): p. 543-548.

1290. Seravali, M.R., et al., *Spontaneous splenic rupture due to dengue fever: report of two cases.* Brazilian Journal of Infectious Diseases, 2008. **12**(6): p. 538-540.

1291. Serpa, L.L., et al., *[Seasonal variation of* Aedes Aegypti *and* Aedes albopictus *in a city of Southeastern Brazil].* Revista de Saúde Pública, 2006. **40**(6): p. 1101-1105.

1292. Serpa, L.L., I. Kakitani, and J.C. Voltolini, *[Competition between* Aedes Aegypti *and* Aedes albopictus *larvae in the laboratory].* Revista da Sociedade Brasileira de Medicina Tropical, 2008. **41**(5): p. 479-484.

1293. Serufo, J.C., et al., *Isolation of Dengue Virus Type-1 from Larvae of* Aedes-Albopictus *in Campos-Altos City, State of Minas-Gerais, Brazil.* Memorias Do Instituto Oswaldo Cruz, 1993. **88**(3): p. 503-504.

1294. Shaalan, E.A.S., et al., *Synergistic efficacy of botanical blends with and without synthetic insecticides against* Aedes Aegypti *and* Culex annulirostris *mosquitoes.* Journal of Vector Ecology, 2005. **30**(2): p. 284-288.

1295. Shamaan, N.A., et al., *Insecticide toxicity, glutathione transferases and carboxylesterase activities in the larva of the Aedes mosquito.* Comparative Biochemistry and Physiology. C: Comparative Pharmacology, 1993. **104**(1): p. 107-110.

1296. Sharma, N., et al., *Dengue fever related acalculous cholecystitis in a North Indian tertiary care hospital.* Journal of Gastroenterology and Hepatology, 2006. **21**(4): p. 664-667.

1297. Sharma, S.K., et al., *Observations on the breeding habitat of* Aedes *species in the steel township, Rourkela.* Journal of Communicable Diseases, 2001. **33**(1): p. 28-35.

1298. Sharma, S.N., et al., *Entomological indices of* Aedes Aegypti *at some international airports and seaports of southern India--a report.* Journal of Communicable Diseases, 2005. **37**(3): p. 173-181.

1299. Sharma, S.N., S. Lal, and V.K. Saxena, *Surveillance of dengue vector at thiruvananthapuram (Kerala) International Airport.* Journal of Communicable Diseases, 2004. **36**(2): p. 136-143.

1300. Sheppard, P.M., Macdonal.Ww, and R.J. Tonn, *A New Method of Measuring Relative Prevalence of* Aedes Aegypti*.* Bulletin of the World Health Organization, 1969. **40**(3): p. 467-468.

1301. Shetty, P.S., V. Dhanda, and R.B. Deobhankar, *Year Round Study of* Aedes-Aegypti *in Barsi Town, Maharashtra State.* Indian Journal of Medical Research, 1978. **67**(Jun): p. 942-946.

1302. Shetty, P.S. and G. Geevarghese, *Tree-Hole Breeding of* Aedes-Aegypti *in Poona City.* Indian Journal of Medical Research, 1977. **66**(2): p. 172-174.

1303. Shirai, Y., et al., *Landing sites on the human body preferred by* Aedes albopictus*.* Journal of the American Mosquito Control Association, 2002. **18**(2): p. 97-99.

1304. Shriram, A.N. and S.C. Sehgal, Aedes Aegypti *(L) in Port Blair, Andaman and Nicobar islands-distribution and larval ecology.* Journal of Communicable Diseases, 1999. **31**(3): p. 185-192.

1305. Shriram, A.N., A.P. Sugunan, and P. Vijayachari, *Infiltration of* Aedes Aegypti *into peri-urban areas in South Andaman.* Indian Journal of Medical Research, 2008. **127**(6): p. 618-620.

1306. Shu, L.P., et al., *[Susceptibility of 15 collections of* Aedes albopictus *from Guizhou to dengue virus oral infection].* Chinese Journal of Experimental and Clinical Virology - Zhonghua Shi Yan He Lin Chuang Bing Du Xue Za Zhi 2004. **18**(3): p. 234-237.

1307. Silva Jdos, S., et al., *[*Anopheles (Nyssorhynchus) argyritarsis *larvae found in artificial breeding sites in the State of Mato Grosso].* Revista da Sociedade Brasileira de Medicina Tropical, 2008. **41**(3): p. 313-314.

1308. Silva, J.J. and J. Mendes, *Susceptibility of* Aedes Aegypti *(L) to the insect growth regulators diflubenzuron and methoprene in Uberlandia, State of Minas Gerais.* Revista da Sociedade Brasileira de Medicina Tropical, 2007. **40**(6): p. 612-616.

1309. Silver, D. *DENGUE/DHF UPDATE 2007 (12)*. PROMED, 2007.

1310. Simard, F., et al., *Geographic distribution and breeding site preference of* Aedes albopictus *and* Aedes Aegypti *(Diptera: culicidae) in Cameroon, Central Africa.* Journal of Medical Entomology, 2005. **42**(5): p. 726-731.

1311. Singfield, P. *DENGUE - BELIZE (02)*. 1997 [cited 1997 27 October]; the "Reporter" newspaper, Belize.

1312. Singh, J., et al., *Silent spread of dengue and dengue haemorrhagic fever to Coimbatore and Erode districts in Tamil Nadu, India, 1998: need for effective surveillance to monitor and control the disease.* Epidemiology and Infection 2000. **125**(1): p. 195-200.

1313. Singh, J., et al., *Silent spread of dengue and dengue haemorrhagic fever to Coimbatore and Erode districts in Tamil Nadu, India, 1998: need for effective surveillance to monitor and control the disease.* Epidemiology and Infection, 2000. **125**(1): p. 195-200.

1314. Singh, K.R.P. and K.M. Pavri, *Experimental Studies with Chikungunya Virus in* Aedes Aegypti *and Aedes Alpopictus.* Acta Virologica, 1967. **11**(6): p. 517-526.

1315. Singh, K.R.P. and J.K. Sarkar, *Isolation of Chikungunya Virus from* Aedes Aegypti *from Calcutta India.* Current Science, 1965. **34**(16): p. 480-481.

1316. Singh, R. and K. Sharma, *Density of Potential Vector of Dengue Hemorrhagic-Fever,* Aedes-Aegypti *(Diptera, Culicidae).* Entomon, 1984. **9**(2): p. 93-95.

1317. Singh, U.B. and P. Seth, *Use of nucleotide sequencing of the genomic cDNA fragments of the capsid/premembrane junction region for molecular epidemiology of dengue type 2 viruses.* Southeast Asian Journal of Tropical Medicine and Public Health, 2001. **32**(2): p. 326-335.

1318. Siqueira, J.B., et al., *Dengue and dengue hemorrhagic fever, Brazil, 1981-2002.* Emerging Infectious Diseases, 2005. **11**(1): p. 48-53.

1319. Sithiprasasna, R., et al., *Field evaluation of a lethal ovitrap for the control of* Aedes Aegypti *(Diptera : Culicidae) in Thailand.* Journal of Medical Entomology, 2003. **40**(4): p. 455-462.

1320. Sithiprasasna, R., et al., *The geographic information system as an epidemiological tool in the surveillance of dengue virus-infected Aedes mosquitos.* Southeast Asian Journal of Tropical Medicine and Public Health, 2004. **35**(4): p. 918-926.

1321. Sithiprasasna, R., et al., *The geographic information system as an epidemiological tool in the surveillance of dengue virus-infected Aedes mosquitos.* Southeast Asian Journal of Tropical Medicine and Public Health, 2004. **35**(4): p. 918-926.

1322. Sivagnaname, N., et al., *Oviposition attractancy of an infusion from a wood inhabiting fungus for vector mosquitoes.* Indian Journal of Medical Research, 2001. **114**: p. 18-24.

1323. Smith, C.E., et al., *Dengue risk among visitors to Hawaii during an outbreak.* Emerging Infectious Diseases, 2005. **11**(5): p. 750-756.

1324. Smith, D.R., et al., *Evaluation of methods to assess transmission potential of Venezuelan equine encephalitis virus by mosquitoes and estimation of mosquito saliva titers.* American Journal of Tropical Medicine and Hygiene, 2005. **73**(1): p. 33-39.

1325. Smith, J.P., T.M. Loyless, and J.A. Mulrennan, Jr., *An update on* Aedes albopictus *in Florida.* Journal of the American Mosquito Control Association, 1990. **6**(2): p. 318-320.

1326. Soman, R.S., *Studies on* Aedes-Aegypti *in Bangalore City.* Indian Journal of Medical Research, 1977. **65**(1): p. 8-16.

1327. Soman, R.S., *Studies on Diel Periodicity in Landing of* Aedes-Aegypti *on Man in Bangalore City.* Indian Journal of Medical Research, 1978. **67**(Jun): p. 937-941.

1328. Sota, T., *Response to Selection for Desiccation Resistance in* Aedes-Albopictus *Eggs (Diptera, Culicidae).* Applied Entomology and Zoology, 1993. **28**(2): p. 161-168.

1329. Sota, T. and M. Mogi, *Interspecific Variation in Desiccation Survival-Time of Aedes (Stegomyia) Mosquito Eggs Is Correlated with Habitat and Egg Size.* Oecologia, 1992. **90**(3): p. 353-358.

1330. Sota, T., M. Mogi, and E. Hayamizu, *Seasonal distribution and habitat selection by* Aedes albopictus *and* Ae. riversi *(Diptera: Culicidae) in northern Kyushu, Japan.* Journal of Medical Entomology, 1992. **29**(2): p. 296-304.

1331. Sota, T., M. Mogi, and E. Hayamizu, *Habitat Stability and the Larval Mosquito Community in Treeholes and Other Containers on a Temperate Island.* Researches on Population Ecology, 1994. **36**(1): p. 93-104.

1332. Soubihe, V., et al., *[Prevalence of* Aedes-(Stegomyia)-Albopictus *(Skuse) in the City of Sao-Paulo Sp, Brazil].* Revista de Saude Publica, 1992. **26**(1): p. 57-57.

1333. Sousa-Polezzi, R.D. and H.E.M.D. Bicudo, *Effect of phenobarbital on inducing insecticide tolerance and esterase changes in* Aedes Aegypti *(Diptera : Culicidae).* Genetics and Molecular Biology, 2004. **27**(2): p. 275-283.

1334. Sousa-Polezzi, R.D. and H.E.M.D. Bicudo, *Genetic variation along time in a brazilian population of* Aedes Aegypti *(Diptera : Culicidae), detected by changes in the esterase patterns.* Genetica, 2005. **125**(1): p. 43-53.

1335. Southowood, T.R., et al., *Studies on Life Budget of* Aedes-Aegypti *in Wat Samphaya, Bangkok, Thailand.* Bulletin of the World Health Organization, 1972. **46**(2): p. 211-226.

1336. Souza, L.J., et al., *Aminotransferase changes and acute hepatitis in patients with dengue fever: analysis of 1,585 cases.* Brazilian Journal of Infectious Diseases, 2004. **8**(2): p. 156-63.

1337. Souza-Santos, R., *[The factors associated with the occurrence of immature forms of* Aedes Aegypti *in Ilha do Governador, Rio de Janeiro, Brazil].* Revista da Sociedade Brasileira de Medicina Tropical, 1999. **32**(4): p. 373-382.

1338. Spencer, C.Y., T.H. Pendergast, and L.C. Harrington, *Fructose variation in the dengue vector, Aedes Aegypti, during high and low transmission seasons in the Mae Sot region of Thailand.* Journal of the American Mosquito Control Association, 2005. **21**(2): p. 177-181.

1339. Spielman, A. and F.M. Feinsod, *Differential distribution of peridomestic Aedes mosquitoes on Grand Bahama Island.* Transactions of the Royal Society of Tropical Medicine and Hygiene, 1979. **73**(4): p. 381-384.

1340. Sprenger, D. and T. Wuithiranyagool, *The Discovery and Distribution of* Aedes-Albopictus *in Harris County, Texas.* Journal of the American Mosquito Control Association, 1986. **2**(2): p. 217-219.

1341. Srisuphanunt, M., et al., *ELISA as an alternative tool for epidemiological surveillance for dengue in mosquitoes: a report from Thailand.* Journal of Vector Borne Diseases, 2007. **44**(4): p. 272-276.

1342. Stapp, R.R. and J. Casten, *Field Studies on Lankesteria-Culicis and* Aedes-Aegypti *in Florida.* Mosquito News, 1971. **31**(1): p. 18-22.

1343. Stasiak, R.S., R.H. Grothaus, and W.F. Miner, *Susceptibility of* Aedes-Albopictus *(Skuse) Larvae from South-Vietnam to 5 Insecticides, 1969.* Mosquito News, 1970. **30**(2): p. 246-249.

1344. Stein, M., G.I. Oria, and W.R. Almiron, *[Main breeding-containers for* Aedes Aegypti *and associated culicids, Argentina].* Revista de Saúde Pública, 2002. **36**(5): p. 627-630.

1345. Stein, M., et al., *[Seasonal fluctuation of* Aedes Aegypti *in Chaco Province, Argentina].* Revista de Saúde Pública, 2005. **39**(4): p. 559-564.

1346. Steinly, B.A., R.J. Novak, and D.W. Webb, *A new method for monitoring mosquito oviposition in artificial and natural containers.* Journal of the American Mosquito Control Association, 1991. **7**(4): p. 649-650.

1347. Strickman, D., *Longevity of* Aedes Aegypti *(Diptera: Culicidae) compared in cages and field under ambient conditions in rural Thailand.* Southeast Asian Journal of Tropical Medicine and Public Health, 2006. **37**(3): p. 456-462.

1348. Strickman, D., et al., *Mosquito collections following local transmission of Plasmodium falciparum malaria in Westmoreland County, Virginia.* Journal of the American Mosquito Control Association, 2000. **16**(3): p. 219-222.

1349. Strickman, D. and P. Kittayapong, *Dengue and its vectors in Thailand: introduction to the study and seasonal distribution of Aedes larvae.* American Journal of Tropical Medicine and Hygiene, 2002. **67**(3): p. 247-259.

1350. Strickman, D. and P. Kittayapong, *Dengue and its vectors in Thailand: calculated transmission risk from total pupal counts of* Aedes Aegypti *and association of wing-length measurements with aspects of the larval habitat.* American Journal of Tropical Medicine and Hygiene, 2003. **68**(2): p. 209-217.

1351. Su, Y.C., et al., *Genetic relationships among populations of* Aedes Aegypti *in Taiwan by using phenotypic and random amplified DNA-polymerase chain reaction markers.* Journal of the American Mosquito Control Association, 2003. **19**(4): p. 329-338.

1352. Suarez, O.M., *[Multiplication in and Transmission by* Aedes Aegypti *(L) Mosquitoes of Vsv Virus].* Acta Cientifica Venezolana, 1967. **S 18**: p. 387-394.

1353. Suarez Sarmiento, E., et al., *[Analysis of the results of the eradication campaign against the* Aedes Aegypti *mosquito in the municipality of Consolacion del Sur].* Revista Cubana de Medicina Tropical, 1989. **41**(2): p. 226-235.

1354. Subra, R., *The regulation of preimaginal populations of* Aedes Aegypti *L. (Diptera: Culicidae) on the Kenya coast. I. Preimaginal population dynamics and the role of human behaviour.* Annals of Tropical Medicine and Parasitology, 1983. **77**(2): p. 195-201.

1355. Subra, R. and J. Mouchet, *The regulation of preimaginal populations of* Aedes Aegypti *(L.) (Diptera: Culicidae) on the Kenya coast. II. Food as a main regulatory factor.* Annals of Tropical Medicine and Parasitology, 1984. **78**(1): p. 63-70.

1356. Suguna, S.G., et al., *Disorter-Double Translocation Heterozygote Systems in Aedes-Aegypti.* Genetica, 1977. **47**(2): p. 117-123.

1357. Suguna, S.G., S.J. Kazmi, and C.F. Curtis, *Sex-Ratio Distorter-Translocation Homozygotes in* Aedes-Aegypti*.* Genetica, 1977. **47**(2): p. 125-133.

1358. Suguna, S.G., et al., *Resistance to Meiotic Drive at Md Locus in an Indian Wild Population of* Aedes-Aegypti*.* Genetical Research, 1977. **29**(2): p. 123-132.

1359. Sukonthabhirom, S., et al., *Genetic structure and gene flow among* Aedes Aegypti *(Diptera: Culicidae) populations from central Thailand.* Journal of Medical Entomology, 2005. **42**(4): p. 604-609.

1360. Sulaiman, I., *Development of Dirofilaria repens in* Aedes Aegypti *reared in contrasting habitat.* Southeast Asian Journal of Tropical Medicine and Public Health, 1983. **14**(1): p. 122-126.

1361. Sulaiman, S. and J. Jeffery, *The Ecology of* Aedes-Albopictus *(Skuse) (Diptera, Culicidae) in a Rubber Estate in Malaysia.* Bulletin of Entomological Research, 1986. **76**(4): p. 553-557.

1362. Sulaiman, S. and J. Jeffery, *Field studies on populations of* Aedes albopictus *and Toxorhynchites species in bamboo pots in Malaysia.* Journal of the American Mosquito Control Association, 1994. **10**(3): p. 460-461.

1363. Sulaiman, S., et al., *Field evaluation of alphacypermethrin and lambda-cyhalothrin against* Aedes Aegypti *and* Aedes albopictus *in Malaysia.* Journal of the American Mosquito Control Association, 1995. **11**(1): p. 54-58.

1364. Sulaiman, S., et al., *Relationship between Breteau and House indices and cases of dengue/dengue hemorrhagic fever in Kuala Lumpur, Malaysia.* Journal of the American Mosquito Control Association, 1996. **12**(3 Pt 1): p. 494-496.

1365. Sulaiman, S., et al., *Relationship between Breteau and house indices and cases of dengue/dengue hemorrhagic fever in Kuala Lumpur, Malaysia.* Journal of the American Mosquito Control Association, 1996. **12**(3): p. 494-496.

1366. Sulaiman, S., et al., *The residual effects of alphacypermethrin and permethrin against the dengue vector* Aedes albopictus *(Skuse) in wooden huts in Malaysia.* Journal of Vector Ecology, 1996. **21**(1): p. 85-88.

1367. Sulaiman, S., et al., *Serological identification of the predators of adult* Aedes albopictus *(Skuse) (Diptera: Culicidae) in rubber plantations and a cemetery in Malaysia.* Journal of Vector Ecology, 1996. **21**(1): p. 22-25.

1368. Sulaiman, S., et al., *Field evaluation of cyfluthrin and malathion 96 TG ULV spraying at high-rise flats on dengue vectors in Malaysia.* Journal of Vector Ecology 1998. **23**(1): p. 69-73.

1369. Sulaiman, S., et al., *Field evaluation of cypermethrin and cyfluthrin against dengue vectors in a housing estate in Malaysia.* Journal of Vector Ecology

, 2002. **27**(2): p. 230-234.

1370. Sulaiman, S., H. Yunus, and R. Sohadi, *Evaluation of some adhesives for collecting Musca domestica and Chrysomya megacephala adults or mosquito larvae in sticky traps.* Medical and Veterinary Entomology, 1987. **1**(3): p. 273-278.

1371. Suleman, M., M. Arshad, and K. Khan, *Yellowfever mosquito (Diptera:Culicidae) introduced into Landi Kotal, Pakistan, by tire importation.* Journal of Medical Entomology, 1996. **33**(4): p. 689-693.

1372. Sun, Z., *[Ecological Investigation on* Aedes-Albopictus *(Skuse) in Nan-Young District].* Acta Entomologica Sinica, 1979. **22**(2): p. 213-216.

1373. Sunahara, T. and M. Mogi, *Can the tortoise beat the hare? A possible mechanism for the coexistence of competing mosquitoes in bamboo groves.* Ecological Research, 1997. **12**(1): p. 63-70.

1374. Sunahara, T. and M. Mogi, *Distributions of larval mosquitoes among bamboo-stump pools which vary in persistence and resource input.* Researches on Population Ecology, 1997. **39**(2): p. 173-179.

1375. Sunahara, T. and M. Mogi, *Variability of intra- and interspecific competitions of bamboo stump mosquito larvae over small and large spatial scales.* Oikos, 2002. **97**(1): p. 87-96.

1376. Sunahara, T., M. Mogi, and M. Selomo, *Mosquito immatures in drought-prone and drought-resistant bamboo stumps in Flores, Indonesia.* Journal of the American Mosquito Control Association, 1999. **15**(3): p. 271-275.

1377. Surendran, S.N., et al., *Seasonality and insecticide susceptibility of dengue vectors: an ovitrap based survey in a residential area of northern Sri Lanka.* Southeast Asian Journal of Tropical Medicine and Public Health, 2007. **38**(2): p. 276-282.

1378. Suwonkerd, W., et al., *The effect of host type on movement patterns of* Aedes Aegypti *(Diptera: Culicidae) into and out of experimental huts in Thailand.* Journal of Vector Ecology 2006. **31**(2): p. 311-318.

1379. Suzuki, T. and J.H. Hirshman, *Distribution and Density of* Aedes-Aegypti *in South-Pacific.* New Zealand Medical Journal, 1977. **85**(587): p. 374-380.

1380. Swaddiwudhipong, W., et al., *Effect of health education on community participation in control of dengue hemorrhagic fever in an urban area of Thailand.* Southeast Asian Journal of Tropical Medicine and Public Health, 1992. **23**(2): p. 200-206.

1381. Swanson, J., et al., *Overwintering and establishment of* Aedes albopictus *(Diptera : Culicidae) in an urban La Crosse virus enzootic site in Illinois.* Journal of Medical Entomology, 2000. **37**(3): p. 454-460.

1382. Sweeney, K.J., *Organophosphorous insecticide susceptibility of mosquitoes in Maryland, 1985-89.* Journal of the American Mosquito Control Association, 1993. **9**(1): p. 8-12.

1383. Sweeney, K.J., M.A. Cantwell, and J. Dorothy, *The Collection of* Aedes-Aegypti *and* Aedes-Albopictus *from Baltimore, Maryland.* Journal of the American Mosquito Control Association, 1988. **4**(3): p. 381-382.

1384. Sylla, M., et al., *Gene Flow, Subspecies Composition, and Dengue Virus-2 Susceptibility among* Aedes Aegypti *Collections in Senegal.* Plos Neglected Tropical Diseases, 2009. **3**(4): p. e408.

1385. Tabachnick, W.J., *Identification and linkage relationships of three hexokinase genes in Aedes aegypti.* Biochemical Genetics, 1978. **16**(5-6): p. 571-575.

1386. Tabachnick, W.J., *Geographic and temporal patterns of genetic variation of* Aedes Aegypti *in New Orleans.* American Journal of Tropical Medicine and Hygiene, 1982. **31**(4): p. 849-853.

1387. Tabachnick, W.J., L.E. Munstermann, and J.R. Powell, *Genetic Distinctness of Sympatric Forms of* Aedes-Aegypti *in East-Africa.* Evolution, 1979. **33**(1): p. 287-295.

1388. Tabachnick, W.J. and J.R. Powell, *Genetic Structure of East-African Domestic Populations of Aedes-Aegypti.* Nature, 1978. **272**(5653): p. 535-537.

1389. Tabachnick, W.J. and J.R. Powell, *World-Wide Survey of Genetic-Variation in the Yellow-Fever Mosquito,* Aedes-Aegypti*.* Genetical Research, 1979. **34**(3): p. 215-229.

1390. Tabachnick, W.J., et al., *Oral infection of* Aedes Aegypti *with yellow fever virus: geographic variation and genetic considerations.* American Journal of Tropical Medicine and Hygiene, 1985. **34**(6): p. 1219-1224.

1391. Tadano, T., *Linkage Studies on 2 New Mutants, Frosty-Body and Pigmented Pupa, in the Mosquito* Aedes-Albopictus*.* Mosquito News, 1981. **41**(2): p. 348-355.

1392. Tanner, G.D., *Oviposition Traps and Population Sampling for Distribution of* Aedes Aegypti *(L).* Mosquito News, 1969. **29**(1): p. 116-121.

1393. Tardieux, I., et al., *Variation among strains of* Aedes Aegypti *in susceptibility to oral infection with dengue virus type 2.* American Journal of Tropical Medicine and Hygiene, 1990. **43**(3): p. 308-313.

1394. Tavares-Neto, J., et al., *[Serologic survey for yellow fever and other arboviruses among inhabitants of Rio Branco, Brazil, before and three months after receiving the yellow fever 17D vaccine].* Revista da Sociedade Brasileira de Medicina Tropical, 2004. **37**(1): p. 1-6.

1395. Teixeira, M.G., et al., *[Epidemiology of dengue in Salvador-Bahia, 1995-1999].* Revista da Sociedade Brasileira de Medicina Tropical, 2001. **34**(3): p. 269-274.

1396. Tejerina, E.F., F.F. Almeida, and W.R. Almiron, *Bionomics of* Aedes Aegypti *subpopulations (Diptera: Culicidae) from Misiones Province, northeastern Argentina.* Acta Tropica, 2009. **109**(1): p. 45-49.

1397. Teng, H.J. and C.S. Apperson, *Development and survival of immature* Aedes albopictus *and* Aedes triseriatus *(Diptera : Culicidae) in the laboratory: Effects of density, food, and competition on response to temperature.* Journal of Medical Entomology, 2000. **37**(1): p. 40-52.

1398. Teng, H.J., et al., *Emergency Vector Control in a DENV-2 Outbreak in 2002 in Pingtung City, Pingtung County, Taiwan.* Japanese Journal of Infectious Diseases, 2007. **60**(5): p. 271-279.

1399. Teng, H.J., et al., *[The density and larval habitats of dengue vectors in Chungho city].* Kaohsiung Journal of Medical Sciences - Gaoxiong Yi Xue Ke Xue Za Zhi, 1998. **14**(12): p. 754-761.

1400. Teng, H.J., Y.L. Wu, and T.H. Lin, *Mosquito fauna in water-holding containers with emphasis on dengue vectors (Diptera : Culicidae) in Chungho, Taipei County, Taiwan.* Journal of Medical Entomology, 1999. **36**(4): p. 468-472.

1401. Terry , D. *DENGUE - AUSTRALIA (TORRES STRAIT ISLANDS)*. 1997 [cited 1997 3 February].

1402. Tesh, R.B., D.J. Gubler, and L. Rosen, *Variation among goegraphic strains of* Aedes albopictus *in susceptibility to infection with chikungunya virus.* American Journal of Tropical Medicine and Hygiene, 1976. **25**(2): p. 326-335.

1403. Tewari, S.C., et al., *Dengue vector prevalence and virus infection in a rural area in south India.* Tropical Medicine & International Health, 2004. **9**(4): p. 499-507.

1404. Tewari, S.C., et al., *Dengue vector prevalence and virus infection in a rural area in south India.* Tropical Medicine & International Health, 2004. **9**(4): p. 499-507.

1405. Thanispong, K., S. Satfiantriphop, and T. Chareonviriyaphap, *Insecticide resistance of* Aedes Aegypti *and Culex quinquefasciatus in Thailand.* Journal of Pesticide Science, 2008. **33**(4): p. 351-356.

1406. Thaung, U., et al., *Epidemiological features of dengue and chikungunya infections in Burma.* Southeast Asian Journal of Tropical Medicine and Public Health, 1975. **6**(2): p. 276-283.

1407. Thaung, U., C.K. Ming, and M. Thein, *Dengue haemorrhagic fever in Burma.* Southeast Asian Journal of Tropical Medicine and Public Health, 1975. **6**(4): p. 580-591.

1408. Thaung, U., C.K. Ming, and M. Thein, *Insecticide susceptibility of some vector fleas and mosquitoes in Burma.* Southeast Asian Journal of Tropical Medicine and Public Health, 1975. **6**(4): p. 555-561.

1409. Thavara, U., et al., *Dengue vector mosquitos at a tourist attraction, Ko Samui, in 1995.* Southeast Asian Journal of Tropical Medicine and Public Health, 1996. **27**(1): p. 160-163.

1410. Thavara, U., et al., *Dengue vector mosquitos at a tourist attraction, Ko Samui, in 1995.* Southeast Asian Journal of Tropical Medicine and Public Health, 1996. **27**(1): p. 160-163.

1411. Thenmozhi, V., et al., *Natural vertical transmission of dengue viruses in Aedes aegypt in southern India.* Transactions of the Royal Society of Tropical Medicine and Hygiene, 2000. **94**(5): p. 507-507.

1412. Thonnon, J. and G. Chauvancy, *[Evaluation of the immunological and entomological indices of yellow fever in the subprefecture of Tai, Ivory Coast].* Bulletin de la Societe de Pathologie Exotique, 1994. **87**(1): p. 7-10.

1413. Thonnon, J., et al., *Re-emergence of yellow fever in Senegal in 1995.* American Journal of Tropical Medicine and Hygiene, 1998. **59**(1): p. 108-114.

1414. Thonnon, J., et al., *Reemergence of yellow fever in Senegal in 1995.* American Journal of Tropical Medicine and Hygiene, 1998. **59**(1): p. 108-114.

1415. Thonnon, J., et al., *Yellow fever outbreak in Kaffrine, Senegal 1996: epidemiological and entomological findings.* Tropical Medicine & International Health, 1998. **3**(11): p. 872-877.

1416. Thonnon, J., et al., *Yellow fever outbreak in Kaffrine, Senegal 1996: epidemiological and entomological findings.* Tropical Medicine & International Health, 1998. **3**(11): p. 872-877.

1417. Thu, H.M., et al., *Lineage extinction and replacement in dengue type I virus populations are due to stochastic events rather than to natural selection.* Virology, 2005. **336**(2): p. 163-172.

1418. Tidwell, M.A., et al., *Baseline data on* Aedes Aegypti *populations in Santo Domingo, Dominican Republic.* Journal of the American Mosquito Control Association, 1990. **6**(3): p. 514-522.

1419. Tidwell, M.A., et al., *Emergency control of* Aedes Aegypti *in the Dominican Republic using the Scorpion 20 ULV forced-air generator.* Journal of the American Mosquito Control Association, 1994. **10**(3): p. 403-406.

1420. Tien, T.K., et al., Aedes Aegypti *in Ho Chi Minh City (Viet Nam): susceptibility to dengue 2 virus and genetic differentiation.* Transactions of the Royal Society of Tropical Medicine and Hygiene, 1999. **93**(6): p. 581-586.

1421. Tietze, N.S., et al., *Integrated management of waste tire mosquitoes utilizing* Mesocyclops longisetus *(Copepoda: Cyclopidae),* Bacillus thuringiensis *var.* israelensis*,* Bacillus sphaericus*, and methoprene.* Journal of the American Mosquito Control Association, 1994. **10**(3): p. 363-373.

1422. Tikar, S.N., et al., *Susceptibility of immature stages of* Aedes (Stegomyia) aegypti*; vector of dengue and chikungunya to insecticides from India.* Parasitology Research, 2008. **102**(5): p. 907-913.

1423. Tikasingh, E.S. and A. Eustace, *Suppression of* Aedes Aegypti *by predatory Toxorhynchites moctezuma in an island habitat.* Medical and Veterinary Entomology, 1992. **6**(3): p. 272-280.

1424. Tinker, M.E., *[*Aedes-Aegypti *Larval Habitats in Surinam].* Boletin de la Oficina Sanitaria Panamericana, 1976. **80**(5): p. 412-423.

1425. Toledo, M.E., et al., *The unbearable lightness of technocratic efforts at dengue control.* Tropical Medicine & International Health, 2008. **13**(5): p. 728-736.

1426. Toledo, M.E., et al., *Towards active community participation in dengue vector control: results from action research in Santiago de Cuba, Cuba.* Transactions of the Royal Society of Tropical Medicine and Hygiene, 2007. **101**(1): p. 56-63.

1427. Toma, T., S. Sakamoto, and I. Miyagi, *The Seasonal Appearance of* Aedes-Albopictus *in Okinawajima, the Ryukyu Archipelago, Japan.* Mosquito News, 1982. **42**(2): p. 179-183.

1428. Tomori, O. *YELLOW FEVER - LIBERIA (4)*. PROMED, 1995.

1429. Tonn, R.J., et al., *Magnitude of Seasonal Changes in Larval Populations of* Aedes-Aegypti *in Bangkok, Thailand.* Bulletin of the World Health Organization, 1970. **42**(6): p. 943-950.

1430. Tonn, R.J., et al., *Replicate Surveys of Larval Habitats of* Aedes Aegypti *in Relation to Dengue Haemorrhagic Fever in Bangkok, Thailand.* Bulletin of the World Health Organization, 1969. **40**(6): p. 819-829.

1431. Torres, J.R. *DENGUE - VENEZUELA*. 2001 [cited 2001 8 August]; EL UNIVERSAL Vie 3 de Agosto de 2001.

1432. Torres-Estrada, J.L., et al., *Selective oviposition by* Aedes Aegypti *(Diptera: culicidae) in response to Mesocyclops longisetus (Copepoda: Cyclopoidea) under laboratory and field conditions.* Journal of Medical Entomology, 2001. **38**(2): p. 188-192.

1433. Traore-Lamizana, M., et al., *Arbovirus surveillance from 1990 to 1995 in the Barkedji area (Ferlo) of Senegal, a possible natural focus of Rift Valley fever virus.* Journal of Medical Entomology 2001. **38**(4): p. 480-492.

1434. Traore-Lamizana, M., et al., *Surveillance for yellow fever virus in eastern Senegal during 1993.* Journal of Medical Entomology 1996. **33**(5): p. 760-765.

1435. Traore-Lamizana, M., et al., *Dengue 2 outbreak in southeastern Senegal during 1990: virus isolations from mosquitoes (Diptera: Culicidae).* Journal of Medical Entomology 1994. **31**(4): p. 623-627.

1436. Trexler, J.D., et al., *Field and laboratory evaluations of potential oviposition attractants for* Aedes albopictus *(Diptera : Culicidae).* Journal of the American Mosquito Control Association, 2003. **19**(3): p. 228-234.

1437. Trexler, J.D., C.S. Apperson, and C.S. Schal, *Diel oviposition patterns of* Aedes albopictus *(Skuse) and Aedes triseriatus (Say) in the laboratory and the field.* Journal of Vector Ecology, 1997. **22**(1): p. 64-70.

1438. Trout, R.T., et al., *Efficacy of two pyrethroid insecticides applied as barrier treatments for managing mosquito (Diptera : Culicidae) populations in suburban residential properties.* Journal of Medical Entomology, 2007. **44**(3): p. 470-477.

1439. Troyo, A., et al., *Seasonal profiles of* Aedes Aegypti *(Diptera : Culicidae) larval habitats in an urban area of Costa Rica with a history of mosquito control.* Journal of Vector Ecology, 2008. **33**(1): p. 76-88.

1440. Troyo, A., et al., *A geographical sampling method for surveys of mosquito larvae in an urban area using high-resolution satellite imagery.* Journal of Vector Ecology, 2008. **33**(1): p. 1-7.

1441. Trpis, M., *Autogeny in diverse populations of* Aedes Aegypti *from East Africa.* Tropenmedizin und Parasitologie, 1977. **28**(1): p. 77-82.

1442. Trpis, M. and W. Hausermann, *Demonstration of Differential Domesticity of* Aedes-Aegypti *(L) (Diptera, Culicidae) in Africa by Mark Release Recapture.* Bulletin of Entomological Research, 1975. **65**(2): p. 199-208.

1443. Trpis, M. and W. Hausermann, *Genetics of House-Entering Behavior in East-African Populations of* Aedes-Aegypti *(L) (Diptera-Culicidae) and Its Relevance to Speciation.* Bulletin of Entomological Research, 1978. **68**(3): p. 521-532.

1444. Trpis, M. and W. Hausermann, *Dispersal and other population parameters of* Aedes Aegypti *in an African village and their possible significance in epidemiology of vector-borne diseases.* American Journal of Tropical Medicine and Hygiene, 1986. **35**(6): p. 1263-1279.

1445. Trpis, M., W. Hausermann, and G.B. Craig, Jr., *Estimates of population size, dispersal, and longevity of domestic* Aedes Aegypti *aegypti (Diptera: Culicidae) by mark-release-recapture in the village of Shauri Moyo in eastern Kenya.* Journal of Medical Entomology, 1995. **32**(1): p. 27-33.

1446. Tsuzuki, A., et al., *Possibility of false-positive detection for sporozoites in mosquitos (Diptera: Culicidae) by nested polymerase chain reaction using Plasmodium yoelii genomic DNA.* Southeast Asian Journal of Tropical Medicine and Public Health, 2001. **32**(2): p. 275-281.

1447. Tun-Lin, W., T.R. Burkot, and B.H. Kay, *Effects of temperature and larval diet on development rates and survival of the dengue vector* Aedes Aegypti *in north Queensland, Australia.* Medical and Veterinary Entomology, 2000. **14**(1): p. 31-37.

1448. Tun-Lin, W., B.H. Kay, and A. Barnes, *Understanding productivity, a key to* Aedes Aegypti *surveillance.* American Journal of Tropical Medicine and Hygiene, 1995. **53**(6): p. 595-601.

1449. Tun-Lin, W., et al., *Critical examination of* Aedes Aegypti *indices: correlations with abundance.* American Journal of Tropical Medicine and Hygiene, 1996. **54**(5): p. 543-547.

1450. Tun-Lin, W., B.H. Kay, and T.R. Burkot, *Quantitative sampling of immature* Aedes Aegypti *in metal drums using sweep net and dipping methods.* Journal of the American Mosquito Control Association, 1994. **10**(3): p. 390-396.

1451. Tung, Y.C., et al., *Molecular Epidemiology of Dengue Virus Serotype 2 in the Taiwan 2002 Outbreak with Envelope Gene and Nonstructural Protein 1 Gene Analysis.* Kaohsiung Journal of Medical Sciences - Gaoxiong Yi Xue Ke Xue Za Zhi, 2008. **24**(8): p. 398-406.

1452. TunLin, W., B.H. Kay, and A. Barnes, *The premise condition index: A tool for streamlining surveys of Aedes aegypti.* American Journal of Tropical Medicine and Hygiene, 1995. **53**(6): p. 591-594.

1453. Turell, M.J., J.R. Beaman, and R.F. Tammariello, *Susceptibility of selected strains of* Aedes Aegypti *and* Aedes albopictus *(Diptera: Culicidae) to chikungunya virus.* Journal of Medical Entomology, 1992. **29**(1): p. 49-53.

1454. Undeen, A.H. and J.J. Becnel, *A device for monitoring populations of larval mosquitoes in container habitats.* Journal of the American Mosquito Control Association, 1994. **10**(1): p. 101-103.

1455. Urbanelli, S., et al., *Population structure of* Aedes albopictus *(Skuse): the mosquito which is colonizing Mediterranean countries.* Heredity, 2000. **84 ( Pt 3)**: p. 331-337.

1456. Urbinatti, P.R., R.M. de Menezes, and D. Natal, *[*Aedes albopictus *seasonality in a protected area in the city of Sao Paulo, Brazil].* Revista de Saúde Pública, 2007. **41**(3): p. 478-481.

1457. Urdaneta, L., et al., *Detection of dengue viruses in field-caught* Aedes Aegypti *(Diptera: Culicidae) in Maracay, Aragua state, Venezuela by type-specific polymerase chain reaction.* Infection, Genetics and Evolution, 2005. **5**(2): p. 177-84.

1458. Valdes, L., et al., *[Epidemiology of dengue and hemorrhagic dengue in Santiago, Cuba 1997].* Revista Panamericana de Salud Publica, 1999. **6**(1): p. 16-25.

1459. Valerio, L., et al., *Blood-feeding preferences of* Aedes albopictus *(Diptera: Culicidae) in urban and rural settings within the province of Rome, Italy.* Parassitologia, 2008. **50**(1-2): p. 103-104.

1460. van der Sar, A., *An outbreak of dengue haemorrhagic fever on Curacao.* Tropical and Geographical Medicine, 1973. **25**(2): p. 119-129.

1461. Van der Stuyft, P., et al., *Urbanisation of yellow fever in Santa Cruz, Bolivia.* Lancet, 1999. **353**(9164): p. 1558-1562.

1462. Van der Stuyft, P., et al., *Short communication: dengue serotype 2 subtype III ('Jamaica') epidemic in Santa Cruz, Bolivia.* Tropical Medicine & International Health, 1998. **3**(11): p. 857-858.

1463. Van Handel, E., et al., *Plant-Sugar, Glycogen, and Lipid Assay of* Aedes-Aegypti *Collected in Urban Puerto-Rico and Rural Florida.* Journal of the American Mosquito Control Association, 1994. **10**(2): p. 149-153.

1464. Vandehey, R.C., M.G. Leahy, and K.S. Booth, *Analysis of Color Variations in Feral, Peridomestic and Domestic Populations of* Aedes-Aegypti *(L) (Diptera-Culicidae).* Bulletin of Entomological Research, 1978. **68**(3): p. 443-453.

1465. Varejao, J.B., et al., *[*Aedes (Stegomyia) aegypti *(Linnaeus, 1762) breeding sites in native bromeliads in Vitoria City, ES].* Revista da Sociedade Brasileira de Medicina Tropical, 2005. **38**(3): p. 238-240.

1466. Vasconcelos, P.F., et al., *[Dengue epidemic in Fortaleza, Ceara: randomized seroepidemiologic survey].* Revista de Saude Publica, 1998. **32**(5): p. 447-454.

1467. Vasconcelos, P.F., et al., *[A dengue epidemic in Ipupiara and Prado, Bahia. A seroepidemiologic survey].* Revista da Sociedade Brasileira de Medicina Tropical, 2000. **33**(1): p. 61-67.

1468. Vasconcelos, P.F., et al., *[Outbreak of classical fever of dengue caused by serotype 2 in Araguaiana, Tocantins, Brazil].* Revista do Instituto de Medicina Tropical de Sao Paulo, 1993. **35**(2): p. 141-148.

1469. Vasconcelos, P.F., et al., *[Outbreak of classical fever of dengue caused by serotype 2 in Araguaiana, Tocantins, Brazil].* Revista do Instituto de Medicina Tropical de São Paulo, 1993. **35**(2): p. 141-148.

1470. Vasilakis, N., R.B. Tesh, and S.C. Weaver, *Sylvatic dengue virus type 2 activity in humans, Nigeria, 1966.* Emerging Infectious Diseases, 2008. **14**(3): p. 502-504.

1471. Vaughan, J.A., et al., *Passage of ingested Mansonella ozzardi (Spirurida : Onchocercidae) microfilariae through the midgut of* Aedes Aegypti *(Diptera : Culicidae).* Journal of Medical Entomology, 2007. **44**(1): p. 111-116.

1472. Vazeille-Falcoz, M., et al., Aedes albopictus *from Albania: a potential vector of dengue viruses.* Journal of the American Mosquito Control Association, 1999. **15**(4): p. 475-478.

1473. Vazeille-Falcoz, M., et al., *[Oral receptivity of* Aedes Aegypti *formosus from Franceville (Gabon, central Africa) for type 2 dengue virus].* Bulletin De La Societe De Pathologie Exotique, 1999. **92**(5): p. 341-342.

1474. Vazeille-Falcoz, M., et al., *Variation in oral susceptibility to dengue type 2 virus of populations of* Aedes Aegypti *from the islands of Tahiti and Moorea, French Polynesia.* American Journal of Tropical Medicine and Hygiene, 1999. **60**(2): p. 292-299.

1475. Vazquez Ramudo, S., F. Rodhain, and C. Perez-Eid, *[Comparative study of dengue 2 virus replication and isoenzymes in two strains of* Aedes aegypti*].* Bulletin de la Societe de Pathologie Exotique, 1990. **83**(5): p. 628-636.

1476. Vazquez, S., et al., *Kinetics of antibodies in sera, saliva, and urine samples from adult patients with primary or secondary dengue 3 virus infections.* International Journal of Infectious Diseases, 2007. **11**(3): p. 256-262.

1477. Venancio, T.M., et al., *The* Aedes Aegypti *larval transcriptome: a comparative perspective with emphasis on trypsins and the domain structure of peritrophins.* Insect Molecular Biology, 2009. **18**(1): p. 33-44.

1478. Ventura, A.K. and N.J. Ehrenkranz, *Endemic dengue virus infection in Hispaniola. I. Haiti.* Journal of Infectious Diseases, 1976. **134**(5): p. 436-441.

1479. Ventura, A.K. and N.J. Ehrenkranz, *Endemic dengue virus infection in Hispaniola. I. Haiti.* Journal of Infectious Diseases, 1976. **134**(5): p. 436-441.

1480. Vezzani, D., et al., *Detailed assessment of microhabitat suitability for* Aedes Aegypti *(Diptera: Culicidae) in Buenos Aires, Argentina.* Acta Tropica, 2005. **95**(2): p. 123-131.

1481. Vezzani, D. and N. Schweigmann, *Suitability of containers from different sources as breeding sites of* Aedes Aegypti *(L.) in a cemetery of Buenos Aires City, Argentina.* Memorias Do Instituto Oswaldo Cruz, 2002. **97**(6): p. 789-792.

1482. Vezzani, D., S.M. Velazquez, and N. Schweigmann, *Seasonal pattern of abundance of* Aedes Aegypti *(Diptera: Culicidae) in Buenos Aires City, Argentina.* Memorias Do Instituto Oswaldo Cruz, 2004. **99**(4): p. 351-356.

1483. Vicens, R., et al., *[Yellow fever epidemic in the extreme North of Cameroon in 1990: first yellow fever virus isolation in Cameroon].* Bulletin of the World Health Organization, 1993. **71**(2): p. 173-176.

1484. Victor, T.J., *Detection of dengue viral infections in Aedes mosquitoes: An essential tool for epidemiological surveillance.* Indian Journal of Medical Research, 2009. **129**(6): p. 634-636.

1485. Victor, T.J., et al., *Laboratory-based dengue fever surveillance in tamil nadu, India.* Indian Journal of Medical Research, 2007. **126**(2): p. 112-115.

1486. Victor, T.J., et al., *Dengue fever outbreaks in two villages of Dharmapuri district in Tamil Nadu.* Indian Journal of Medical Research, 2002. **116**: p. 133-139.

1487. Vieira, D.S., et al., *Characterization of dengue virus serotype 1 in epidemics in Porto Velho, Rondonia, in 2001-2003.* Revista da Sociedade Brasileira de Medicina Tropical, 2007. **40**(3): p. 268-271.

1488. Villeneuve, L., et al., *[Dengue in Martinique in 1995-1996].* Medecine Tropicale, 1998. **58**(2): p. 145-148.

1489. Vongtangswad, S., C. Tirabutana, and B. Thongkum, *The Biological-Control of* Aedes-Aegypti *on Sa-Med Island, Rayong Province by Means of Toxorhynchites-Splendens, a Predatory Mosquito Larva.* Journal of the Medical Association of Thailand, 1983. **66**: p. 8-12.

1490. Vonwinde.Dl, D.A. Eliason, and H.F. Schoof, *Efficacy of Carbaryl, Propoxur, Abate and Methoxychlor as Larvicides against Field Infestations of Aedes-Aegypti.* Mosquito News, 1971. **31**(1): p. 91-95.

1491. Vorms-le Morvan, J., M.C. Vazeille-Falcoz, and F. Rodhain, *[Experimental infection of* Aedes albopictus *mosquitoes by a spiroplasma strain isolated from Culex annulus in Taiwan].* Bulletin De La Societe De Pathologie Exotique, 1991. **84**(1): p. 15-24.

1492. Vu, S.N., et al., *Elimination of dengue by community programs using Mesocyclops(Copepoda) against* Aedes Aegypti *in central Vietnam.* American Journal of Tropical Medicine and Hygiene, 2005. **72**(1): p. 67-73.

1493. Vythilingam, I., et al., *Isolation of Japanese encephalitis virus from mosquitoes collected in Sabak Bernam, Selangor, Malaysia in 1992.* Journal of the American Mosquito Control Association, 1995. **11**(1): p. 94-98.

1494. Wagatsuma, Y., et al., *Dengue fever outbreak in a recreation club, Dhaka, Bangladesh.* Emerging Infectious Dieseases, 2004. **10**(4): p. 747-750.

1495. Wagatsuma, Y., et al., *Dengue fever outbreak in a recreation club, Dhaka, Bangladesh.* Emerging Infectious Diseases, 2004. **10**(4): p. 747-750.

1496. Wagbatsoma, V.A. and O. Ogbeide, *Towards malaria control in Nigeria: a qualitative study on the population of mosquitoes.* Journal of the Royal Society of Health, 1995. **115**(6): p. 363-365.

1497. Walker, E.D., G.F. OMeara, and W.T. Morgan, *Bacterial abundance in larval habitats of* Aedes albopictus *(Diptera: Culicidae) in a Florida cemetery.* Journal of Vector Ecology, 1996. **21**(2): p. 173-177.

1498. Wallace, H.G., et al., *Dengue hemorrhagic fever in Malaysia: the 1973 epidemic.* Southeast Asian Journal of Tropical Medicine and Public Health, 1980. **11**(1): p. 1-13.

1499. Wallace, H.G., et al., *Dengue hemorrhagic fever in Malaysia: the 1973 epidemic.* Southeast Asian Journal of Tropical Medicine and Public Health, 1980. **11**(1): p. 1-13.

1500. Wallis, G.P., et al., *Selection for susceptibility and refractoriness of* Aedes Aegypti *to oral infection with yellow fever virus.* American Journal of Tropical Medicine and Hygiene, 1985. **34**(6): p. 1225-1231.

1501. Wallis, G.P. and W.J. Tabachnick, *Genetic analysis of rock hole and domestic* Aedes Aegypti *on the Caribbean island of Anguilla.* Journal of the American Mosquito Control Association, 1990. **6**(4): p. 625-630.

1502. Wallis, G.P., W.J. Tabachnick, and J.R. Powell, *Macrogeographic Genetic-Variation in a Human Commensal -* Aedes-Aegypti*, the Yellow-Fever Mosquito.* Genetical Research, 1983. **41**(3): p. 241-258.

1503. Wallis, G.P., W.J. Tabachnick, and J.R. Powell, *Genetic heterogeneity among Caribbean populations of* Aedes aegypti*.* American Journal of Tropical Medicine and Hygiene, 1984. **33**(3): p. 492-498.

1504. Wang, C.H., et al., *Integrated control of the dengue vector* Aedes Aegypti *in Liu-Chiu village, Ping-Tung County, Taiwan.* Journal of the American Mosquito Control Association, 2000. **16**(2): p. 93-99.

1505. Wang, C.H., J.S. Hwang, and J.R. Lay, *[Preliminary study on the biological control of dengue vectors by fish in Liouchyou Prefecture, Pingtung County, Taiwan].* Kaohsiung Journal of Medical Sciences - Gaoxiong Yi Xue Ke Xue Za Zhi, 1990. **6**(7): p. 382-388.

1506. Warren, A.M. and J.M. Crampton, *The* Aedes Aegypti *genome: complexity and organization.* Genetic Research, 1991. **58**(3): p. 225-232.

1507. Warren, A.M., M.A. Hughes, and J.M. Crampton, *Zebedee: a novel copia-Ty1 family of transposable elements in the genome of the medically important mosquito Aedes aegypti.* Molecular & General Genetics, 1997. **254**(5): p. 505-513.

1508. Washburn, J.O. and E.U. Hartmann, *Could* Aedes albopictus *(Diptera: Culicidae) become established in California tree holes?* Journal of Medical Entomology, 1992. **29**(6): p. 995-1005.

1509. Waterman, S.H., et al., *Dengue transmission in two Puerto Rican communities in 1982.* American Journal of Tropical Medicine and Hygiene, 1985. **34**(3): p. 625-632.

1510. Watson, T.M. and B.H. Kay, *Vector competence of Aedes notoscriptus (Diptera: Culicidae) for Barmah Forest virus and of this species and* Aedes Aegypti *(Diptera: Culicidae) for dengue 1-4 viruses in Queensland, Australia.* Journal of Medical Entomology, 1999. **36**(4): p. 508-514.

1511. Watts, D.M., et al., *Failure of secondary infection with American genotype dengue 2 to cause dengue haemorrhagic fever.* Lancet, 1999. **354**(9188): p. 1431-1434.

1512. Weiland, H.T., M.C. Williams, and B. Hull, *Serologic survey of dengue and other arboviruses in Curacao and Aruba, 1973.* Bulletin of the Pan American Health Organization, 1978. **12**(2): p. 134-142.

1513. Weng, M.H., et al., *Isolation of Japanese encephalitis virus from mosquitoes collected in Northern Taiwan between 1995 and 1996.* Journal of Microbiology Immunology and Infection, 1999. **32**(1): p. 9-13.

1514. Weng, M.H., et al., *Susceptibility of three laboratory strains of* Aedes albopictus *(Diptera : Culicidae) to Japanese encephalitis virus from Taiwan.* Journal of Medical Entomology, 1997. **34**(6): p. 745-747.

1515. Whelan, P. *AEDES MOSQUITOES IMPORTED - AUSTRALIA (NT) (02)*. PROMED, 2000.

1516. Whelan, P. *AEDES MOSQUITOES IMPORTED - AUSTRALIA (NT) (03)*. PROMED, 2000.

1517. Williams, C.R., et al., *Rapid Estimation of* Aedes Aegypti *Population Size Using Simulation Modeling, with a Novel Approach to Calibration and Field Validation.* Journal of Medical Entomology, 2008. **45**(6): p. 1173-1179.

1518. Williams, C.R., et al., *The Allee effect in site choice behaviour of egg-laying dengue vector mosquitoes.* Tropical Biomedicine, 2008. **25**(2): p. 140-144.

1519. Willis, F.S. and R.S. Nasci, Aedes albopictus *(Diptera: Culicidae) population density and structure in southwest Louisiana.* Journal of Medical Entomology, 1994. **31**(4): p. 594-599.

1520. Wills, W., et al., *Hepatitis B surface antigen (Australia antigen) in mosquitoes collected in Senegal, West Africa.* American Journal of Tropical Medicine and Hygiene, 1976. **25**(1): p. 186-190.

1521. Winch, P.J., et al., *Variation in* Aedes Aegypti *larval indices over a one year period in a neighborhood of Merida, Yucatan, Mexico.* Journal of the American Mosquito Control Association, 1992. **8**(2): p. 193-195.

1522. Wirth, M.C. and G.P. Georghiou, *Selection and characterization of temephos resistance in a population of* Aedes Aegypti *from Tortola, British Virgin Islands.* Journal of the American Mosquito Control Association, 1999. **15**(3): p. 315-320.

1523. Womack, M.L., *Distribution, abundance and bionomics of* Aedes albopictus *in southern Texas.* Journal of the American Mosquito Control Association, 1993. **9**(3): p. 367-369.

1524. Wong, J., et al., *SSCP analysis of scnDNA for genetic profiling of Aedes aegypti.* American Journal of Tropical Medicine and Hygiene, 2008. **79**(4): p. 511-517.

1525. Woodall, J. *DENGUE/DHF - CUBA (09)*. [http://www.cubaweb.cu/granma/julio4/28julio8i.html] 1997 [cited 1997 2 August]; Granma International 1997. Havana, Cuba.

1526. Xu, G.Z., et al., *An outbreak of dengue virus serotype 1 infection in Cixi, Ningbo, People's Republic of China, 2004, associated with a traveler from Thailand and high density of* Aedes albopictus*.* American Journal of Tropical Medicine and Hygiene, 2007. **76**(6): p. 1182-1188.

1527. Xue, R.D. and D.R. Barnard, *Human host avidity in* Aedes albopictus*: influence of mosquito body size, age, parity, and time of day.* Journal of the American Mosquito Control Association, 1996. **12**(1): p. 58-63.

1528. Xue, R.D. and D.R. Barnard, *Diel patterns of pupation, emergence, and oviposition in a laboratory population of* Aedes albopictus*.* Journal of the American Mosquito Control Association, 1997. **13**(2): p. 205-207.

1529. Xue, R.D., D.R. Barnard, and A. Ali, *Laboratory and field evaluation of insect repellents as oviposition deterrents against the mosquito* Aedes albopictus*.* Medical and Veterinary Entomology, 2001. **15**(2): p. 126-131.

1530. Xue, R.D., D.R. Barnard, and C.E. Schreck, *Influence of body size and age of* Aedes albopictus *on human host attack rates and the repellency of deet.* Journal of the American Mosquito Control Association, 1995. **11**(1): p. 50-53.

1531. Yaicharoen, R., et al., *Characterization of deltamethrin resistance in field populations of* Aedes Aegypti *in Thailand.* Journal of Vector Ecology 2005. **30**(1): p. 144-150.

1532. Yamao, T., et al., *Novel virus discovery in field-collected mosquito larvae using an improved system for rapid determination of viral RNA sequences (RDV ver4.0).* Archives of Virology, 2009. **154**(1): p. 153-158.

1533. Yan, G., D.D. Chadee, and D.W. Severson, *Evidence for genetic hitchhiking effect associated with insecticide resistance in* Aedes aegypti*.* Genetics, 1998. **148**(2): p. 793-800.

1534. Yan, G., et al., *Population genetics of the yellow fever mosquito in Trinidad: comparisons of amplified fragment length polymorphism (AFLP) and restriction fragment length polymorphism (RFLP) markers.* Molecular Ecology, 1999. **8**(6): p. 951-963.

1535. Yan, G., D.W. Severson, and B.M. Christensen, *Costs and benefits of mosquito refractoriness to malaria parasites: Implications for genetic variability of mosquitoes and genetic control of malaria.* Evolution, 1997. **51**(2): p. 441-450.

1536. Yap, H.H., *Distribution of* Aedes Aegypti *(Linnaeus) and* Aedes albopictus *(Skuse) in small towns and villages of Penang Island, Malaysia--an ovitrap survey.* Southeast Asian Journal of Tropical Medicine and Public Health, 1975. **6**(4): p. 519-524.

1537. Yap, H.H., *Effectiveness of soap formulations containing deet and permethrin as personal protection against outdoor mosquitoes in Malaysia.* Journal of the American Mosquito Control Association, 1986. **2**(1): p. 63-67.

1538. Yap, H.H., et al., *Field efficacy of a new repellent, KBR 3023, against* Aedes albopictus *(SKUSE) and* Culex quinquefasciatus *(SAY) in a tropical environment.* Journal of Vector Ecology, 1998. **23**(1): p. 62-68.

1539. Yasuno, M. and R.J. Tonn, *Bionomics of Toxorhynchites-Splendens in Larval Habitats of* Aedes-Aegypti *in Bangkok, Thailand.* Bulletin of the World Health Organization, 1970. **43**(5): p. 762-766.

1540. Yasuno, M. and R.J. Tonn, *Study of Biting Habits of* Aedes-Aegypti *in Bangkok-Thailand.* Bulletin of the World Health Organization, 1970. **43**(2): p. 319-325.

1541. Yee, D.A., M.G. Kaufman, and S.A. Juliano, *The significance of ratios of detritus types and micro-organism productivity to competitive interactions between aquatic insect detritivores.* Journal of Animal Ecology, 2007. **76**(6): p. 1105-1115.

1542. Yee, D.A., B. Kesavaraju, and S.A. Juliano, *Larval feeding behavior of three co-occurring species of container mosquitoes.* Journal of Vector Ecology, 2004. **29**(2): p. 315-322.

1543. Yee, D.A., B. Kesavaraju, and S.A. Juliano, *Direct and indirect effects of animal detritus on growth, survival, and mass of invasive container mosquito* Aedes albopictus *(Diptera : Culicidae).* Journal of Medical Entomology, 2007. **44**(4): p. 580-588.

1544. Yong, H.S., et al., *Multiple glucose phosphate isomerase alleles in* Aedes albopictus *(Diptera:Culicidae) from Peninsular Malaysia.* Comparative Biochemistry and Physiology. B, Comparative Biochemistry, 1982. **73**(2): p. 265-267.

1545. Yong, H.S., et al., *Multiple alleles and low variability of glycerol-3-phosphate dehydrogenase in* Aedes albopictus *(Diptera: Culicidae) from Peninsular Malaysia.* Comparative Biochemistry and Physiology. B, Comparative Biochemistry, 1983. **75**(1): p. 43-45.

1546. Yoosuf, A.A., et al., *First report of chikungunya from the Maldives.* Transactions of the Royal Society of Tropical Medicine and Hygiene, 2009. **103**(2): p. 192-196.

1547. Young, E.C., *Mosquitoes of Rarotonga, Cook Islands: a survey of breeding sites.* New Zealand Journal of Zoology, 2007. **34**(1): p. 57-61.

1548. Zamburlini, R., *[*Aedes albopictus *(Skuse) (Diptera, Culicidae) in eastern Veneto].* Parassitologia, 1994. **36**(3): p. 301-304.

1549. Zeidler, J.D., et al., *Dengue virus in* Aedes Aegypti *larvae and infestation dynamics in Roraima, Brazil.* Revista de Saude Publica, 2008. **42**(6): p. 986-991.

1550. Zeller, H.G., et al., *Enzootic activity of Rift Valley fever virus in Senegal.* American Journal of Tropical Medicine and Hygiene, 1997. **56**(3): p. 265-272.

1551. Zequi, J.A.C., J. Lopes, and I.M. Medri, *[Immature specimens of Culicidae (Diptera) found in installed recipients in forest fragments in the Londrina, Parana, Brazil].* Revista Brasileira De Zoologia, 2005. **22**(3): p. 656-661.

1552. Zeze, D.G. and A.A. Koffi, *[Stegomyian indices and epidemiological status of yellow fever in a rural area of the Ivory Coast].* Medecine Tropicale, 1994. **54**(4): p. 324-330.

1553. Zhang, L.Y. and C.L. Lei, *Evaluation of sticky ovitraps for the surveillance of* Aedes (Stegomyia) albopictus *(Skuse) and the screening of oviposition attractants from organic infusions.* Annals of Tropical Medicine and Parasitology, 2008. **102**(5): p. 399-407.

1554. Zhang, S. and G.M. He, *[Studies on the susceptibility among geographic strains of* Aedes albopictus *in China to infection with dengue viruses].* Chinese Journal of Epidemiology - Zhonghua Liu Xing Bing Xue Za Zhi 1989. **10**(6): p. 348-351.

1555. Zuo, L. and L.P. Shu, *Isolation, identification, and phylogenetic analysis of a dengue virus strain from* Aedes albopictus *collected in Mawei town in Guizhou Province, China.* Chinese Medical Journal, 2004. **117**(12): p. 1847-1849.

1556. Zytoon, E.M., et al., *Susceptibility of* Aedes albopictus *mosquitoes (Oahu strain) to infection with Dirofilaria immitis.* Kobe Journal of Medical Sciences, 1992. **38**(5): p. 289-305.
