# Supplementary figures and images for: Global temperature constraints on Aedes aegypti and Ae. albopictus persistence and competence for dengue virus transmission
Source: Parasit Vectors. 2014 Jul 22;7:338. doi: 10.1186/1756-3305-7-338 (PMC4148136; doi:10.1186/1756-3305-7-338)

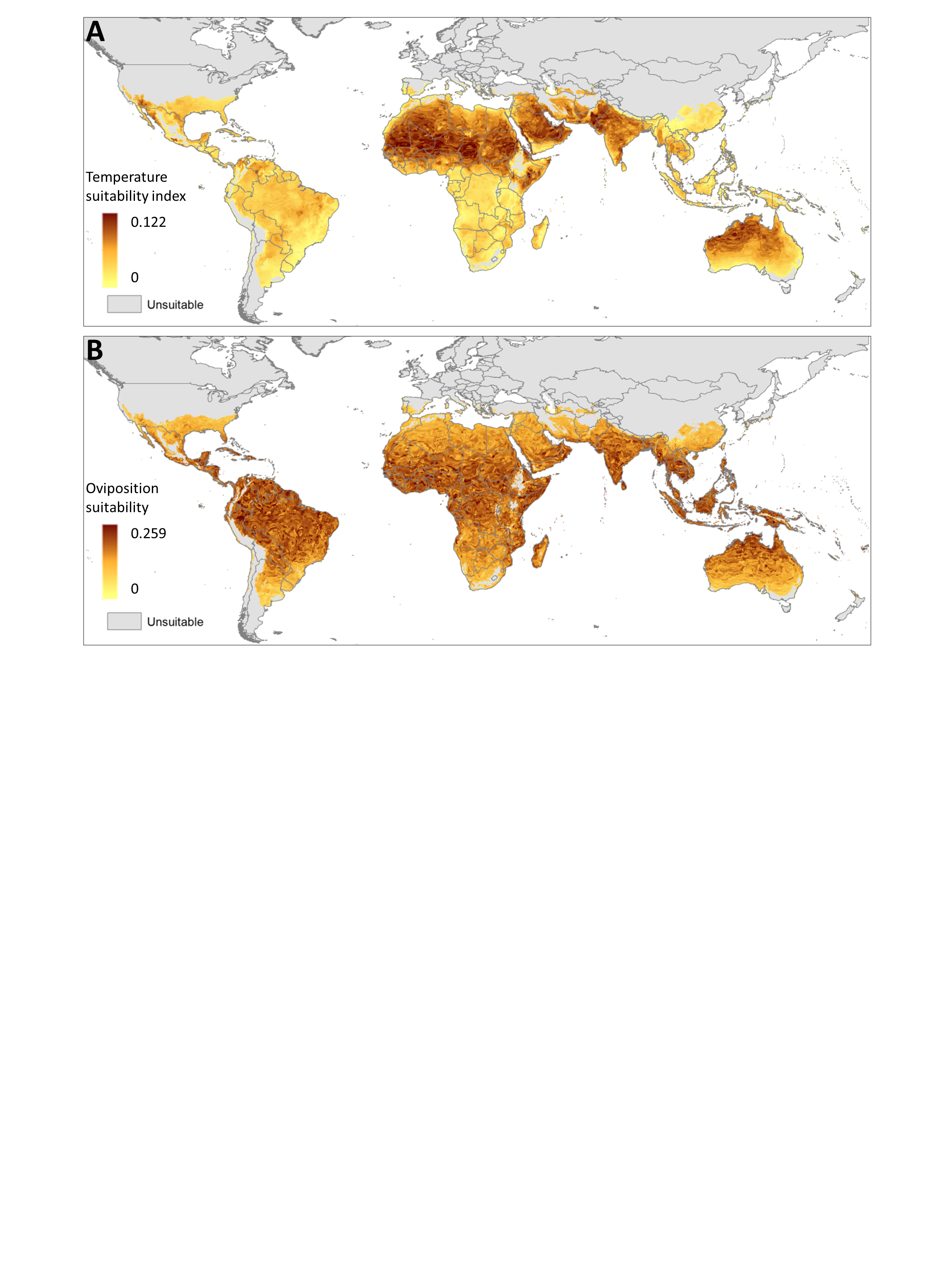

Supplement: Additional file 9 — Ae. aegypti model prediction uncertainty for the temperature suitability index (A) and oviposition suitability (B). The output shows the interquartile range in predictions presented in maps 3A and 3D. [file 1756-3305-7-338-S9.tiff]

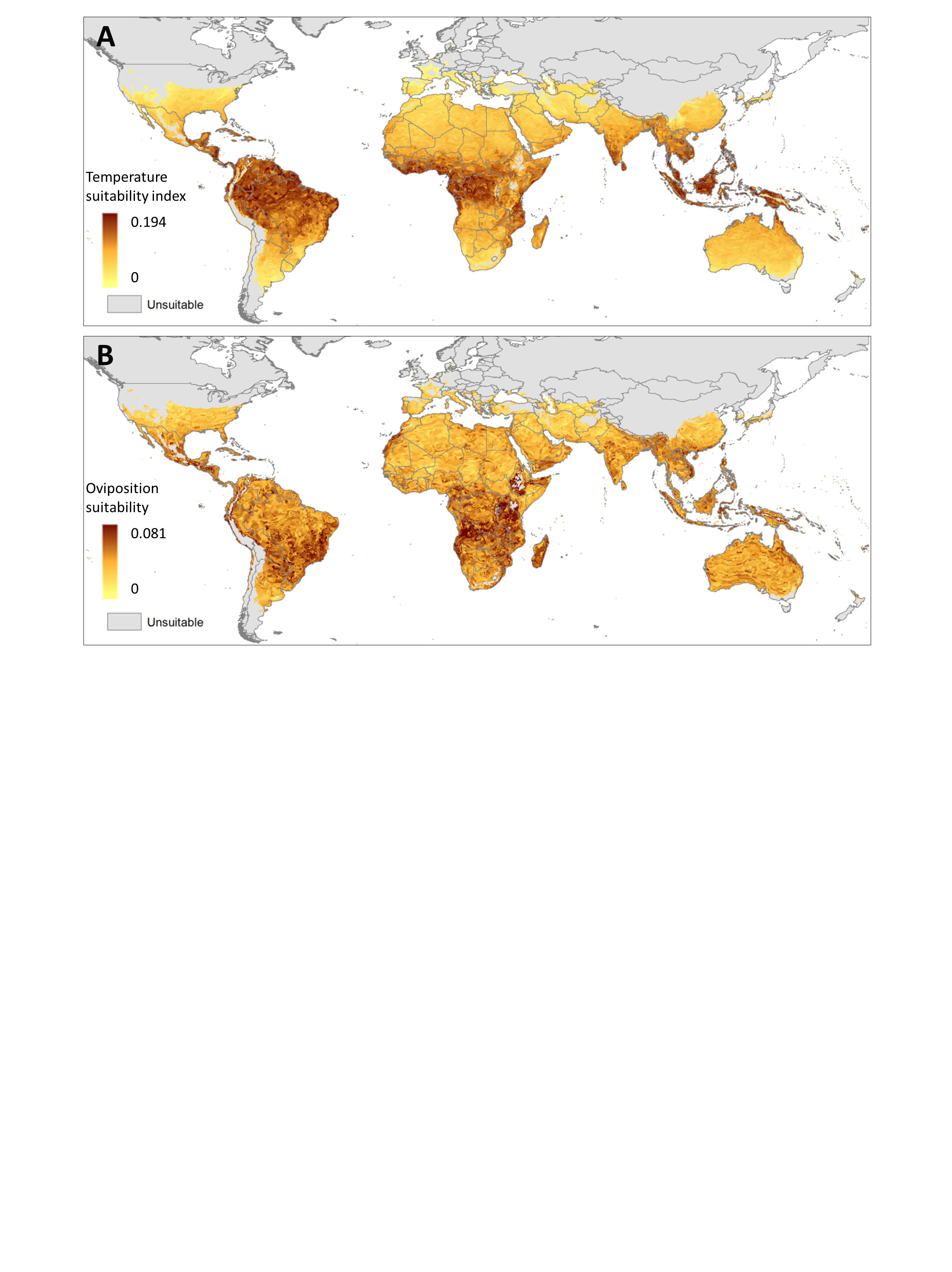

Supplement: Additional file 10 — Ae. albopictus model prediction uncertainty for the temperature suitability index (A) and oviposition suitability (B). The output shows the interquartile range in predictions presented in maps 4A and 4D. [file 1756-3305-7-338-S10.tiff]
